# Supplementary material for: The Genetic Architecture of the Human Corpus Callosum and its Subregions
Source: Nat Commun. 2025 Nov 4;16:9708. doi: 10.1038/s41467-025-64791-3 (PMC12586663; doi:10.1038/s41467-025-64791-3)
Supplement: Supplementary file 1 — Supplementary Information [file 41467_2025_64791_MOESM1_ESM.pdf]

Supplementary Information for: The Genetic Architecture of the Human Corpus Callosum and its Subregions

Ravi R. Bhatt,[1*] Shruti P. Gadewar,[1*] Ankush Shetty,[1] Iyad Ba Gari,[1] Elizabeth Haddad,[1] Shayan Javid,[1] Abhinaav Ramesh,[1] Elnaz Nourollahimoghadam,[1] Alyssa H. Zhu,[1] Christaan de Leeuw,[2] Paul M. Thompson,[1] Sarah E. Medland,[3] Neda Jahanshad[1]

[1]Imaging Genetics Center, Mark and Mary Stevens Neuroimaging and Informatics Institute, Keck School of Medicine, University of Southern California, Marina del Rey, CA, USA

[2]Department of Complex Trait Genetics, Centre for Neurogenomics and Cognitive Research, VU University, Amsterdam, The Netherlands

[3]Psychiatric Genetics, QIMR Berghofer Medical Research Institute, Brisbane 4006, Australia

*Co-first authors

Table of contents

Supplementary Information..2
1. Supplementary Figure 1: *Data Augmentation Techniques*..2
2. Supplementary Figure 2: *Midsagittal corpus callosum segmentation via SMACC*........... 3
3. Supplementary Figure 3: *Reliability of SMACC Metrics in a Test-Retest Dataset*.............3
4. Supplementary Figure 4: *UK Biobank MDS in European Ancestry Individuals*................ 4
5. Supplementary Figure 5: *Adolescent Behavioral Cognitive Development study MDS in European Ancestry Individuals*..5

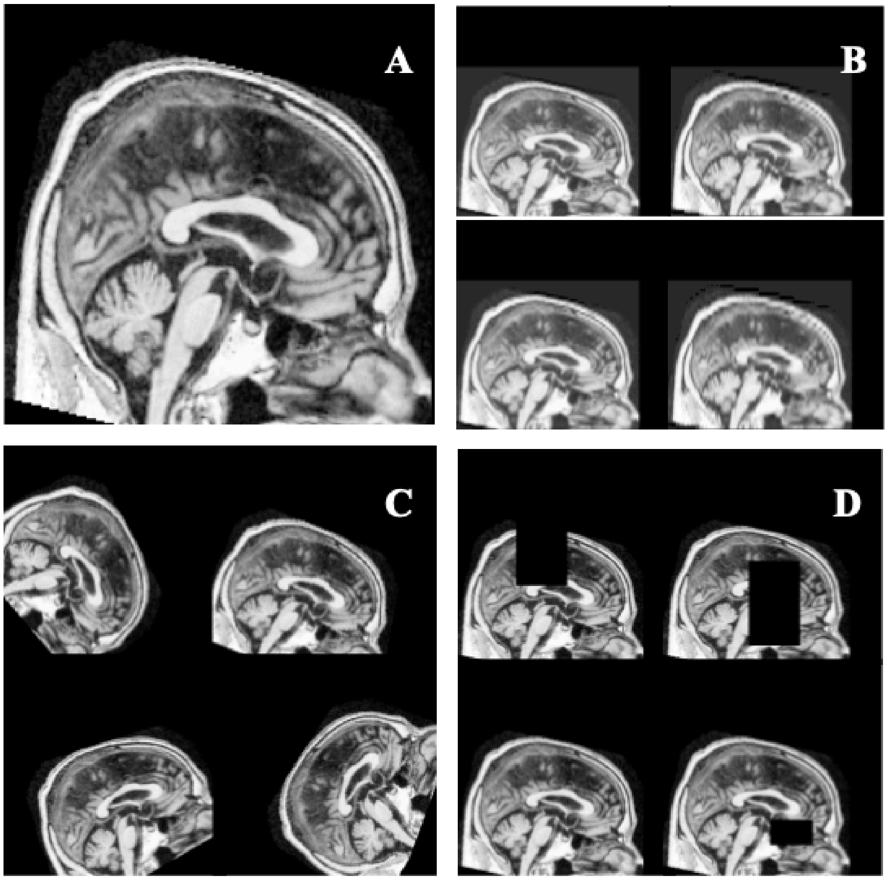

Supplementary Figure 1: Data augmentation techniques - **A.** A T1w MR image **B**. Downsampled MR images by a factor of 2, 3, 4 and 5. **C**. MR images rotated in increments of 15 degrees. **D**. Black boxes of various width X height ((100 X 60), (60 X 100), (50 X 30), (30 X 50)) were added at random locations in the midCC slice to imitate partial agenesis cases.

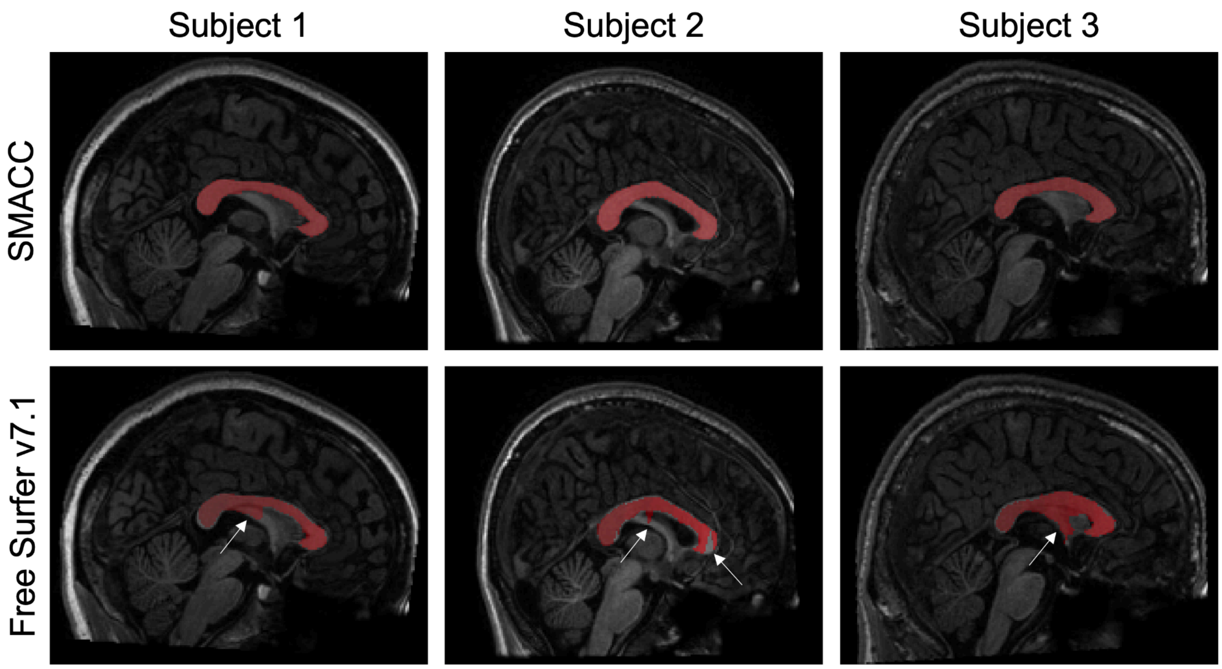

Supplementary Figure 2: **Midsagittal corpus callosum segmentation via SMACC:** Examples of midsagittal corpus callosum (midCC) segmentation from three different participants (left to right) using our SMACC tool[1] (top) and FreeSurfer (bottom) in Hangzhou Normal University (HNU) dataset[2].

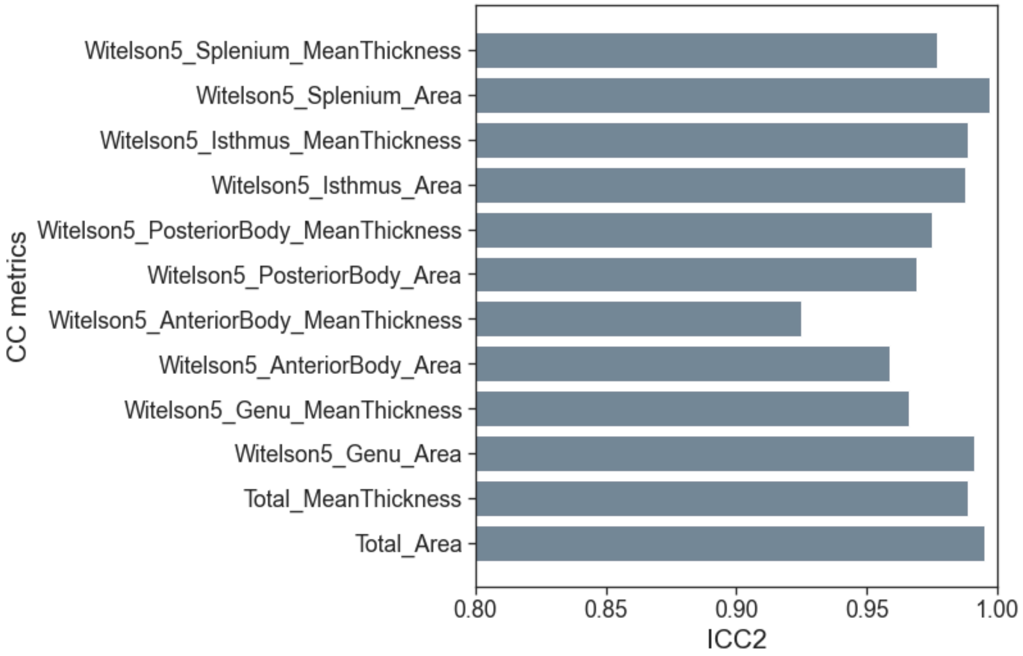

Supplementary Figure 3: **Reliability of SMACC metrics in a test-retest dataset:** Average intraclass correlation (ICC) coefficients for different CC metrics between sessions for all subjects

in Hangzhou Normal University (HNU) dataset[2]. Higher ICC shows that our segmentations across all the sessions for a subject are very similar and hence highly reliable.

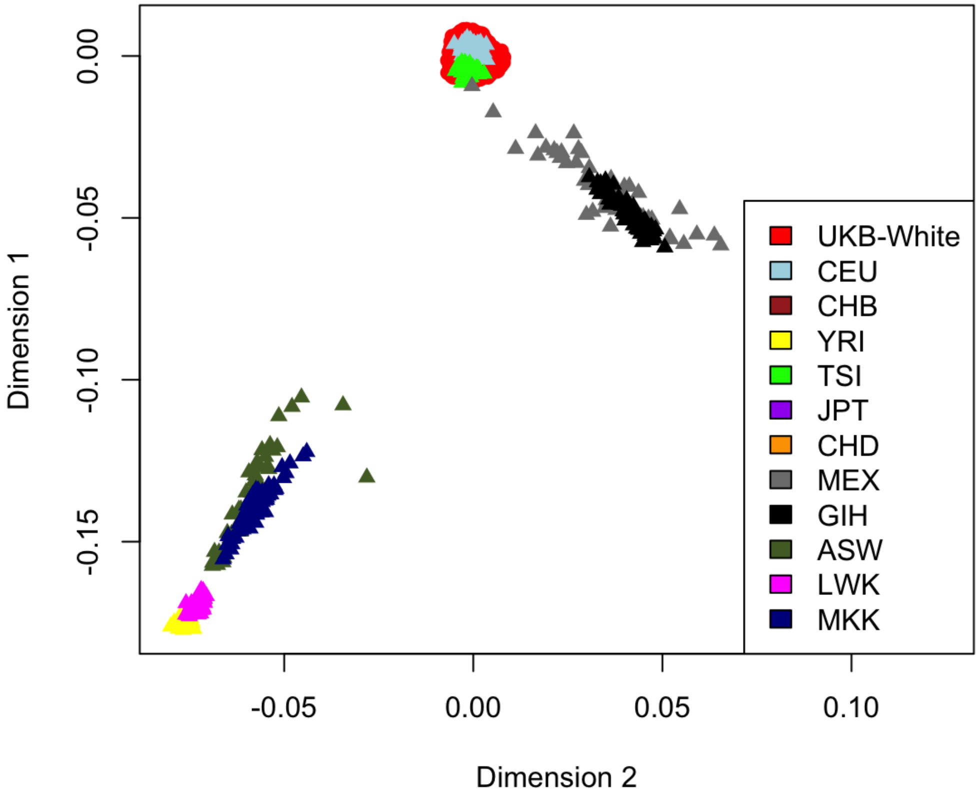

Supplementary Figure 4: UK Biobank MDS in European Ancestry Individuals: European individuals (red points) in UK Biobank[3] as defined via MDS. UK Biobank sample is overlaid with the HapMap3 data release[4]. CEU individuals represent Utah residents with Northern and Western European ancestry from the Centre d′Etudes du Polymorphisme Humain (CEPH) collection. TSI individuals represent Toscani in Italy.

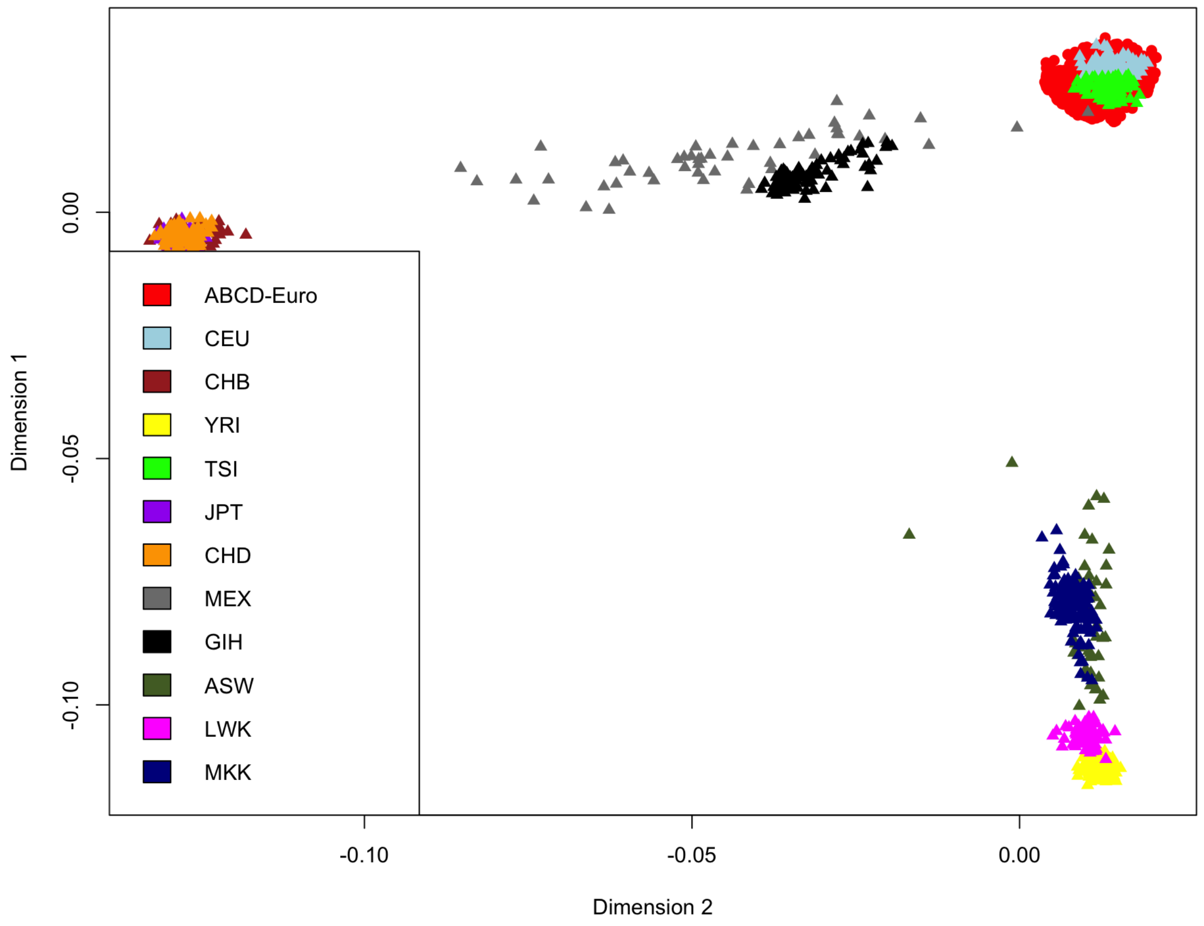

Supplementary Figure 5: **Adolescent Behavioral Cognitive Development study MDS in European Ancestry Individuals:** European Individuals (red points) in the Adolescent Behavioral Cognitive Development study (ABCD) as defined via MDS[5]. The ABCD sample is overlaid with the HapMap3 data release[4]. CEU individuals represent Utah residents with Northern and Western European ancestry from the Centre d'Etudes du Polymorphisme Humain (CEPH) collection. TSI individuals represent Toscani in Italy.

References

1. Gadewar, S. P. *et al.* A Comprehensive Corpus Callosum Segmentation Tool for Detecting Callosal Abnormalities and Genetic Associations from Multi Contrast MRIs. *Conf. Proc. IEEE Eng. Med. Biol. Soc.* **2023**, 1–4 (2023).
2. Gorgolewski, K. J. *et al.* A high resolution 7-Tesla resting-state fMRI test-retest dataset with cognitive and physiological measures. *Sci Data* **2**, 140054 (2015).

3. Bycroft, C. *et al.* The UK Biobank resource with deep phenotyping and genomic data. *Nature* **562**, 203–209 (2018).

4. International HapMap 3 Consortium *et al.* Integrating common and rare genetic variation in diverse human populations. *Nature* **467**, 52–58 (2010).

5. Volkow, N. D. *et al.* The conception of the ABCD study: From substance use to a broad NIH collaboration. *Dev. Cogn. Neurosci.* **32**, 4–7 (2018).
