# Supplementary material for: The Genetic Architecture of the Human Corpus Callosum and its Subregions
Source: Nat Commun. 2025 Nov 4;16:9708. doi: 10.1038/s41467-025-64791-3 (PMC12586663; doi:10.1038/s41467-025-64791-3)
Supplement: Supplementary file 2 — Description of Additional Supplementary Files [file 41467_2025_64791_MOESM2_ESM.pdf]

Description of Additional Supplementary Files

Supplementary Data 1: Distinct genomic loci associated with CC Morphometry
The most strongly associated lead SNPs in independent genomic loci are shown after merging regions < 250 KB apart into a single locus. chr = chromosome; pos = base pair position; start = start of locus base-pair position; end = end of locus base-pair position; Allele1 = effect allele; Allele2 = non-effect allele; Freq1 = weighted average of frequency for allele 1 across all studies; FreqSE = corresponding standard error for allele frequency estimatel; MinFreq = minimum frequency for allele 1 across all studies; MaxFreq = maximum frequency for allele 1 across all studies; Effect = overall estimated effect size for allele 1; Std = overall standard error for effect size estimate; P = meta-analysis p-value (two-sided); Direction = summary of effect direction for each study, with one '+' or '-' per study; TotalN = total sample size including all studies; nearestGene = nearest gene of the SNP based on ANNOVAR annotations; dist = distance to the nearest gene. SNPs which are locating in the gene body or 1kb up- or down-stream of TSS or TES have 0; func = functional consequence of the SNP on the gene obtained from ANNOVAR; CADD = Combined Annotation-Dependent depletion score, which predict how deleterious the SNP effect is on protein structure/function (higher scores indicate more deleterious); RDB = RegulomeDB scores predict likelihood of regulatory functionality (lower scores indicate higher likelihood); minChrState = minimum chromatin state across 127 tissue types (lower scores indicate more open chromatin); commonChrState = most common chromatin state in 127 tissue types; uniqID = Unique ID of SNPs consists of chr:position:allele1:allele2 where alleles are alphabetically ordered; nSNPs = Number of unique candidate SNPs in the genomic locus, including non-GWAS-tagged SNPs; nGWASSNPs : Number of the GWAS-tagged candidate SNPs within the genomic locus; nIndSigSNPs : Number of the independent significant SNPs in the genomic locus; IndSigSNPs : rsID of independent significant SNPs in the genomic locus; nLeadSNPs : The number of lead SNPs in the genomic locus; LeadSNPs : rsID of lead SNPs in the genomic locus, EU Effect in Non-EU Range: Wheather the effect of the prinicpal European results falls within the 95% confidence interval of the Non-European results. Effect Direction Condorance: Whether the effect direction from both cohorts are matching direction.

Supplementary Data 2. Independent significant SNPs associated with CC Morphometry
The most strongly associated lead SNPs in independent genomic loci are shown after merging regions < 250 KB apart into a single locus. chr = chromosome; pos = base pair position; start = start of locus base-pair position; end = end of locus base-pair position; Allele1 = effect allele; Allele2 = non-effect allele; Freq1 = weighted average of frequency for allele 1 across all studies; FreqSE = corresponding standard error for allele frequency estimatel; MinFreq = minimum frequency for allele 1 across all studies; MaxFreq = maximum frequency for allele 1 across all studies; Effect = overall estimated effect size for allele 1; Std = overall standard error for effect size estimate; P = meta-analysis p-value (two-sided); Var % = percent variance explained; Direction = summary of effect direction for each study, with one '+' or '-' per study; TotalN = total sample size including all studies; nearestGene = nearest gene of the SNP based on ANNOVAR annotations; dist = distance to the nearest gene. SNPs which are locating in the gene body or 1kb up- or down-stream of TSS or TES have 0; func = functional consequence of the SNP on the gene obtained from ANNOVAR; CADD = Combined Annotation-Dependent depletion score, which predict how deleterious the SNP effect is on protein structure/function (higher scores indicate more deleterious); RDB = RegulomeDB scores predict likelihood of regulatory functionality (lower scores indicate higher likelihood); minChrState = minimum chromatin state across 127 tissue types (lower scores indicate more open chromatin); commonChrState = most common chromatin state in 127 tissue types; uniqID = Unique ID of SNPs consists of chr:position:allele1:allele2 where alleles are alphabetically ordered

Supplementary Data 3. Results from MAGMA gene-based association analysis for total area of the CC

Results from MAGMA gene-based association analysis. Exome-wide significant (after Bonferroni correction) genes associated with CC phenotypes. Chromosome (CHR), number of SNPs in the genes (N SNPS) and number of relevant parameters used in the model (N PARAM), two-sided association P-values (P) are shown. The Z-statistic of the gene was used as the measure of association in the gene-level analysis. All GWAS results were input into MAGMA with the experiment-wide Bonferroni threshold correcting for all traits at 6.13e-9. Bonferroni threshold for each traits analysis was set in MAGMA again at P = 0.05/18191 = 2.749e-6 by correcting for every gene tested.

Supplementary Data 4. Overlap in SNPs, Genomic Risk Loci and Mapped Genes Across CC Traits

The number of overlapping signficant SNPS, genomic risk loci and genes across all CC traits

Supplementary Data 5. Cross-Cohort Genetic Correlations Across Phenotypes via LDSC

h2 = heritability computed via LDSC; h2 SE = standard error of heritability estimate, mean Chi2 = mean chi-squared statistic; LambdaGC = measure of GWAS inflation; rg = genetic correlation; SE = standard error of genetic correlation

Supplementary Data 6. Cross-Cohort Genetic Correlations Across Phenotypes via GCTA

GCTA SNP h2 = heritability computed via GCTA; GCTA SE = standard error of heritability estimate, rg = genetic correlation; SE = standard error of genetic correlation, p-value = p-value (two-sided)

Supplementary Data 7. Cross-Trait Genetic Correlations Across CC Phenotypes via LDSC

h2 = heritability computed via LDSC; h2 SE = standard error of heritability estimate, mean Chi2 = mean chi-squared statistic; LambdaGC = measure of GWAS inflation; rg = genetic correlation; SE = standard error of genetic correlation; P = p-value (two-sided)

Supplementary Data 8. Meta-Analysis Heritability of CC Phenotypes via LDSC

h2 = heritability computed via LDSC; h2 SE = standard error of heritability estimate, mean Chi2 = mean chi-squared statistic; LambdaGC = measure of GWAS inflation

Supplementary Data 9. Genetic Correlation between CC Phenotypes and Cortical Surface Area

rg = genetic correlation; SE = standard error, P = p-value (two-sided); The Bonferroni threshold was set to 0.05/(34+34+12) = 0.000625. This is based on every trait of cortial surface area (SA), cortical thickness (CT), and every CC trait used (12)

Supplementary Data 10. Genetic Correlation between CC Phenotypes and Cortical Thickness

rg = genetic correlation; SE = standard error, P = p-value (two-sided); The Bonferroni threshold was set to 0.05/(34+34+12) = 0.000625. This is based on every trait of cortial surface area (SA), cortical thickness (CT), and every CC trait used (12)

Supplementary Data 11. Genetic Correlation between CC Phenotypes and Neuropsychiatric Traits

rg = genetic correlation; SE = standard error, P = p-value; AD = Alzheimer's Disease; ADHD = Attention Deficit Hyperactive Disorder; ASD = Austism Spectrum Disorder; BPD = Bipolar Disorder; BPDI = Bipolar Disorder Type I; BPDII = Bipolar Disorder Type II; IQ = Intelligence Quotient; OCD = Obessive Comulsive Disorder; PTSD = Post-Traumatic Stress Disorder; SCZ = Schizophrenia; The Bonferonni threshold was set to 0.05/(12+15) = 0.0015. This accounts for every CC trait and neuropsychiatric trait used.

Supplementary Data 12. Phenotypic Correlation between CC Phenotypes (using UK Biobank) Pearson's correlation (r) was used to determine phenotypic correlations. The Bonferroni threshold was set to 0.05/(12*12) = 3.4722e-4.This accounted for every CC phenotype used.

Supplementary Data 13. Phenotypic Correlation between CC Phenotypes and Cortical Phenotypes (using UK Biobank)
Pearson's correlation (r) was used to determine phenotypic correlations. The Bonferroni threshold was set to 0.05/(12*68) = 6.12745098e-5. This accounted for every CC and cortical phenotype used.

Supplementary Data 14. Mendelian Randomization Results between CC Phenotypes and Cortical Traits
bxy = ; se = standard error; p = p-value (two-sided); nsnp = number of SNPs

Supplementary Data 15. Mendelian Randomization Results between CC Phenotypes and Neuropsychiatric Traits
bxy = ; se = standard error; p = p-value (two-sided); nsnp = number of SNPs

Supplementary Data 16. Local Genetic Correlations between CC Phenotypes and Cortical Traits via LAVA
chr = chromosome; start = starting base-pair location of locus; end = ending base-pair location of locus; n.snp = number of SNPs in locus; n.pcs = number of prinicple components in locus; phen1 = cortical region; phen1_type = surface area (SA) or cortical thickness (CT) of phen1; phen2 = CC region (Area and MeanThickness correspond to total CC); phen2_type = area or mean thickness of phen2; rho = genetic correlation, rho.lower = lower limit of rho; rho.upper = upper limit of rho; r2 = R-Squared; r2.lower = lower limit of r2; r2.upper = upper limit of r2; p = p-value (two-sided); gene = gene corepsonding to locus; signficance threshold was set at the bonferonni corrected level of 0.05/22973 = 2.176468e-06. This was chosen by correcting for every test across all CC phenotypes and all cortical phenotypes.

Supplementary Data 17. Local Genetic Correlations between CC Phenotypes and Neuropsychiatric Traits via LAVA
chr = chromosome; start = starting base-pair location of locus; end = ending base-pair location of locus; n.snp = number of SNPs in locus; n.pcs = number of prinicple components in locus; phen1 = cortical region; phen1_type = surface area (SA) or cortical thickness (CT) of phen1; phen2 = CC region (Area and MeanThickness correspond to total CC); phen2_type = area or mean thickness of phen2; rho = genetic correlation, rho.lower = lower limit of rho; rho.upper = upper limit of rho; r2 = R-Squared; r2.lower = lower limit of r2; r2.upper = upper limit of r2; p = p-value (two-sided); gene = gene corepsonding to locus; signficance threshold was set at the bonferonni corrected level of 0.05/17909 = 2.791892e-06. This was chosen by correcting for every test run across all CC phenotypes and all neuropsychiatric traits.

Supplementary Data 18. MAGMA Gene-Set Analysis (Pathway Enrichment)
Significant results of the MAGMA gene-set analysis using 18,191 gene sets via MAGMA v1.08. NGENES = Number of genes; BETA (STD) = standardized beta; SE = standard error; P = p-value (two-sided); Bonferonni P = bonferonni corrected p-value; GOMF = gene ontology molecular function; GOCC = gene ontology cellular component; GOMF = gene ontology molecular function. All input into the MAGMA analysis was based on the experiment-wide threshold of p = 6.13e-9 correcting for all traits. Bonferronni correction was applied again for each trait when conducting the MAGMA analysis and testing for each gene-set within the FUMA platform.

Supplementary Data 19. MAGMA Tissue Expression Anlaysis using GTEX-8 Data (Enrichment) Significant results of the MAGMA Tissue Expression analysis using GTEX-8 data. NGENES = Number of genes; BETA (STD) = standardized beta; SE = standard error; P = p-value (two- sided); Bonferonni P = bonferonni corrected p-value. All input into the MAGMA analysis was based on the experiment-wide threshold of p = 6.13e-9 correcting for all traits. Bonferronni correction was applied again for each trait when conducting the MAGMA analysis and testing for each gene-set within the FUMA platform.

Supplementary Data 20. MAGMA Tissue Expression Anlaysis using BrainSpan 11 General Developmental Stages Data (Enrichment)
Significant results of the MAGMA Tissue Expression analysis using BrainSpan 11 general developmenatal stages data. NGENES = Number of genes; BETA (STD) = standardized beta; SE = standard error; P = p-value (two-sided); Bonferonni P = bonferonni corrected p-value. All input into the MAGMA analysis was based on the experiment-wide threshold of p = 6.13e-9 correcting for all traits. Bonferronni correction was applied again for each trait when conducting the MAGMA analysis and testing for each gene-set within the FUMA platform.

Supplementary Data 21. MAGMA Tissue Expression Anlaysis using BrainSpan 29 Different Ages Data (Enrichment)
Significant results of the MAGMA Tissue Expression analysis using BrainSpan 29 different ages data. NGENES = Number of genes; BETA (STD) = standardized beta; SE = standard error; P = p-value (two-sided); Bonferonni P = bonferonni corrected p-value; pcw = post-conception week. All input into the MAGMA analysis was based on the experiment-wide threshold of p = 6.13e-9 correcting for all traits. Bonferronni correction was applied again for each trait when conducting the MAGMA analysis and testing for each gene-set within the FUMA platform.

Supplementary Data 22. MAGMA gene-property analysis of the relationship between CC total mean thickness and cell type specific gene expression.
The table contains all possible pairs of cell types retained from step 2; Dataset: Dataset name; Cell_type: Cell type name; MODEL: Index of the MODEL. Cell types with the same MODEL index are conditioned each other; NGENES: Number of genes used in the analysis; BETA: Effect size; BETA_STD: Standardised effect size; SE: Standard error; P: Cross-datasets conditional P-value conditioned on the other cell type with the same MODEL index (and the average expression of the corresponding dataset); CDM.BETA: Cross-datasets marginal effect size; CDM.BETA_STD: Cross-datasets marginal standardised effect size; CDM.SE: Cross-datasets marginal standard error; CDM.P: Cross-datasets P-value conditioned on the average of the dataset of other cell type with the same MODEL index; Marginal.P: P-value without conditioning on the other cell type (nor the average of the dataset); PS: Proportional significance of the CD conditional P-value relative to the CD marginal P-value in log 10 scale.
PS = -log10(P)/-log10(CDM.P)
Note that when CDM is NA due to collinearlity, PS=-log10(P)/-log10(Marginal.P)
*Pair of cell types have values "NA" when they are collinear.

Supplementary Data 23. LDSC Partitioned Heritability (Enrichment)
Results of the LDSC-SEG analysis. Prop. SNPs = proportion of SNPs, Prop. h2 = proportion heritability, Prop. h2 std error. Enrichment std error = enrichment standard error. Enrichment p = p-value (calculated from two sided Z-statistic). Green bolding represents Bonferroni corrected significance experiment-wide for every trait used in the study (0.05/(53*12)) = 7.86e-5. Orange bolding represents Bonferroni corrected significance trait-wide (0.05/53) = 9.4e-4).

Supplementary Data 24. LDSC Partionined Heritability Using Gene-Expression Data from Multiple Tissues (Enrichment)
Results of the LDSC-SEG analysis using gene-expression data from multiple tissues. Coefficent std error = enrichment standard error. Coefficient p = p-value (calculated from two sided Z-statistic). Bonferroni corrected signifiance experiment-wide threshold is 0.05/(205*12) = 2.03e-5 and the Bonferroni corrected signifiance trait-wide threshold is 0.05/205 = 2.43e-4. There are no significant results for either.

Supplementary Data 25. LDSC Partionined Heritability Using Gene-Expression Data From Chromatin Data (Enrichment)
Results of the LDSC-SEG analysis using chromatin data. Coefficent std error = enrichment standard error. Coefficient p = p-value (calculated from two sided Z-statistic). Green bolding represents Bonferroni corrected signifiance experiment-wide (0.05/(489*12) = 8.52e-6). Orange bolding represents Bonferroni corrected signifiance trait-wide (0.05/489 = 1.02e-4).

Supplementary Data 26. LDSC Partionined Heritability From ImmGen Data (Enrichment)
Results of the LDSC-SEG analysis using ImmGen data. Coefficent std error = enrichment standard error. Coefficient p = p-value (calculated from two sided Z-statistic). Green bolding represents Bonferroni corrected signifiance experiment-wide (0.05/(292*12) = 1.43e-5) for which there are no significant results. Orange bolding represents Bonferroni corrected signifiance trait-wide (0.05/292 = 1.71e-4).

Supplementary Data 27. LDSC Partionined Heritability Using Gene-Expression Data From Different Brain Cell Types (Enrichment)
Results of the LDSC-SEG analysis using brain cell type data. Coefficent std error = enrichment standard error. Coefficient p = p-value (calculated from two sided Z-statistic). Green bolding represents Bonferroni corrected signifiance experiment-wide (0.05/(3*12) = 1.39e-3). Orange bolding represents Bonferroni corrected signifiance trait-wide (0.05/3 = 1.67e-2).

Supplementary Data 28. LDSC Partionined Heritability From GTEx Brain Data (Enrichment)
Results of the LDSC-SEG analysis using GTEx brain data. Coefficent std error = enrichment standard error. Coefficient p = p-value (calculated from two sided Z-statistic). Bonferroni corrected signifiance experiment-wide threshold is 0.05/(13*12) = 3.21e-4 and Bonferroni corrected signifiance trait-wide is 0.05/13 = 3.85e-4. There are no significant results.

Supplementary Data 29. LAVA-TWAS using GTEx-8 Samples (eQTLs)
chr = chromosome; start = starting base-pair location of locus; end = ending base-pair location of locus; n.snp = number of SNPs in locus; n.pcs = number of prinicple components in locus; phen1 = cortical region; phen1_type = surface area (SA) or cortical thickness (CT) of phen1; phen2 = eqtl: expression quantitive trait loci; phen2_type = area or mean thickness of phen2; rho = genetic correlation, rho.lower = lower limit of rho; rho.upper = upper limit of rho; r2 = R-Squared; r2.lower = lower limit of r2; r2.upper = upper limit of r2; p = p-value (two-sided t-test); gene = gene corepsonding to locus; signficance threshold was set at the Bonferroni corrected level of 0.05/24853 = 2.01183e-06. This represents correction for test run between every eQTL with every CC phenotype. Green bolding represents Bonferroni corrected signifiance.

Supplementary Data 30. LAVA-TWAS using GTEx-8 Samples (sQTLs)
chr = chromosome; start = starting base-pair location of locus; end = ending base-pair location of locus; n.snp = number of SNPs in locus; n.pcs = number of principal components in locus; phen1 = cortical region; phen1_type = surface area (SA) or cortical thickness (CT) of phen1; phen2 = eqtl: expression quantitative trait loci; phen2_type = area or mean thickness of phen2;

rho = genetic correlation, rho.lower = lower limit of rho; rho.upper = upper limit of rho; r2 = R-Squared; r2.lower = lower limit of r2; r2.upper = upper limit of r2; p = p-value (two-sided t-test); gene = gene corresponding to locus; significance threshold was set at the Bonferroni corrected level of 0.05/91791 = 5.447157e-07. This represents correction for test run between every sQTL with every CC phenotype. Green bolding represents Bonferroni corrected significance.

Supplementary Data 31. LAVA-TWAS eQTL Enrichment
OR = odds ratio, P = p-value (two-sided), ENS.IDs = Ensembl IDs, p(bon) = 8.1103e-06 (no significant results). Bonferonni significance threshold by correcting for every test run with every gene test (0.05/6165)

Supplementary Data 32. LAVA-TWAS sQTL Enrichment
OR = odds ratio, P = p-value (two-sided), ENS.IDs = Ensembl IDs, p(bon) = 3.341799e-06 (no significant results). Bonferonni significance threshold by correcting for every test run with every gene test (0.05/14962)

Supplementary Data 33. Datasets demographics used in SMACC
Demographic information of datasets used in training and testing of mid CC segmentation and AutoQC of metrics extracted in SMACC

Supplementary Data 34. Comparison of AutoQC models
Performance metrics comparison of different models for automatic quality assurance based on midCC shape metrics

Supplementary Data 35. FreeSurfer and SMACC mid CC segmentation comparison in T1 weighted brain scans
Dice scores comparison for mid CC segmentations using Freesurfer and SMACC in HNU test retest dataset. Examples of two subjects are shown visually.

Supplementary Data 36. Ancestry composition of non-European individuals used in GWAS via KING Ancestry composition of all "non-European" individuals in UK Biobank and ABCD used for the CC GWAS as determined by KING. The HapMap3 Ancestry Panel was used. The number of individuals and percentage of the whole population sample is shown. ASW – African ancestry in Southwest USA, CEU – Utah residents with Northern and Western European ancestry from the CEPH collection, CHB – Han Chinese in Beijing, China, CHD – Chinese in Metropolitan Denver, Colorado, GIH – Gujarati Indians in Houston, Texas, JPT – Japanese in Tokyo, Japan, LWK – Luhya in Webuye, Kenya, MXL – Mexican ancestry in Los Angeles, California, MKK – Maasai in Kinyawa, Kenya, TSI – Toscani in Italia, YRI – Yoruba in Ibadan, Nigeria. Individauls who have multiple mixed ancesteries, but are in a group making up less than 1% of the sample, are labelled as "Other mixed trace ancestries"

Supplementary Data 37 Distinct genomic loci associated with CC Morphometry with ICV as a covariate The most strongly associated lead SNPs in independent genomic loci are shown after merging regions < 250 KB apart into a single locus. chr = chromosome; pos = base pair position; start = start of locus base-pair position; end = end of locus base-pair position; Allele1 = effect allele; Allele2 = non-effect allele; Freq1 = weighted average of frequency for allele 1 across all studies; FreqSE = corresponding standard error for allele frequency estimatel; MinFreq = minimum frequency for allele 1 across all studies; MaxFreq = maximum frequency for allele 1 across all studies; Effect = overall estimated effect size for allele 1; Std = overall standard error for effect size estimate; P = meta-analysis p-value (two-sided); Direction = summary of effect direction for each study, with one '+' or '-' per study; TotalN = total sample size including all studies;

nearestGene = nearest gene of the SNP based on ANNOVAR annotations; dist = distance to the nearest gene. SNPs which are locating in the gene body or 1kb up- or down-stream of TSS or TES have 0; func = functional consequence of the SNP on the gene obtained from ANNOVAR; CADD = Combined Annotation-Dependent depletion score, which predict how deleterious the SNP effect is on protein structure/function (higher scores indicate more deleterious); RDB = RegulomeDB scores predict likelihood of regulatory functionality (lower scores indicate higher likelihood); minChrState = minimum chromatin state across 127 tissue types (lower scores indicate more open chromatin); commonChrState = most common chromatin state in 127 tissue types; uniqID = Unique ID of SNPs consists of chr:position:allele1:allele2 where alleles are alphabetically ordered; nSNPs = Number of unique candidate SNPs in the genomic locus, including non-GWAS-tagged SNPs; nGWASSNPs : Number of the GWAS-tagged candidate SNPs within the genomic locus; nIndSigSNPs : Number of the independent significant SNPs in the genomic locus; IndSigSNPs : rsID of independent significant SNPs in the genomic locus; nLeadSNPs : The number of lead SNPs in the genomic locus; LeadSNPs : rsID of lead SNPs in the genomic locus, EU Effect in Non-EU Range: Wheather the effect of the prinicpal European results falls within the 95% confidence interval of the Non-European results. Effect Direction Condorance: Whether the effect direction from both cohorts are matching direction.

Supplementary Data 38: Comparison of Significant Loci in Analyses without and with ICV as a covariate
the number of common and unique genes identified in each trait-level GWAS with and without ICV included as a covariate. Genes are defined as the nearest to the genomic loci based on the FUMA platform. The table includes Number of Common Genes and their names, Number of Unique Genes in each condition (noICV and ICV), The Overlap Coefficient (Szymkiewicz–Simpson coefficient), and summary LDSC statistics: genetic correlation (rG), standard error (se), Z-score (Z), and p-value (p). ICV = intracranial volume.

Supplementary Data 39: Multi-gene-list analysis conducted in g:Profiler to test for enrichment of genes mapped to genomic loci identified by the present corpus callosum GWAS with and without controlling for ICV
Enrichment of gene onotology categories, biological pathways and transcription factors idenitifed by genes common in the ICV vs no ICV GWAS, specific to no ICV and specific to ICV are shown. GO: Gene Ontology, BP: Biological Process, CC: Cellular Component, KEGG: Kyoto Encyclopedia of Genes and Genomes, REAC: Reactome Pathway Database, TF: TRANSFAC Database. All adjusted p-values (two-sided) within g:Profiler were adjusted using the established g:SCS (sets, counts and sizes) threshold.

Supplementary Data 40: Multi-gene-list analysis conducted in g:Profiler to test for enrichment of genes mapped to genomic loci identified by corpus callosum area, thickness and volume (previous studies) phenotypes
Enrichment of gene onotology categories, biological pathways and transcription factors idenitifed by genes in the present area and thickness GWAS, and previous volume GWAS of the CC. GO: Gene Ontology, BP: Biological Process, CC: Cellular Component, KEGG: Kyoto Encyclopedia of Genes and Genomes, REAC: Reactome Pathway Database, TF: TRANSFAC Database. All adjusted p-values (two-sided) within g:Profiler were adjusted using the established g:SCS (sets, counts and sizes) threshold.

Supplementary Data 41: Multi-gene-list analysis conducted in g:Profiler to test for enrichment of genes mapped to genomic loci identified by corpus callosum area, thickness (with ICV) and volume (previous studies) phenotypes

Enrichment of gene onotology categories, biological pathways and transcription factors idenitifed by genes in the present area and thickness with ICV GWAS, and previous volume GWAS of the CC. GO: Gene Ontology, BP: Biological Process, CC: Cellular Component, KEGG: Kyoto Encyclopedia of Genes and Genomes, REAC: Reactome Pathway Database, TF: TRANSFAC Database. All adjusted p-values (two-sided) within g:Profiler were adjusted using the established g:SCS (sets, counts and sizes) threshold.

Supplementary Data 42: Regional Association Plots

Regional association plots of the meta-analyzed SNP results for each corpus callosum trait.
