# Supplementary material for: The Genetic Architecture of the Human Corpus Callosum and its Subregions
Source: Nat Commun. 2025 Nov 4;16:9708. doi: 10.1038/s41467-025-64791-3 (PMC12586663; doi:10.1038/s41467-025-64791-3)

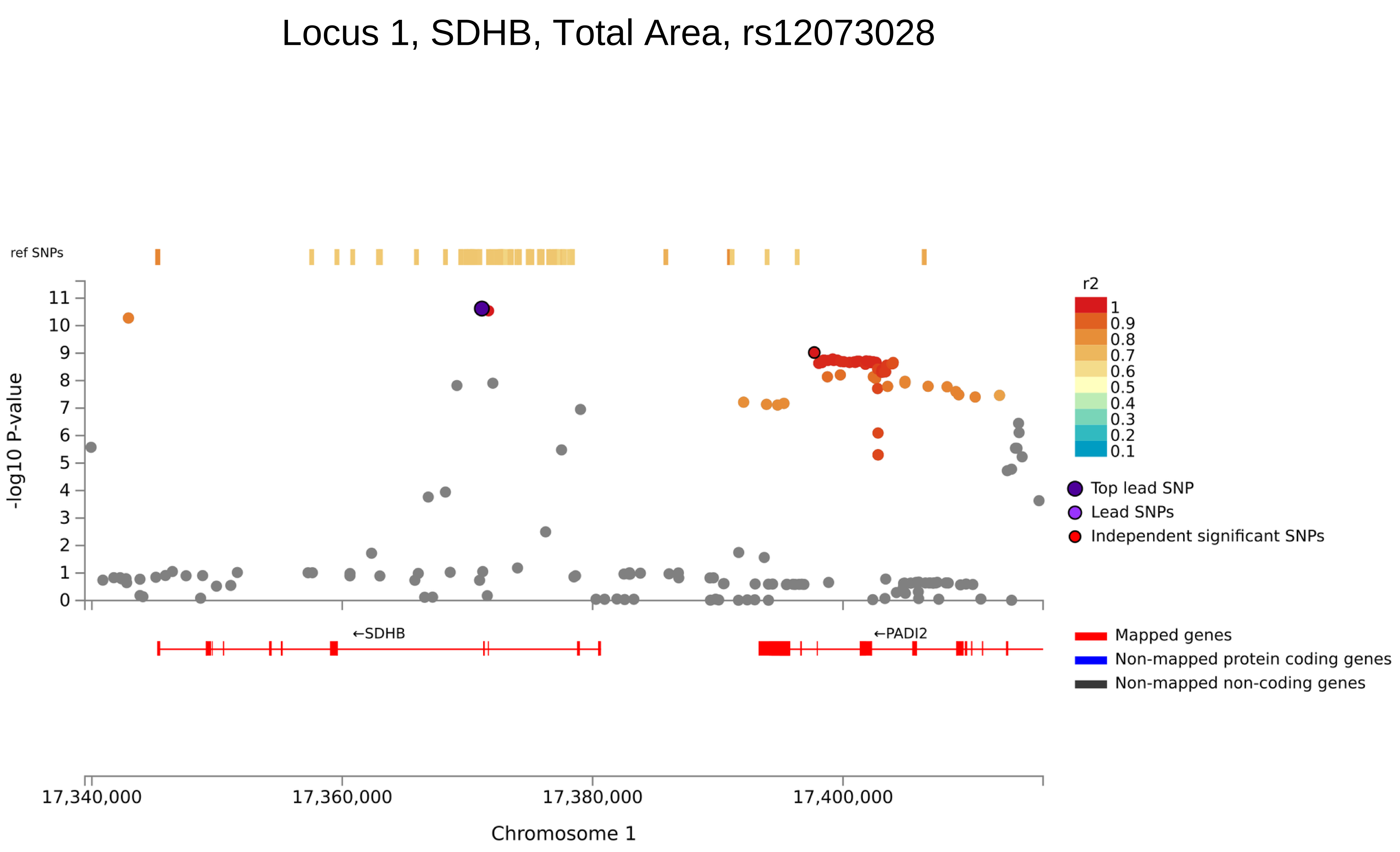
Locus 1, SDHB, Total Area, rs12073028
ref SNPs
-log10 P-value
r2
1
0.9
0.8
0.7
0.6
0.5
0.4
0.3
0.2
0.1
Top lead SNP
Lead SNPs
Independent significant SNPs
←SDHB
←PADI2
Mapped genes
Non-mapped protein coding genes
Non-mapped non-coding genes
17,340,000
17,360,000
17,380,000
17,400,000
Chromosome 1

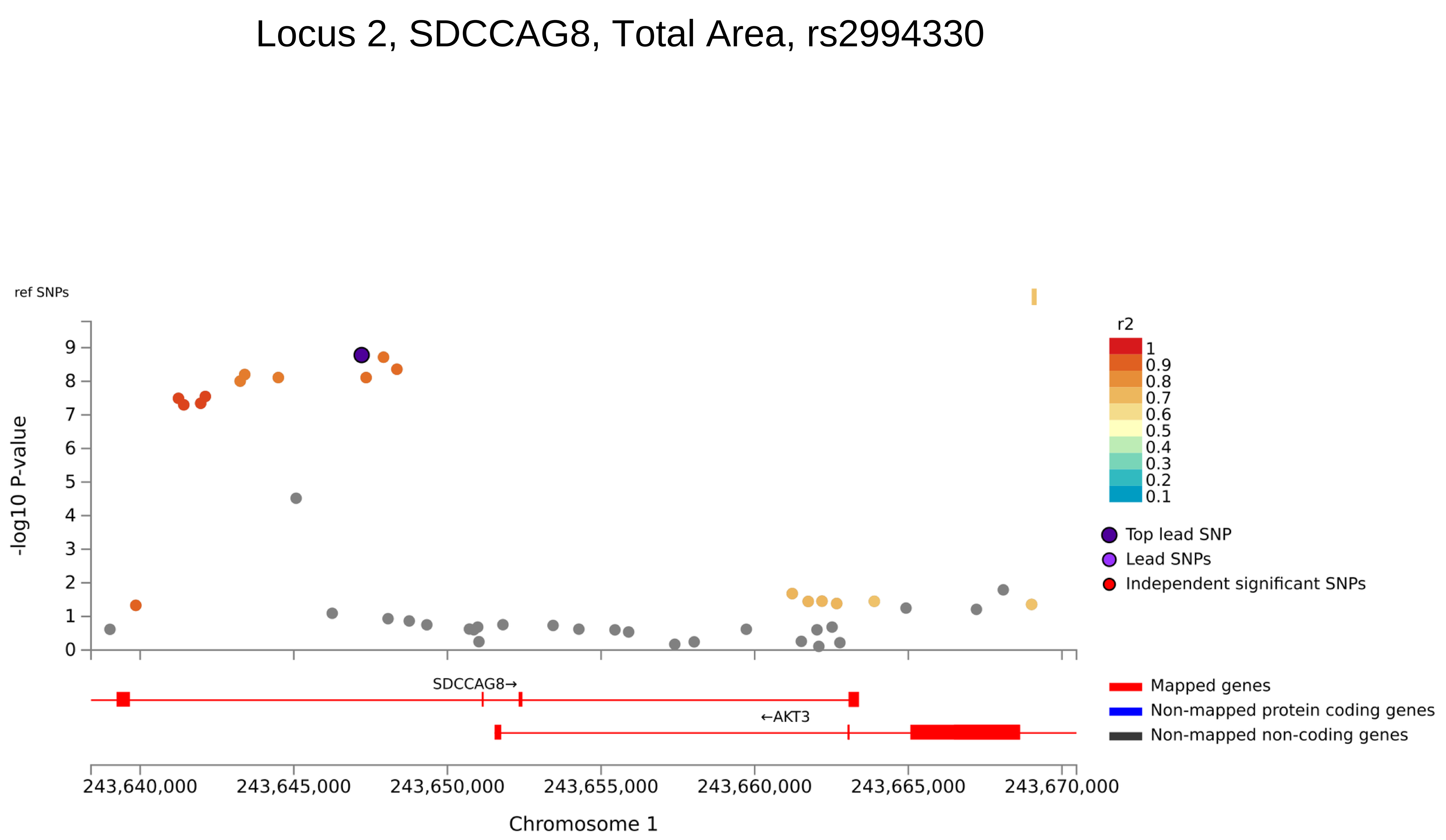
Locus 2, SDCCAG8, Total Area, rs2994330
ref SNPs
-log10 P-value
r2
1
0.9
0.8
0.7
0.6
0.5
0.4
0.3
0.2
0.1
Top lead SNP
Lead SNPs
Independent significant SNPs
SDCCAG8→
←AKT3
Mapped genes
Non-mapped protein coding genes
Non-mapped non-coding genes
243,640,000
243,645,000
243,650,000
243,655,000
243,660,000
243,665,000
243,670,000
Chromosome 1

Locus 3, STRN, Total Area, rs7561572

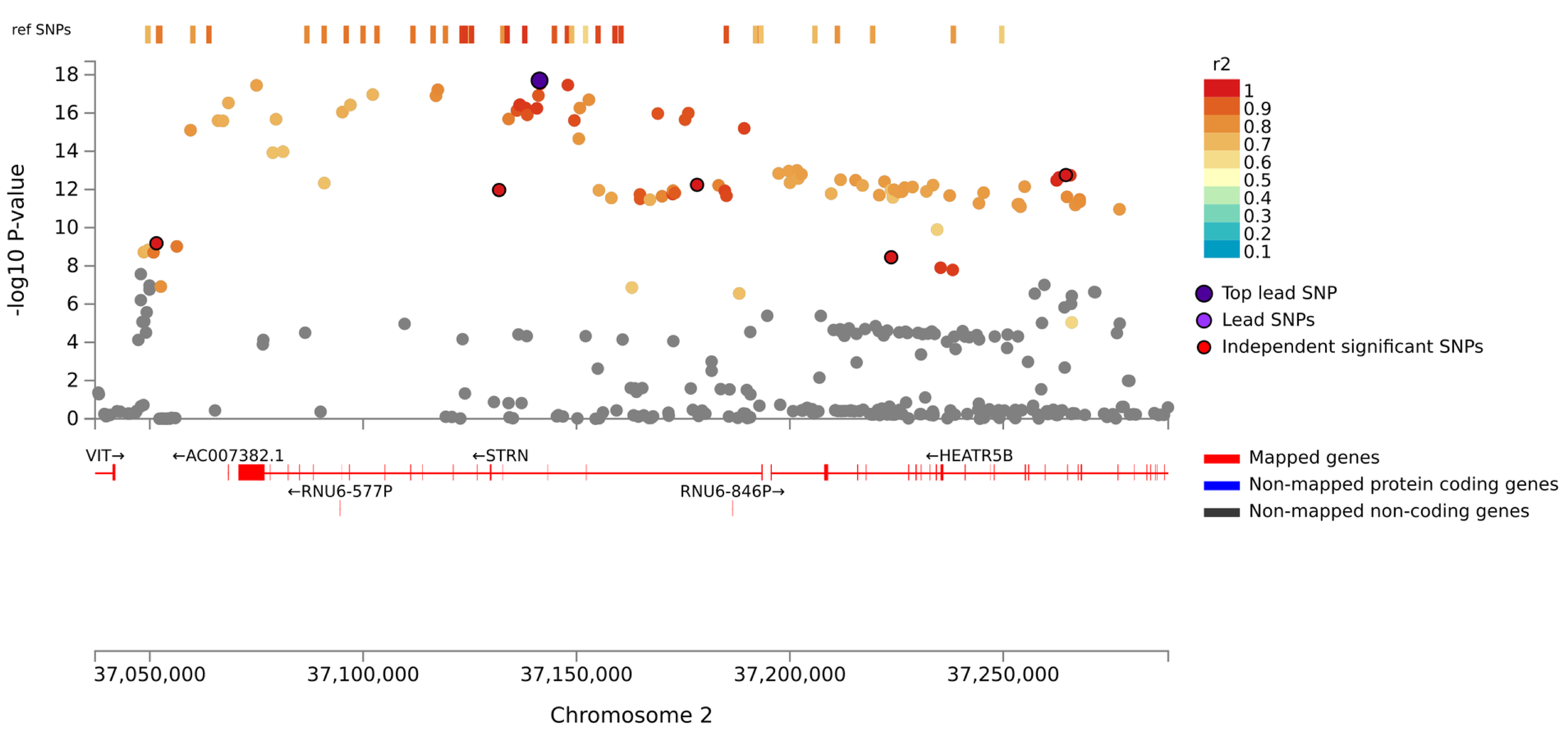

Locus 4, AC016727.1, Total Area, rs778760

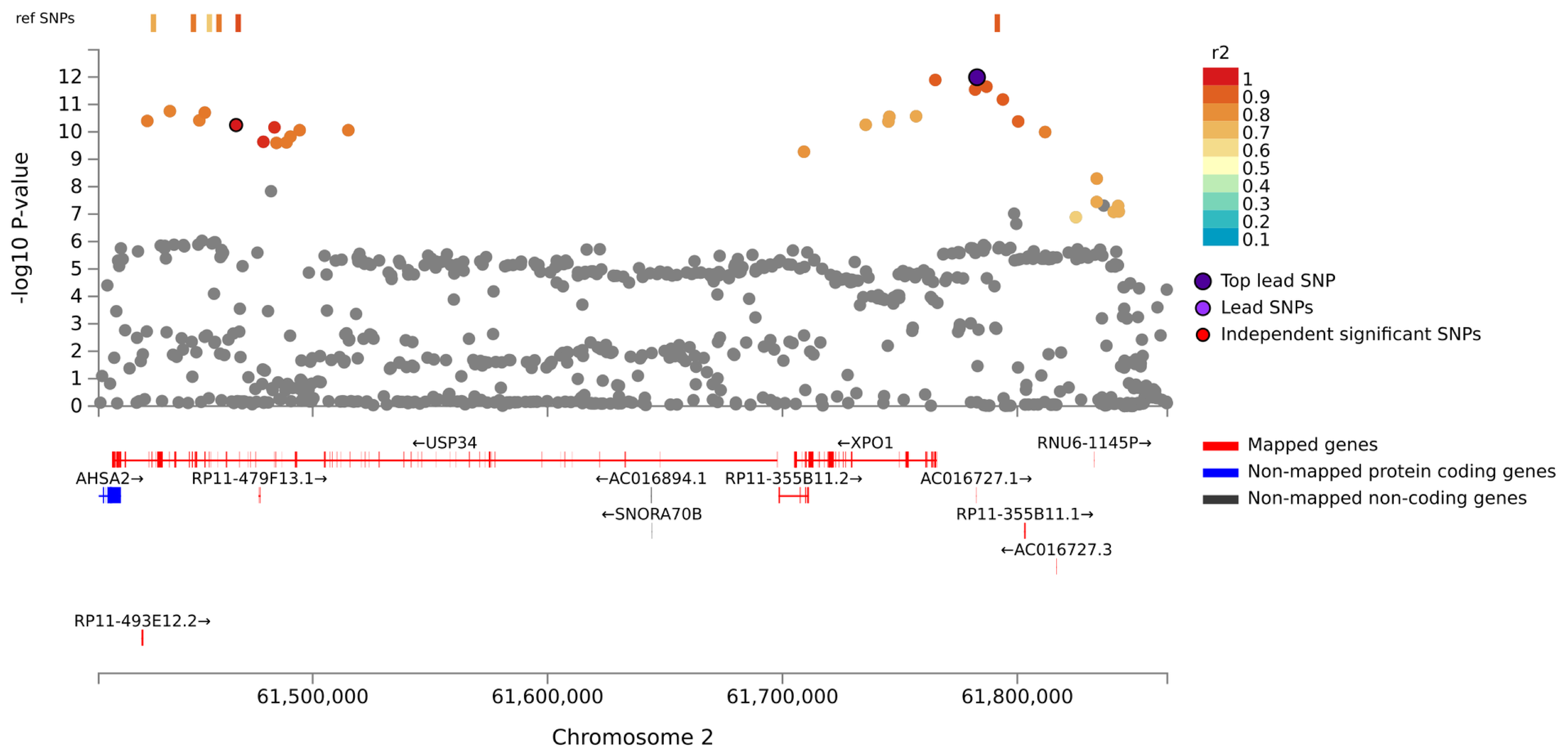

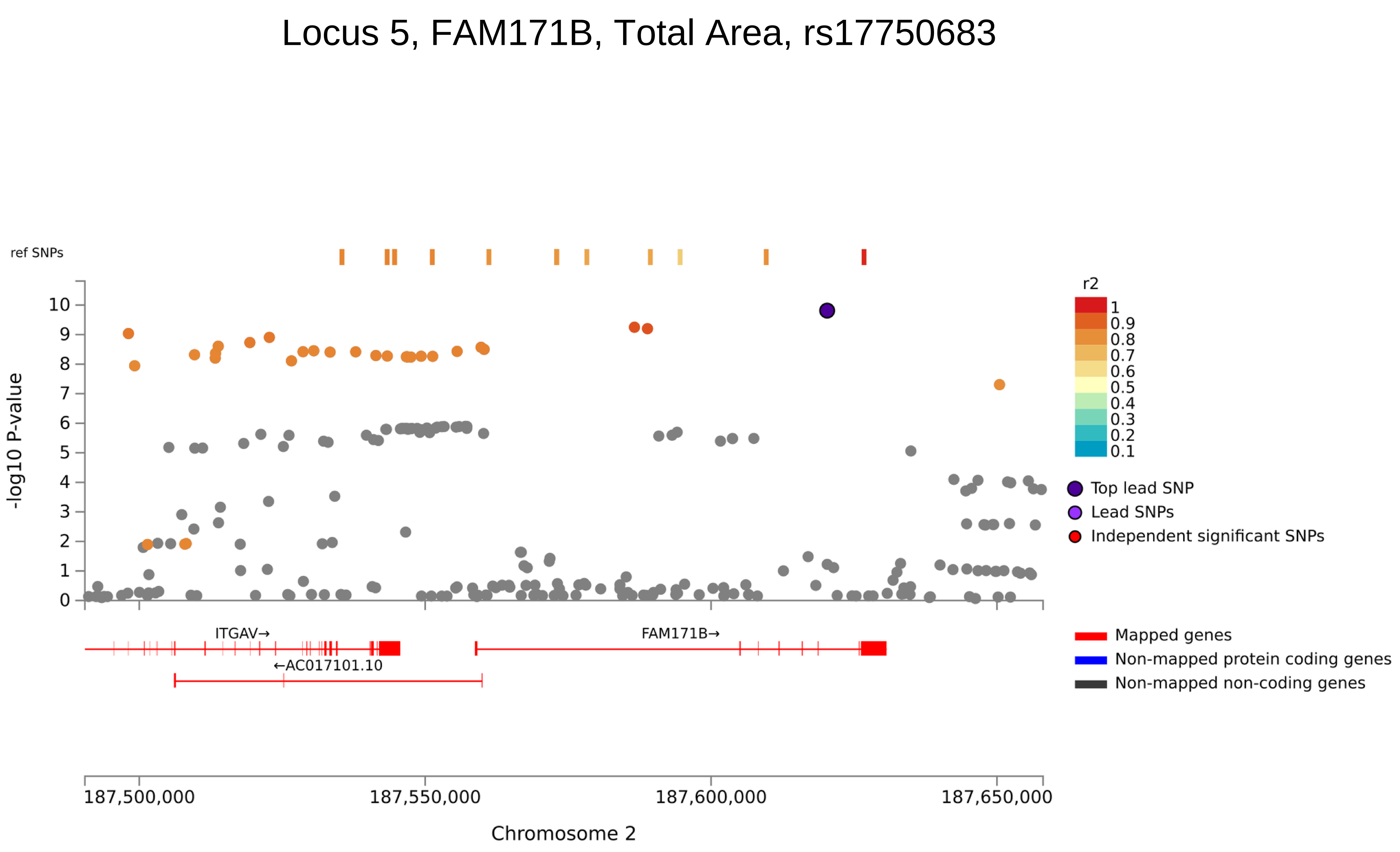
Locus 5, FAM171B, Total Area, rs17750683
ref SNPs
-log10 P-value
r2
Top lead SNP
Lead SNPs
Independent significant SNPs
ITGAV→
←AC017101.10
FAM171B→
Mapped genes
Non-mapped protein coding genes
Non-mapped non-coding genes
187,500,000
187,550,000
187,600,000
187,650,000
Chromosome 2

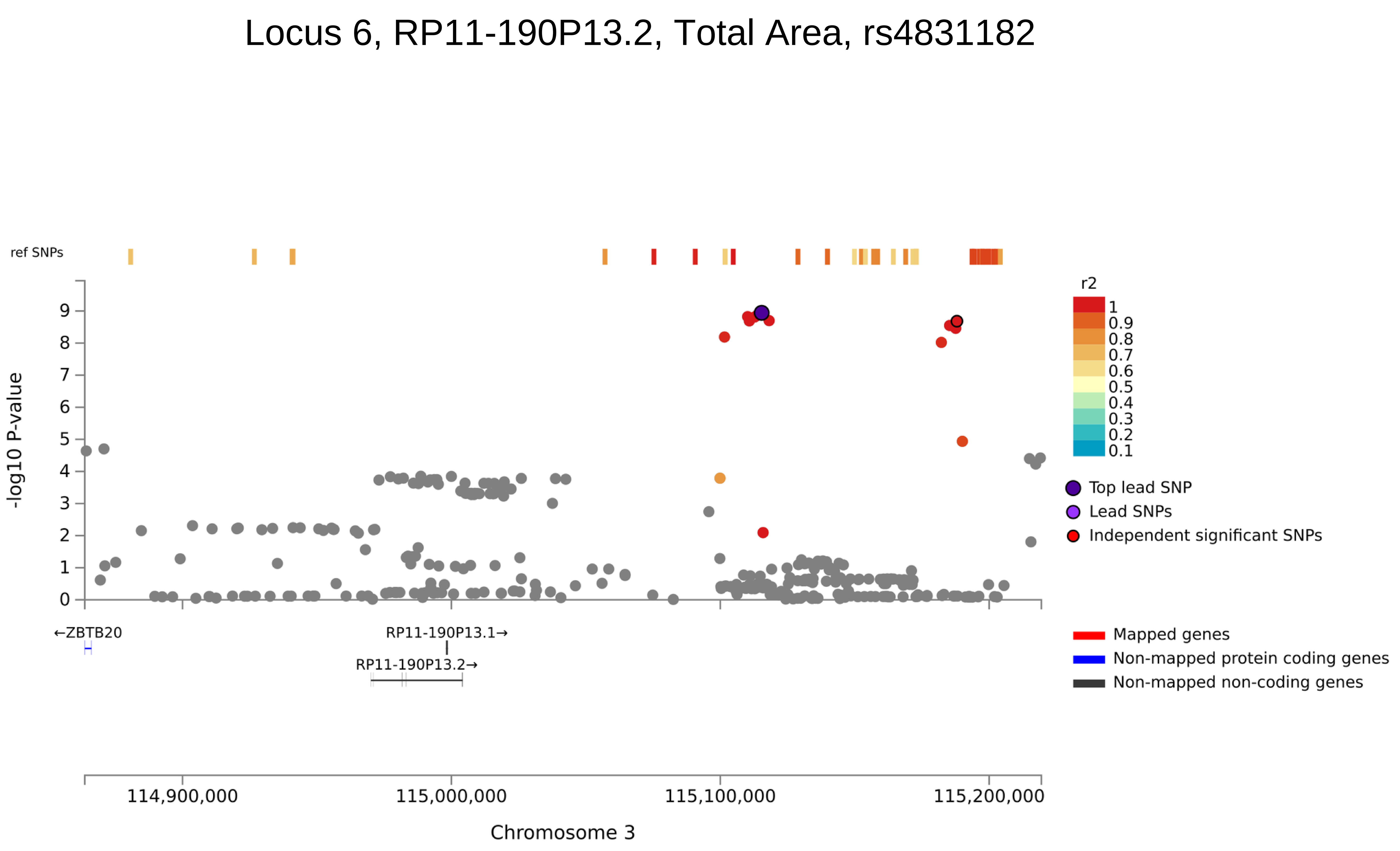
Locus 6, RP11-190P13.2, Total Area, rs4831182
ref SNPs
-log10 P-value
r2
Top lead SNP
Lead SNPs
Independent significant SNPs
←ZBTB20
RP11-190P13.1→
RP11-190P13.2→
Mapped genes
Non-mapped protein coding genes
Non-mapped non-coding genes
114,900,000
115,000,000
115,100,000
115,200,000
Chromosome 3

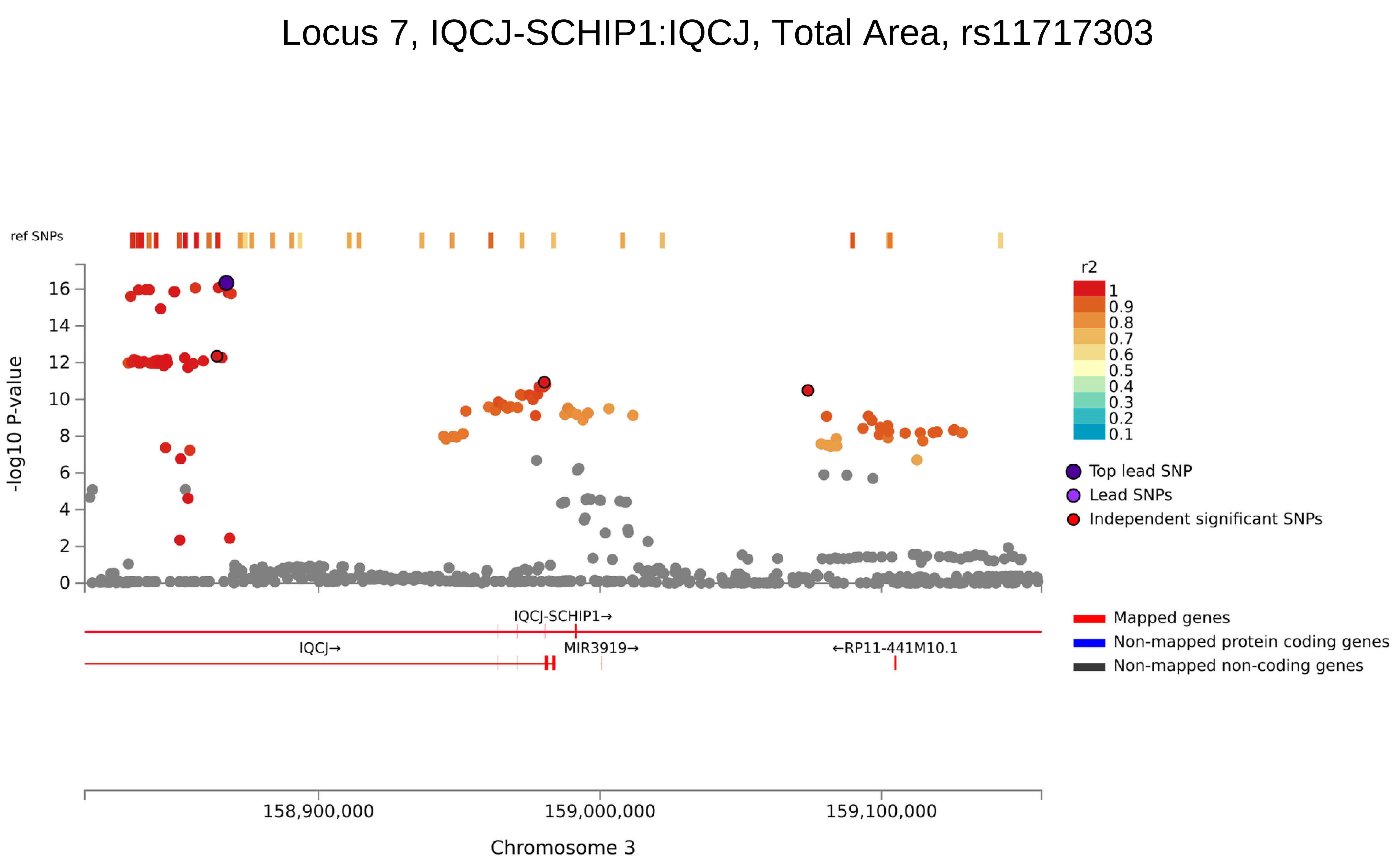
Locus 7, IQCJ-SCHIP1:IQCJ, Total Area, rs11717303
ref SNPs
-log10 P-value
r2
Top lead SNP
Lead SNPs
Independent significant SNPs
Mapped genes
Non-mapped protein coding genes
Non-mapped non-coding genes
IQCJ-SCHIP1→
IQCJ→
MIR3919→
←RP11-441M10.1
158,900,000
159,000,000
159,100,000
Chromosome 3

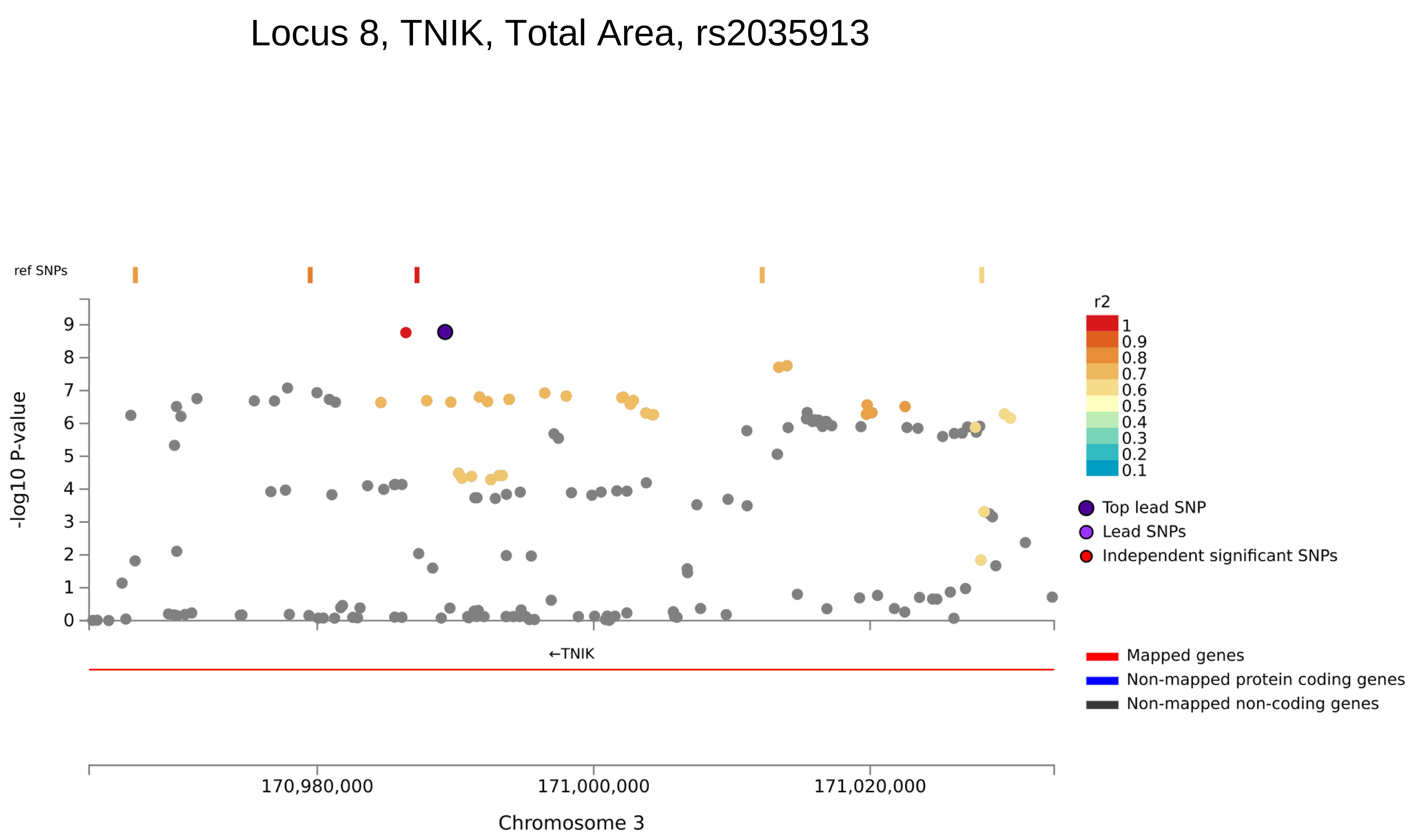
Locus 8, TNIK, Total Area, rs2035913
ref SNPs
-log10 P-value
r2
Top lead SNP
Lead SNPs
Independent significant SNPs
Mapped genes
Non-mapped protein coding genes
Non-mapped non-coding genes
←TNIK
170,980,000
171,000,000
171,020,000
Chromosome 3

Locus 9, FIP1L1, Total Area, rs6835429

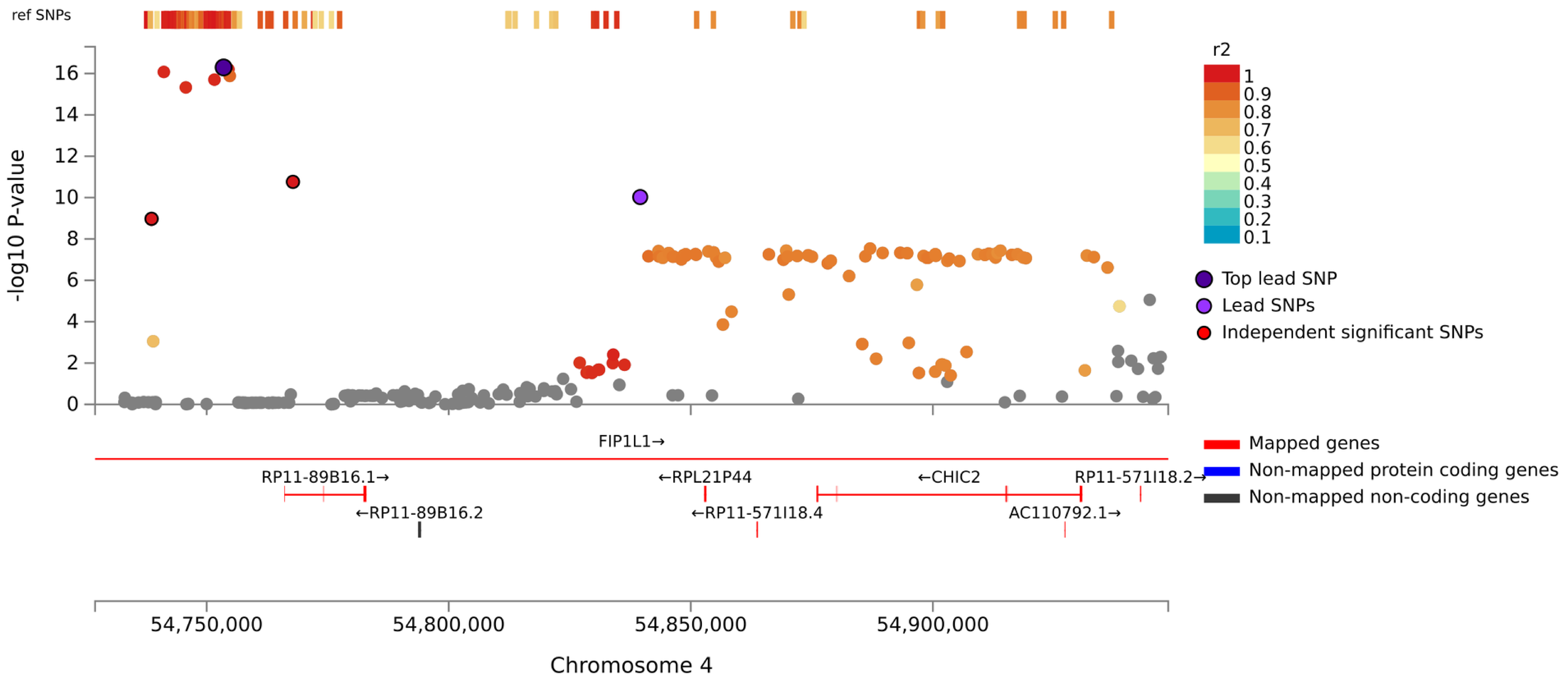

Locus 10, CTD-2316B1.1, Total Area, rs2919904

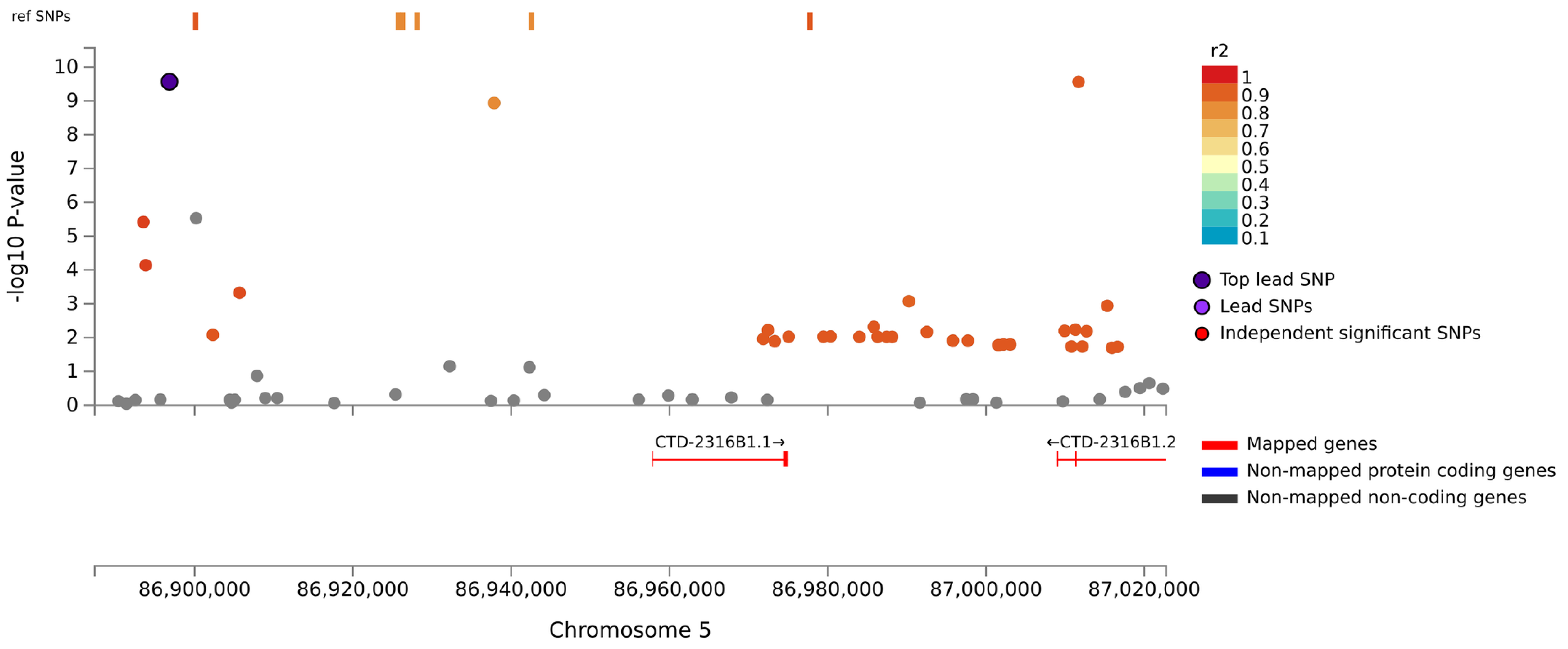

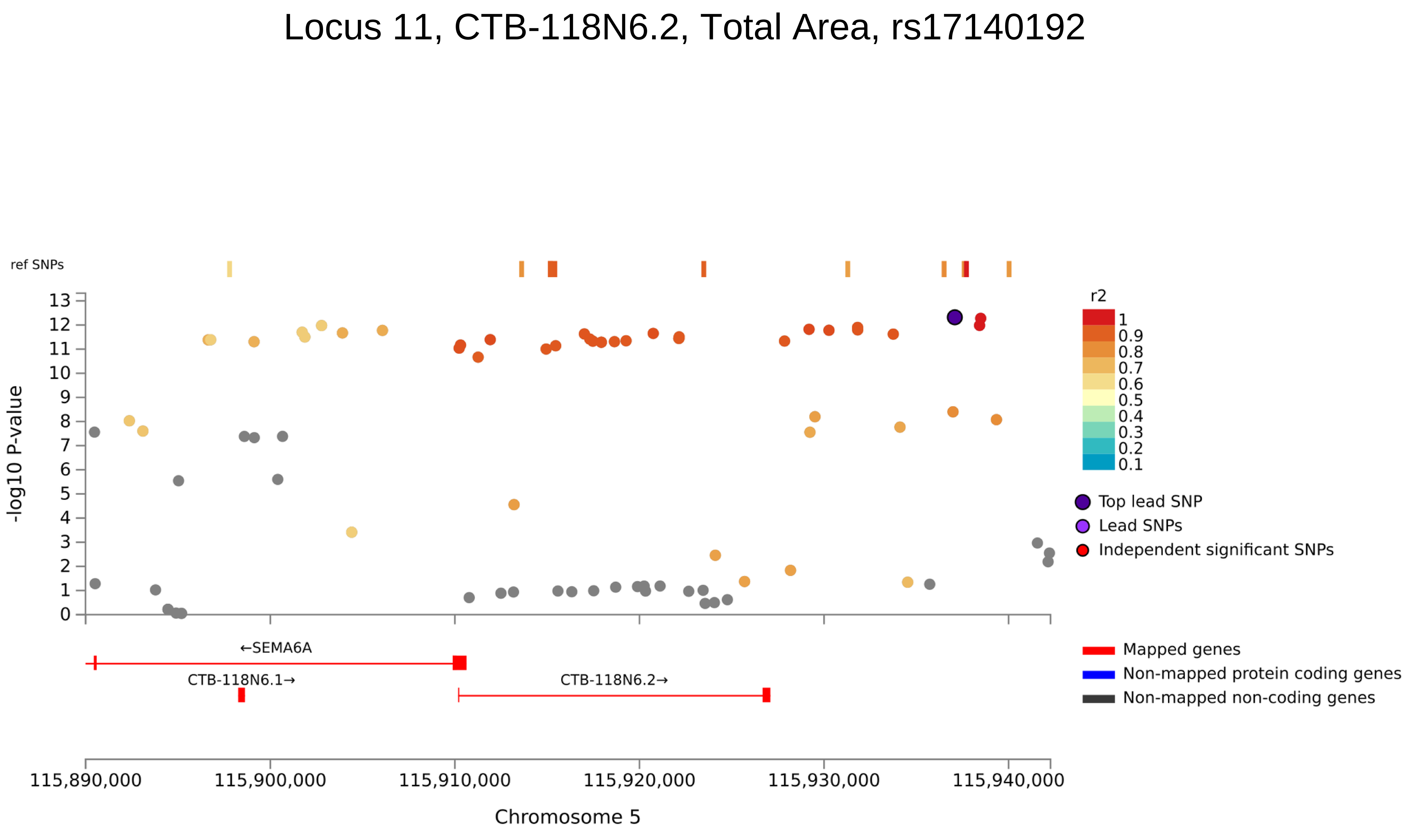

Locus 11, CTB-118N6.2, Total Area, rs17140192
ref SNPs
-log10 P-value
r2
1
0.9
0.8
0.7
0.6
0.5
0.4
0.3
0.2
0.1
Top lead SNP
Lead SNPs
Independent significant SNPs
←SEMA6A
CTB-118N6.1→
CTB-118N6.2→
Mapped genes
Non-mapped protein coding genes
Non-mapped non-coding genes
115,890,000
115,900,000
115,910,000
115,920,000
115,930,000
115,940,000
Chromosome 5

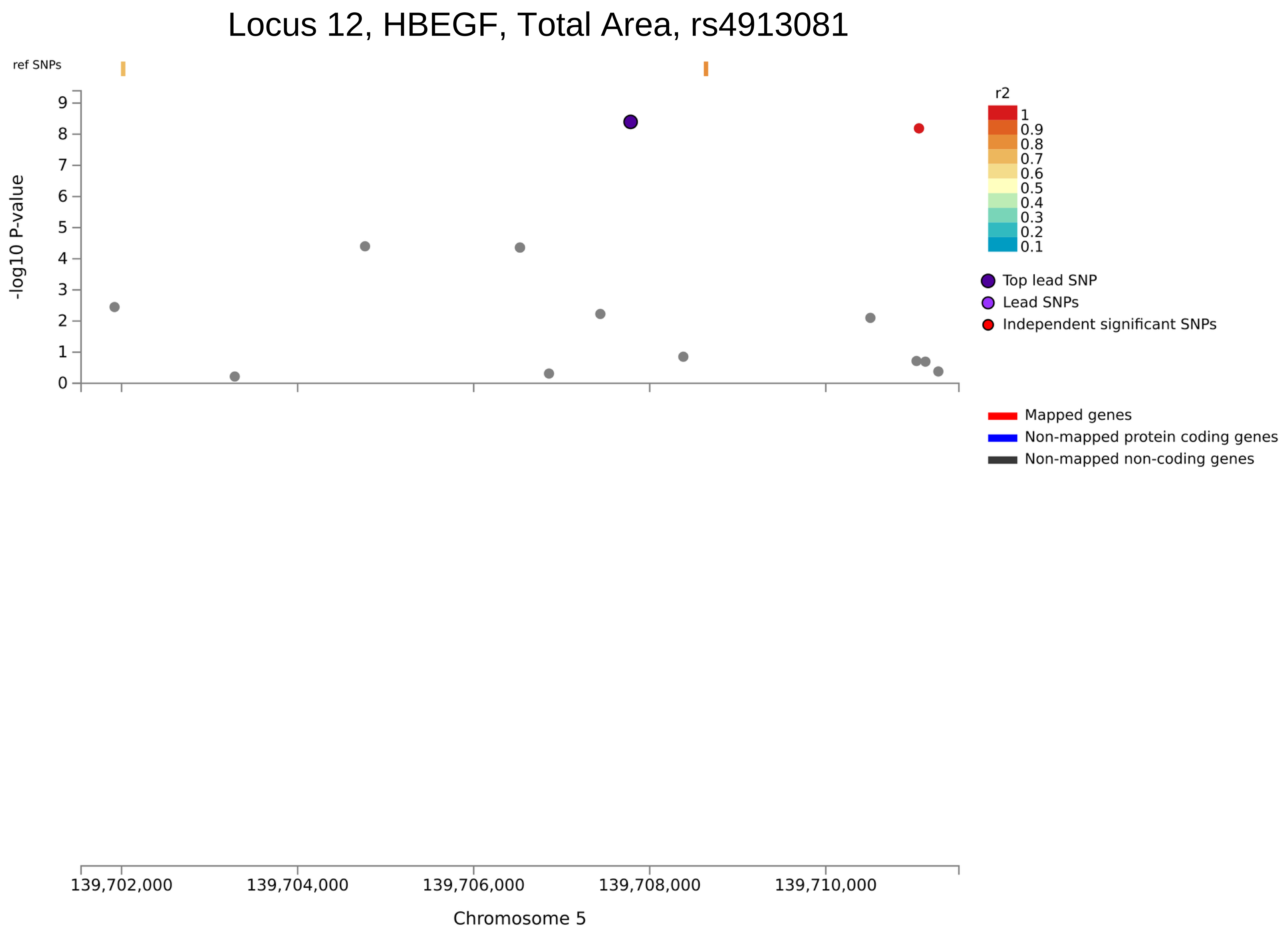

Locus 12, HBEGF, Total Area, rs4913081
ref SNPs
-log10 P-value
r2
Top lead SNP
Lead SNPs
Independent significant SNPs
Mapped genes
Non-mapped protein coding genes
Non-mapped non-coding genes
139,702,000
139,704,000
139,706,000
139,708,000
139,710,000
Chromosome 5

Locus 13, FOXO3, Total Area, rs9486902

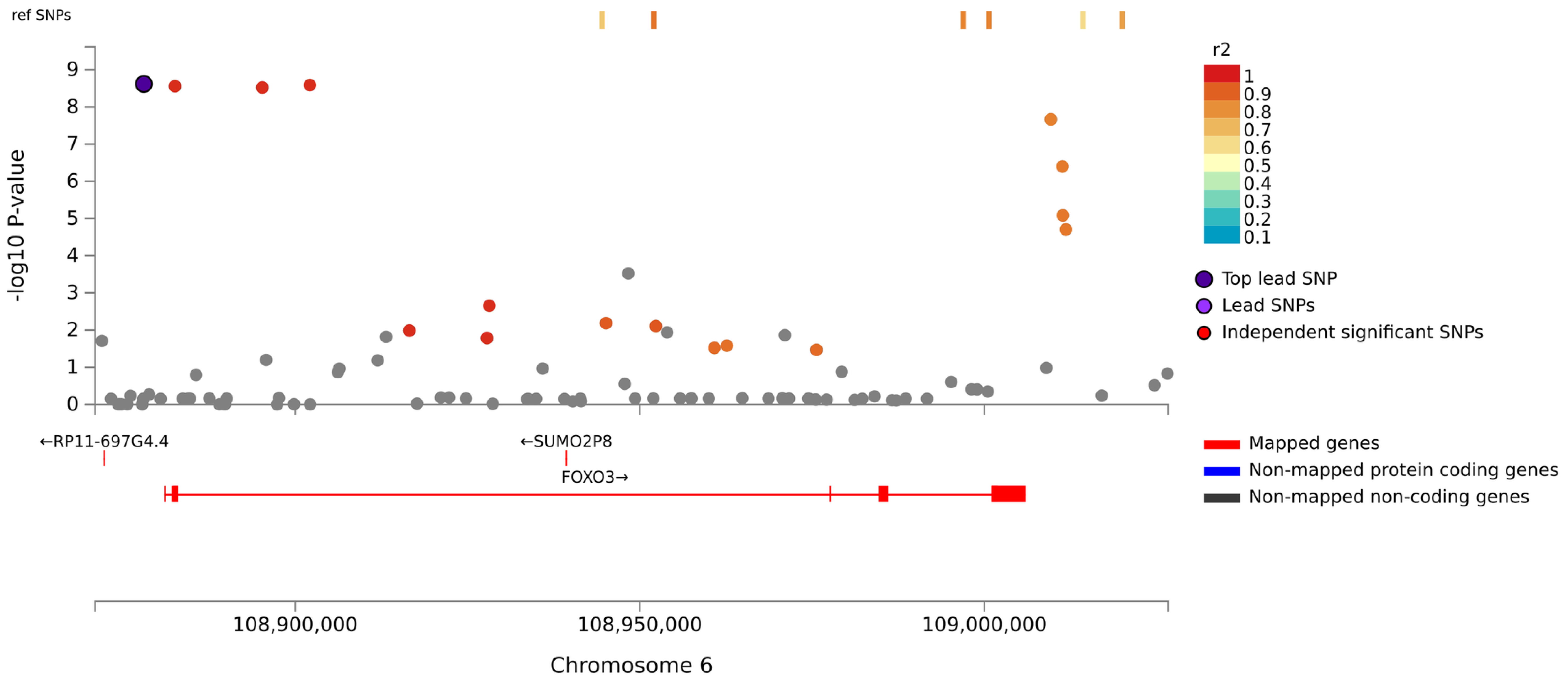

Locus 14, SNORA73, Total Area, rs76928645

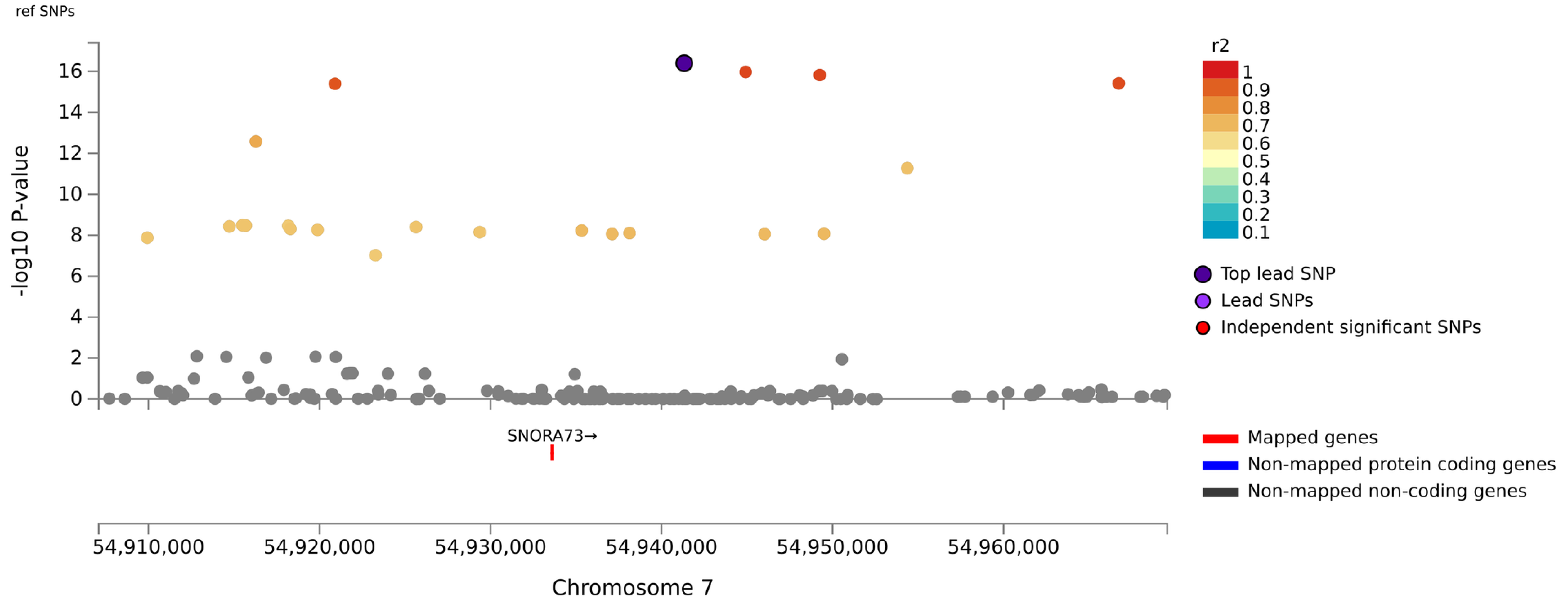

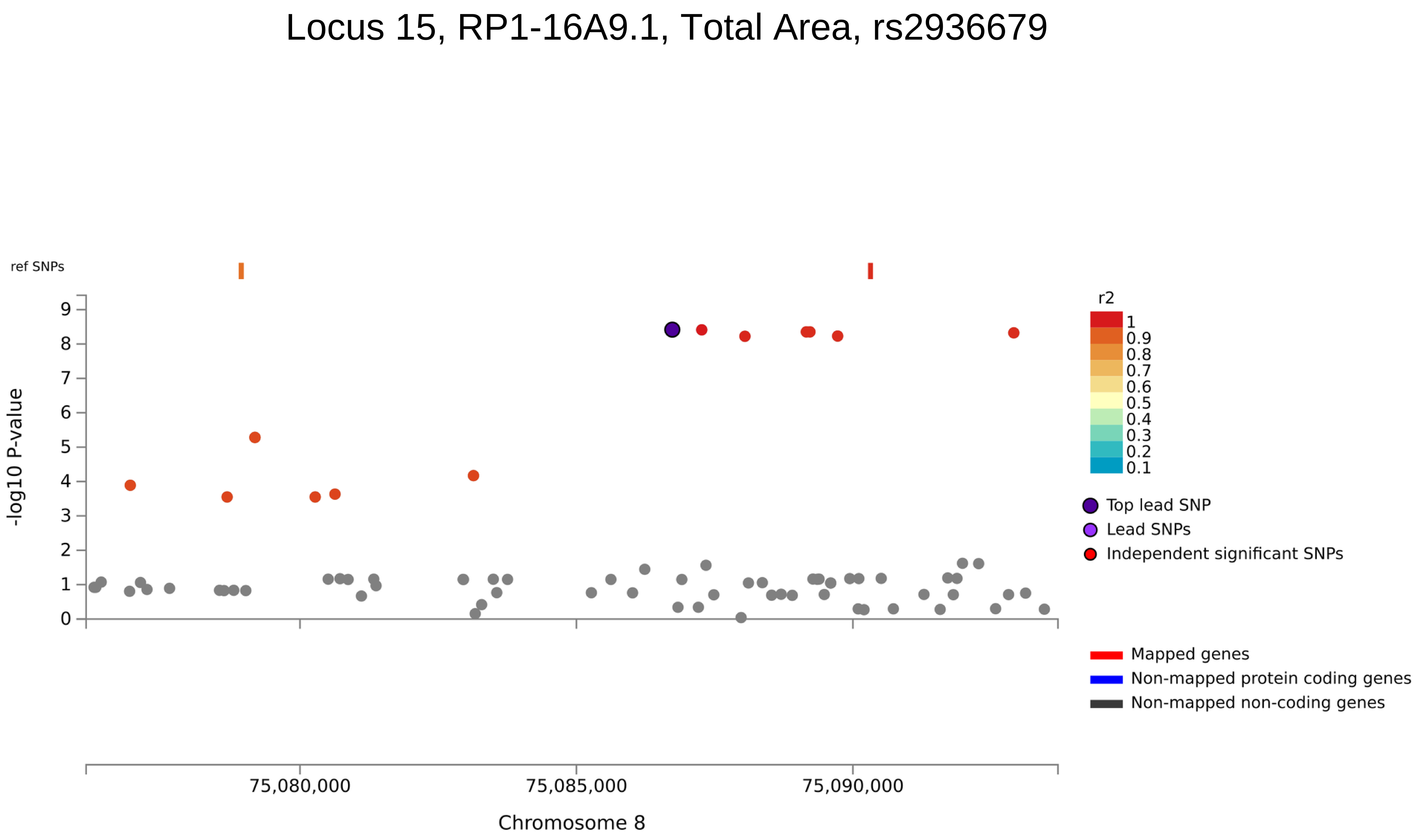
Locus 15, RP1-16A9.1, Total Area, rs2936679
ref SNPs
-log10 P-value
r2
Top lead SNP
Lead SNPs
Independent significant SNPs
Mapped genes
Non-mapped protein coding genes
Non-mapped non-coding genes
75,080,000
75,085,000
75,090,000
Chromosome 8

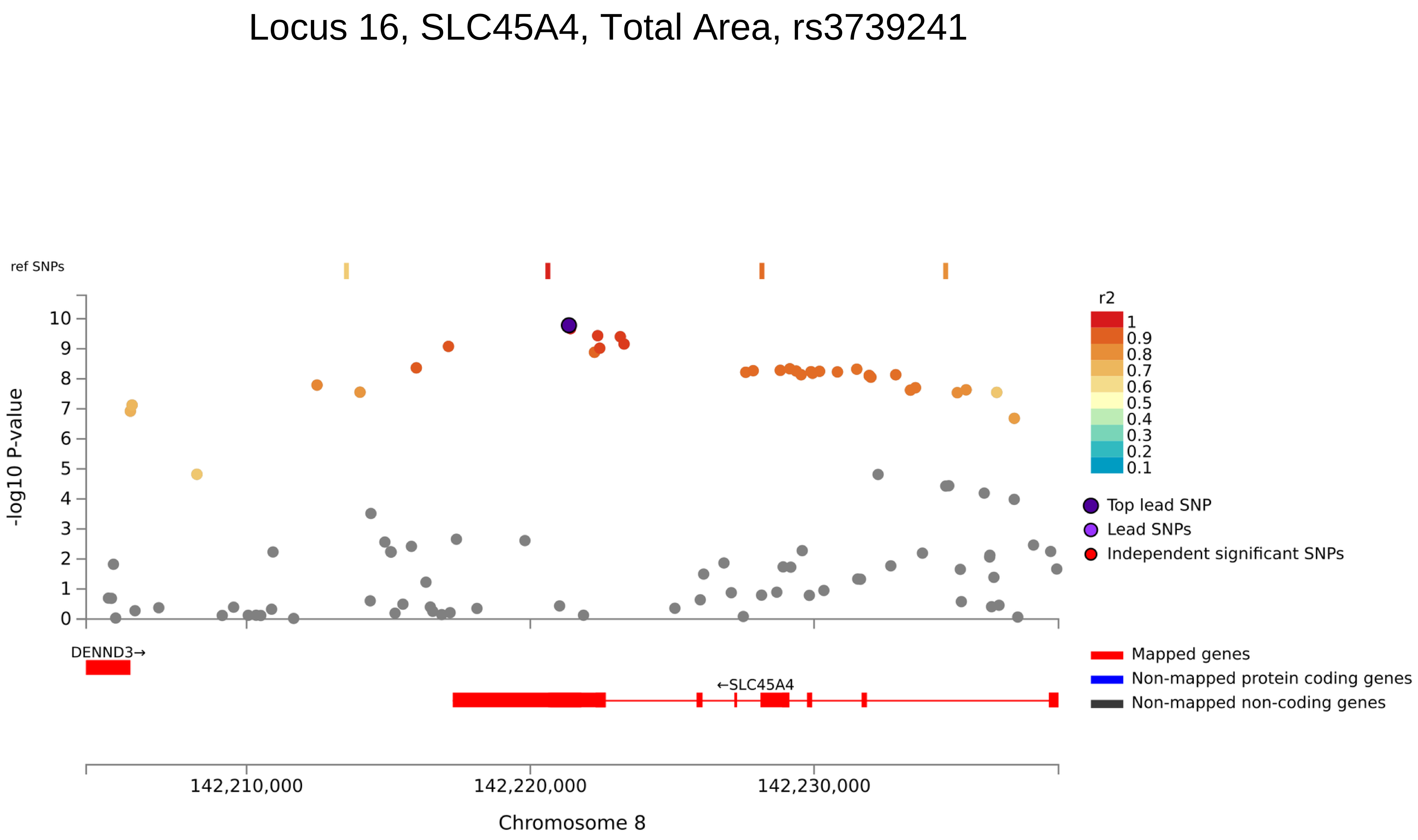
Locus 16, SLC45A4, Total Area, rs3739241
ref SNPs
-log10 P-value
r2
Top lead SNP
Lead SNPs
Independent significant SNPs
DENND3→
←SLC45A4
Mapped genes
Non-mapped protein coding genes
Non-mapped non-coding genes
142,210,000
142,220,000
142,230,000
Chromosome 8

Locus 17, PLEC, Total Area, rs55646585

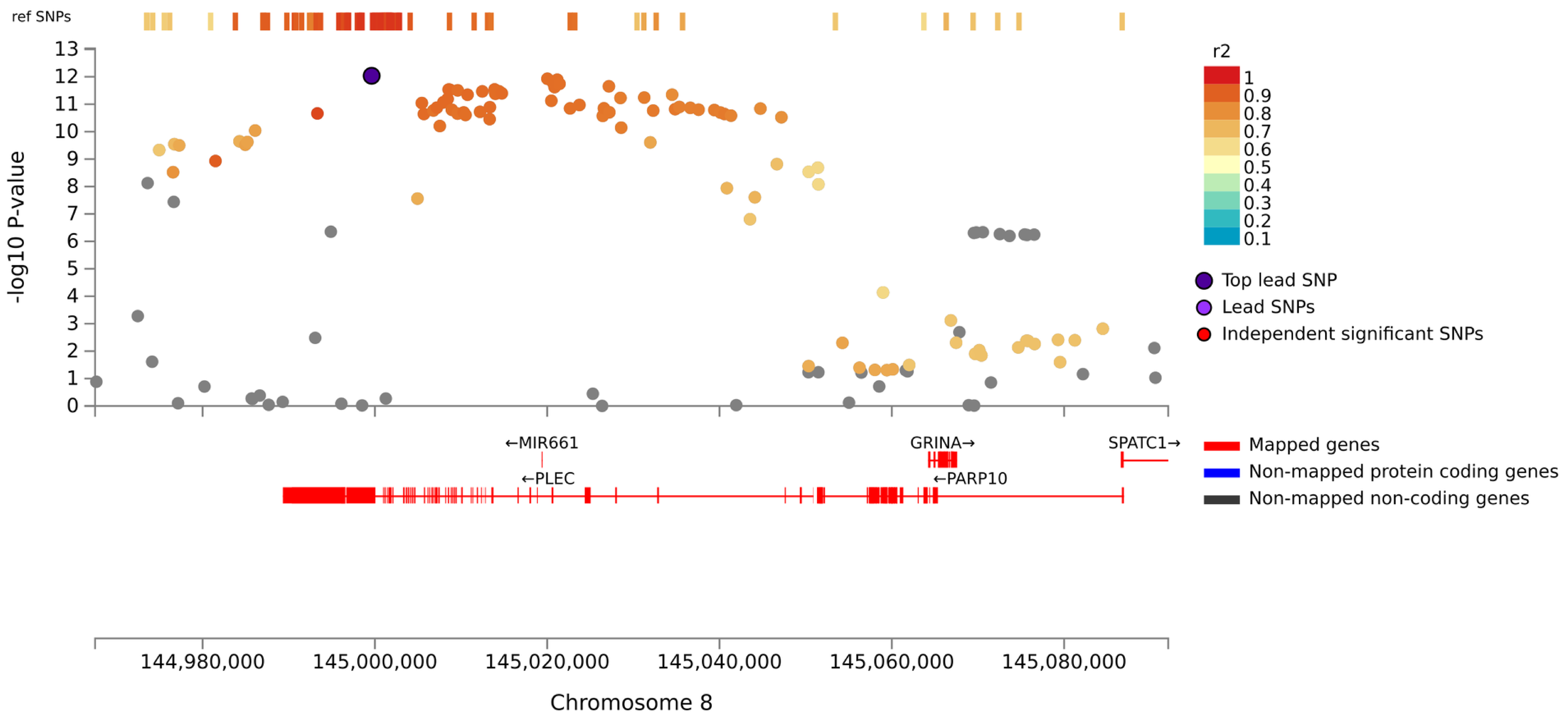

Locus 18, CDKN2B-AS1, Total Area, rs4451405

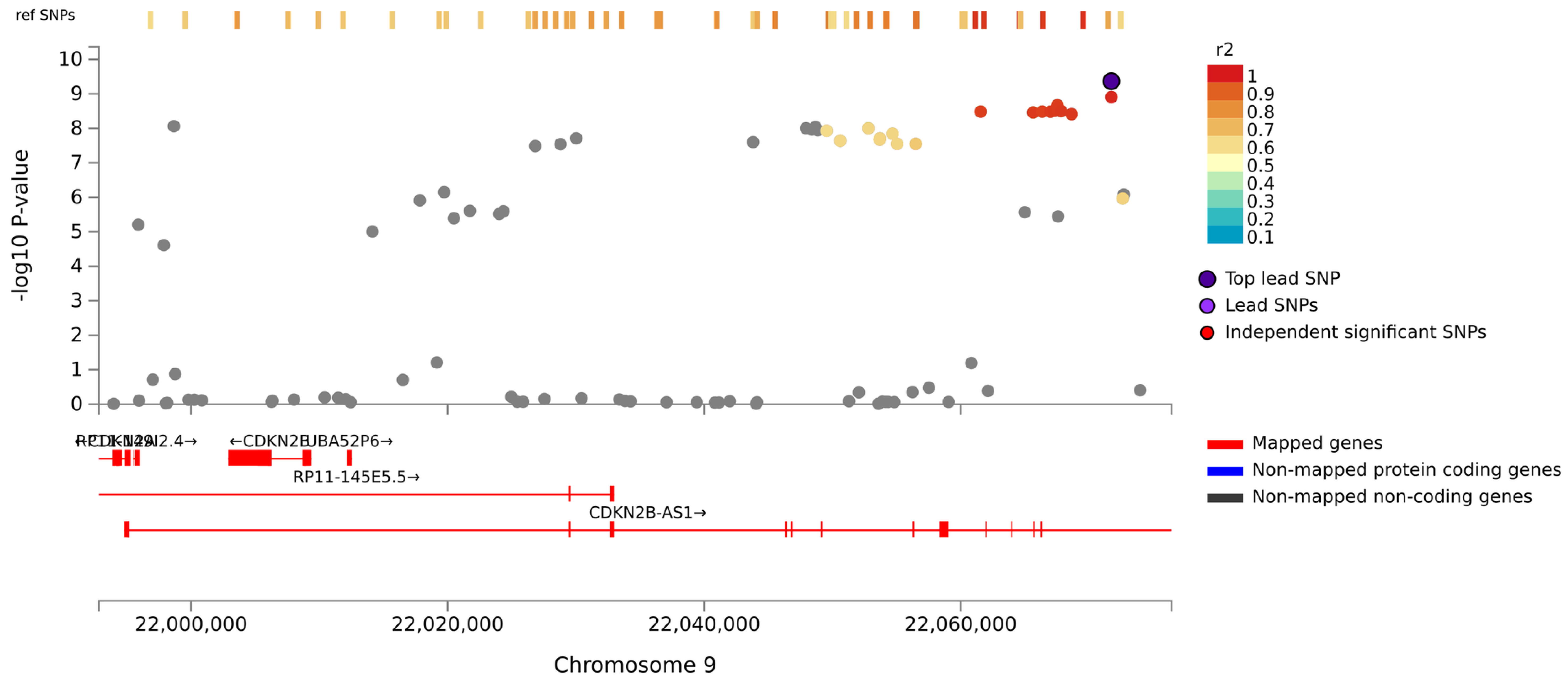

Locus 19, BICD2, Total Area, rs10992447

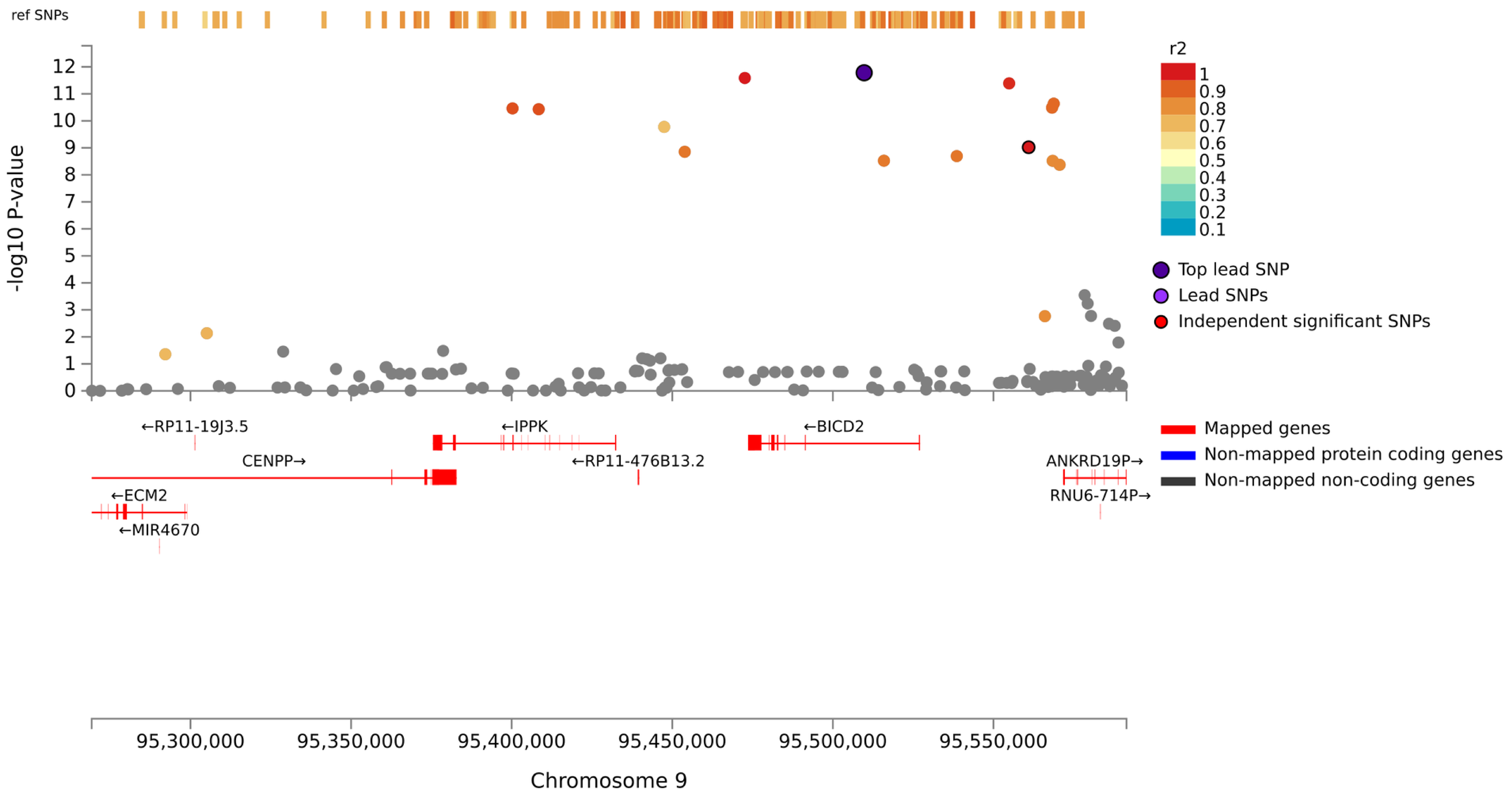

Locus 20, FAM107B, Total Area, rs1410071

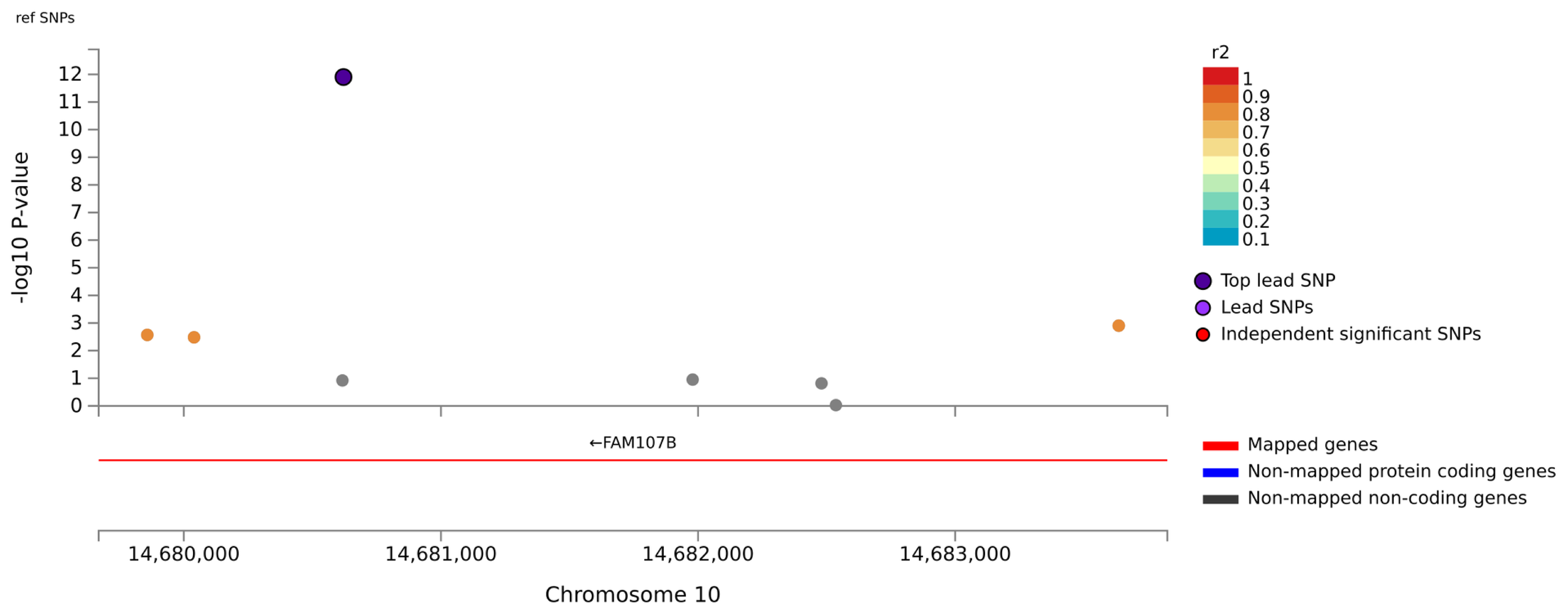

Locus 21, KIAA1598, Total Area, rs1122688

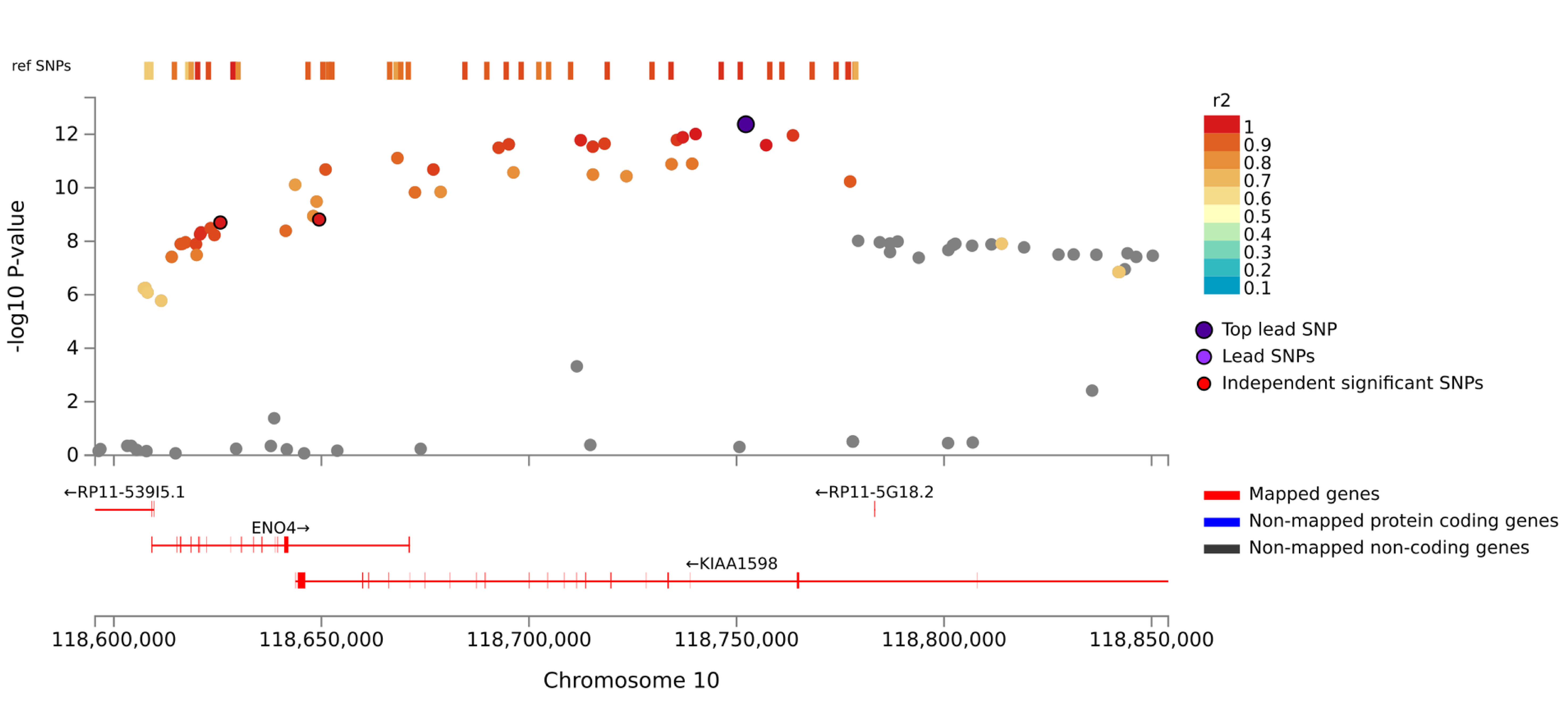

Locus 22, BRSK2, Total Area, rs7947308

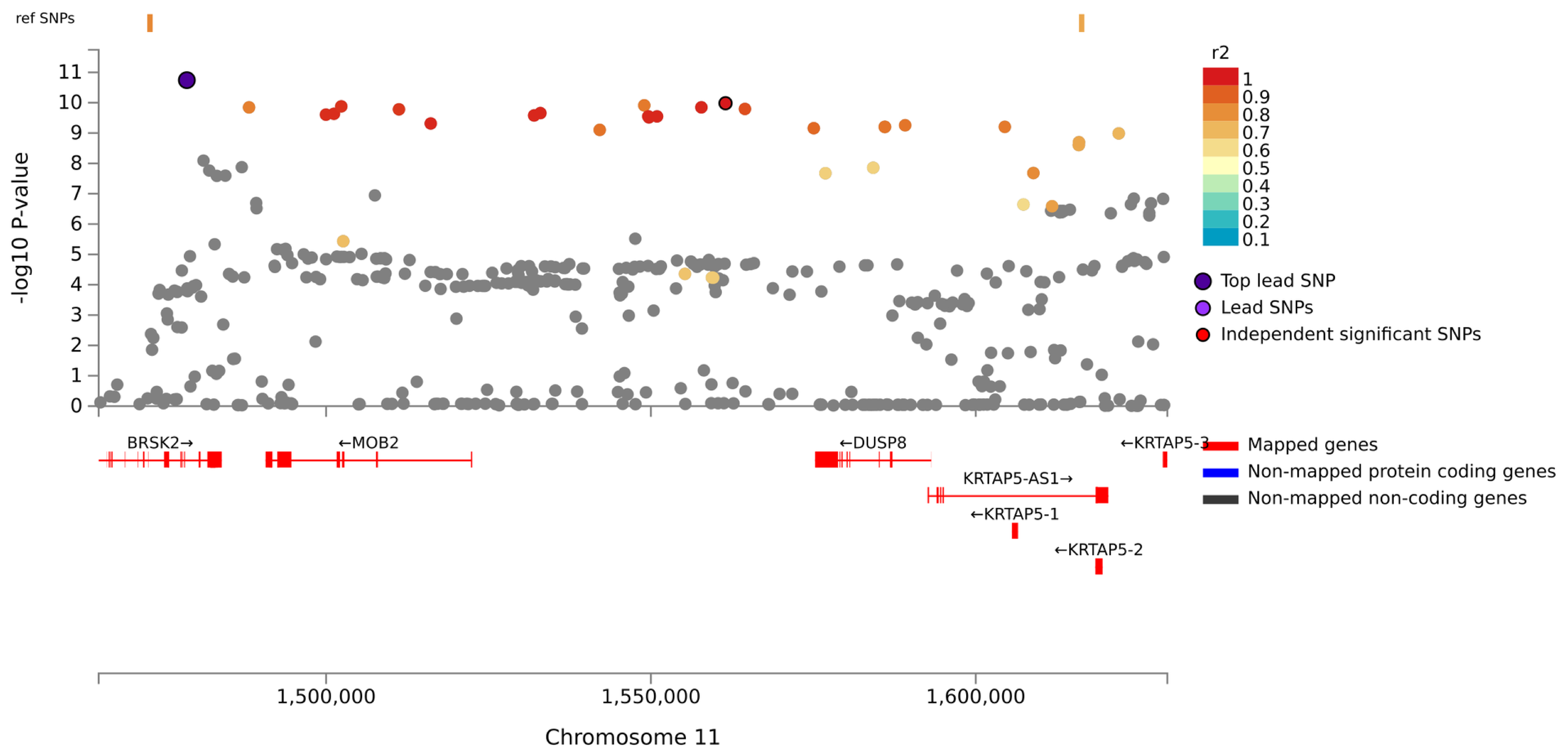

Locus 23, NAV2, Total Area, rs2625320

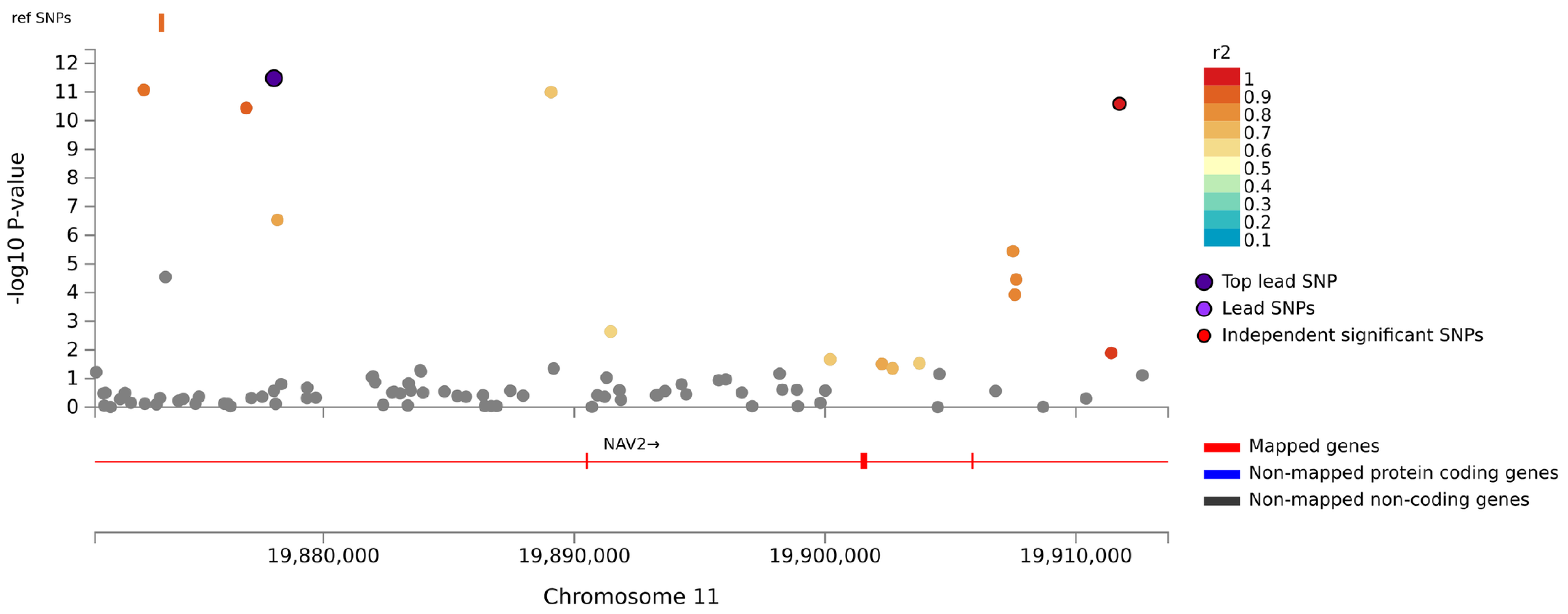

Locus 24, PPP2R5E, Total Area, rs11435884

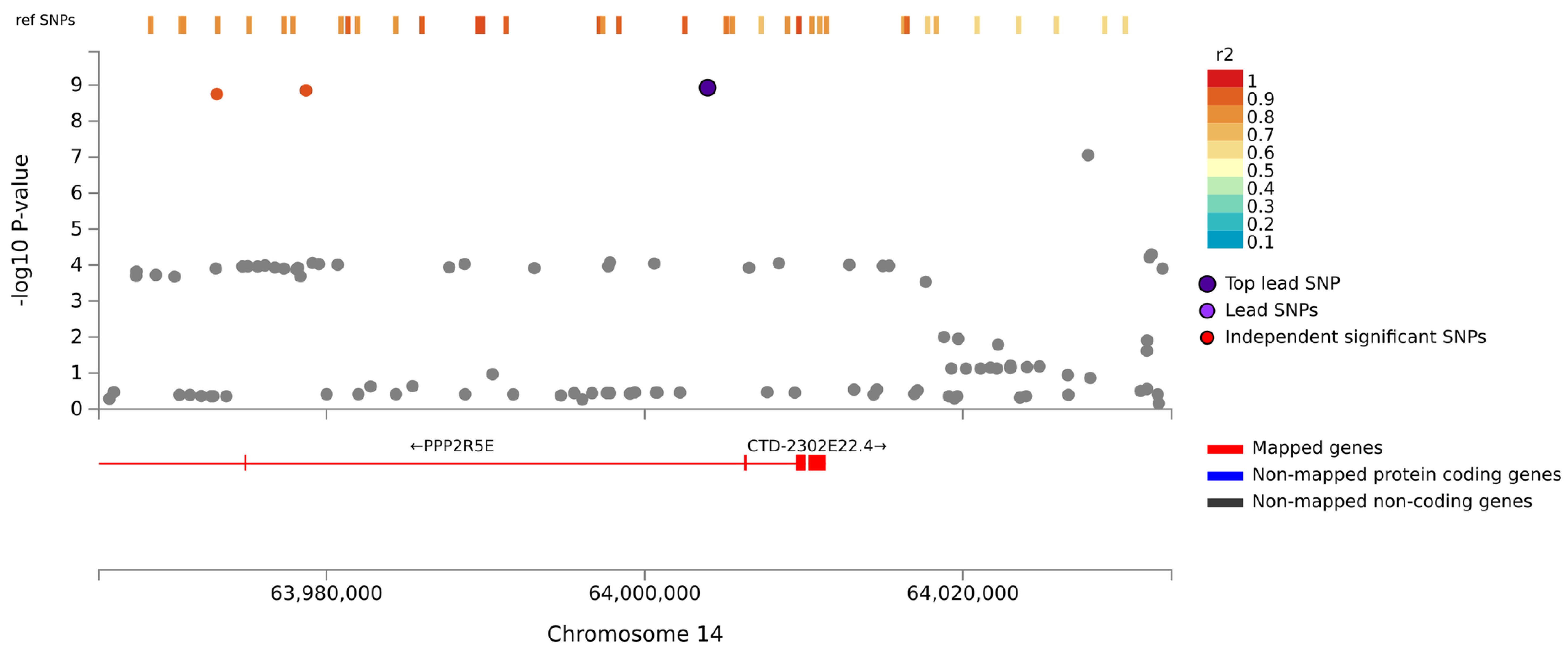

Locus 25, KPNA2, Total Area, rs62086903

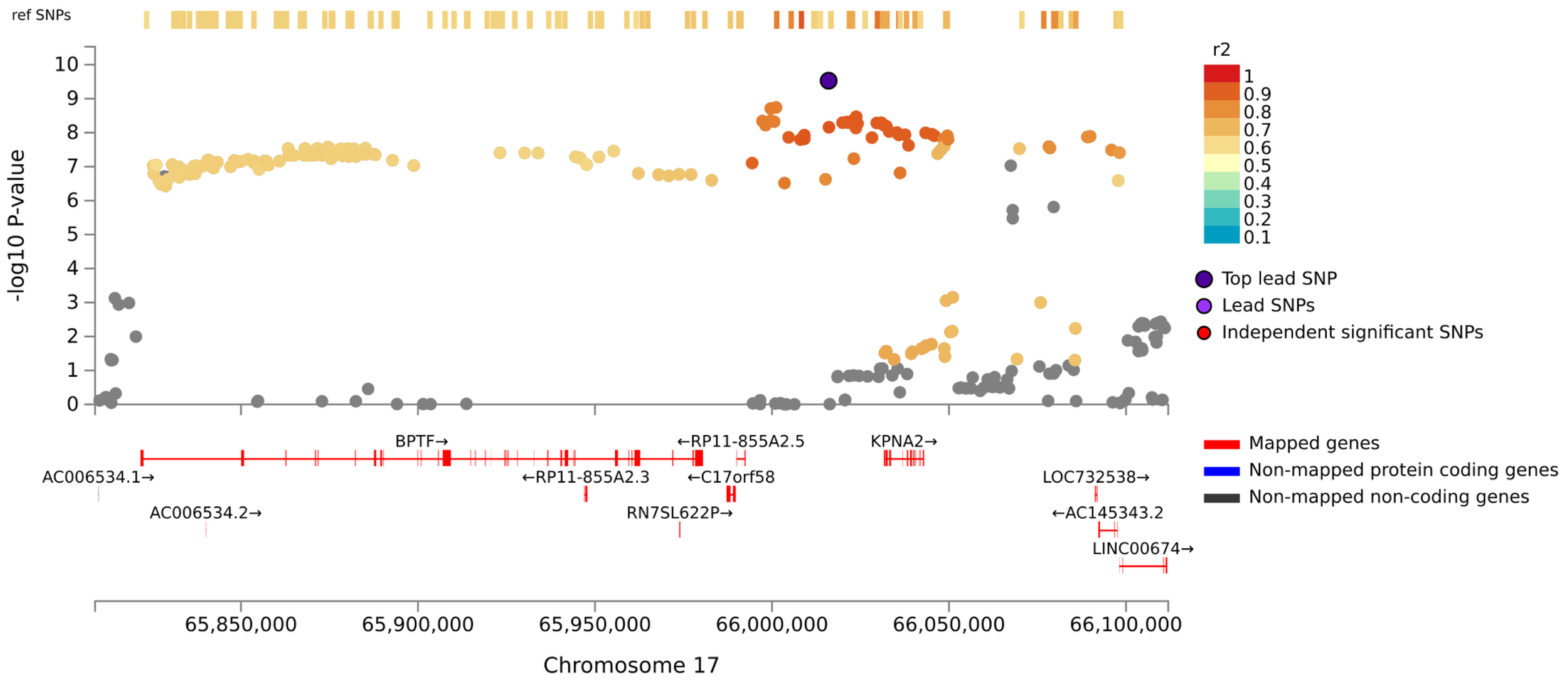

Locus 26, TTC39C:RP11-799B12.2, Total Area, rs8083625

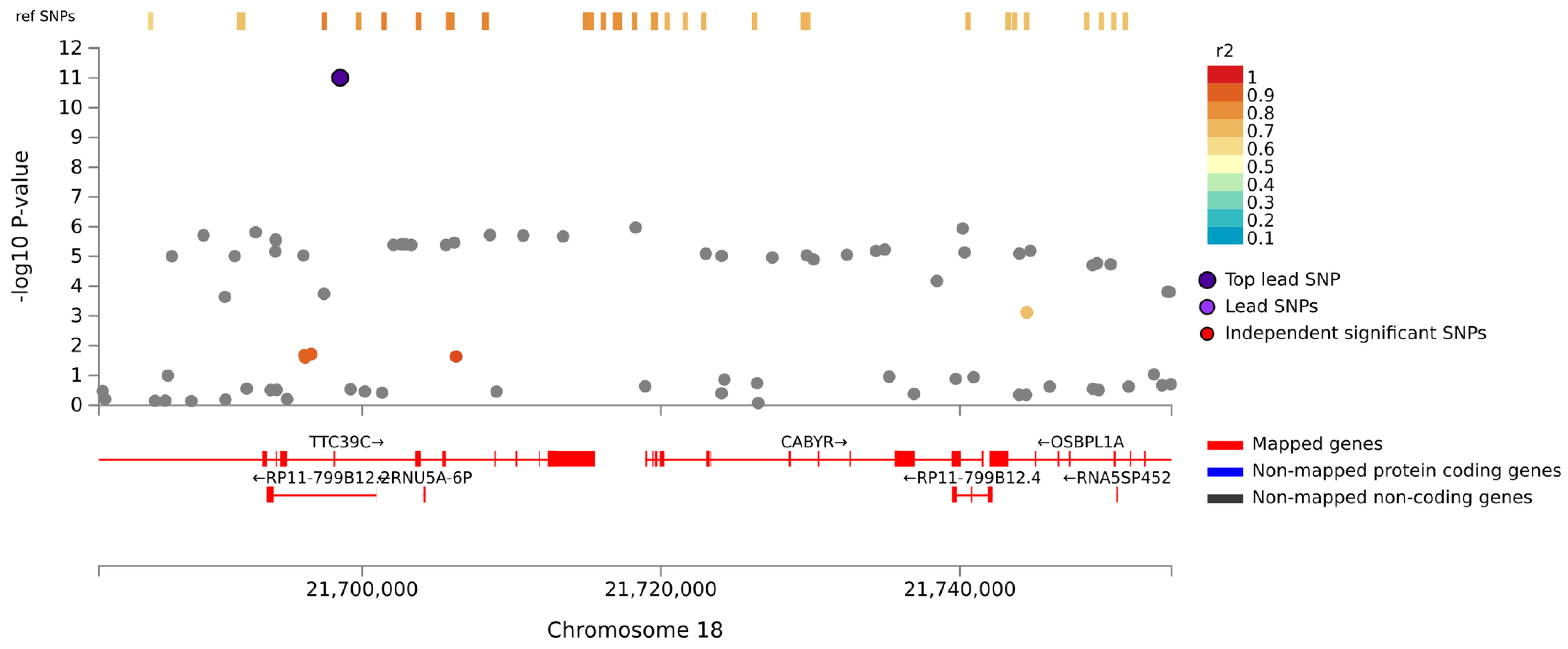

Locus 27, GAL3ST1, Total Area, rs2267158

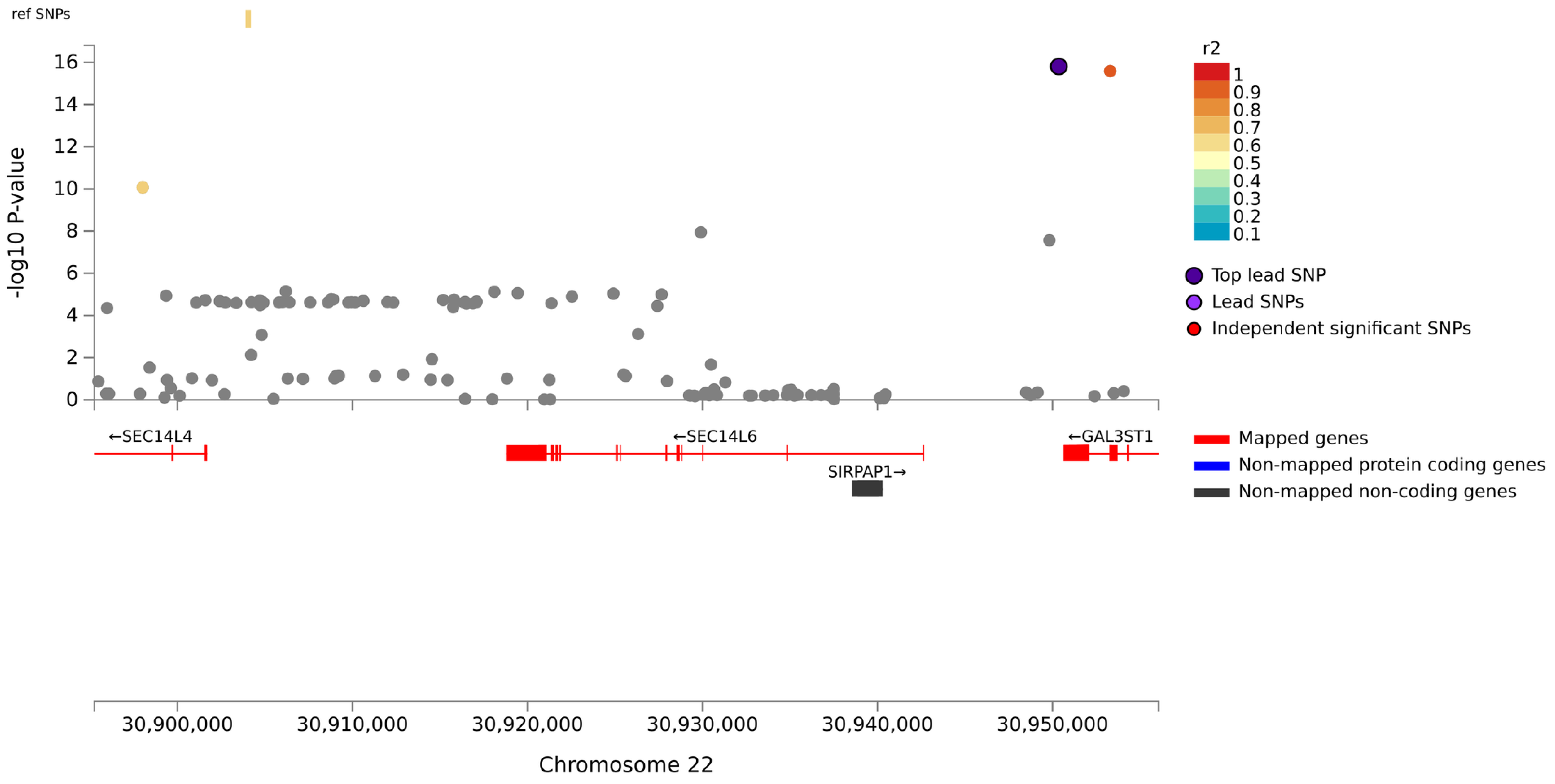

Locus 28, PICK1:RP5-1039K5.13, Total Area, rs760975

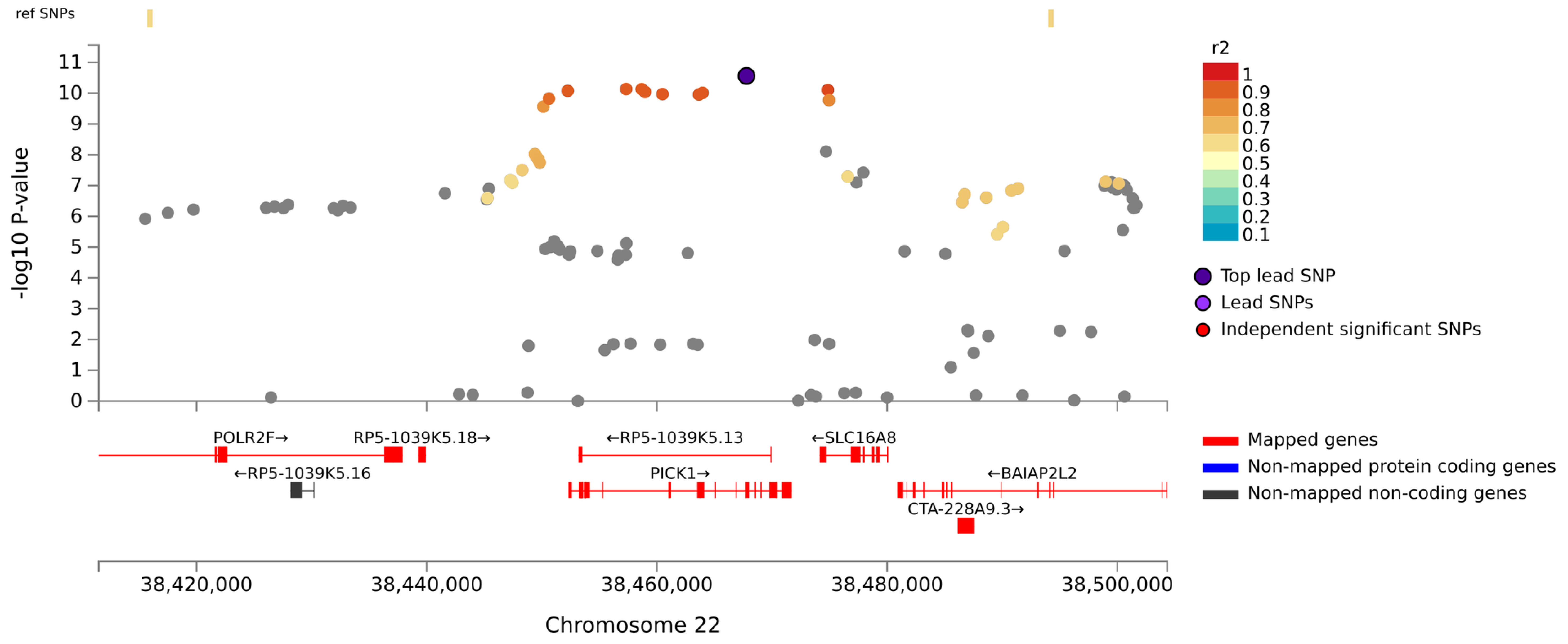

Locus 1, Y_RNA, Total Mean Thickness, rs9427220

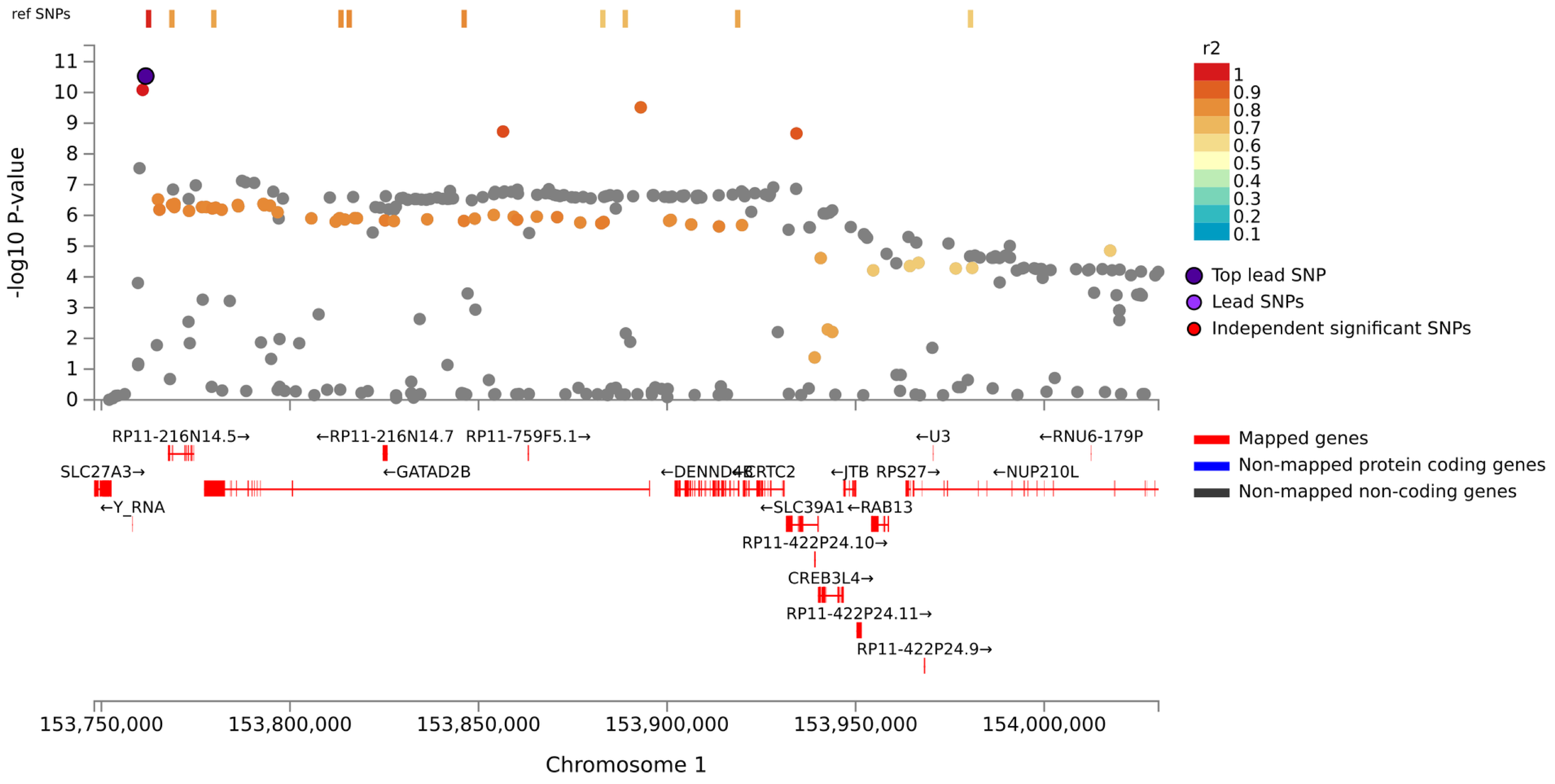

Locus 2, IQCJ-SCHIP1:IQCJ, Total Mean Thickness, rs12632564

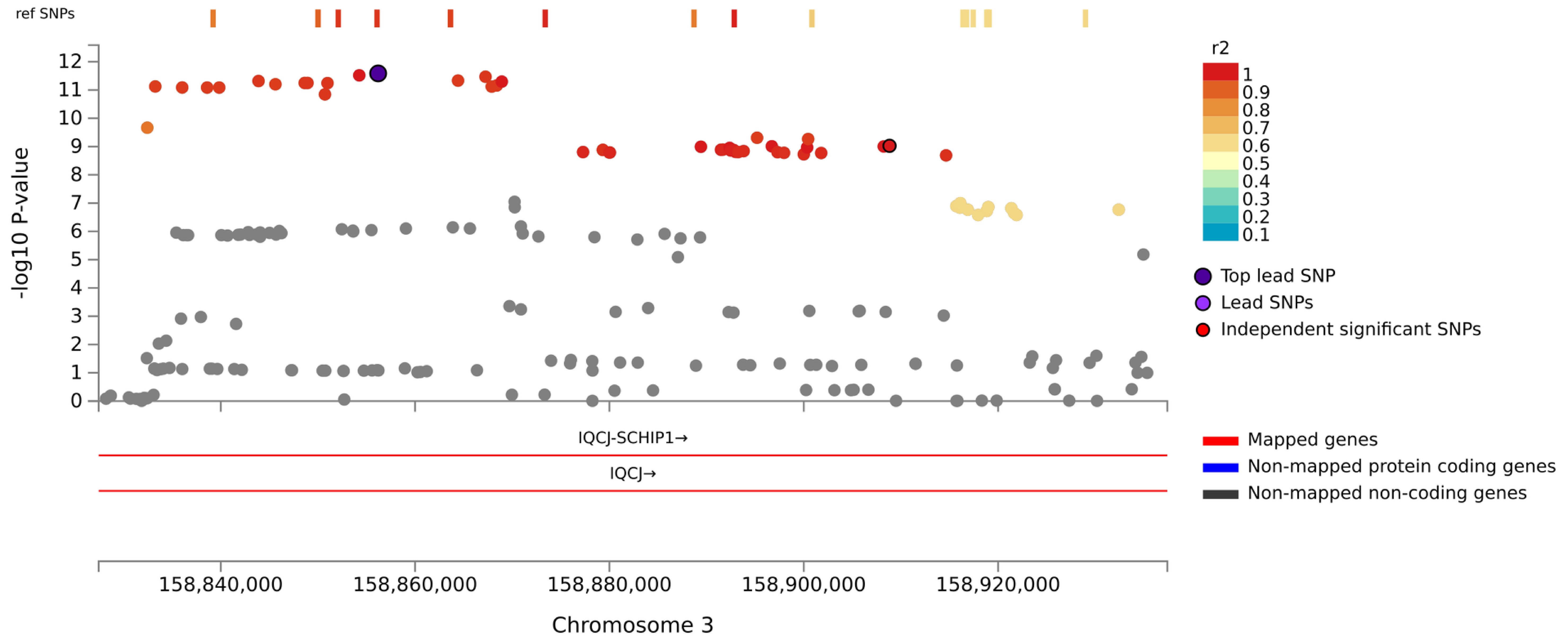

Locus 3, FIP1L1, Total Mean Thickness, rs6849897

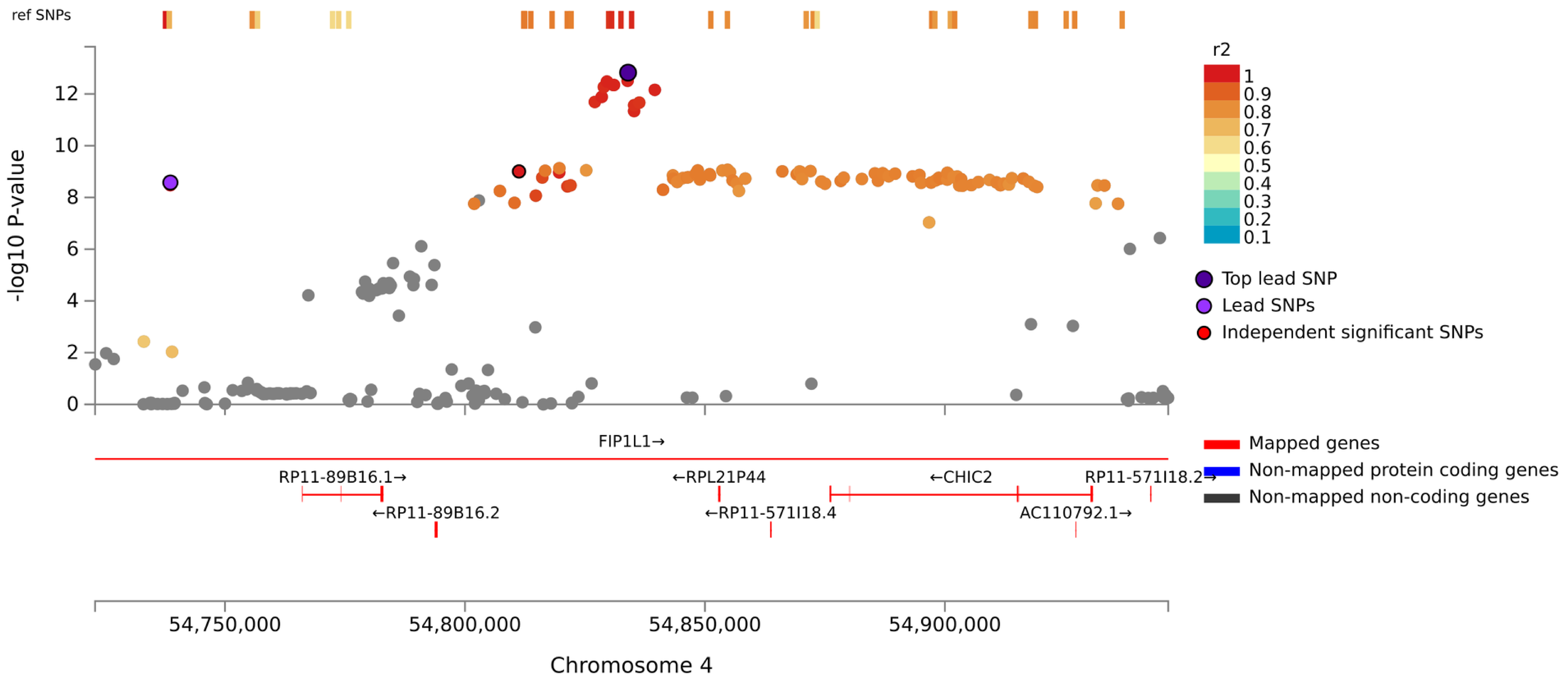

Locus 4, HBEGF, Total Mean Thickness, rs4150211

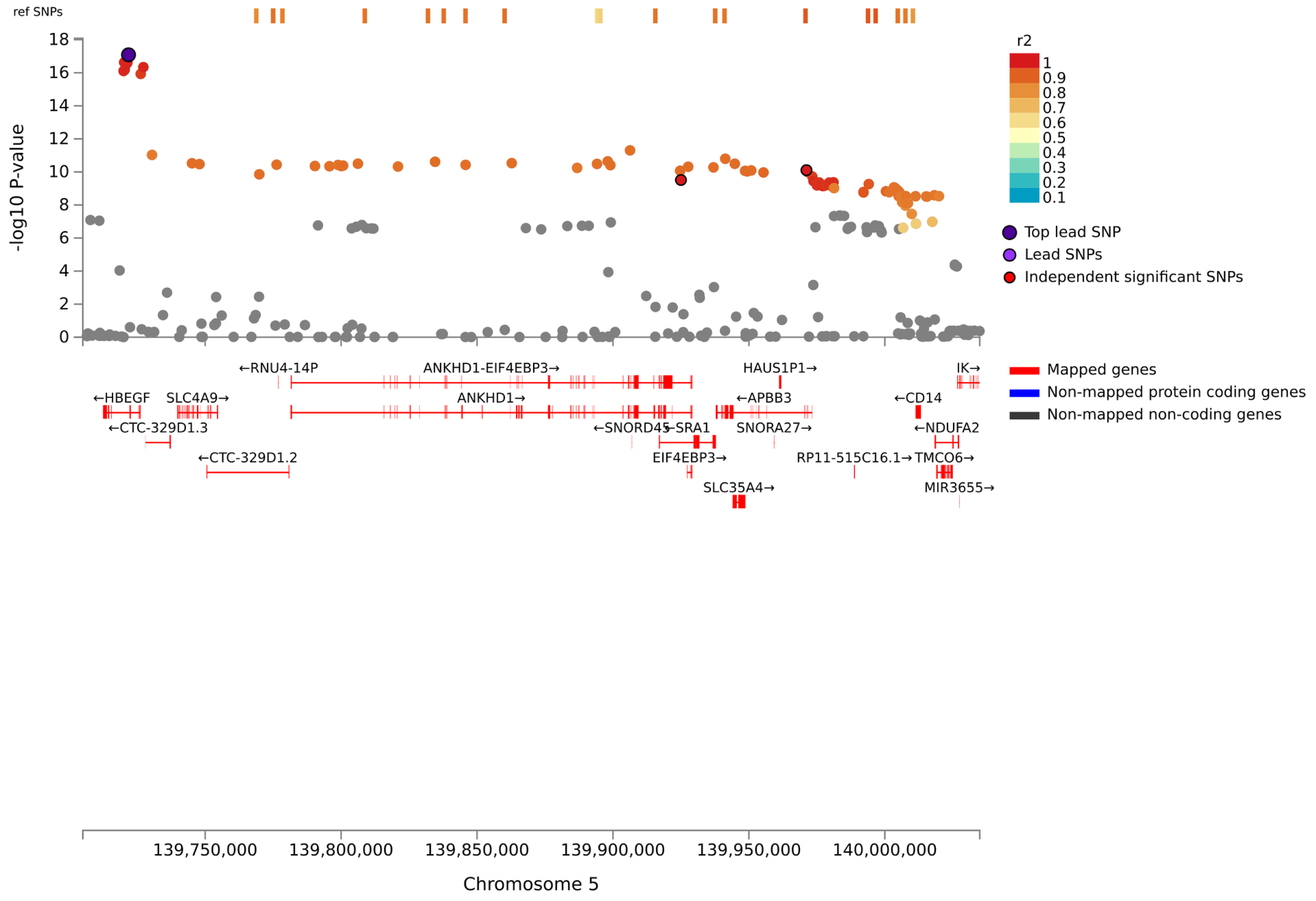

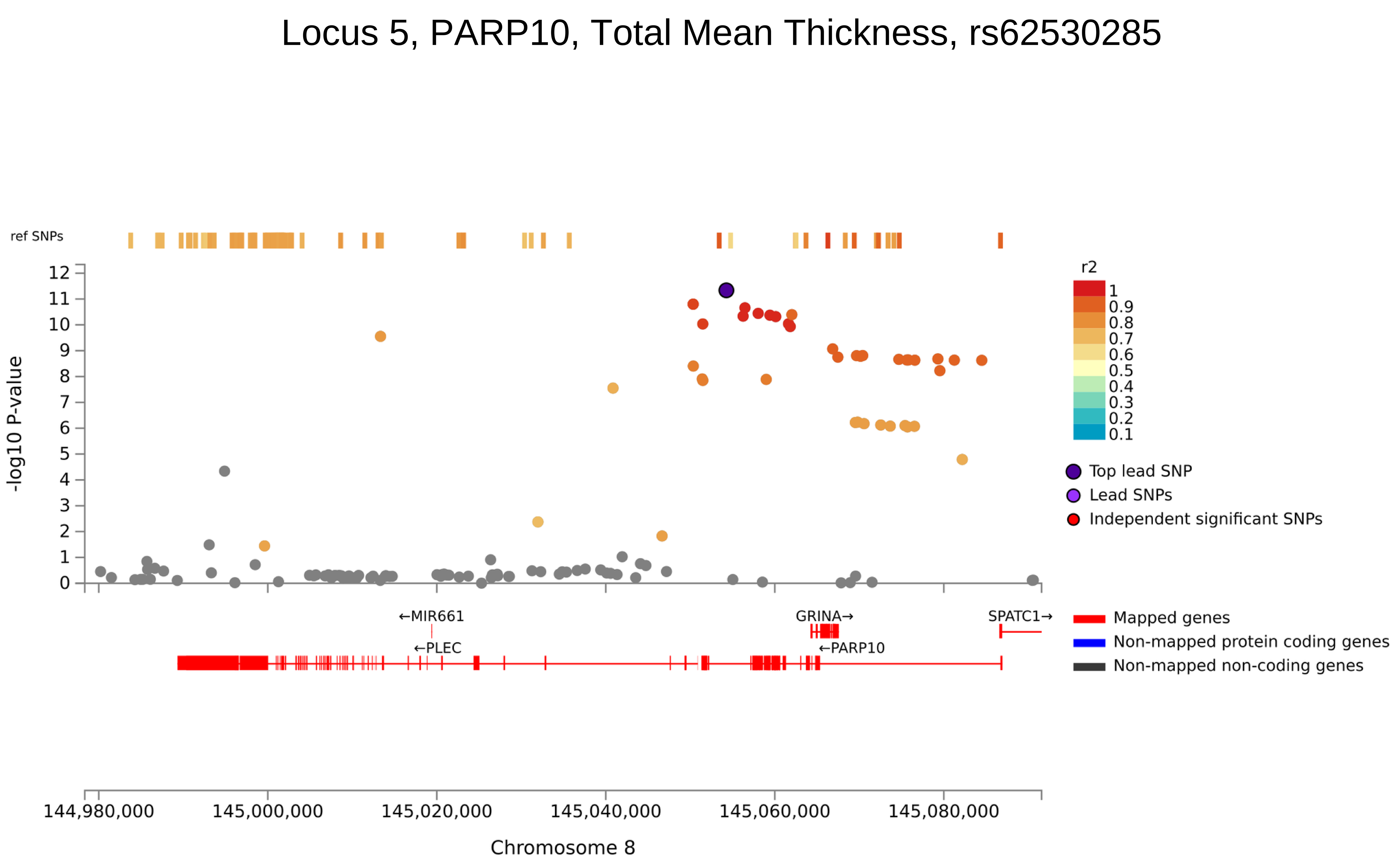

Locus 5, PARP10, Total Mean Thickness, rs62530285
ref SNPs
-log10 P-value
r2
Top lead SNP
Lead SNPs
Independent significant SNPs
←MIR661
←PLEC
GRINA→
←PARP10
SPATC1→
Mapped genes
Non-mapped protein coding genes
Non-mapped non-coding genes
144,980,000
145,000,000
145,020,000
145,040,000
145,060,000
145,080,000
Chromosome 8

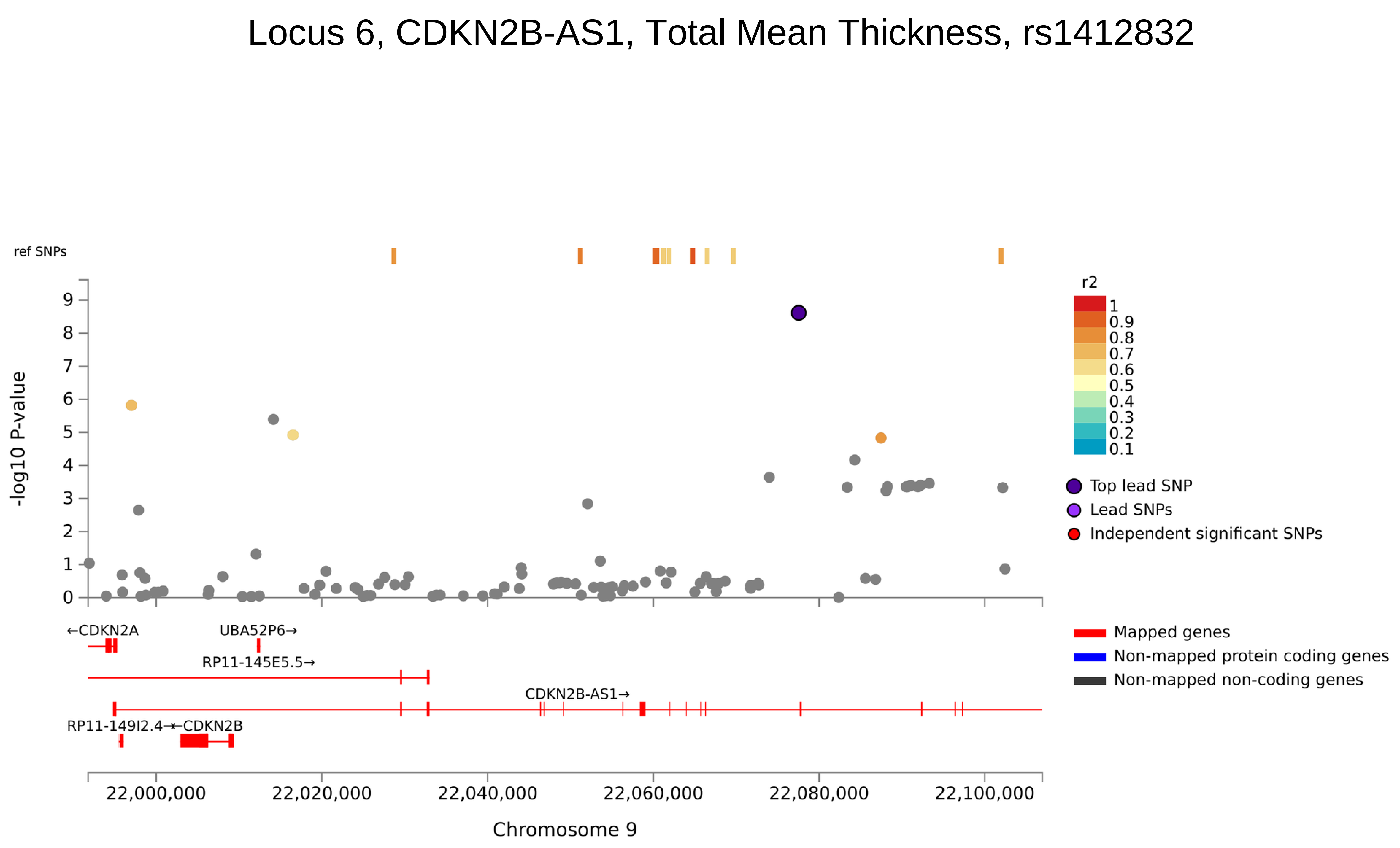

Locus 6, CDKN2B-AS1, Total Mean Thickness, rs1412832
ref SNPs
-log10 P-value
r2
Top lead SNP
Lead SNPs
Independent significant SNPs
←CDKN2A
UBA52P6→
RP11-145E5.5→
CDKN2B-AS1→
RP11-149I2.4→
←CDKN2B
Mapped genes
Non-mapped protein coding genes
Non-mapped non-coding genes
22,000,000
22,020,000
22,040,000
22,060,000
22,080,000
22,100,000
Chromosome 9

Locus 7, FAM107B, Total Mean Thickness, rs878733

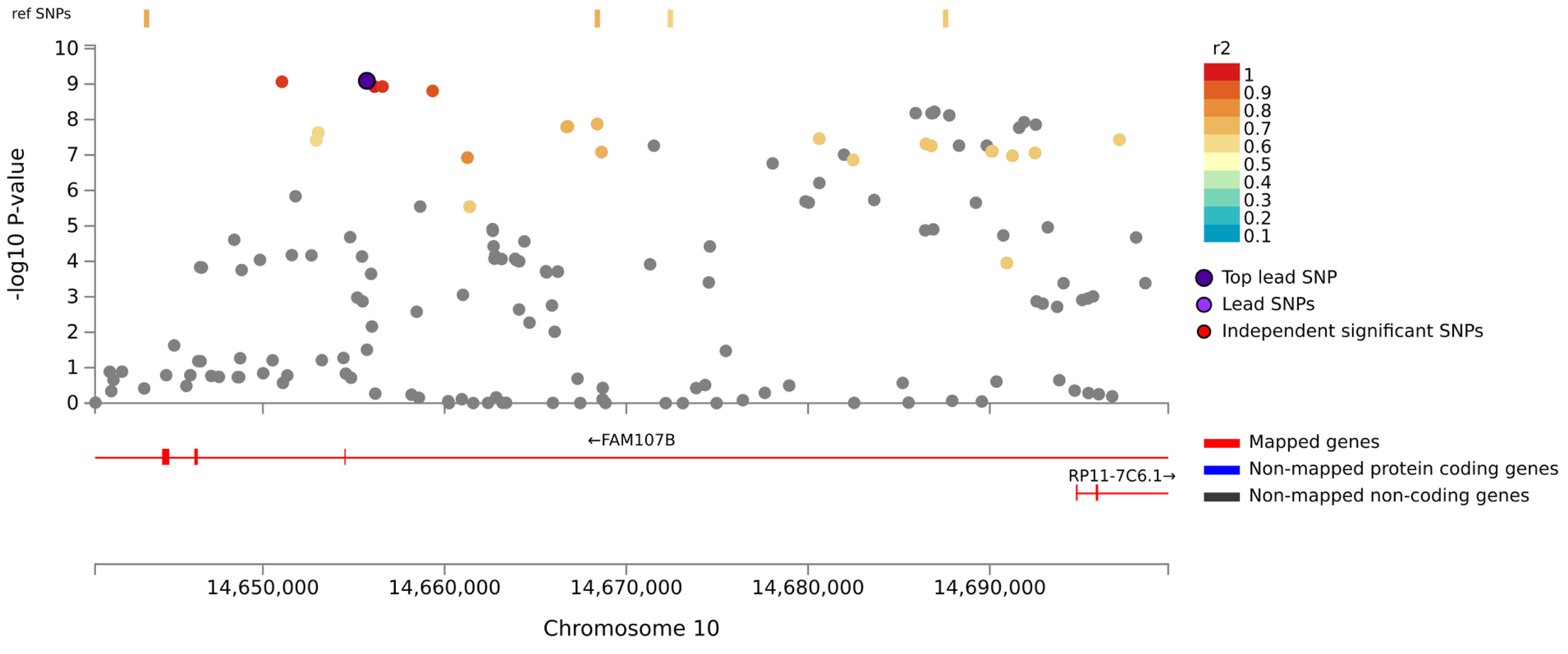

Locus 8, C16orf95, Total Mean Thickness, rs4843550

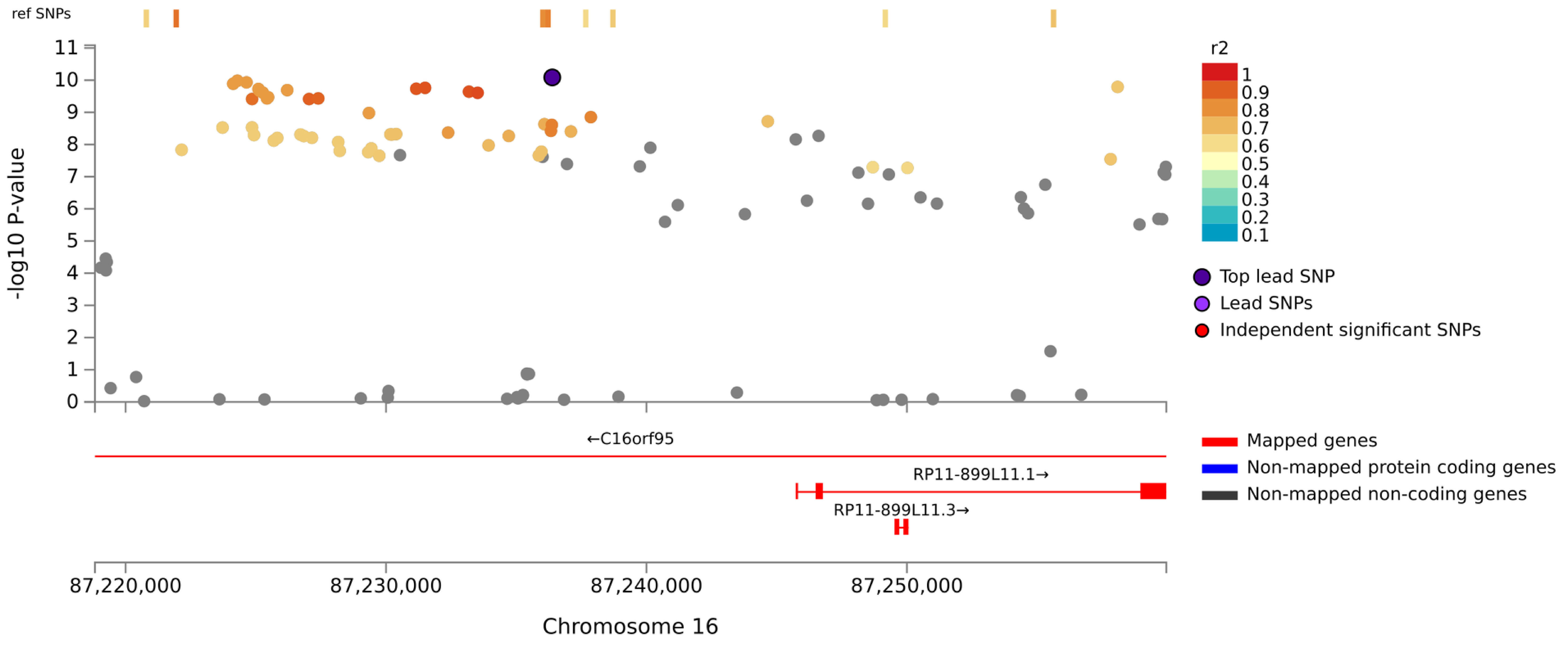

Locus 9, CABYR, Total Mean Thickness, rs752797

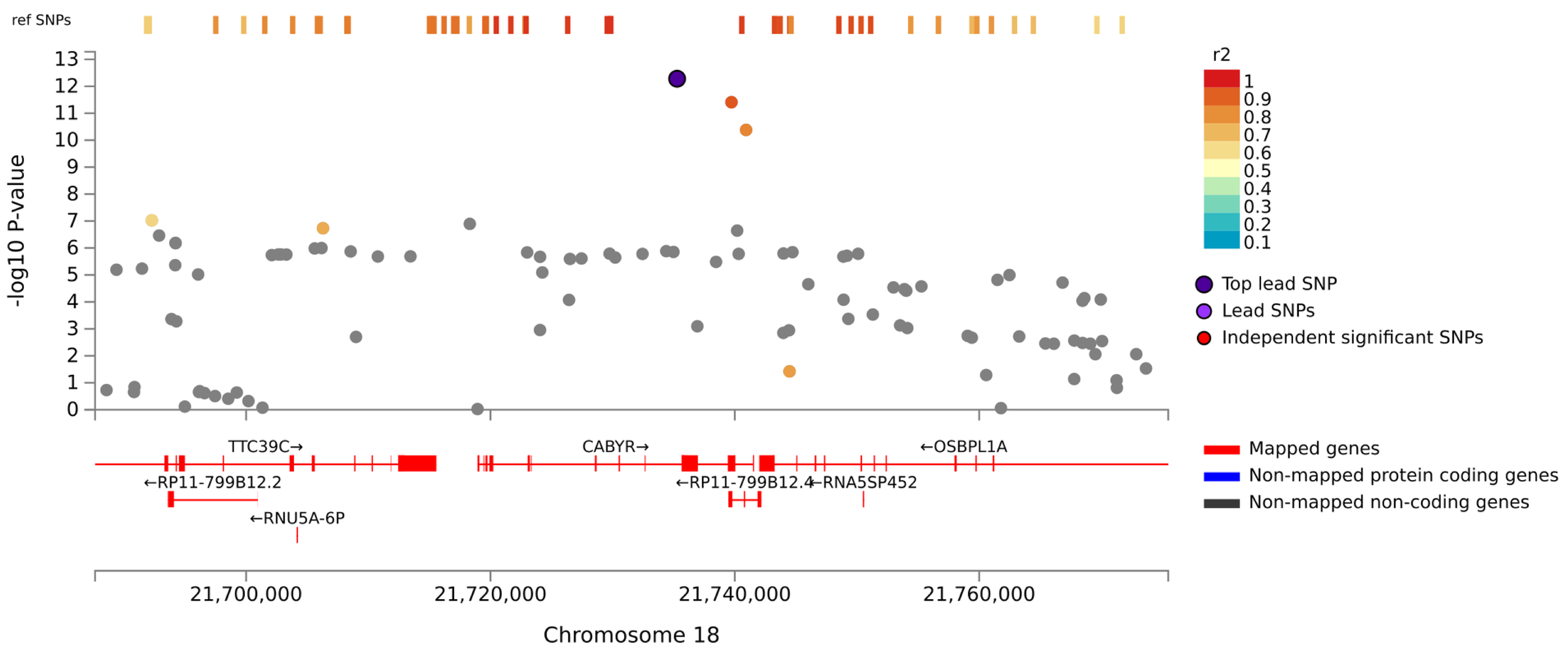

Locus 1, STX6, Genu Area, rs35306826

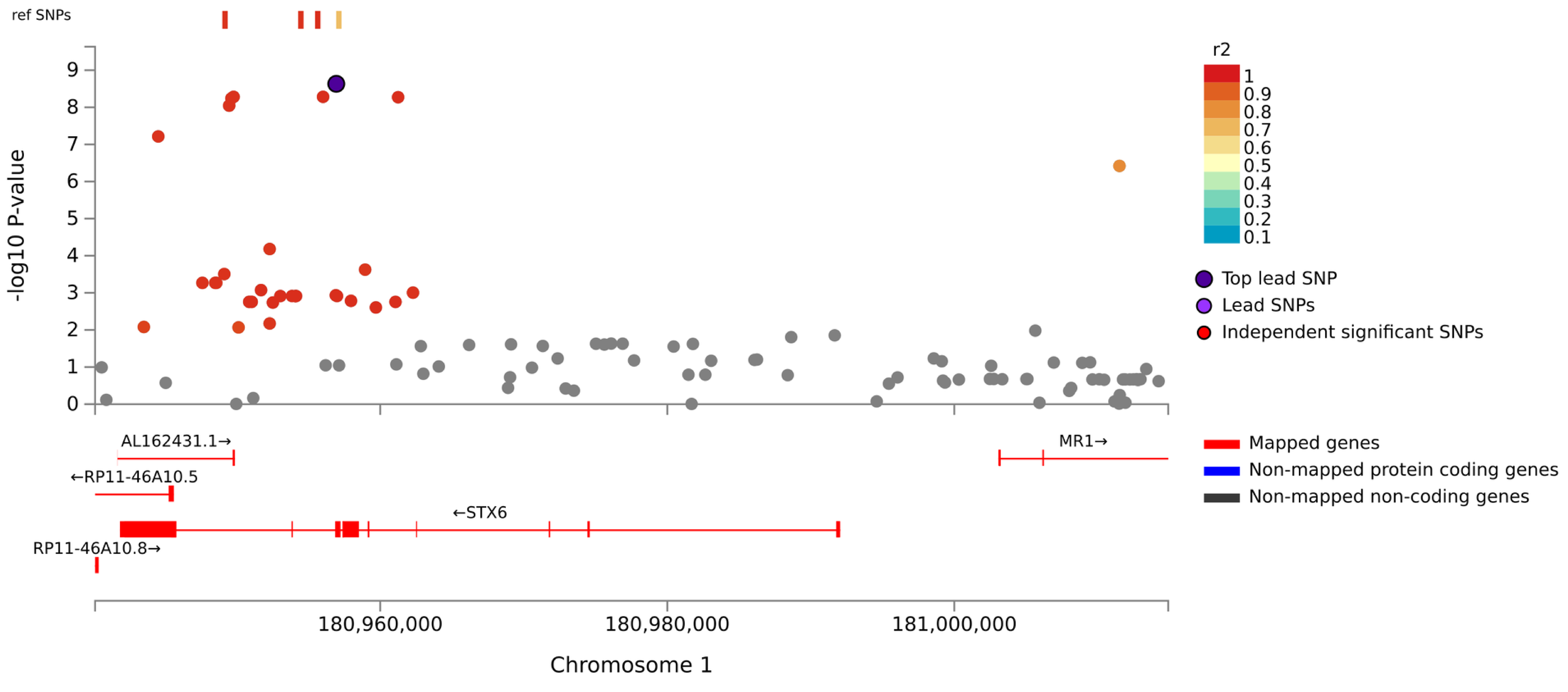

Locus 2, STRN, Genu Area, rs62132550

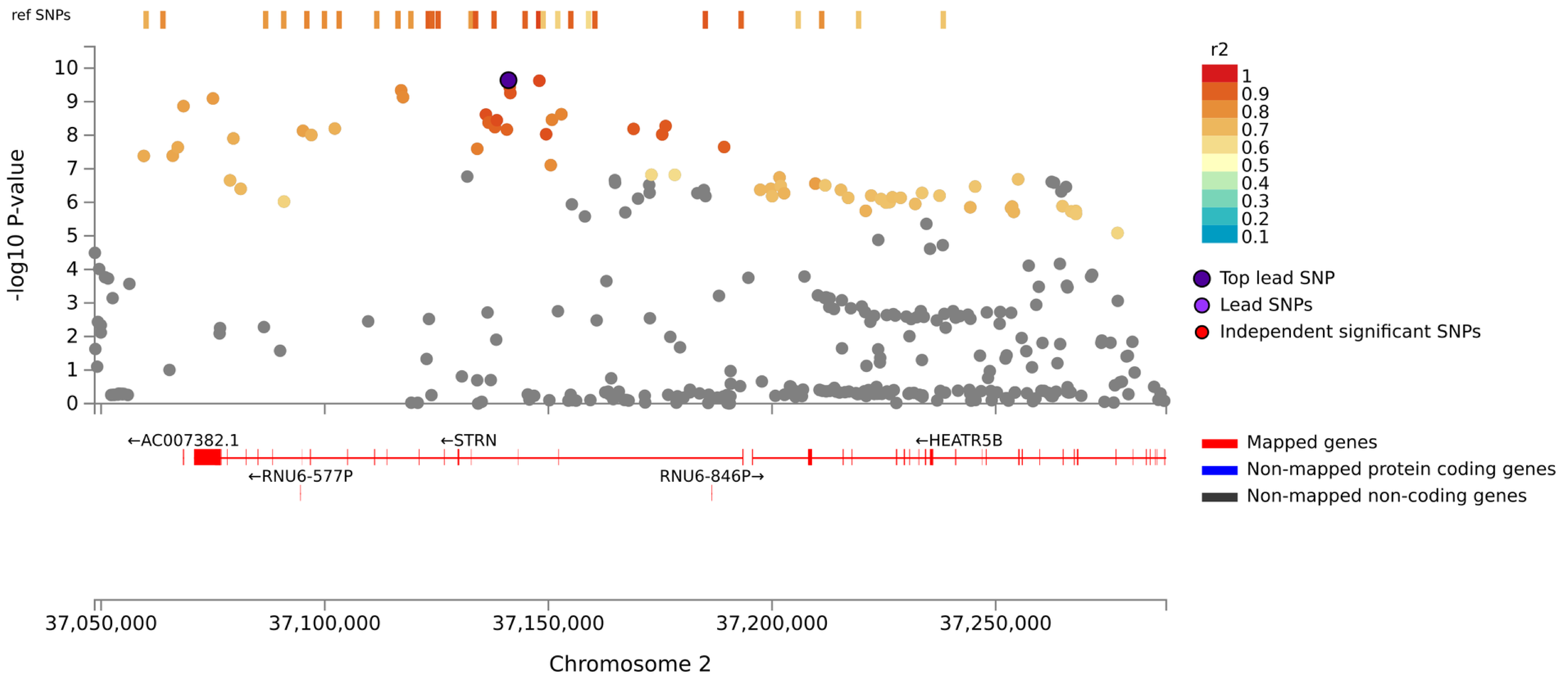

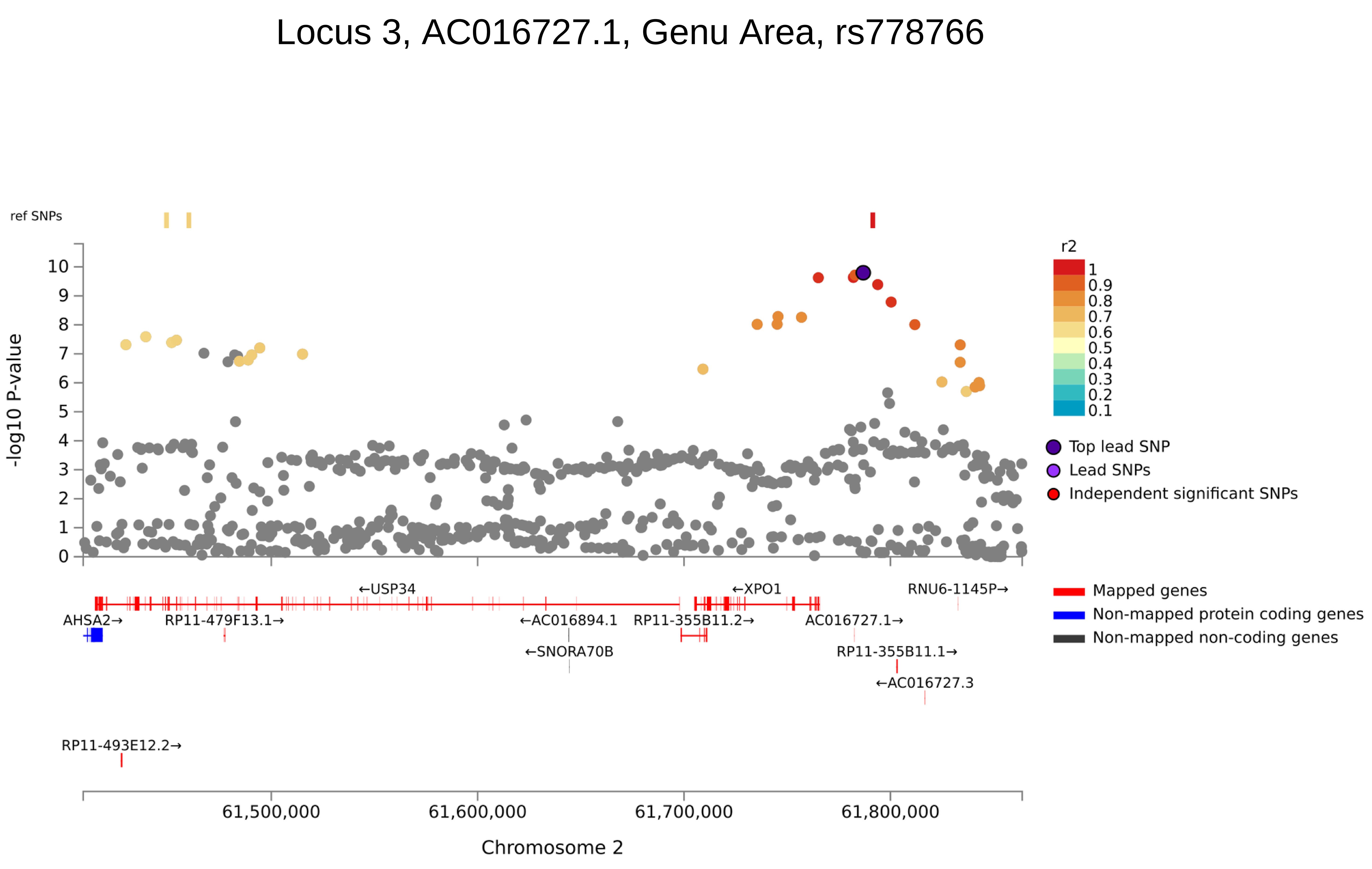

Locus 3, AC016727.1, Genu Area, rs778766
ref SNPs
-log10 P-value
r2
Top lead SNP
Lead SNPs
Independent significant SNPs
←USP34
←XPO1
RNU6-1145P→
AHSA2→
RP11-479F13.1→
←AC016894.1
RP11-355B11.2→
AC016727.1→
←SNORA70B
RP11-355B11.1→
←AC016727.3
RP11-493E12.2→
Mapped genes
Non-mapped protein coding genes
Non-mapped non-coding genes
61,500,000
61,600,000
61,700,000
61,800,000
Chromosome 2

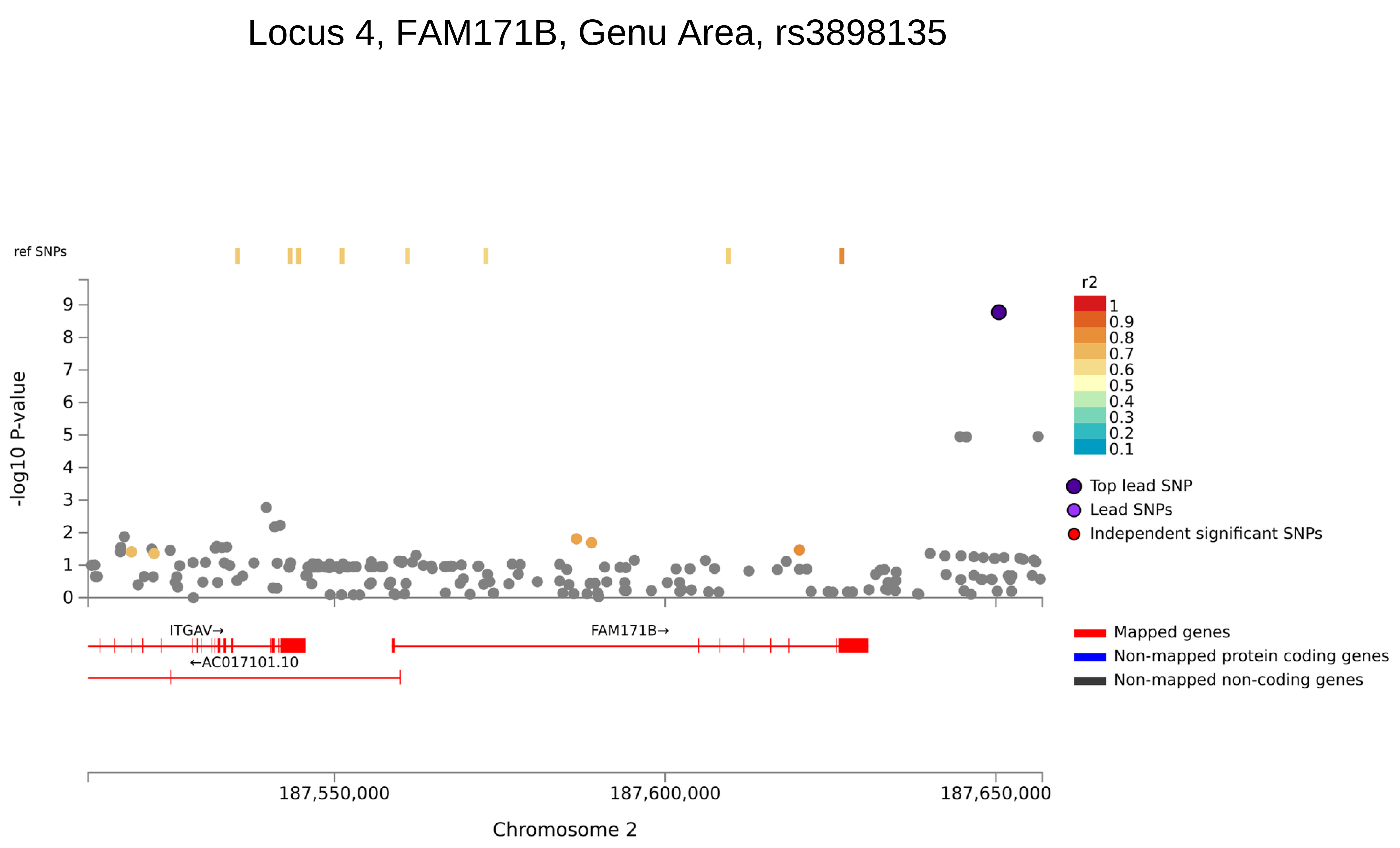

Locus 4, FAM171B, Genu Area, rs3898135
ref SNPs
-log10 P-value
r2
Top lead SNP
Lead SNPs
Independent significant SNPs
ITGAV→
←AC017101.10
FAM171B→
Mapped genes
Non-mapped protein coding genes
Non-mapped non-coding genes
187,550,000
187,600,000
187,650,000
Chromosome 2

Locus 5, RPL37A, Genu Area, rs62180637

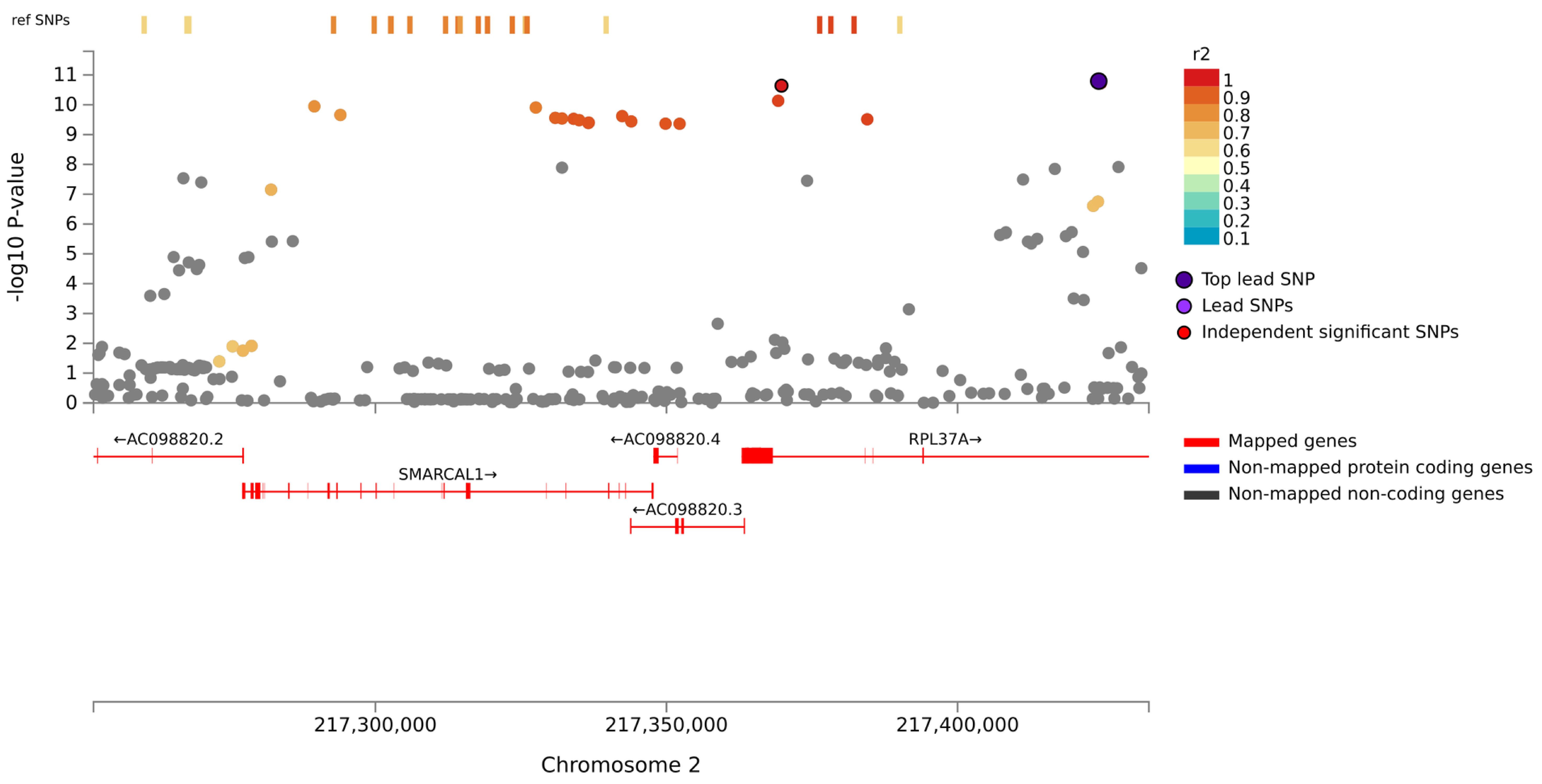

Locus 6, IQCJ-SCHIP1:IQCJ, Genu Area, rs12632564

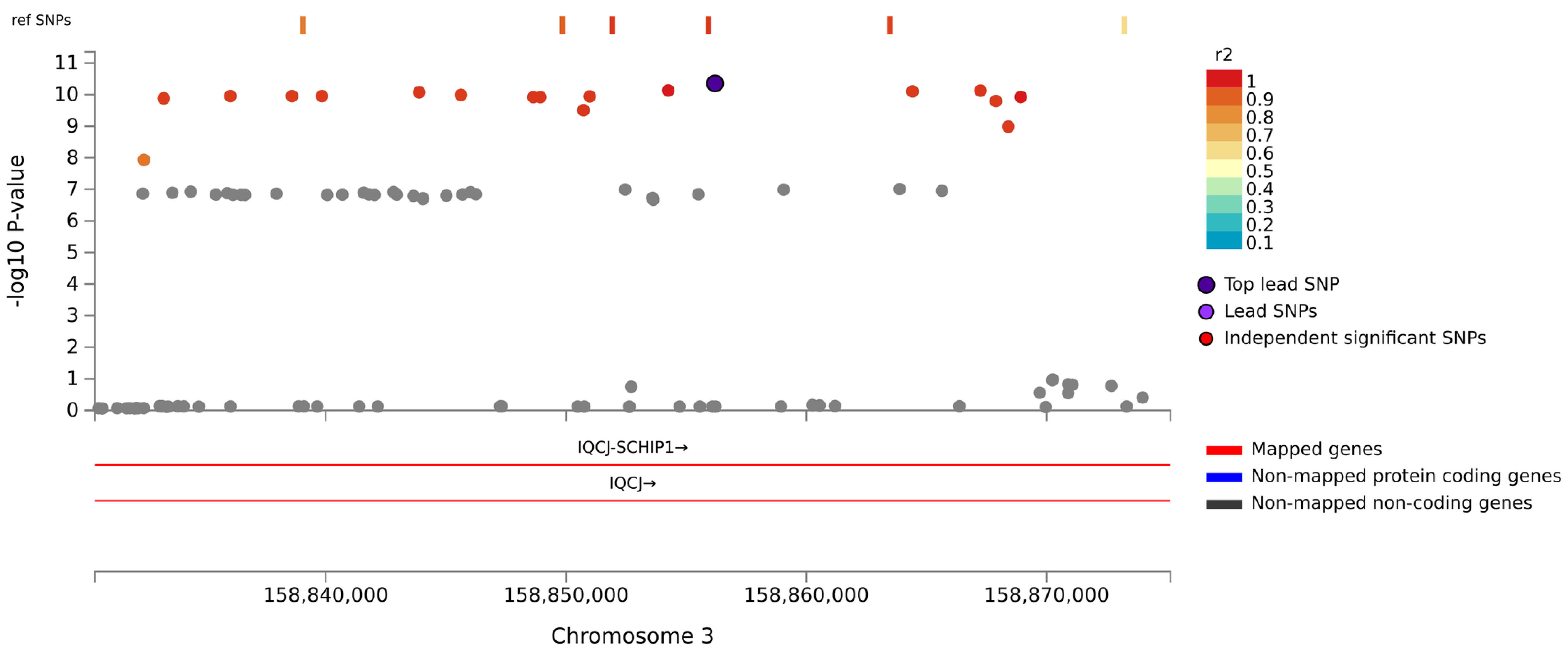

Locus 7, CTC-448D22.1, Genu Area, rs721065

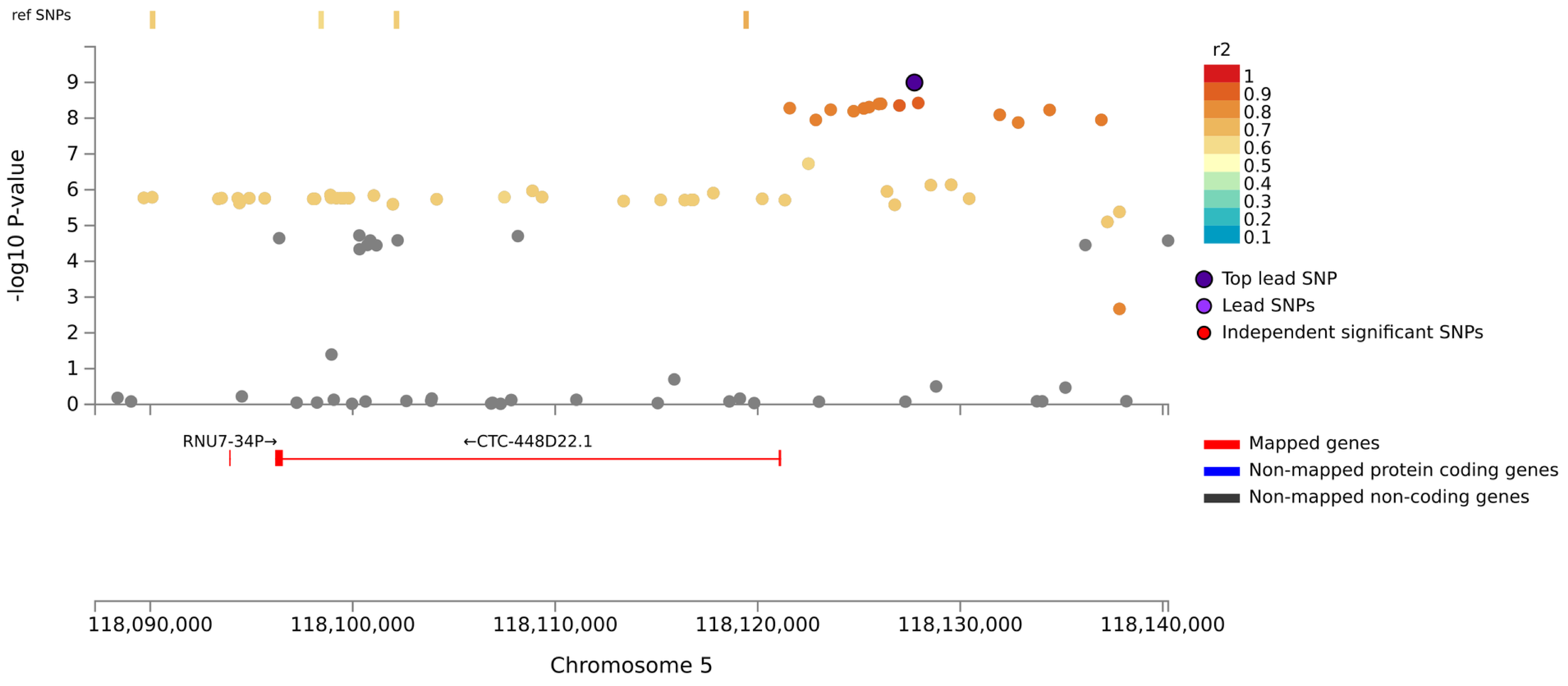

Locus 8, SNORA73, Genu Area, rs76928645

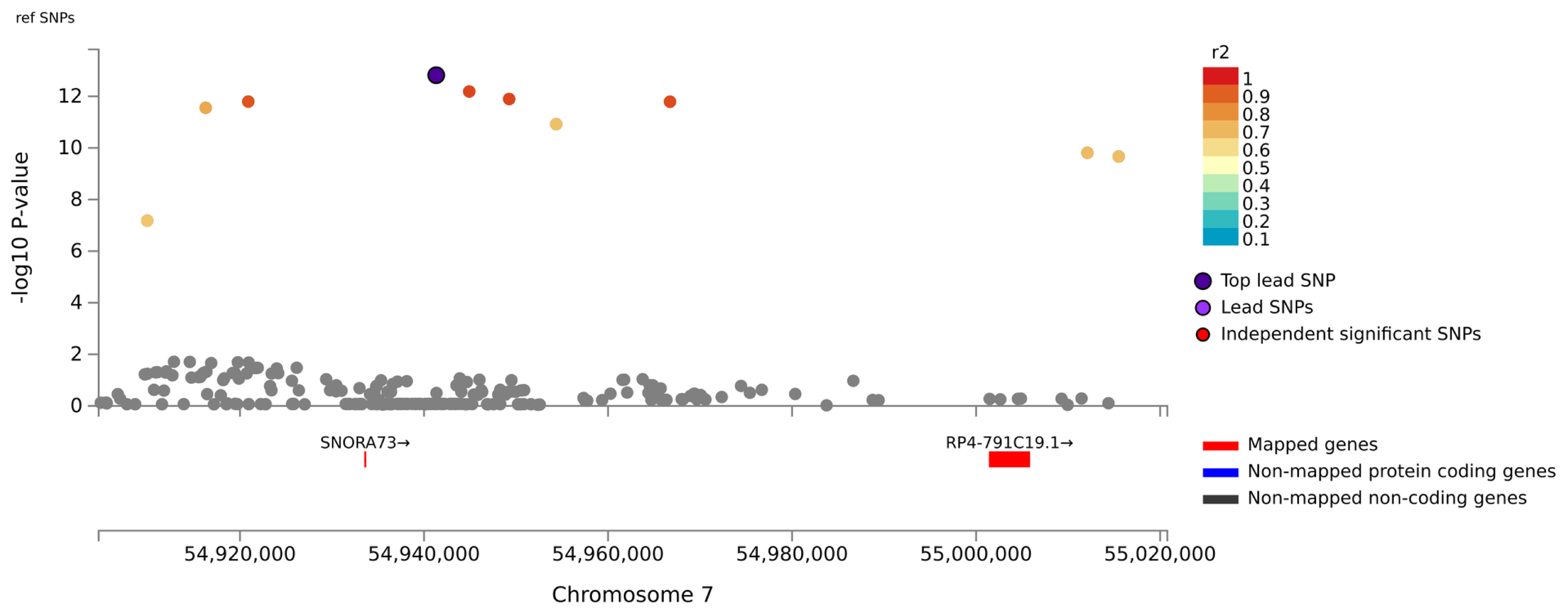

Locus 9, AC003084.2, Genu Area, rs7784849

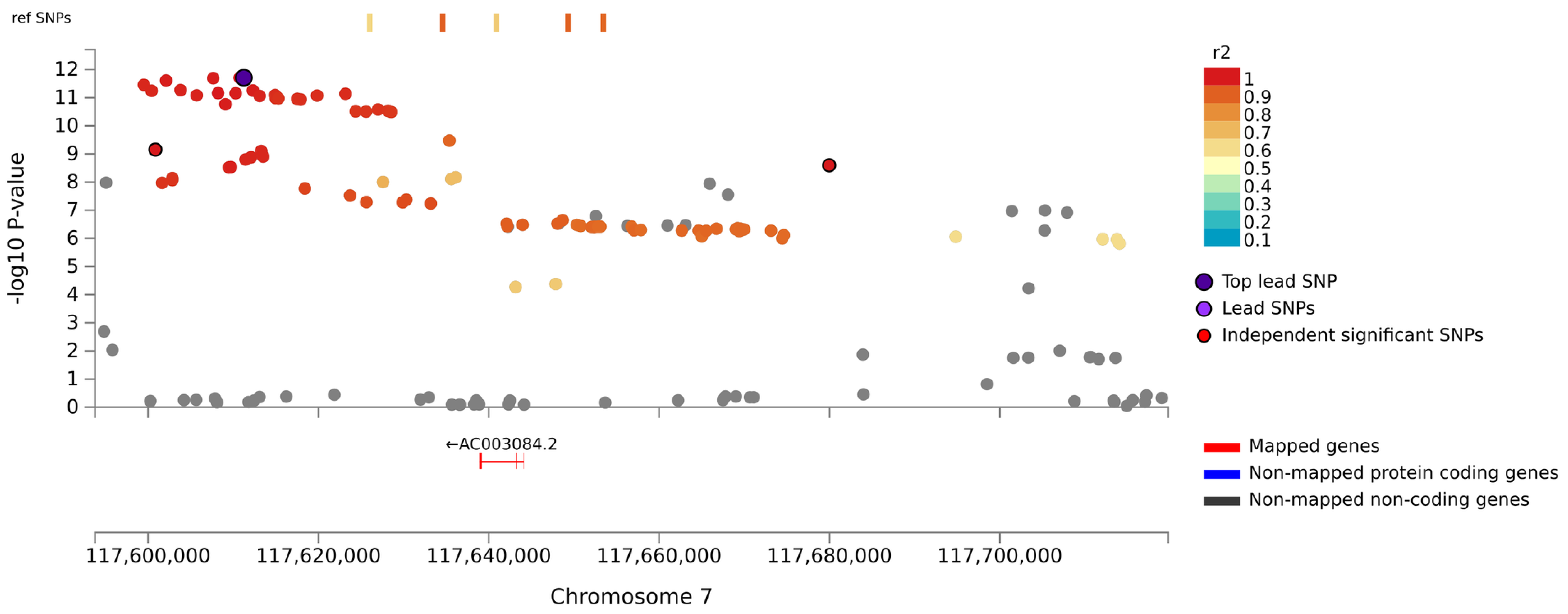

Locus 10, PLEC, Genu Area, rs56401356

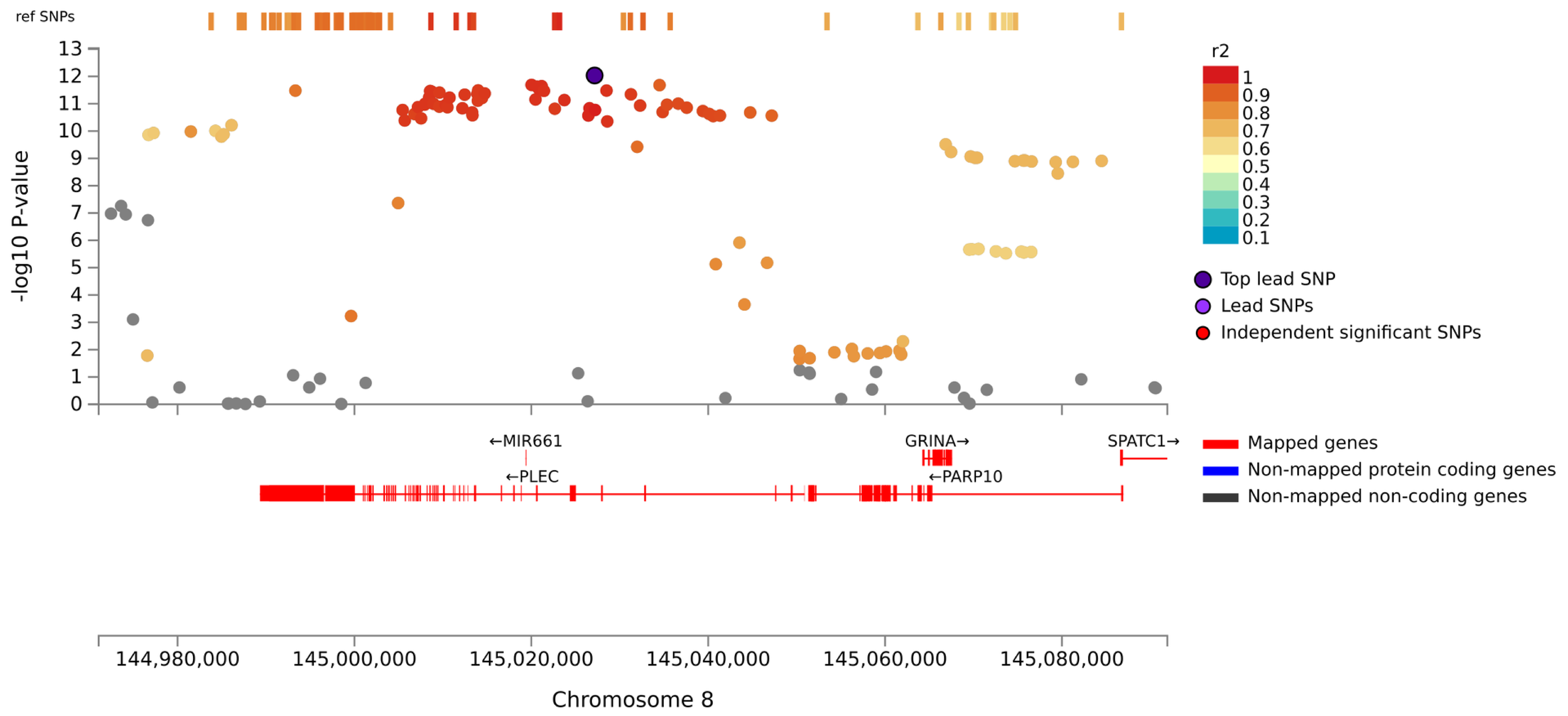

Locus 11, KIAA1598, Genu Area, rs1122688

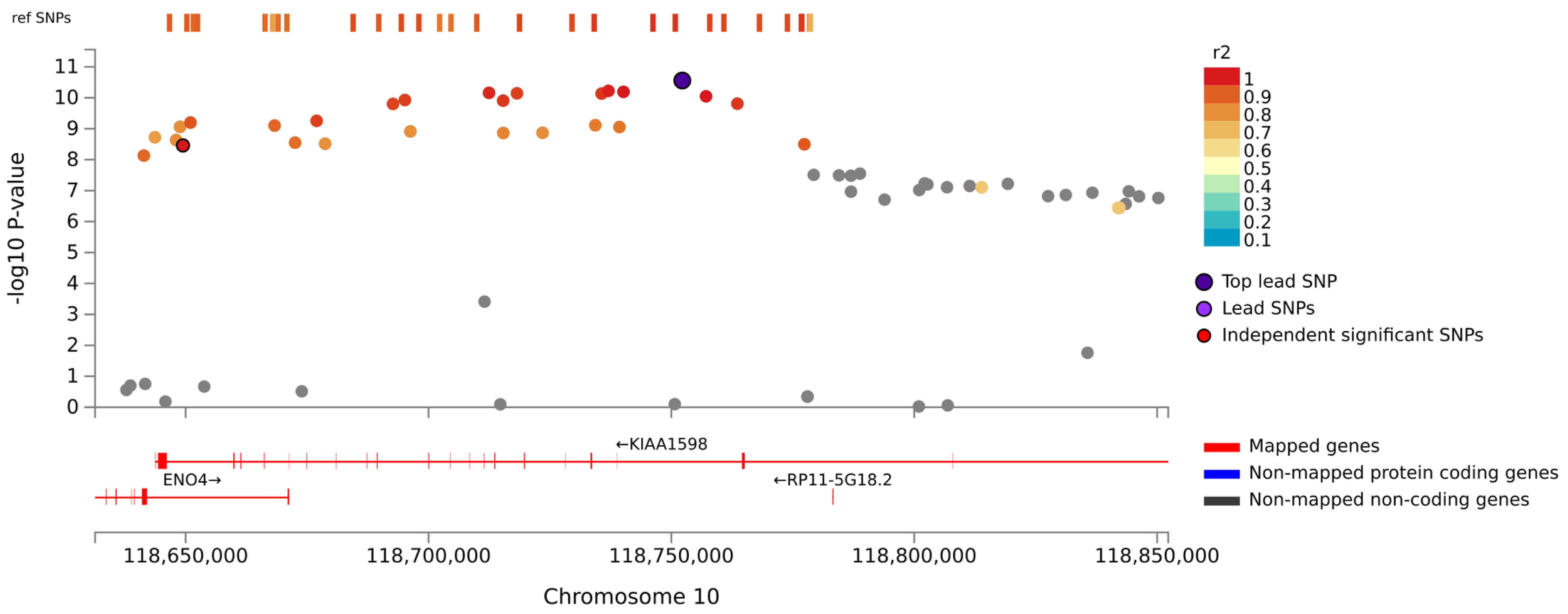

Locus 12, NAV2, Genu Area, rs2625302

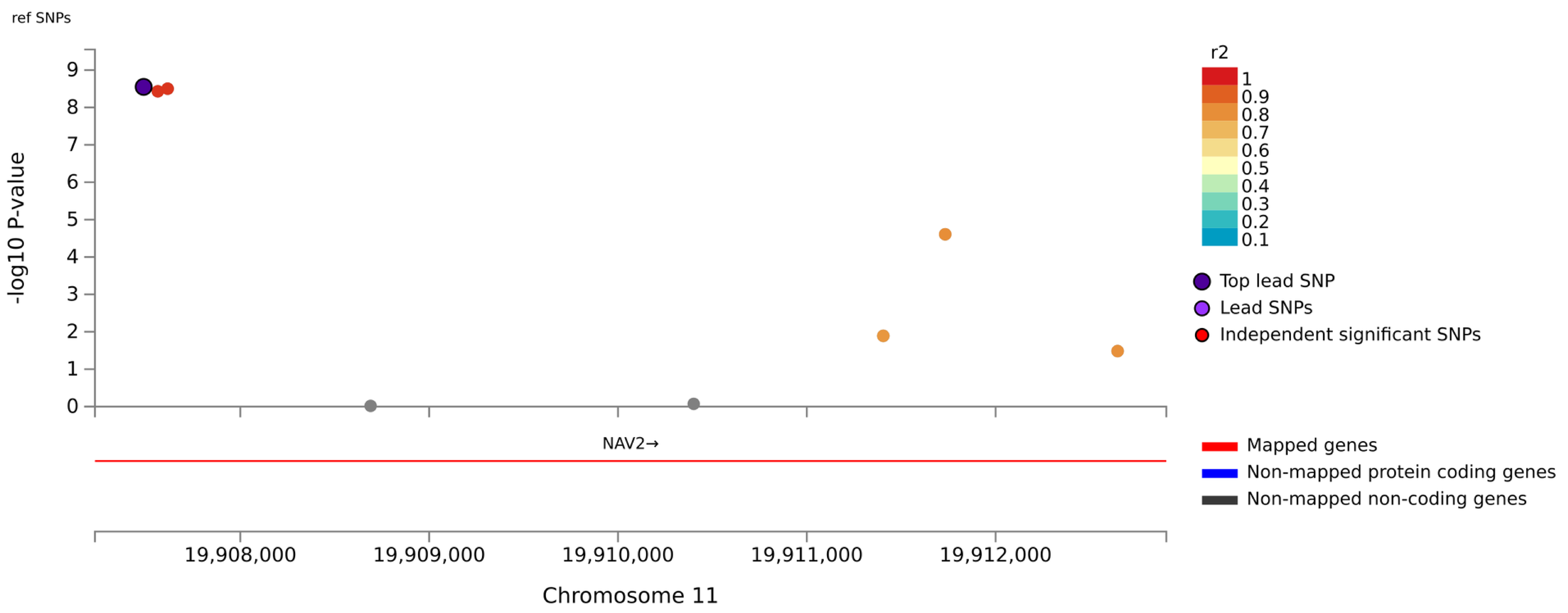

Locus 13, CELF1, Genu Area, rs11039266

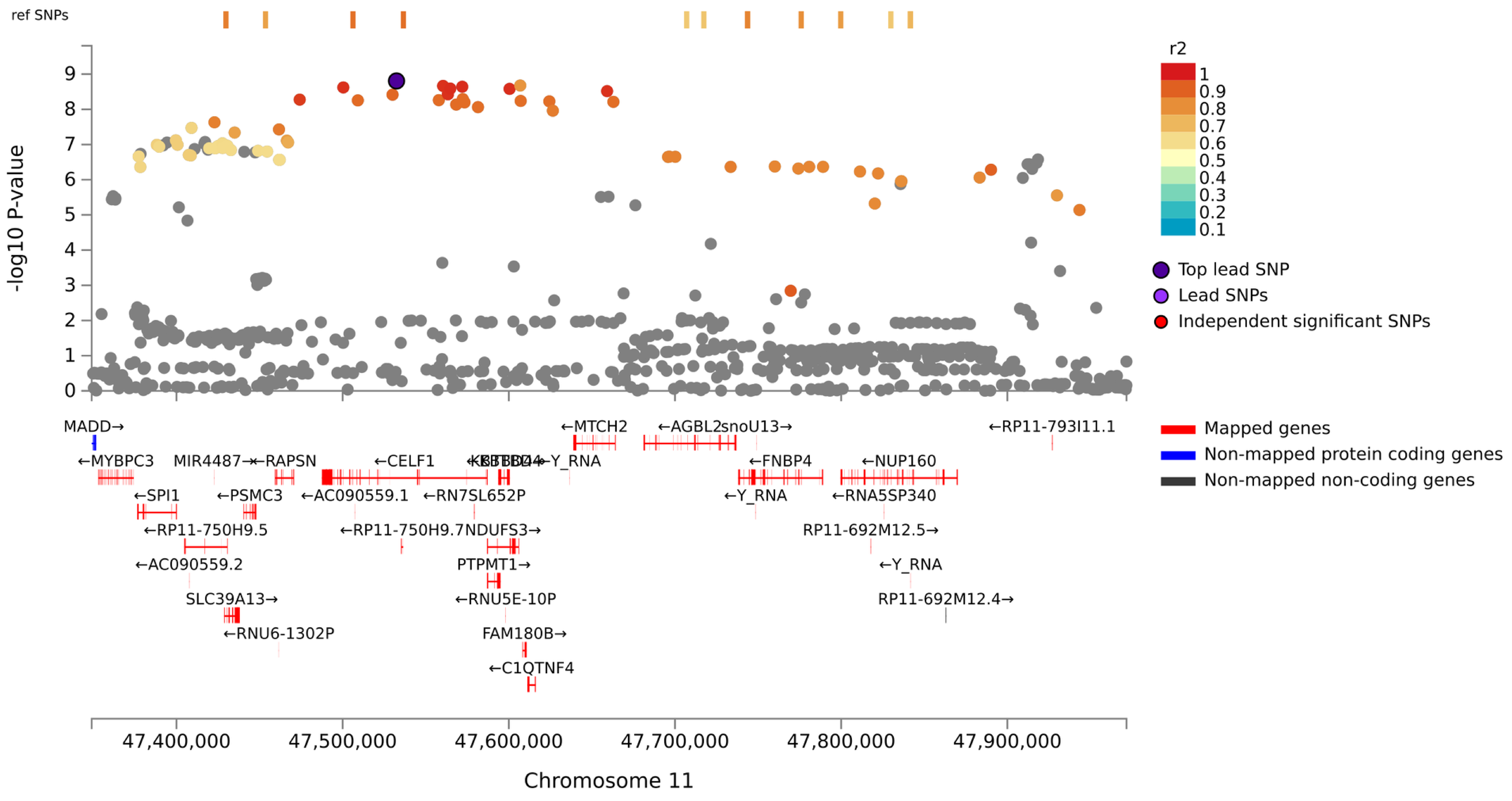

Locus 14, NAV3, Genu Area, rs10506772

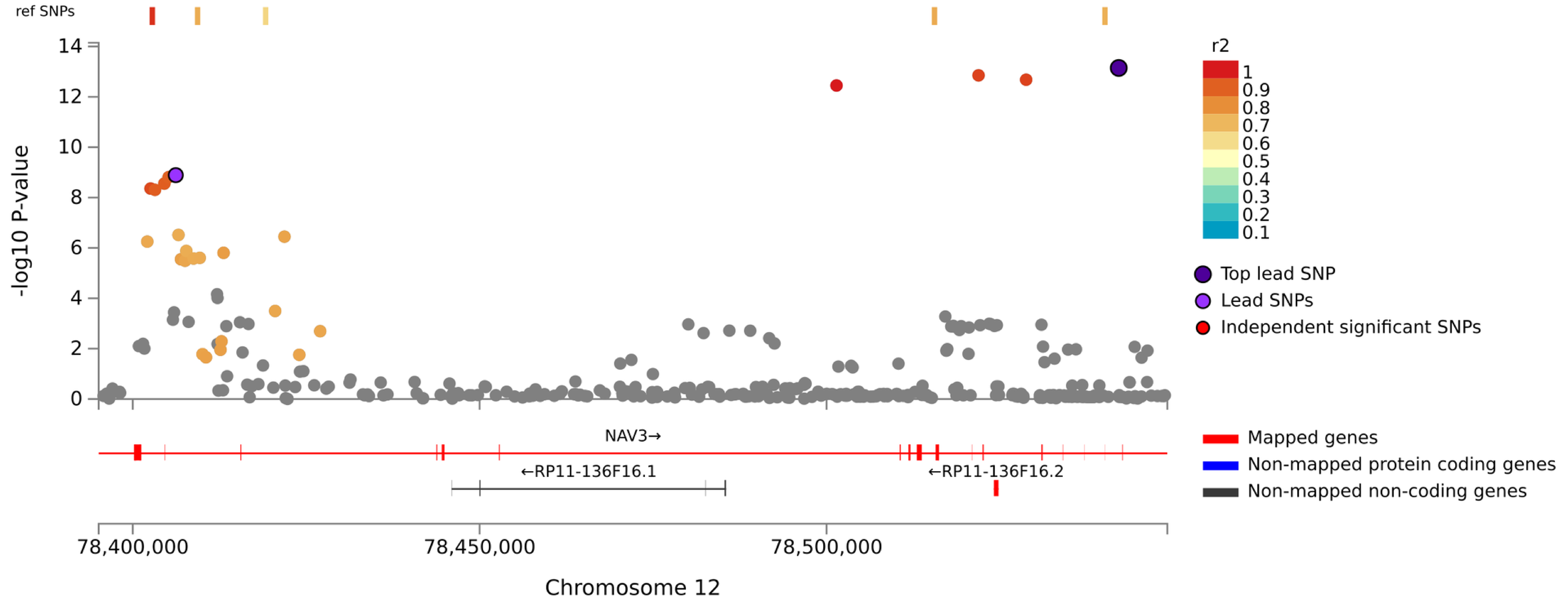

Locus 15, KANSL1, Genu Area, rs2532402

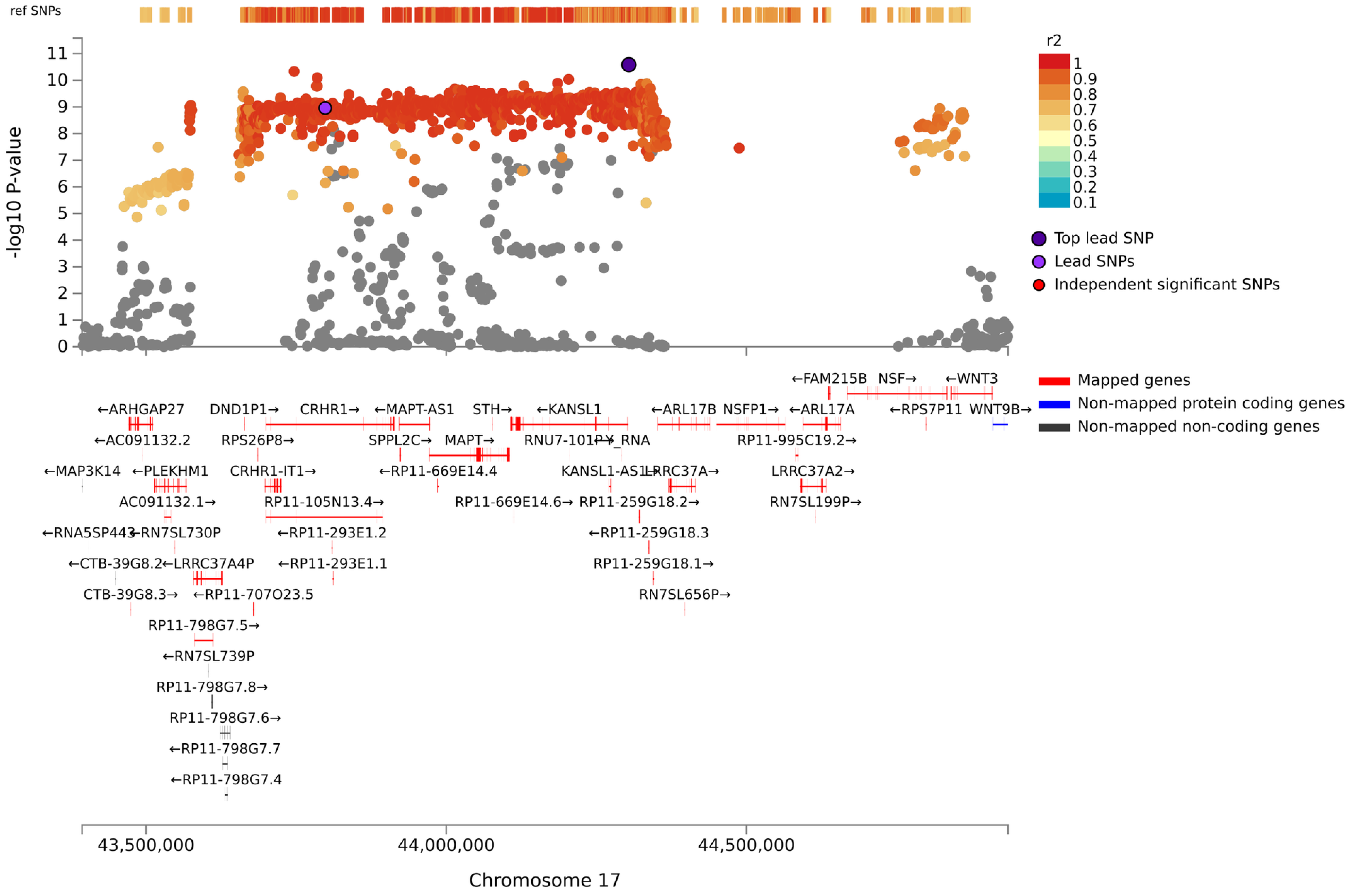

Locus 16, GAL3ST1, Genu Area, rs2267161

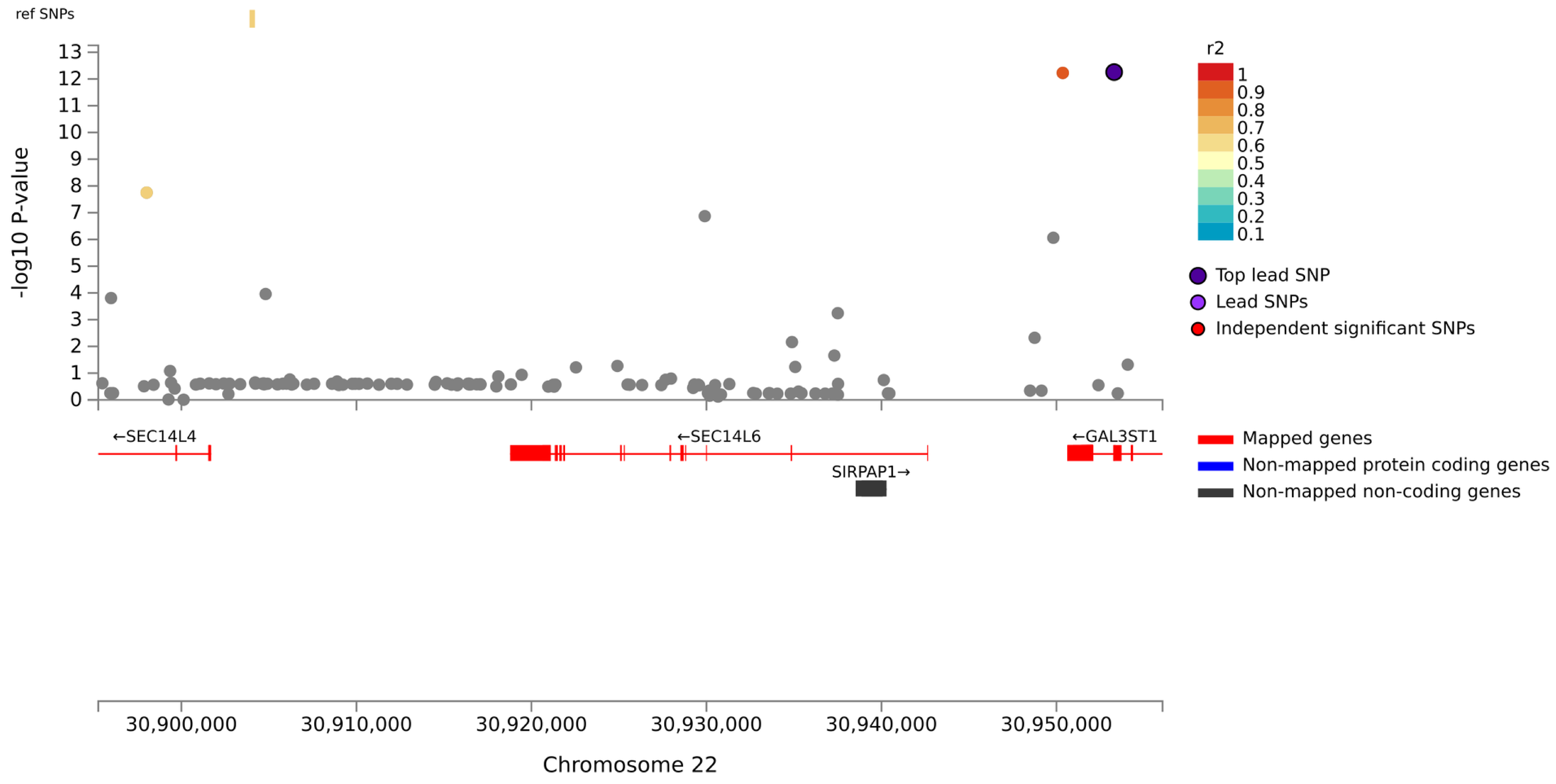

Locus 17, PICK1:RP5-1039K5.13, Genu Area, rs738443

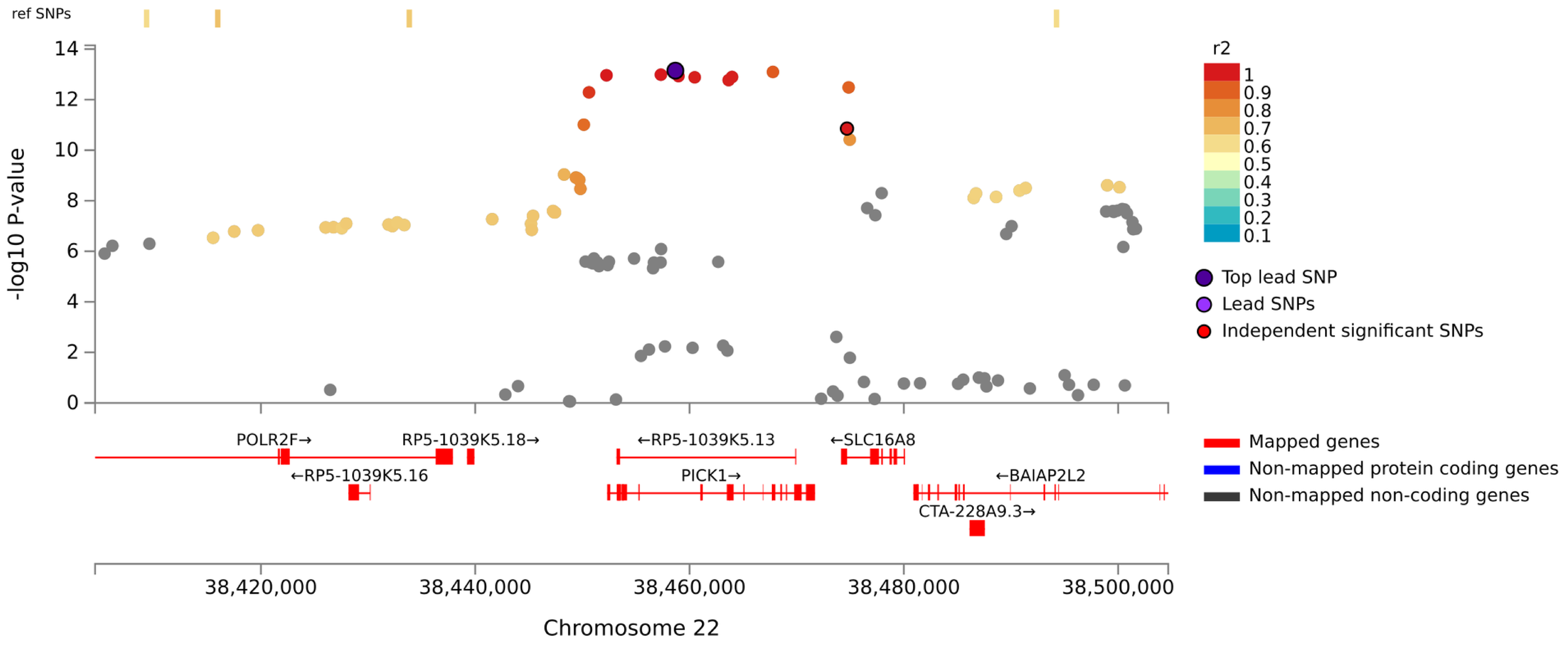

Locus 1, SDHB, Anterior Body Area, rs3754507

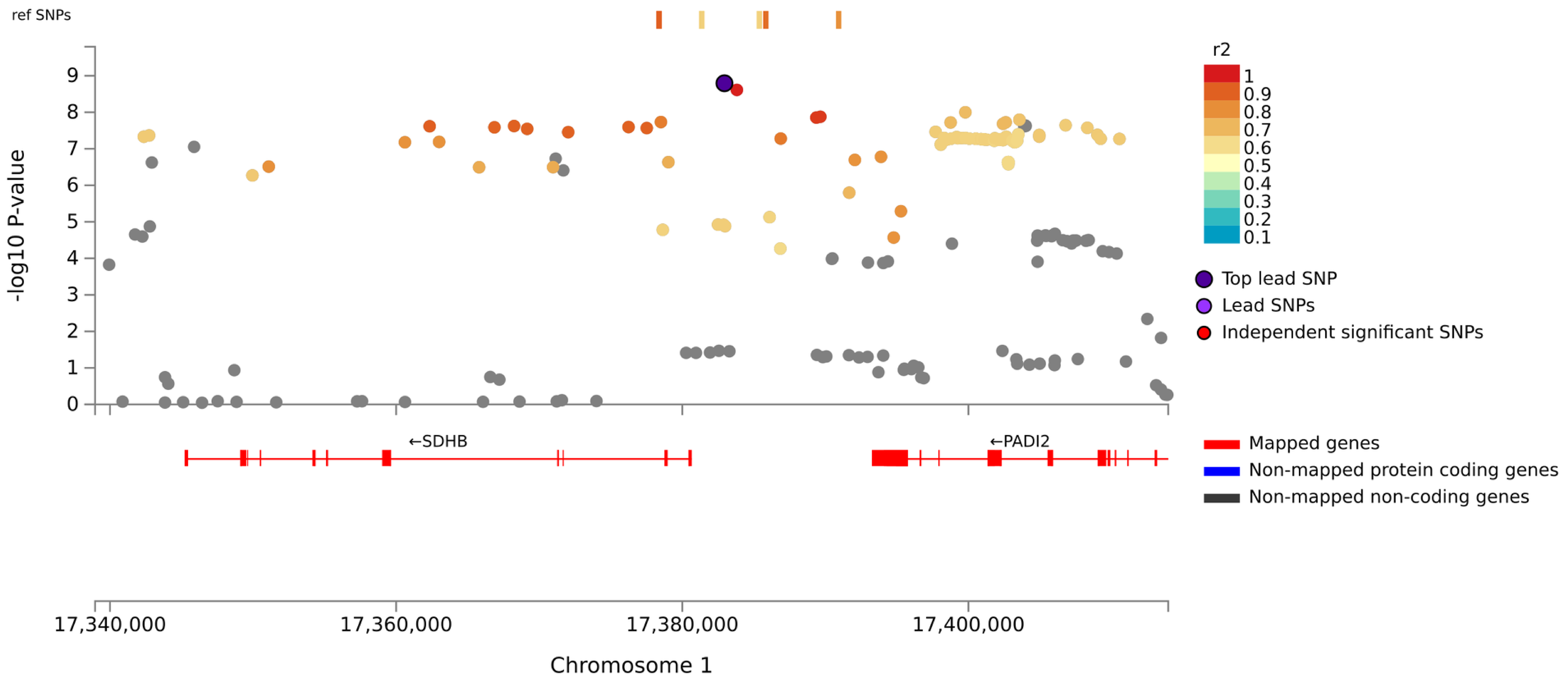

Locus 2, SOAT1, Anterior Body Area, rs3753526

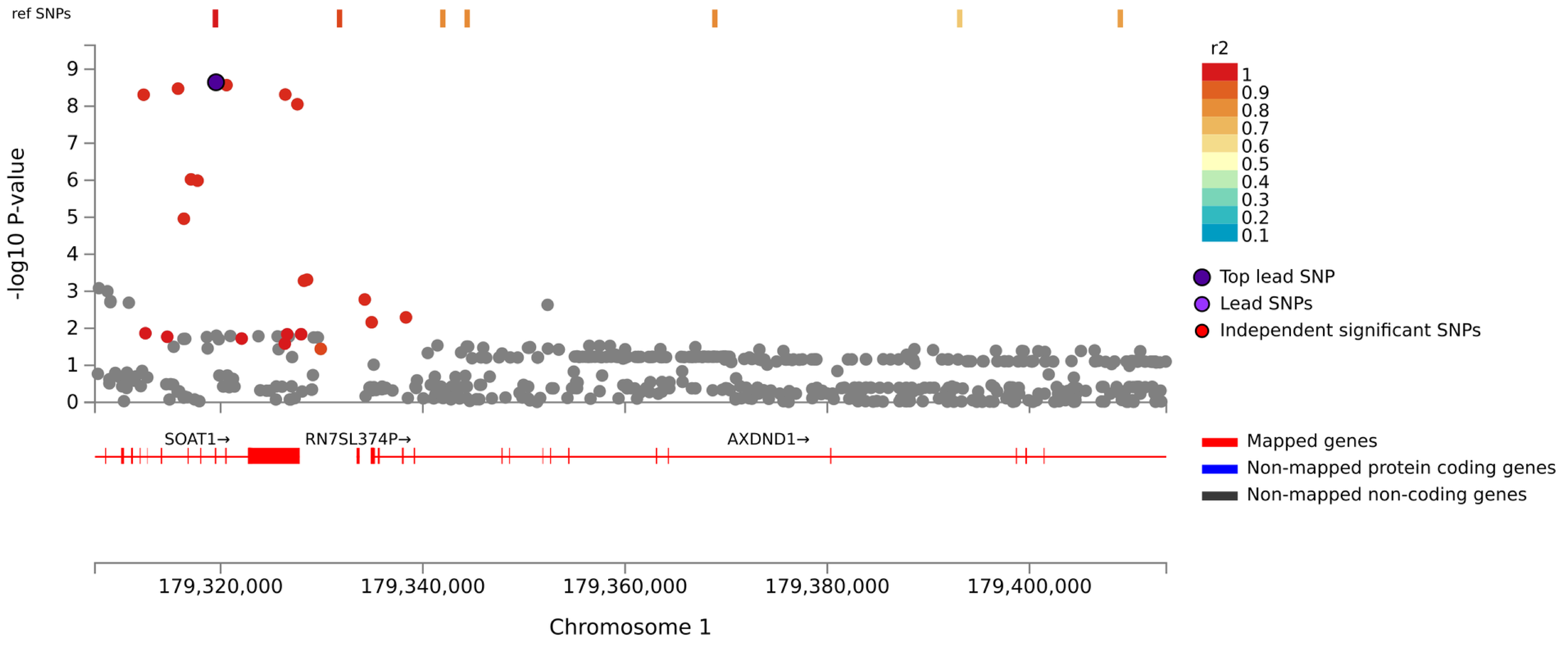

Locus 3, STRN, Anterior Body Area, rs7561572

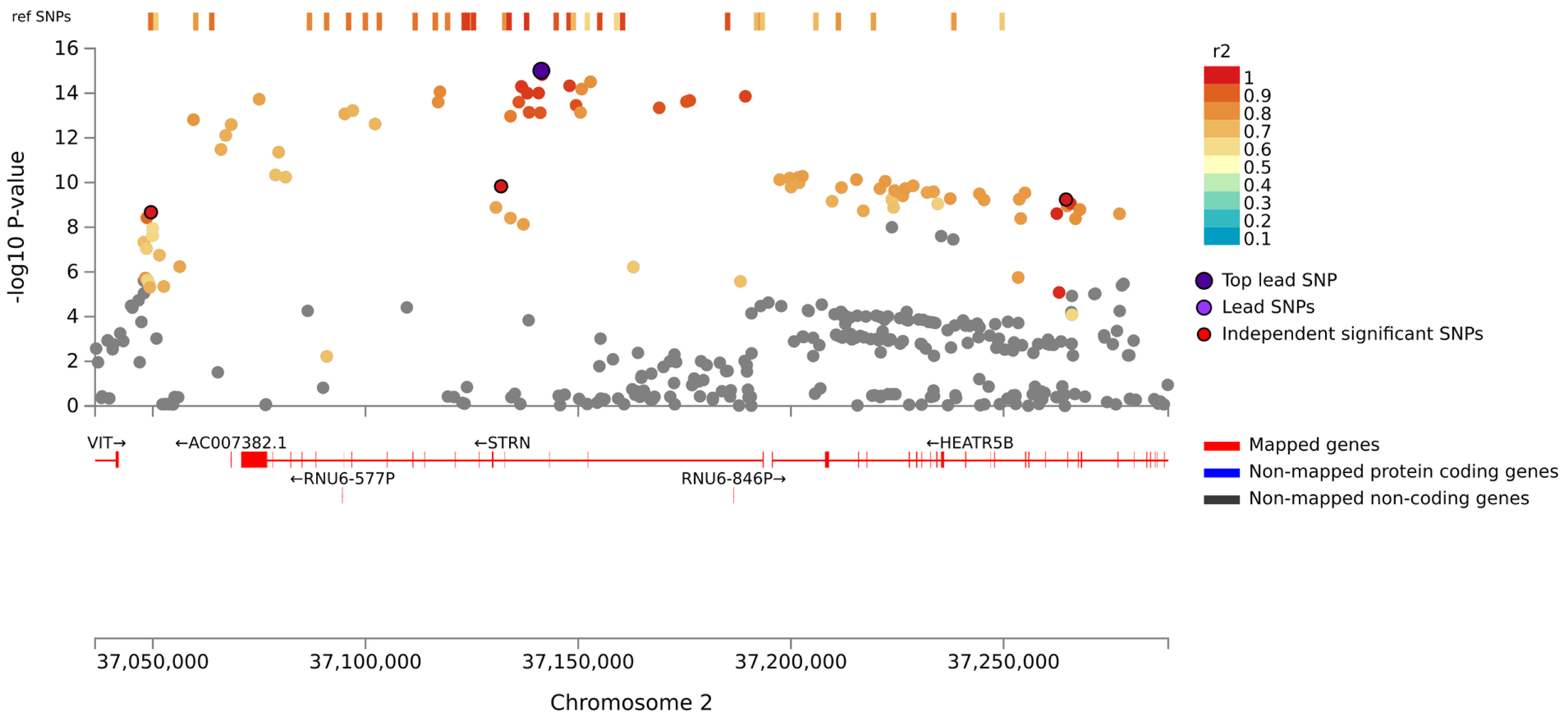

Locus 4, FIP1L1, Anterior Body Area, rs1466831

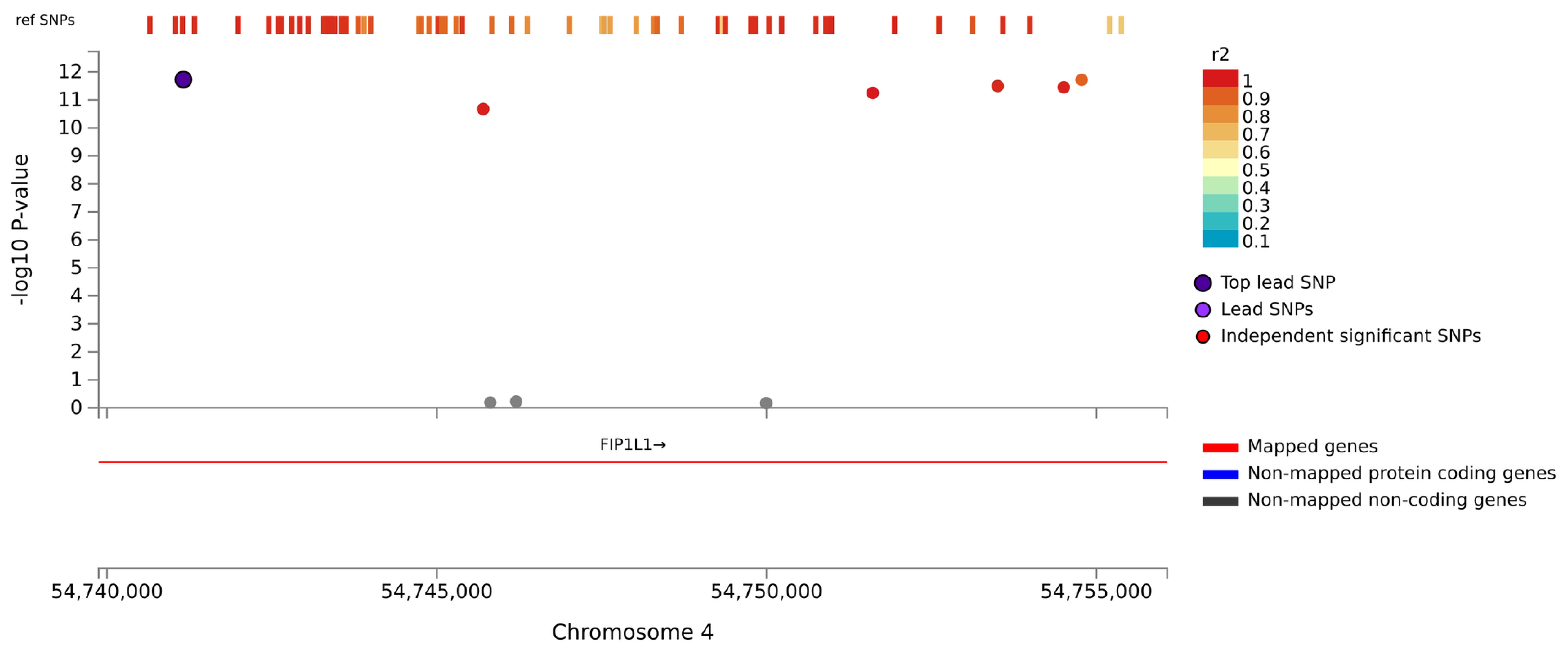

Locus 5, RNU6-727P, Anterior Body Area, rs71637276

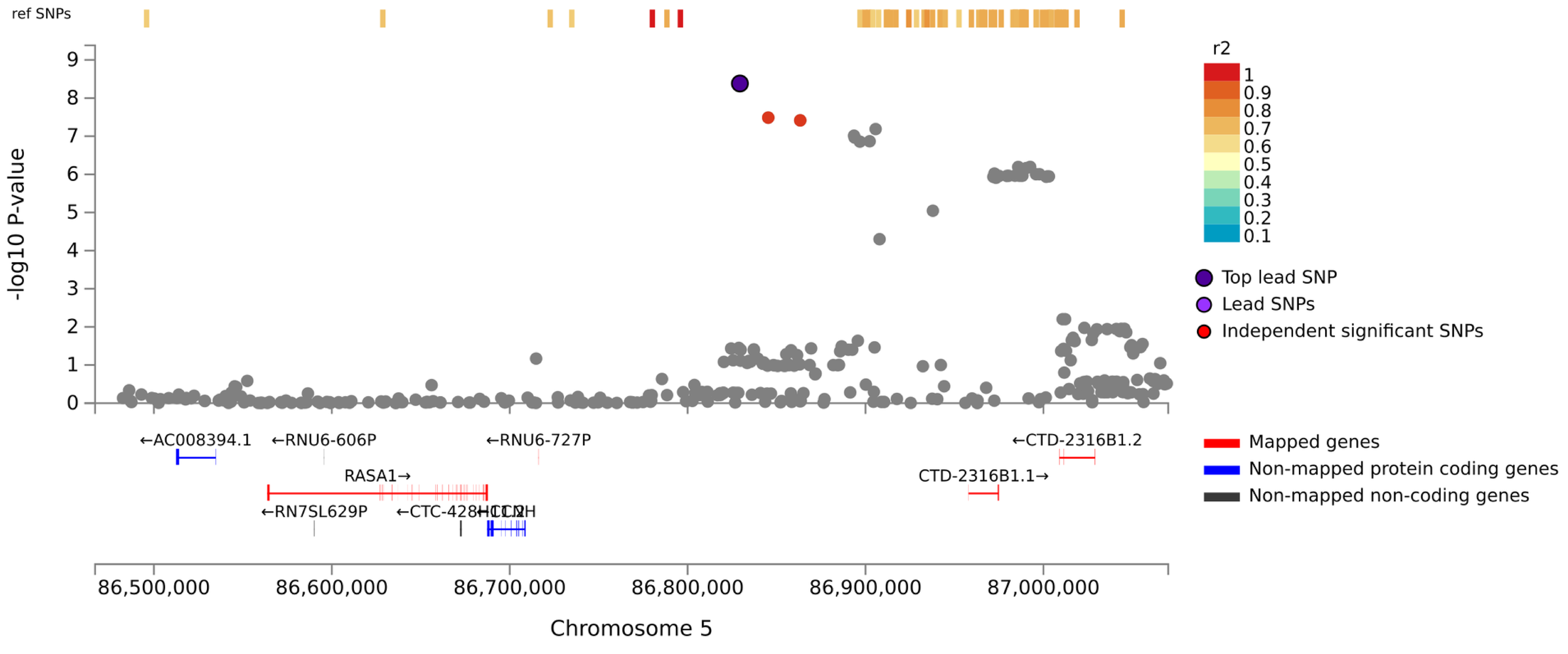

Locus 6, FOXO3, Anterior Body Area, rs1268163

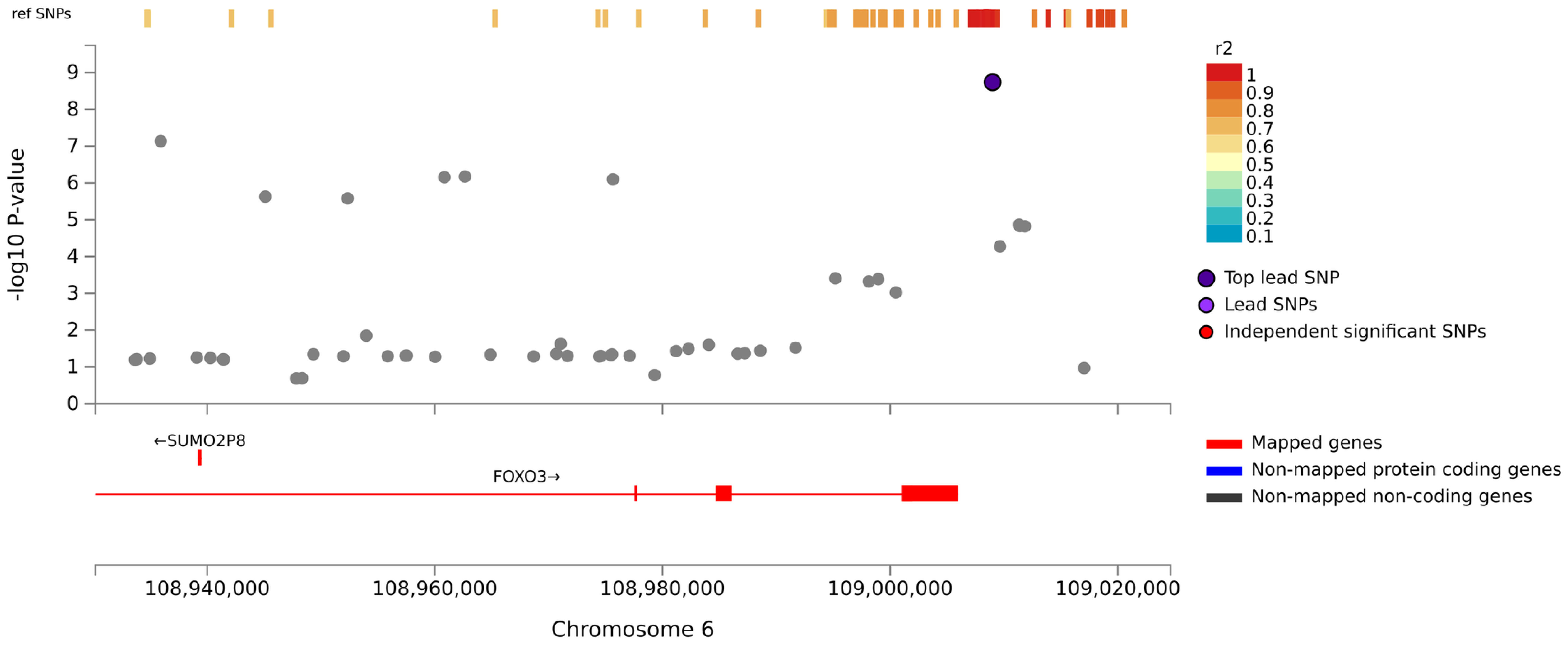

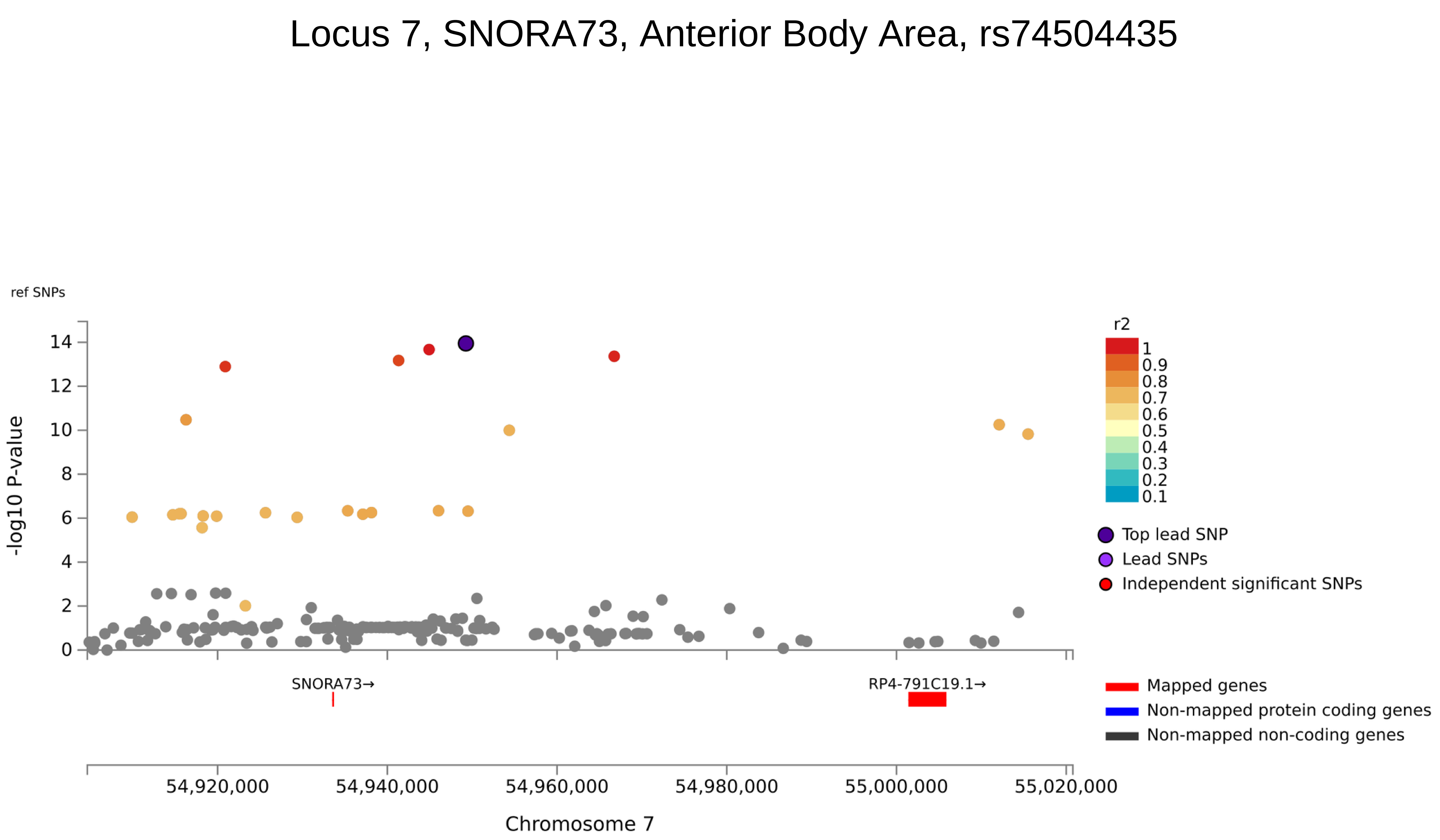

Locus 7, SNORA73, Anterior Body Area, rs74504435
ref SNPs
-log10 P-value
r2
Top lead SNP
Lead SNPs
Independent significant SNPs
SNORA73→
RP4-791C19.1→
Mapped genes
Non-mapped protein coding genes
Non-mapped non-coding genes
54,920,000
54,940,000
54,960,000
54,980,000
55,000,000
55,020,000
Chromosome 7

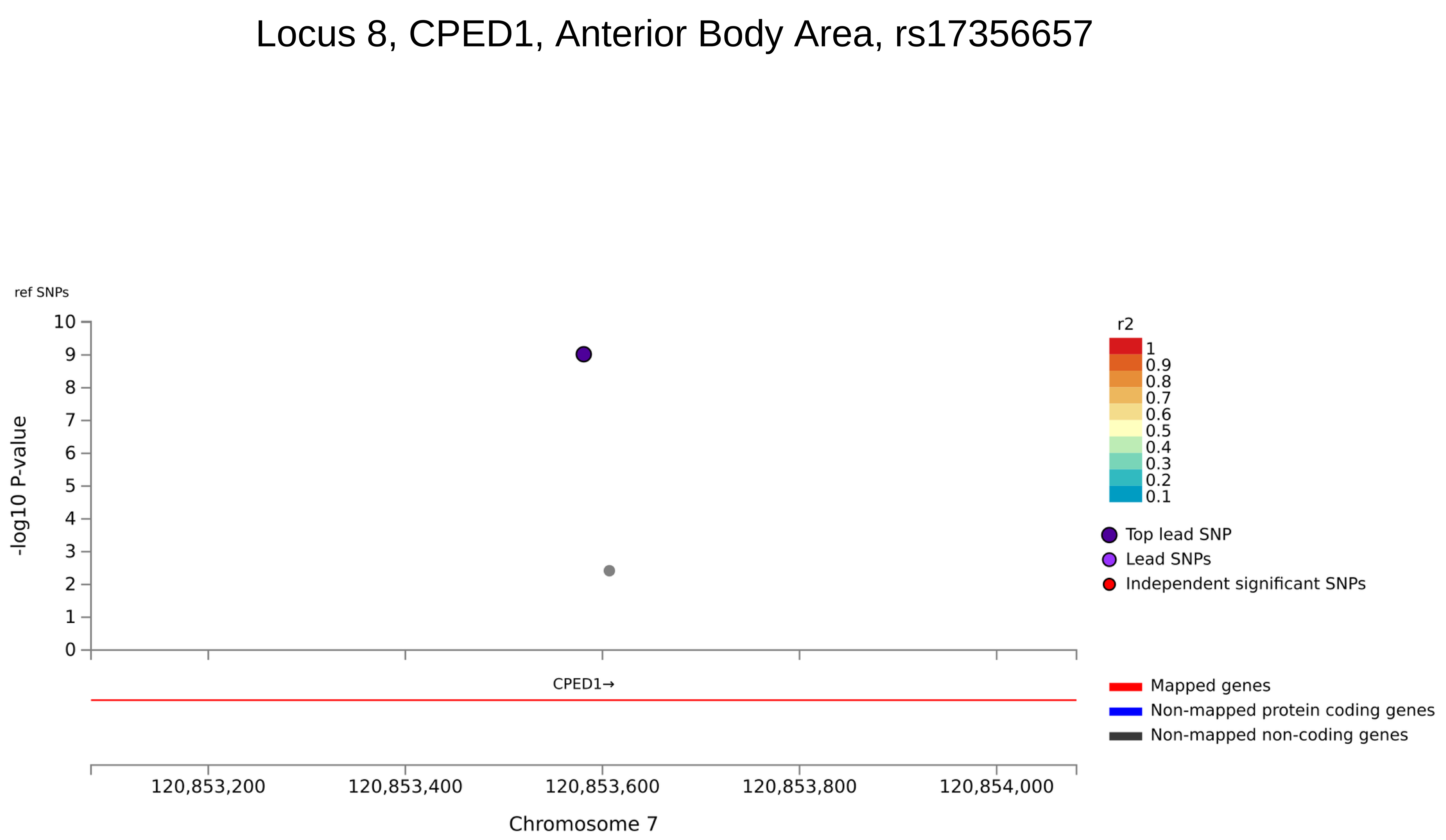

Locus 8, CPED1, Anterior Body Area, rs17356657
ref SNPs
-log10 P-value
r2
Top lead SNP
Lead SNPs
Independent significant SNPs
CPED1→
Mapped genes
Non-mapped protein coding genes
Non-mapped non-coding genes
120,853,200
120,853,400
120,853,600
120,853,800
120,854,000
Chromosome 7

Locus 9, PLEC, Anterior Body Area, rs55646585

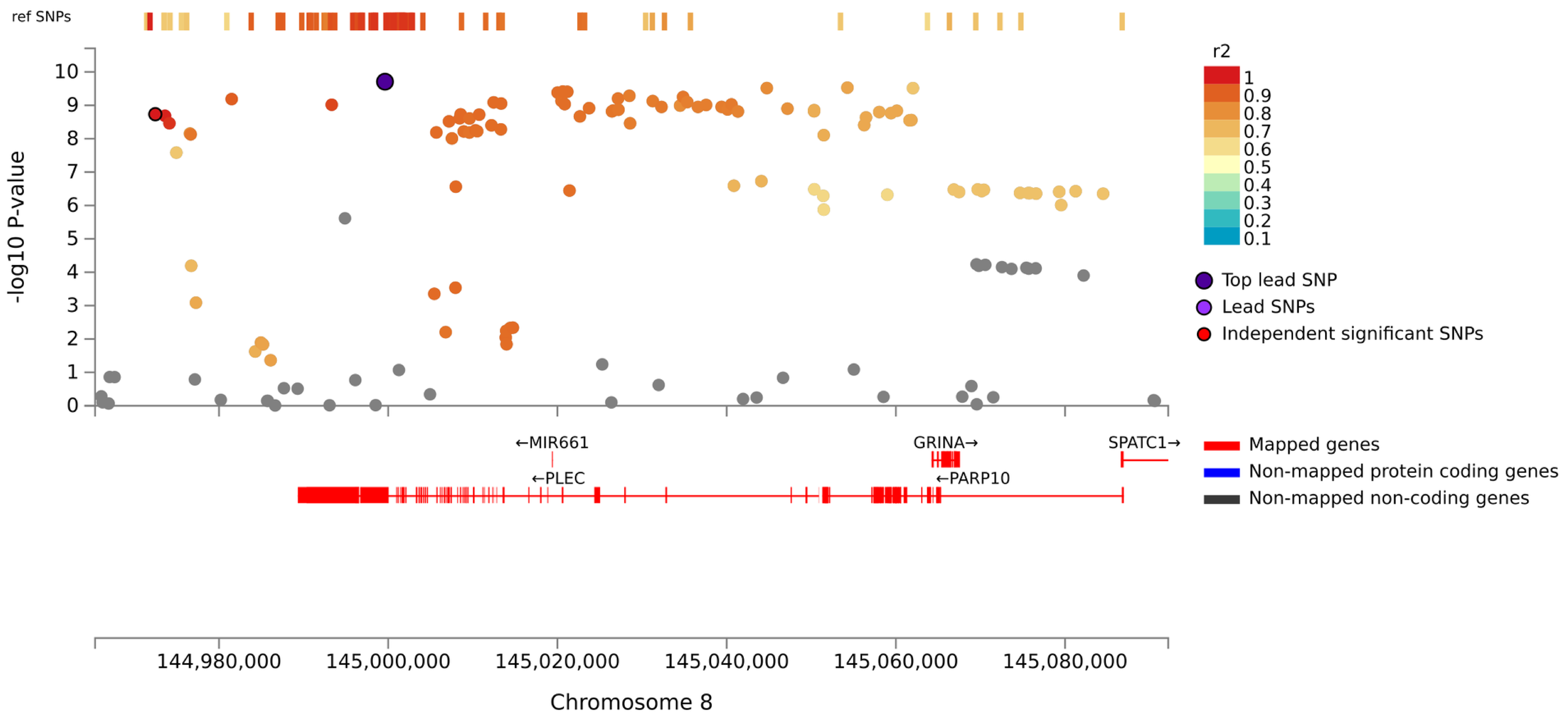

Locus 10, FAM107B, Anterior Body Area, rs10906729

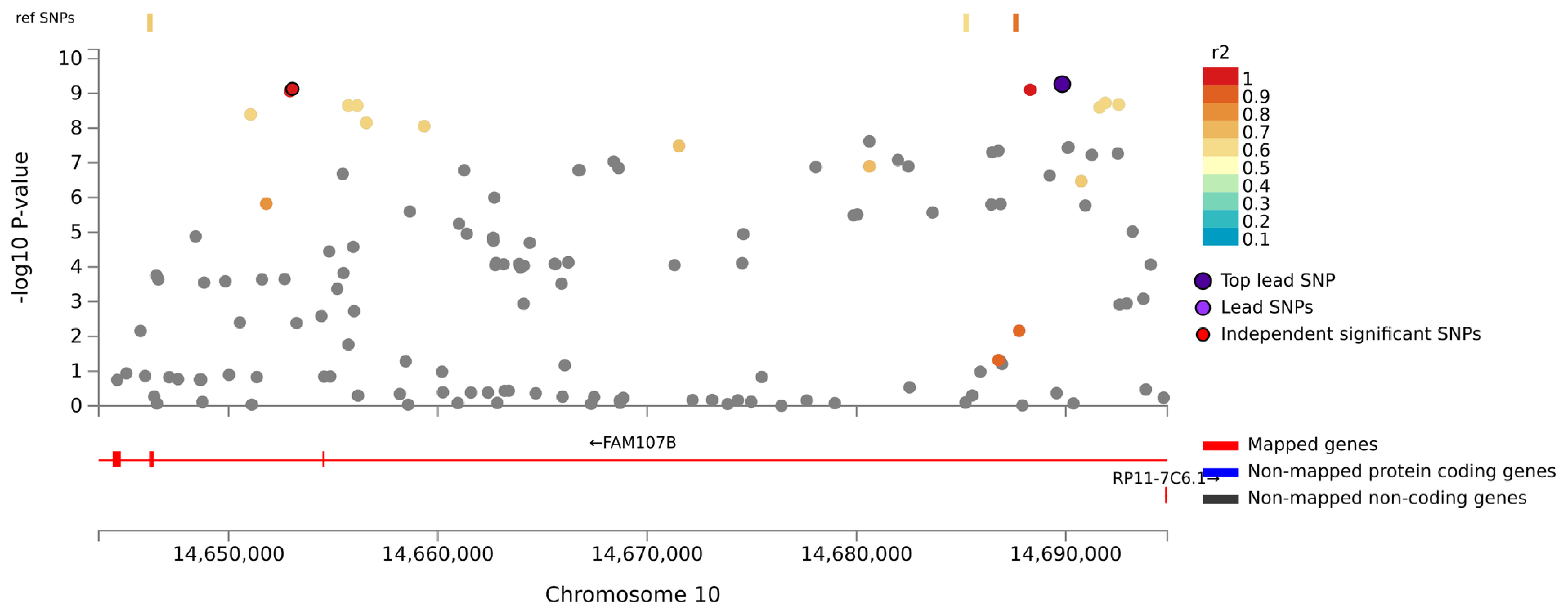

Locus 11, KIAA1598, Anterior Body Area, rs1122688

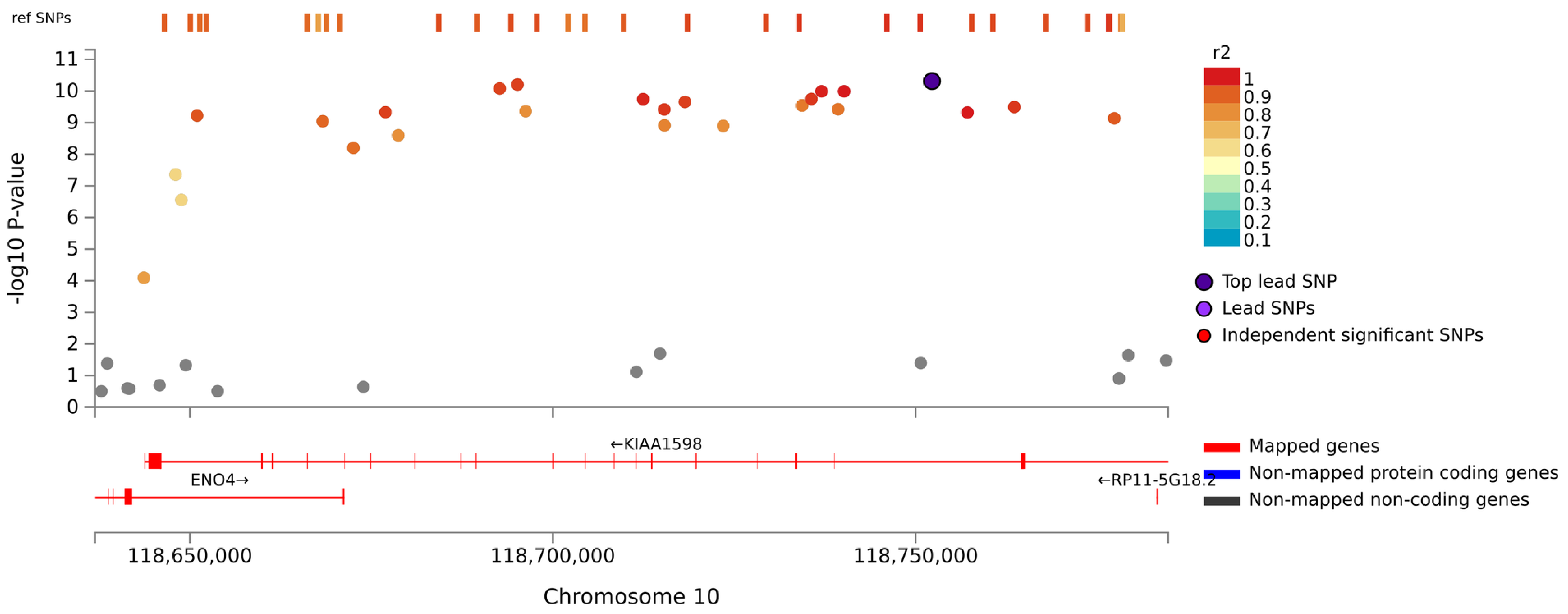

Locus 12, DUSP8, Anterior Body Area, rs7127282

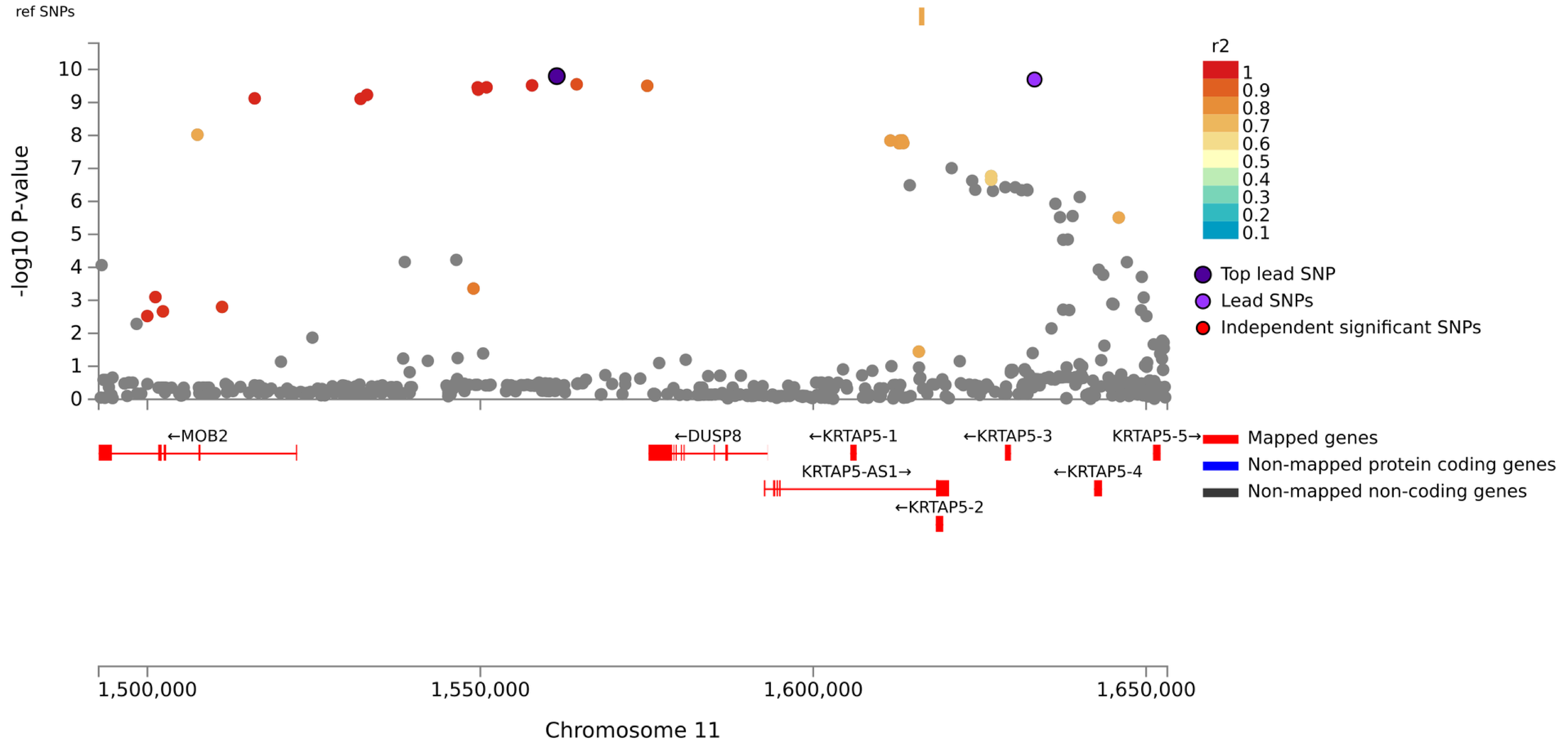

Locus 13, RP1-34H18.1, Anterior Body Area, rs696451

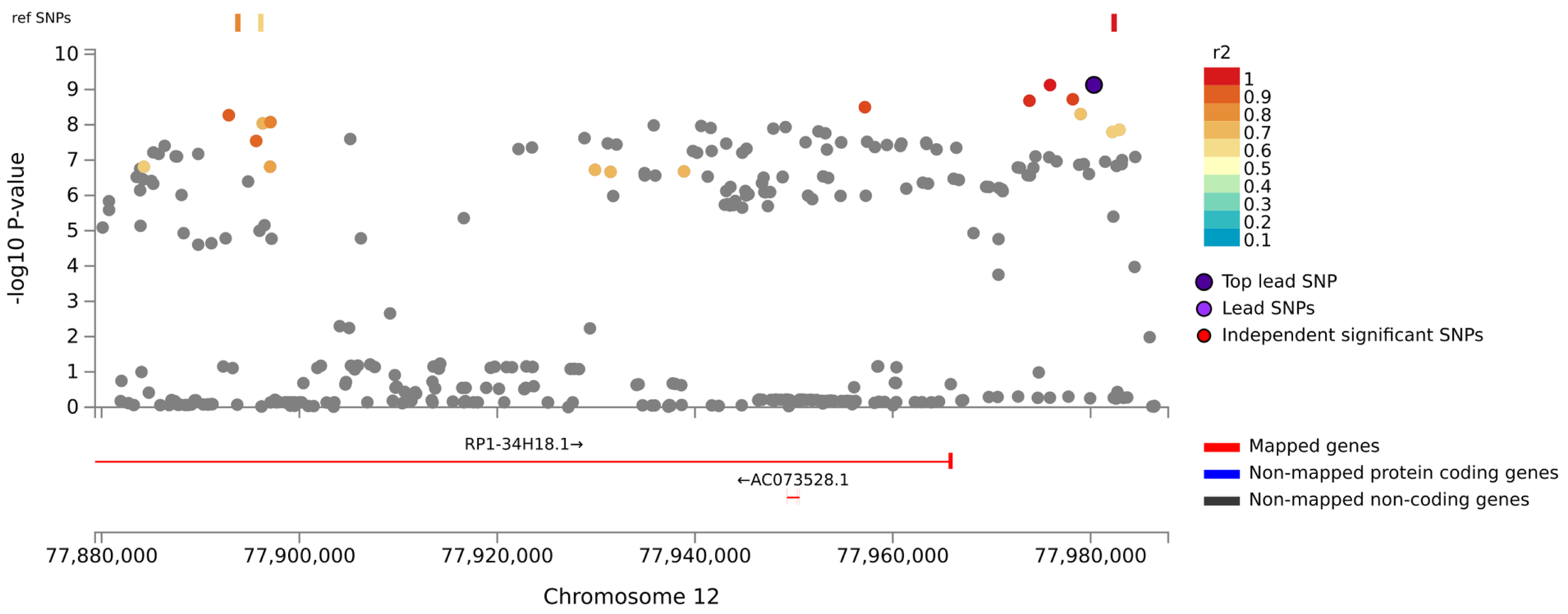

Locus 14, CABYR, Anterior Body Area, rs752797

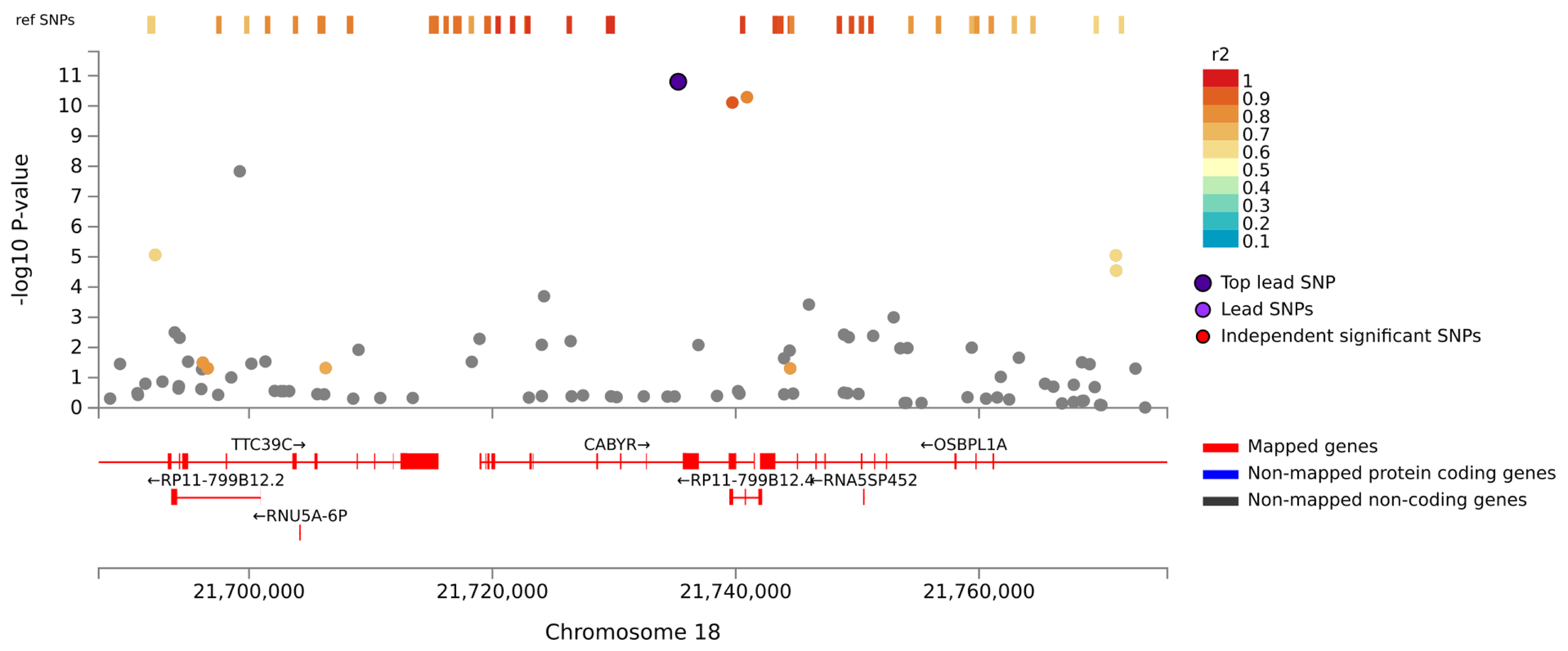

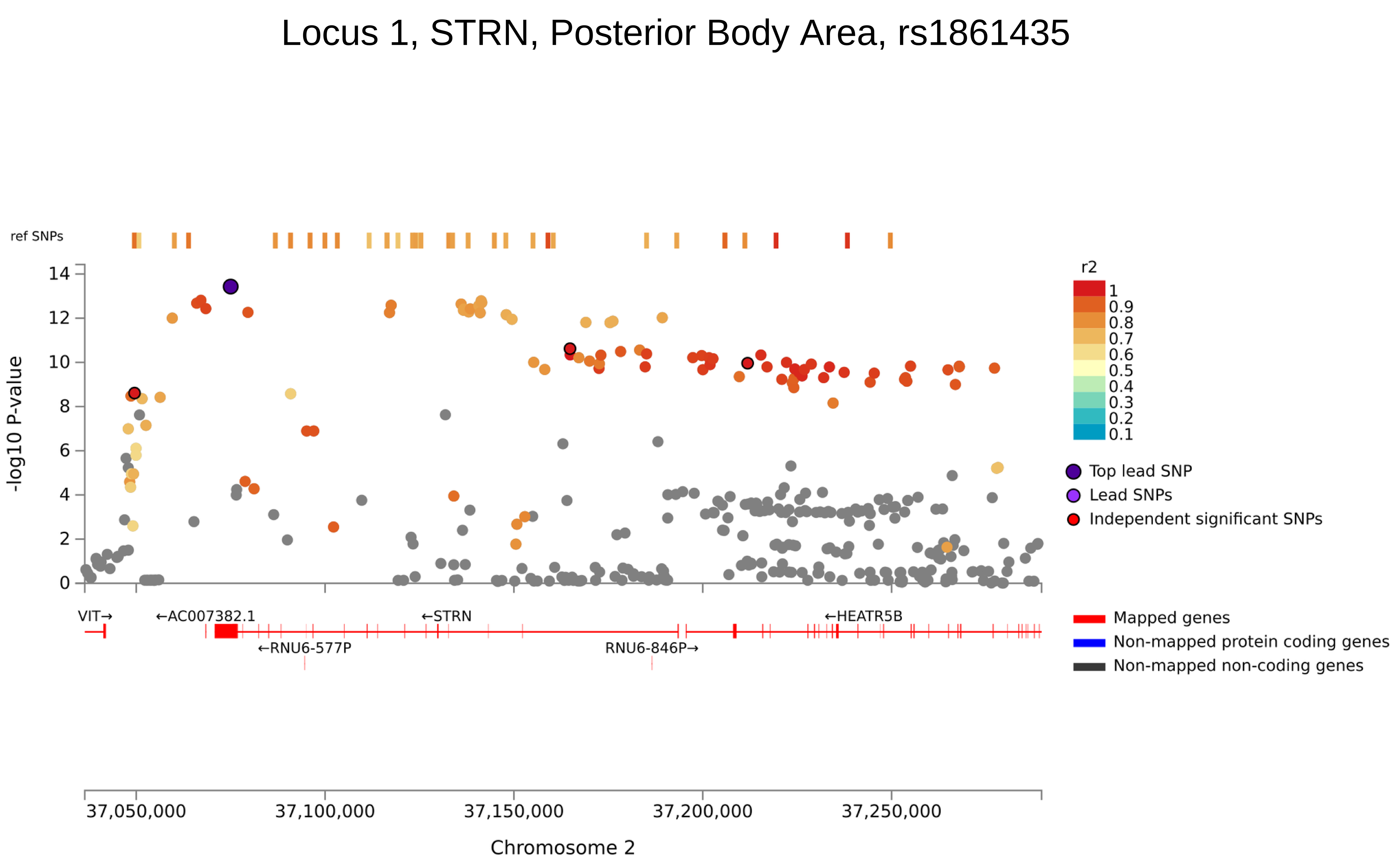
Locus 1, STRN, Posterior Body Area, rs1861435
ref SNPs
-log10 P-value
r2
Top lead SNP
Lead SNPs
Independent significant SNPs
VIT→
←AC007382.1
←STRN
←HEATR5B
←RNU6-577P
RNU6-846P→
Mapped genes
Non-mapped protein coding genes
Non-mapped non-coding genes
37,050,000
37,100,000
37,150,000
37,200,000
37,250,000
Chromosome 2

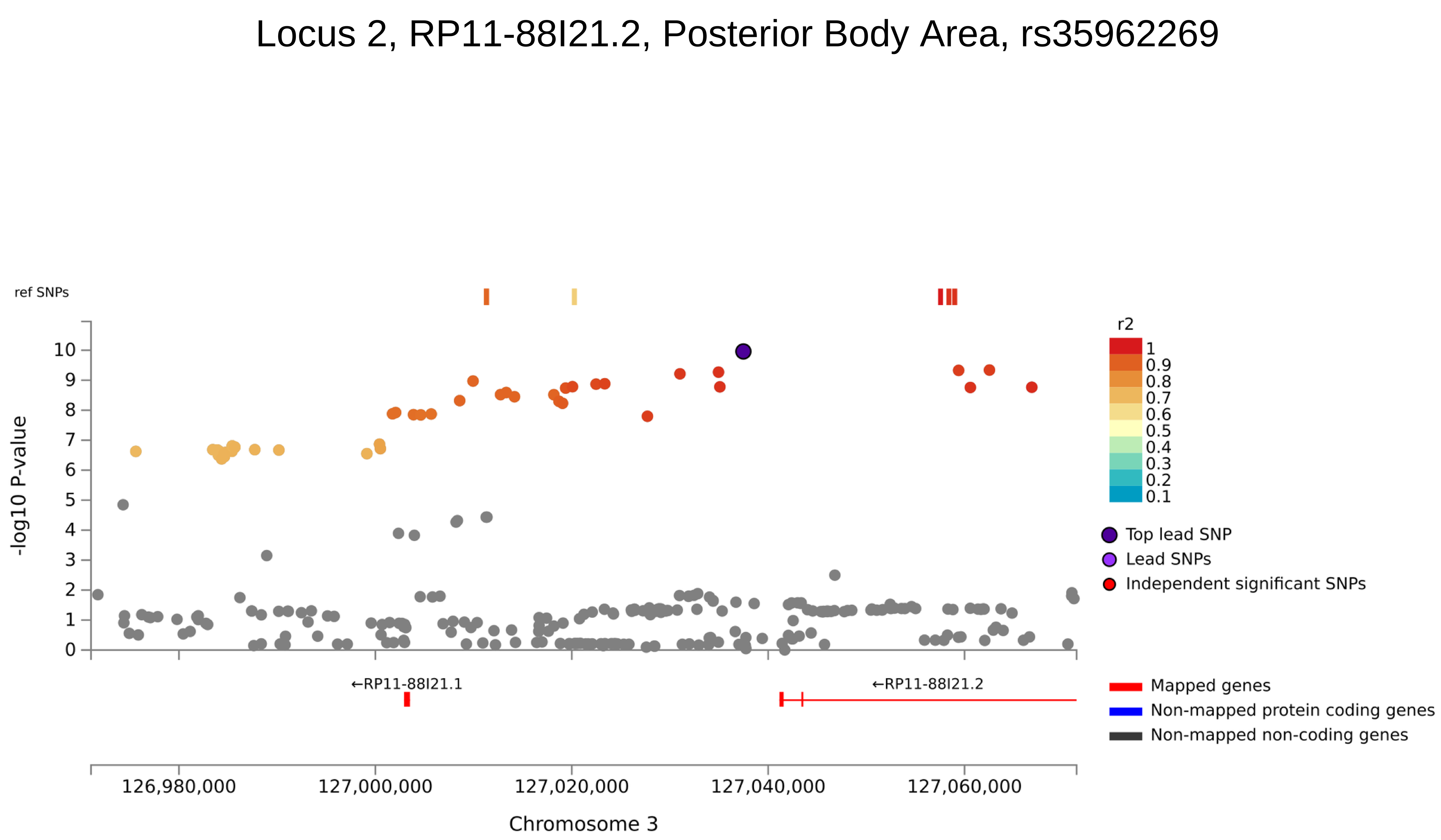
Locus 2, RP11-88I21.2, Posterior Body Area, rs35962269
ref SNPs
-log10 P-value
r2
Top lead SNP
Lead SNPs
Independent significant SNPs
←RP11-88I21.1
←RP11-88I21.2
Mapped genes
Non-mapped protein coding genes
Non-mapped non-coding genes
126,980,000
127,000,000
127,020,000
127,040,000
127,060,000
Chromosome 3

Locus 3, FIP1L1, Posterior Body Area, rs6835429

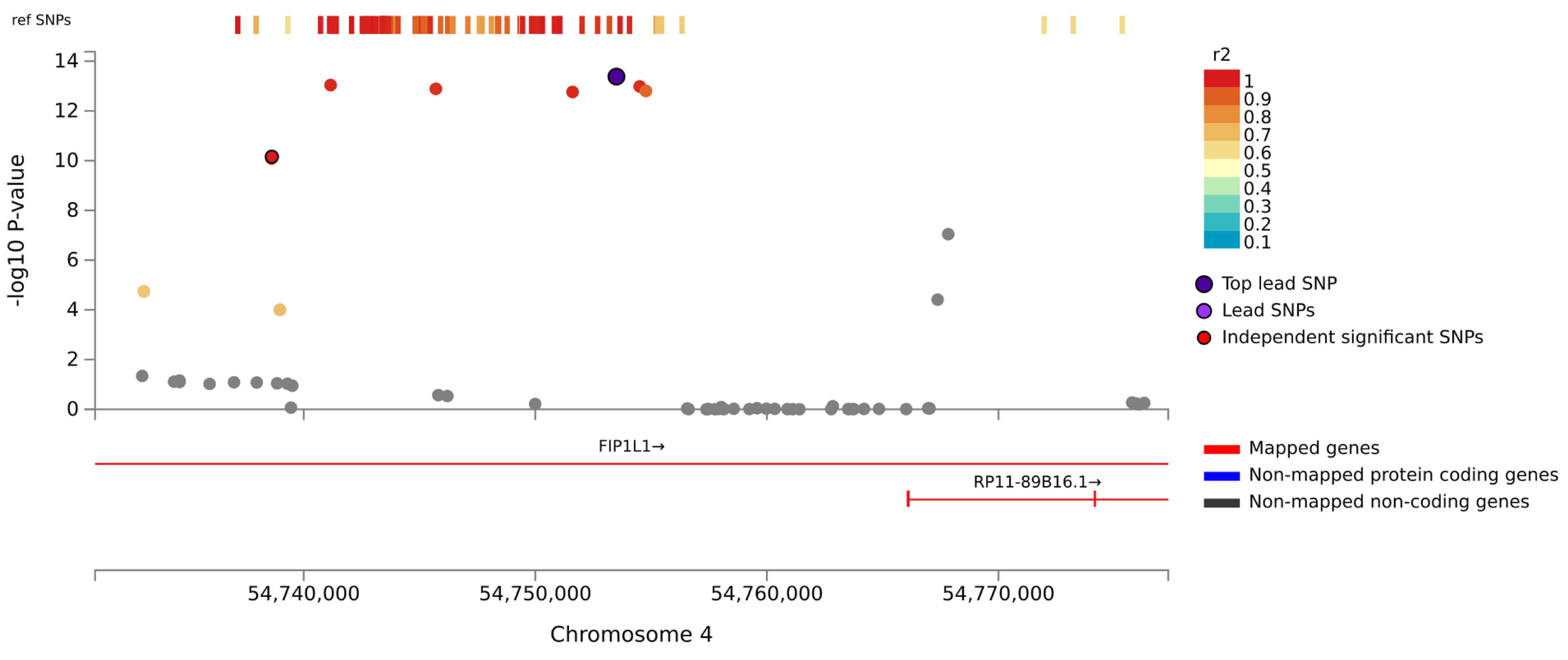

Locus 4, HBEGF, Posterior Body Area, rs4150214

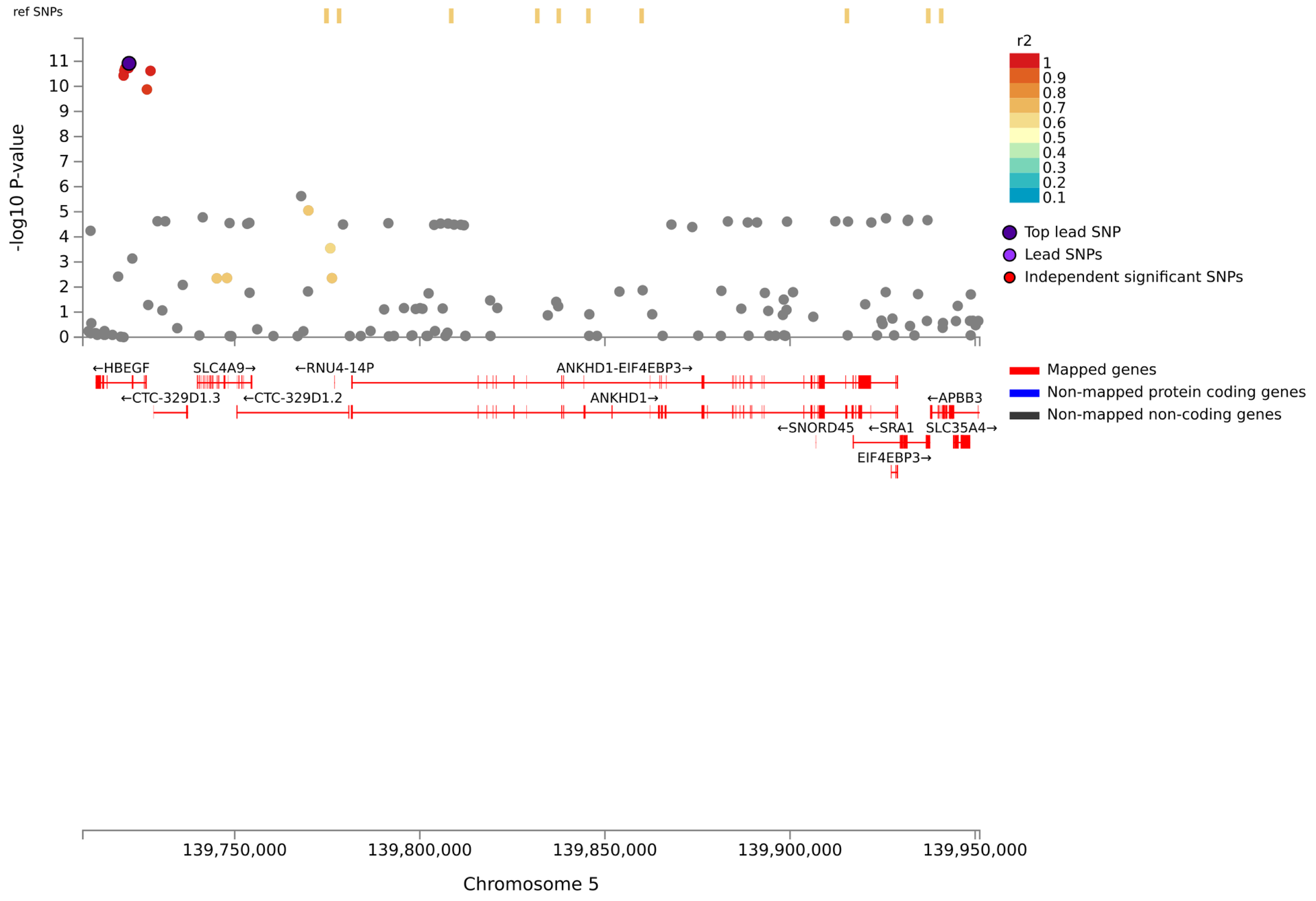

Locus 5, FOXO3, Posterior Body Area, rs1268163

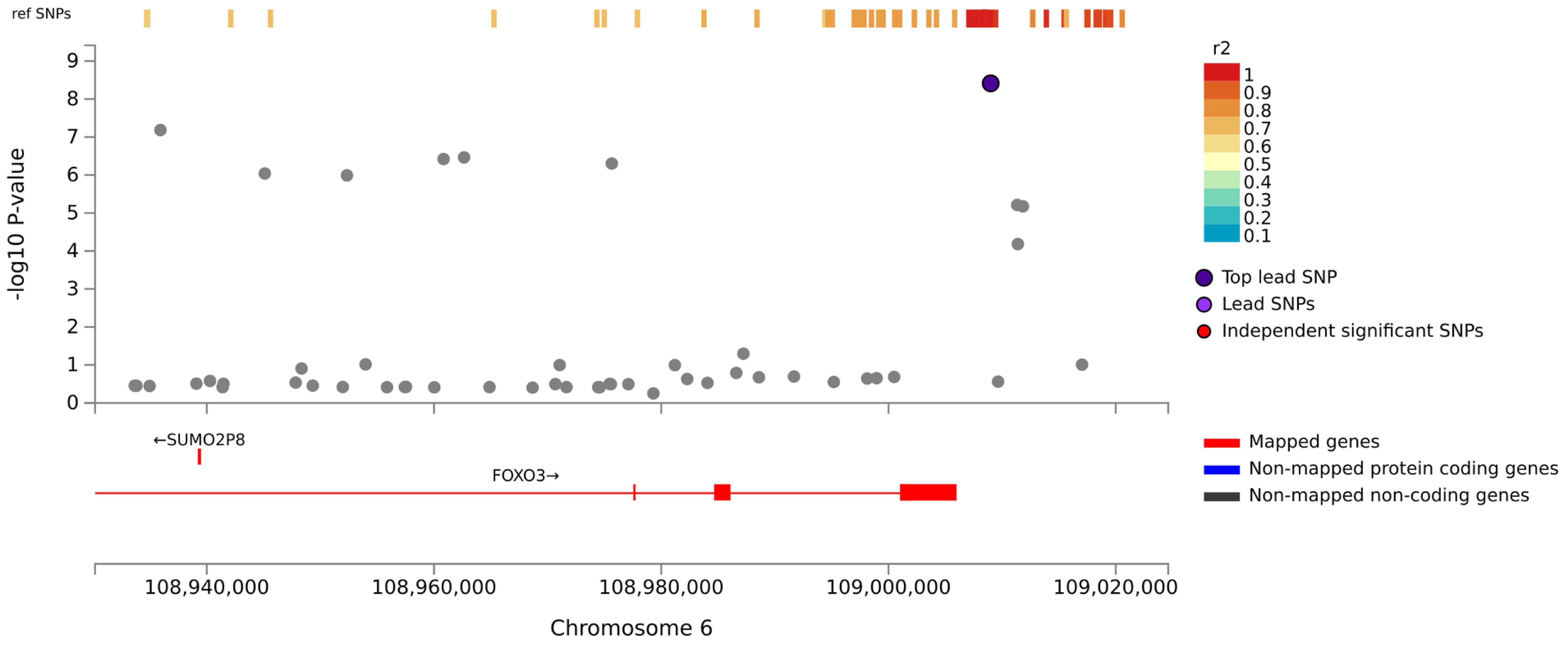

Locus 6, RP1-34H18.1, Posterior Body Area, rs4761385

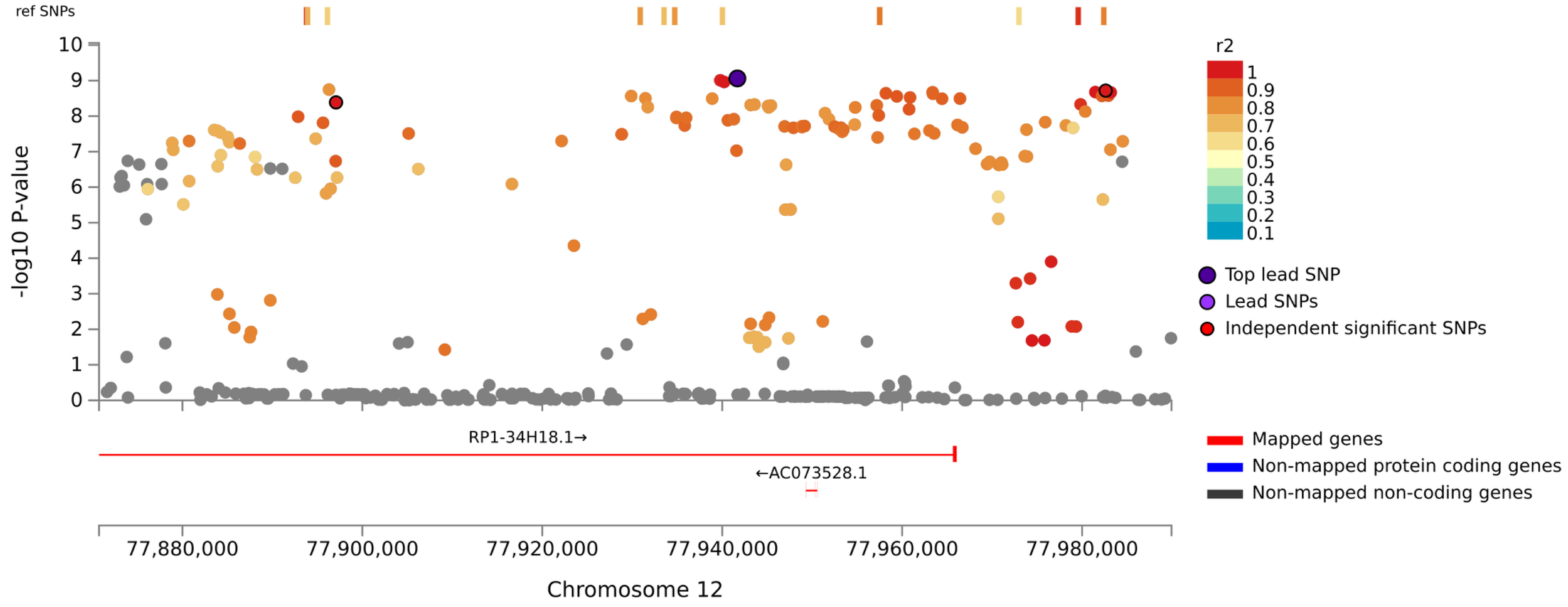

Locus 7, TTC39C, Posterior Body Area, rs12967609

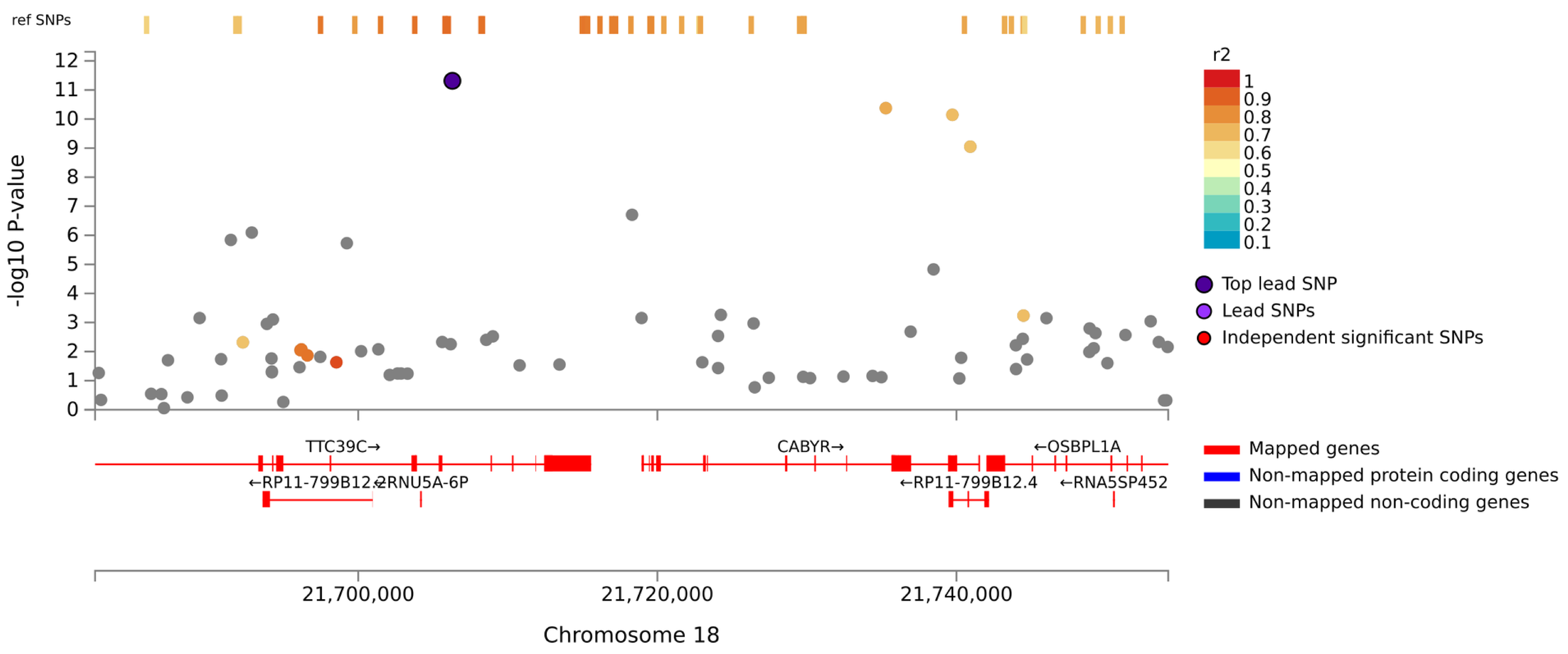

Locus 1, RP1-37C10.3, Isthmus Area, rs6682671

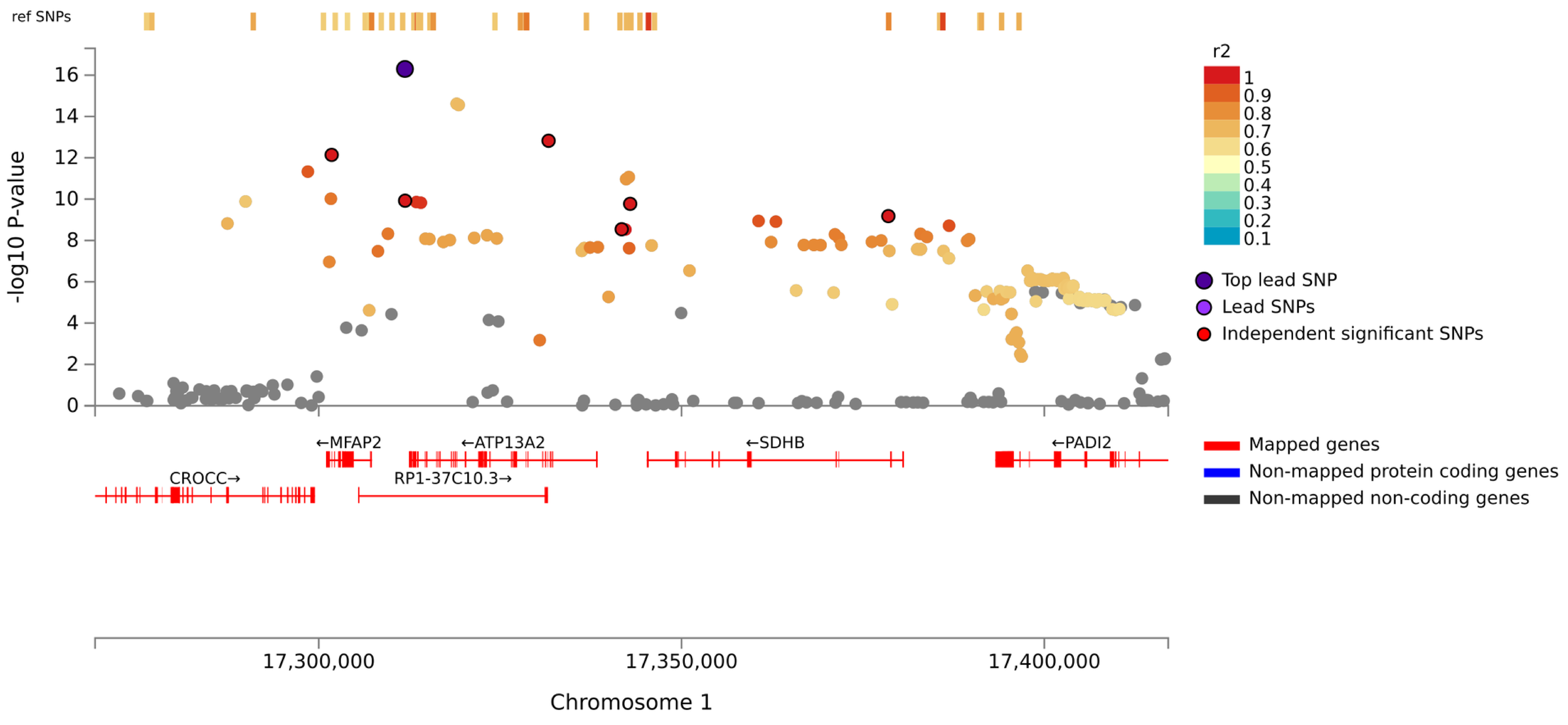

Locus 2, STRN, Isthmus Area, rs10193295

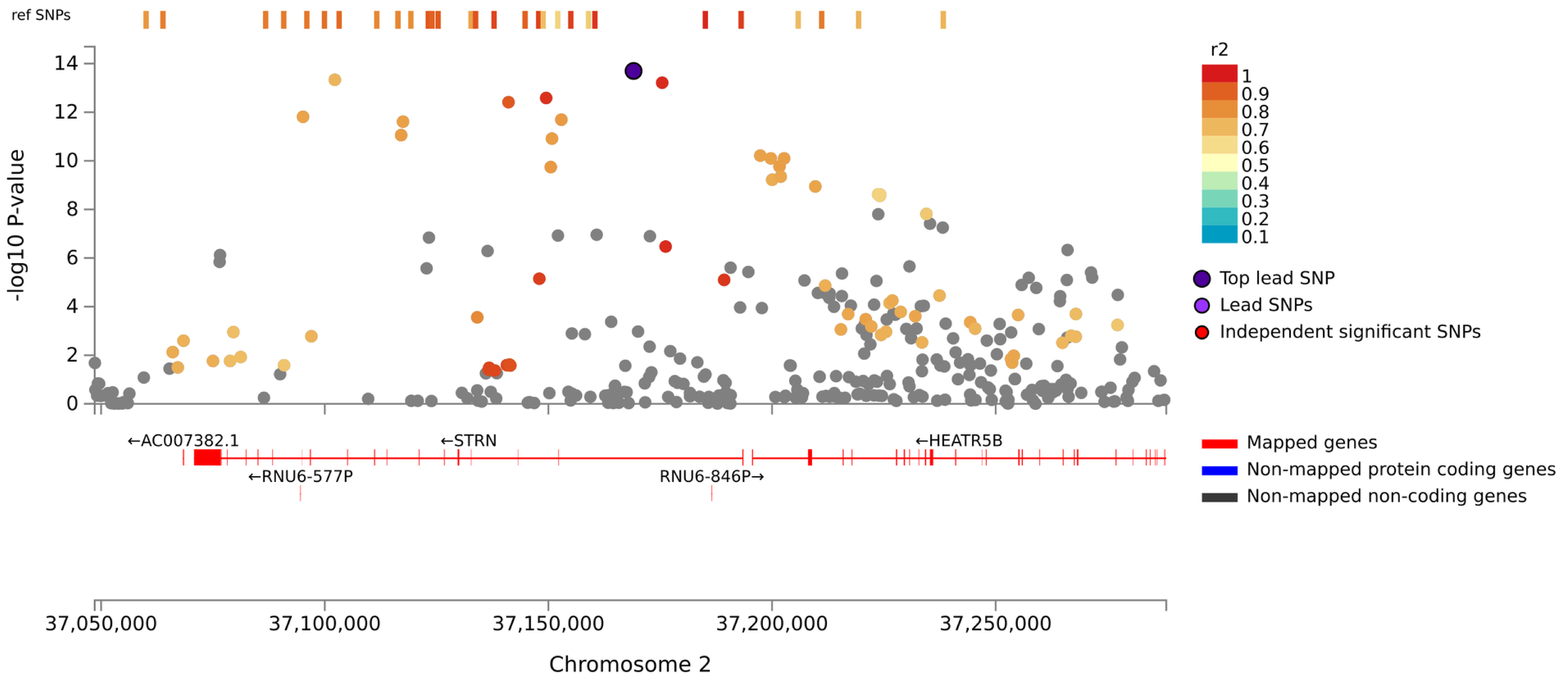

Locus 3, CCDC75P1, Isthmus Area, rs9860128

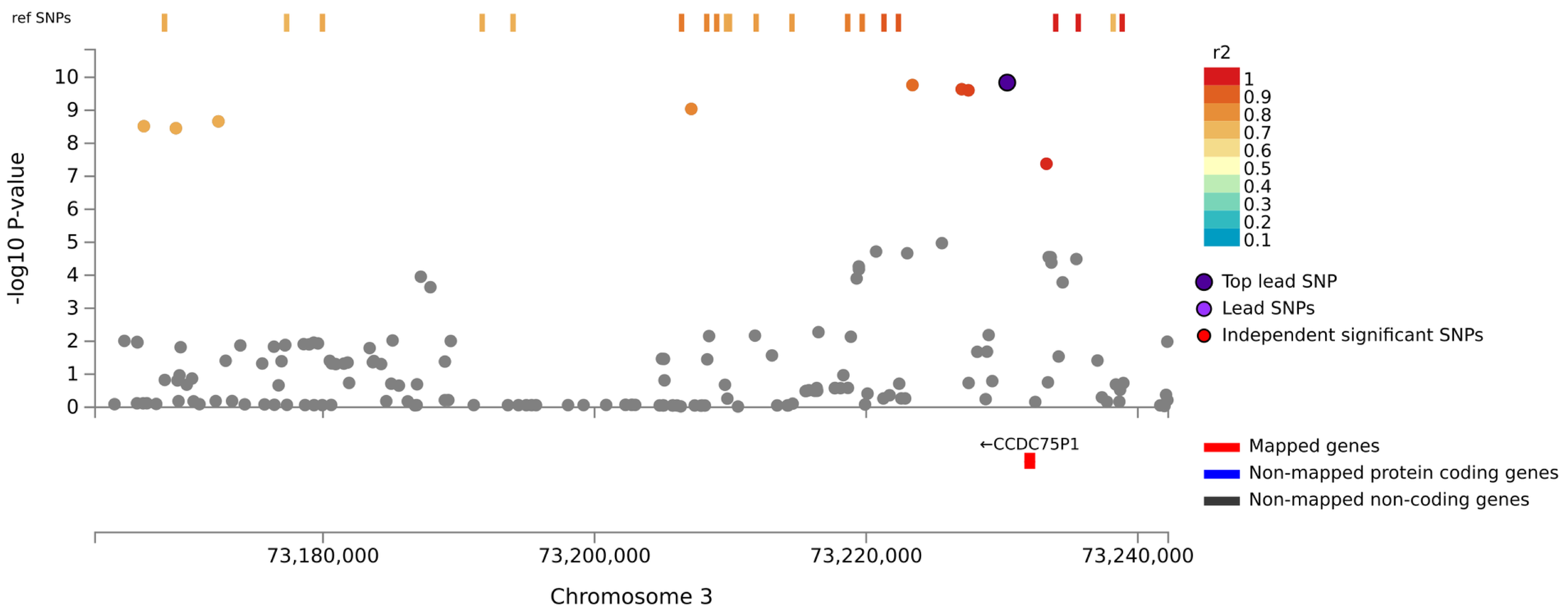

Locus 4, IQCJ-SCHIP1:IQCJ, Isthmus Area, rs11717303

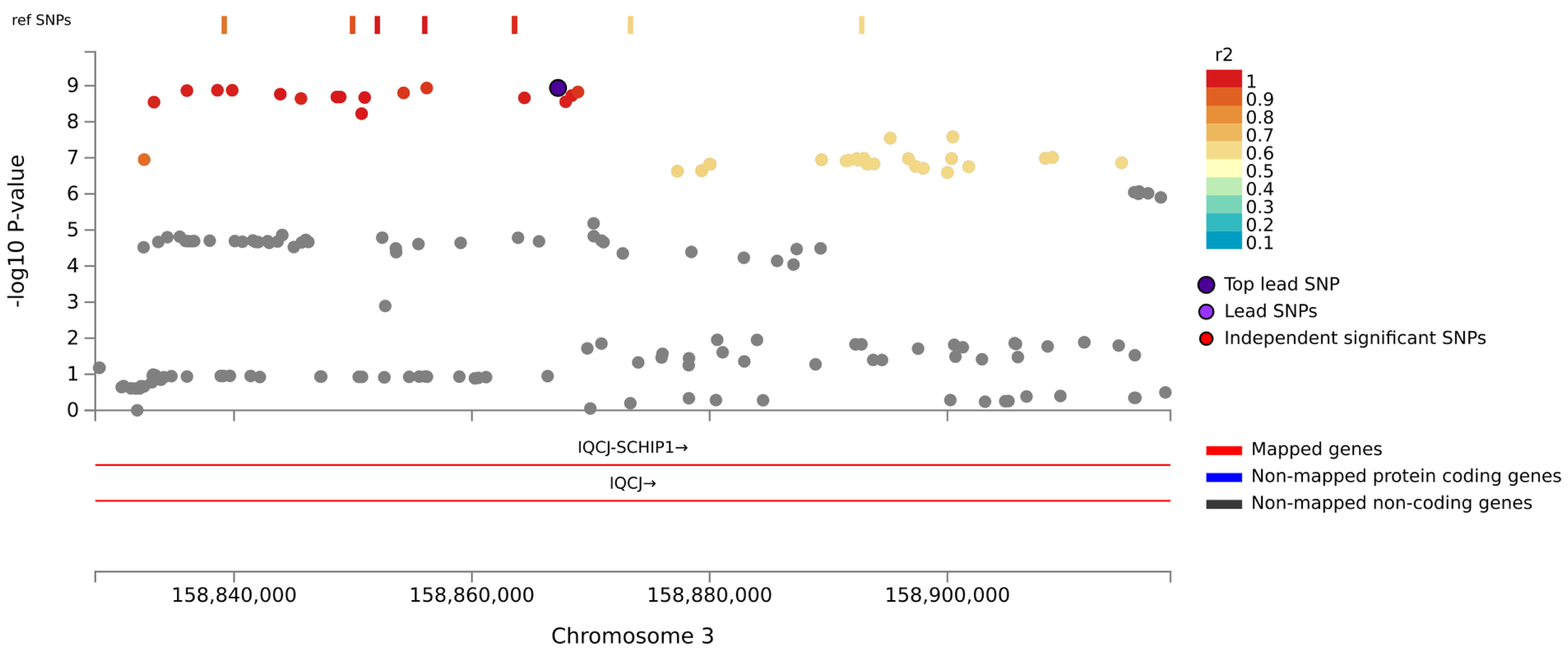

Locus 5, TNIK, Isthmus Area, rs2035913

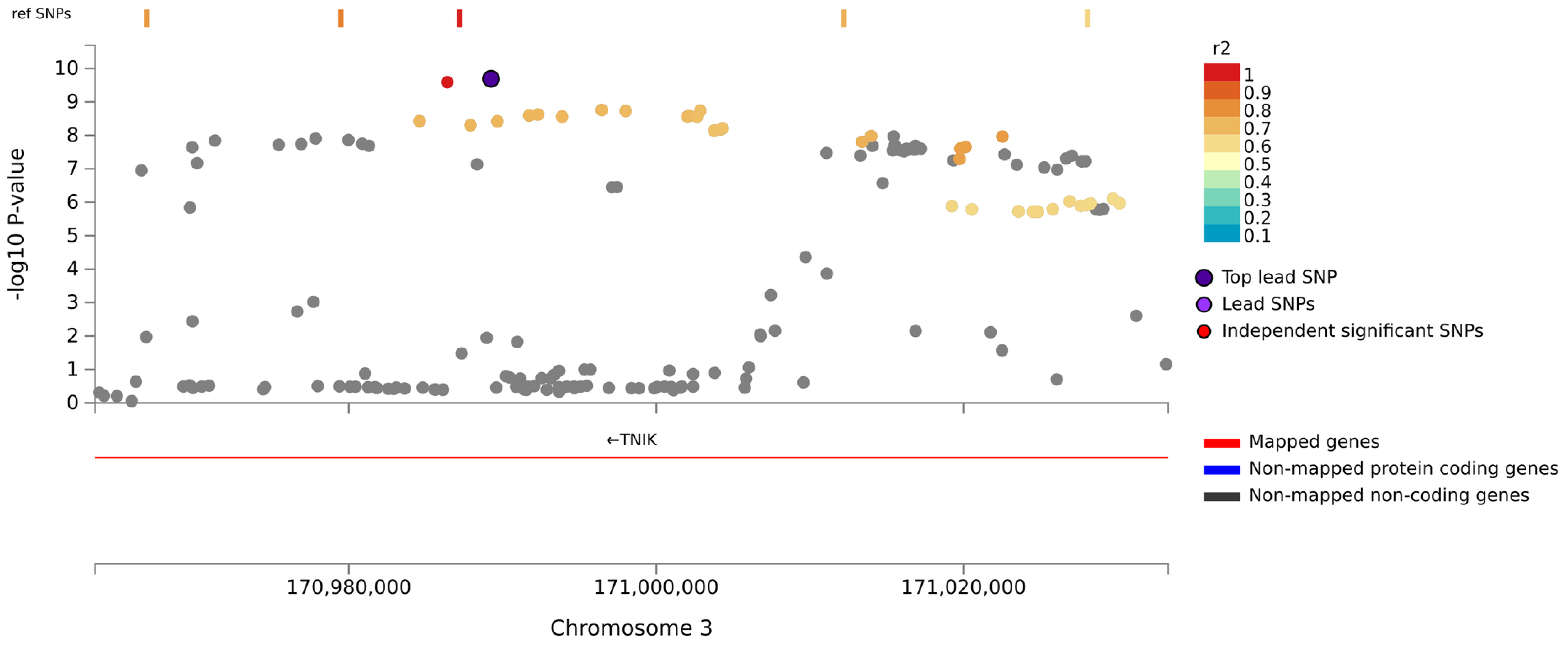

Locus 6, ADD1, Isthmus Area, rs12645803

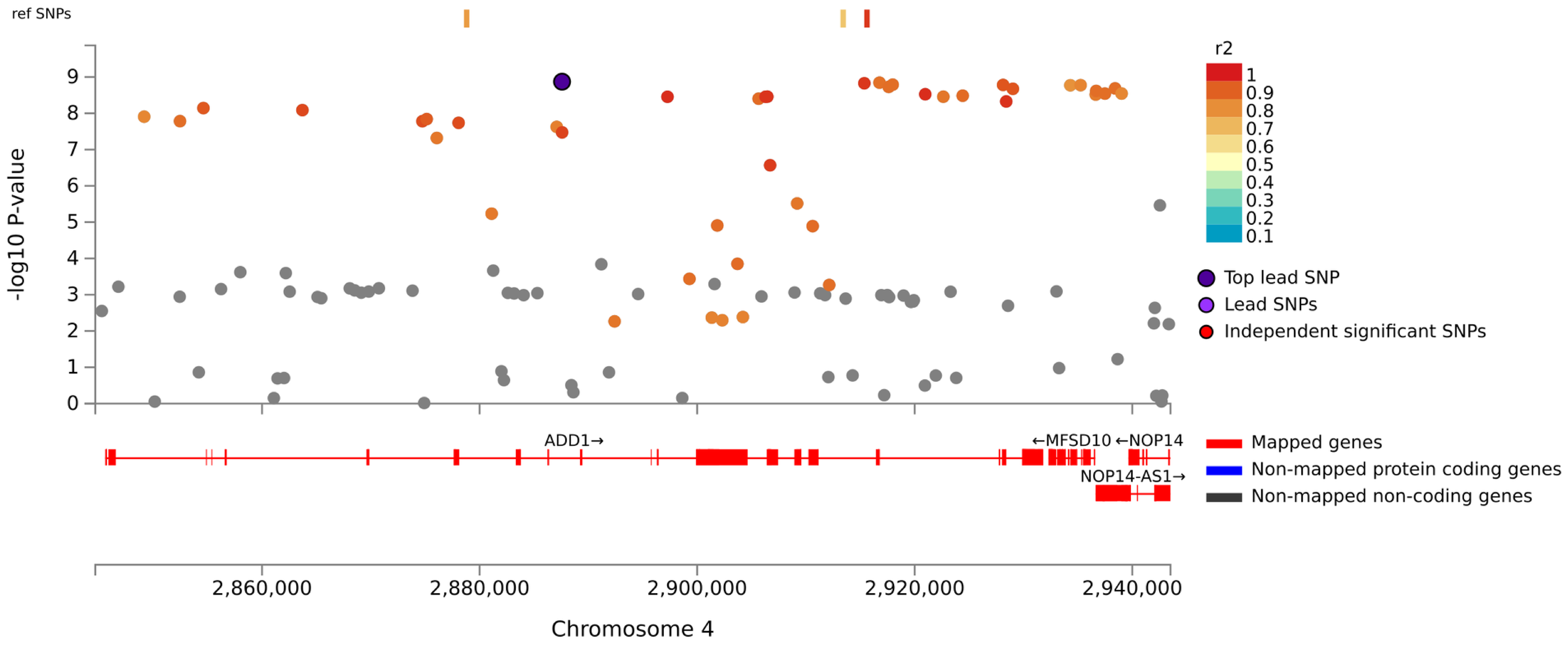

Locus 7, TBC1D14, Isthmus Area, rs529090333

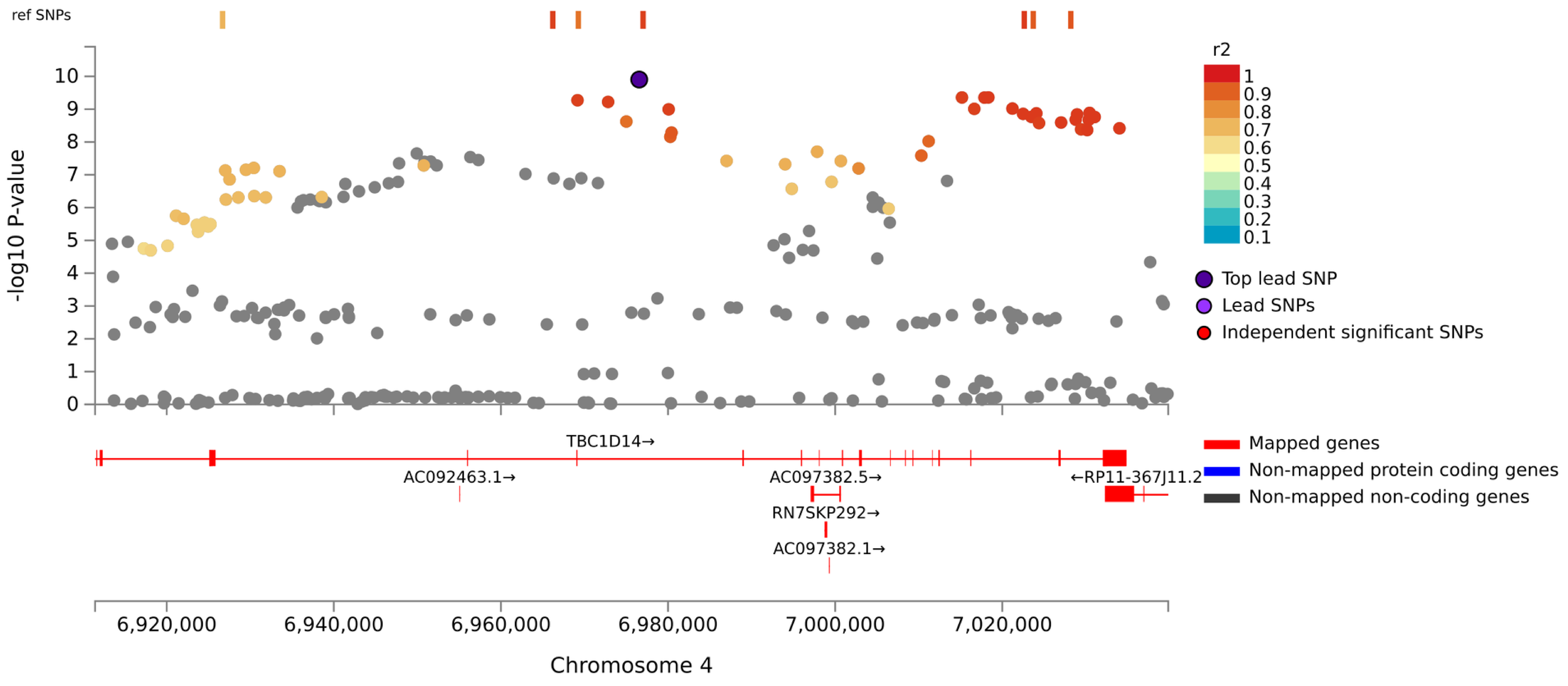

Locus 8, FIP1L1:RP11-89B16.1, Isthmus Area, rs62297576

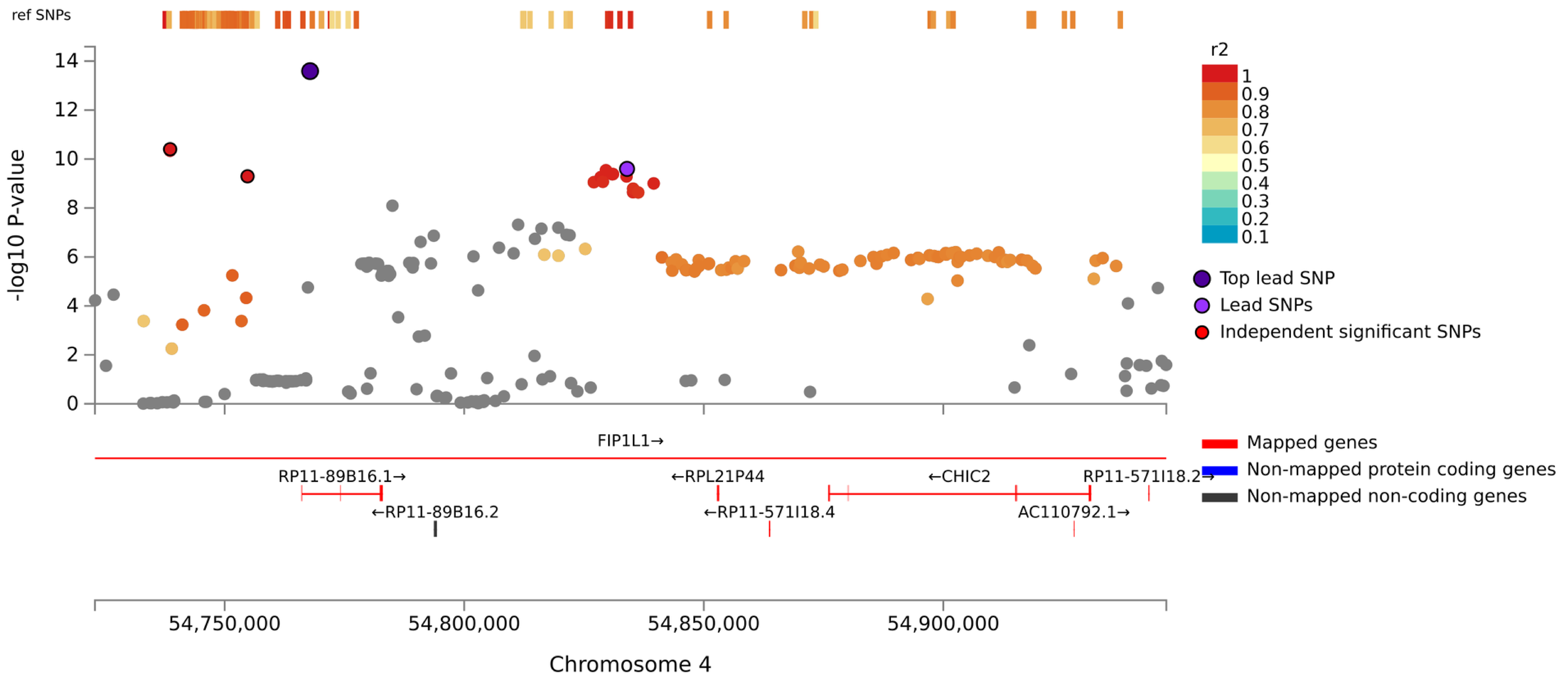

Locus 9, CTB-118N6.2, Isthmus Area, rs3806915

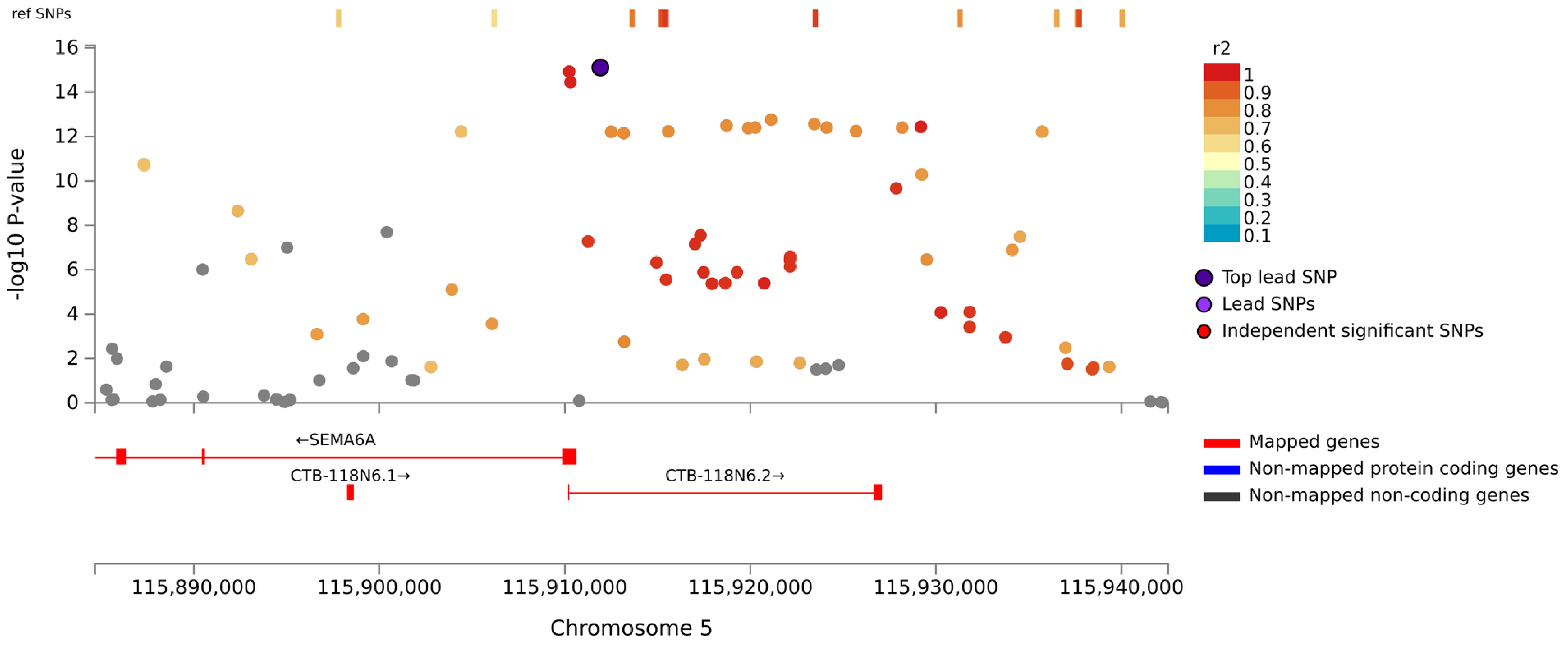

Locus 10, HBEGF, Isthmus Area, rs4150210

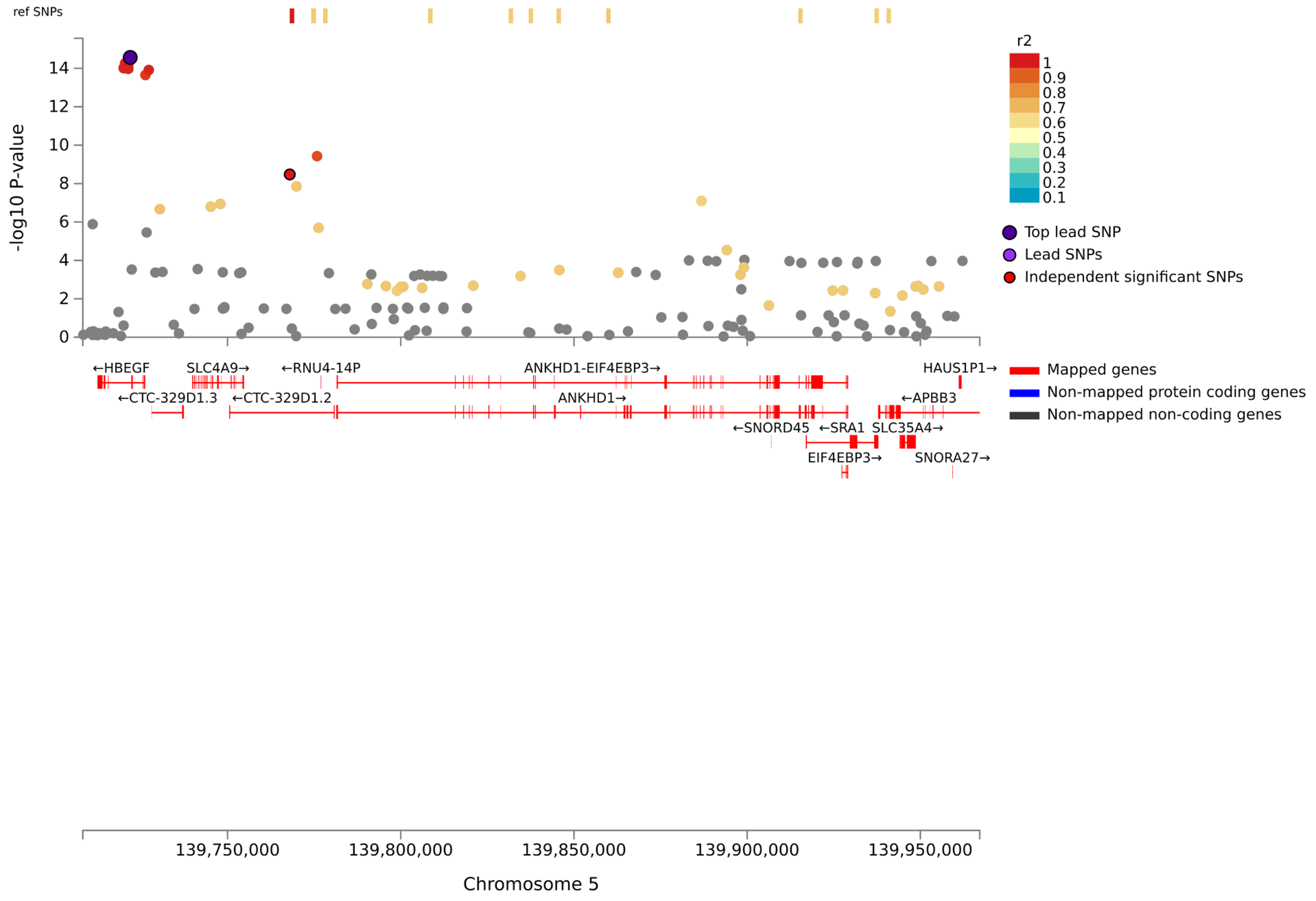

Locus 11, FOXO3, Isthmus Area, rs1268163

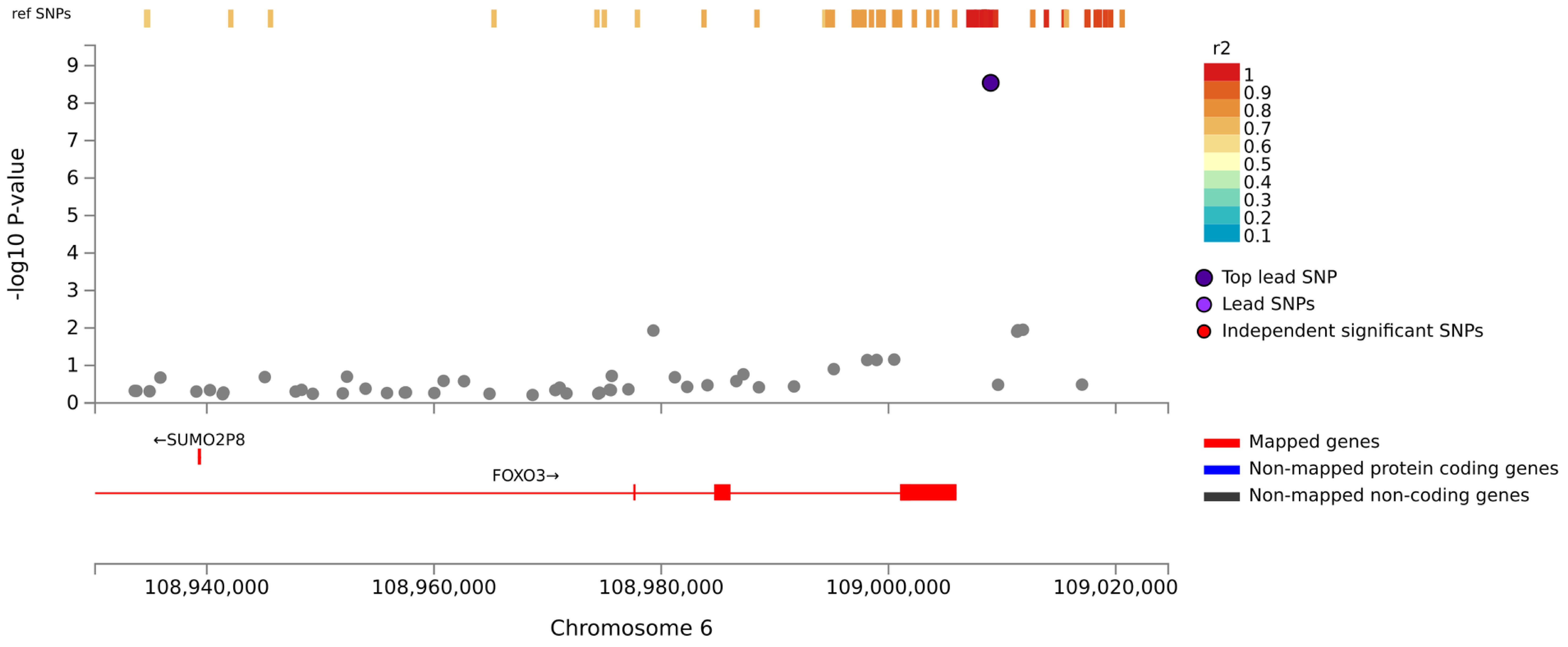

Locus 12, PLEC, Isthmus Area, rs6991364

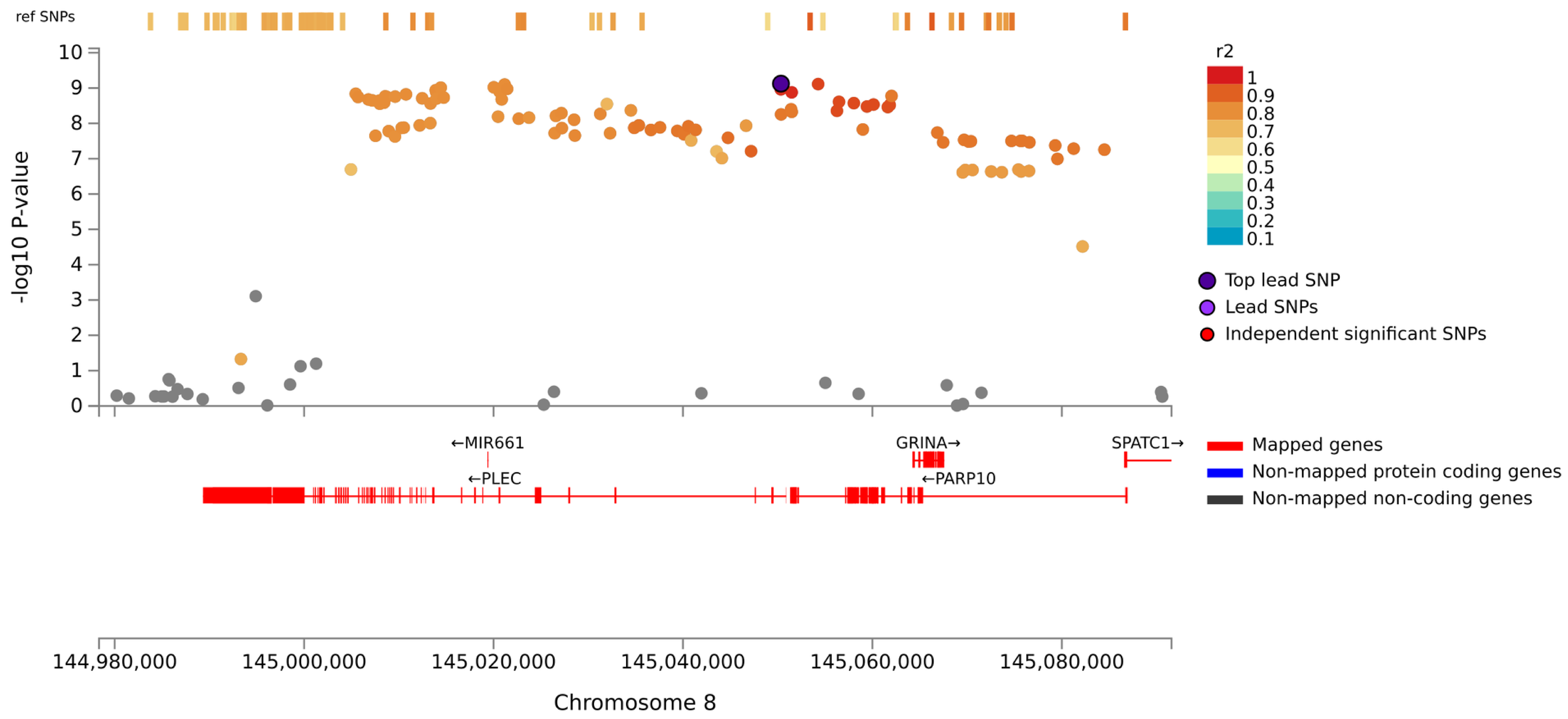

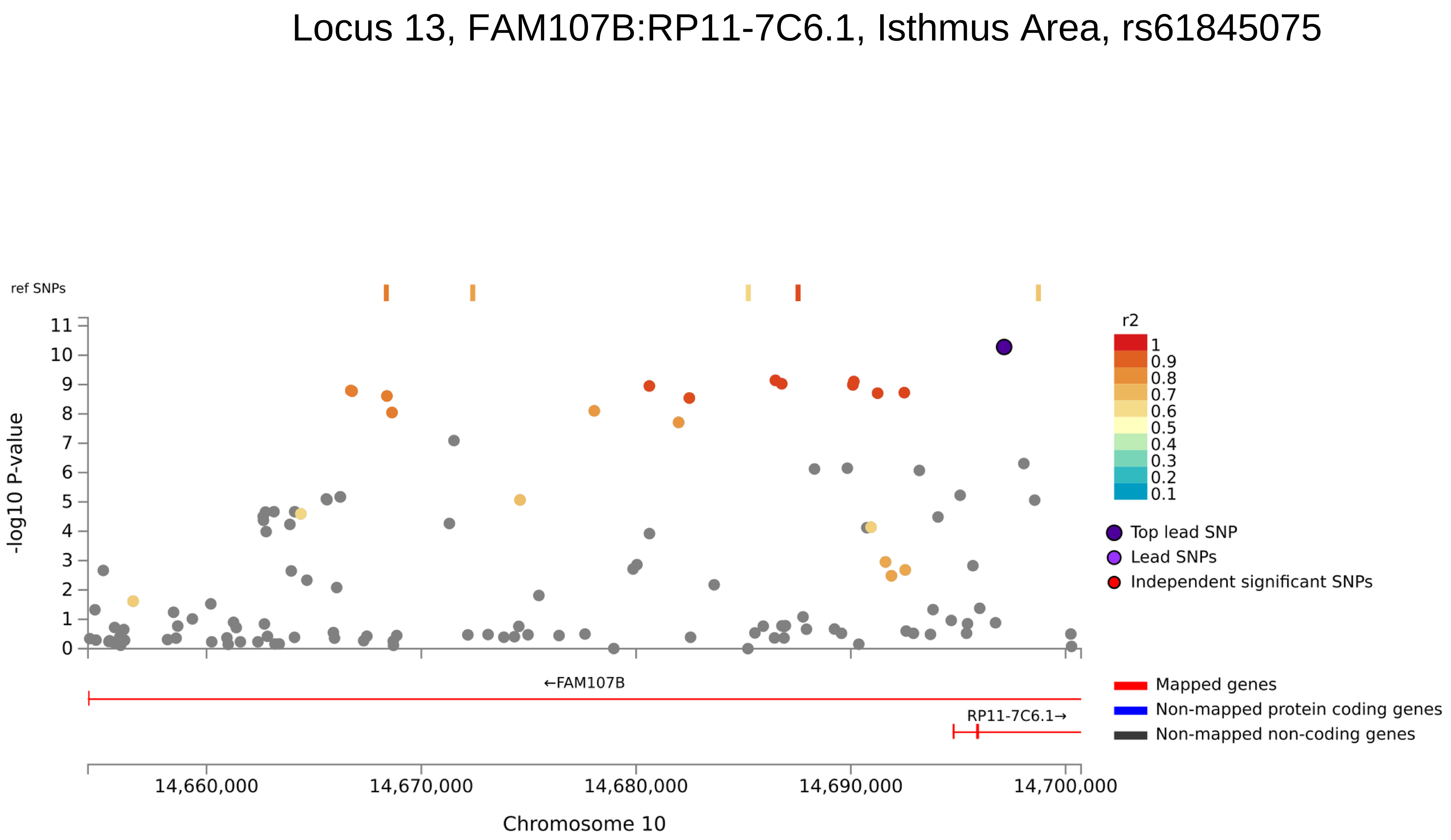
Locus 13, FAM107B:RP11-7C6.1, Isthmus Area, rs61845075
ref SNPs
-log10 P-value
r2
Top lead SNP
Lead SNPs
Independent significant SNPs
←FAM107B
RP11-7C6.1→
Mapped genes
Non-mapped protein coding genes
Non-mapped non-coding genes
14,660,000
14,670,000
14,680,000
14,690,000
14,700,000
Chromosome 10

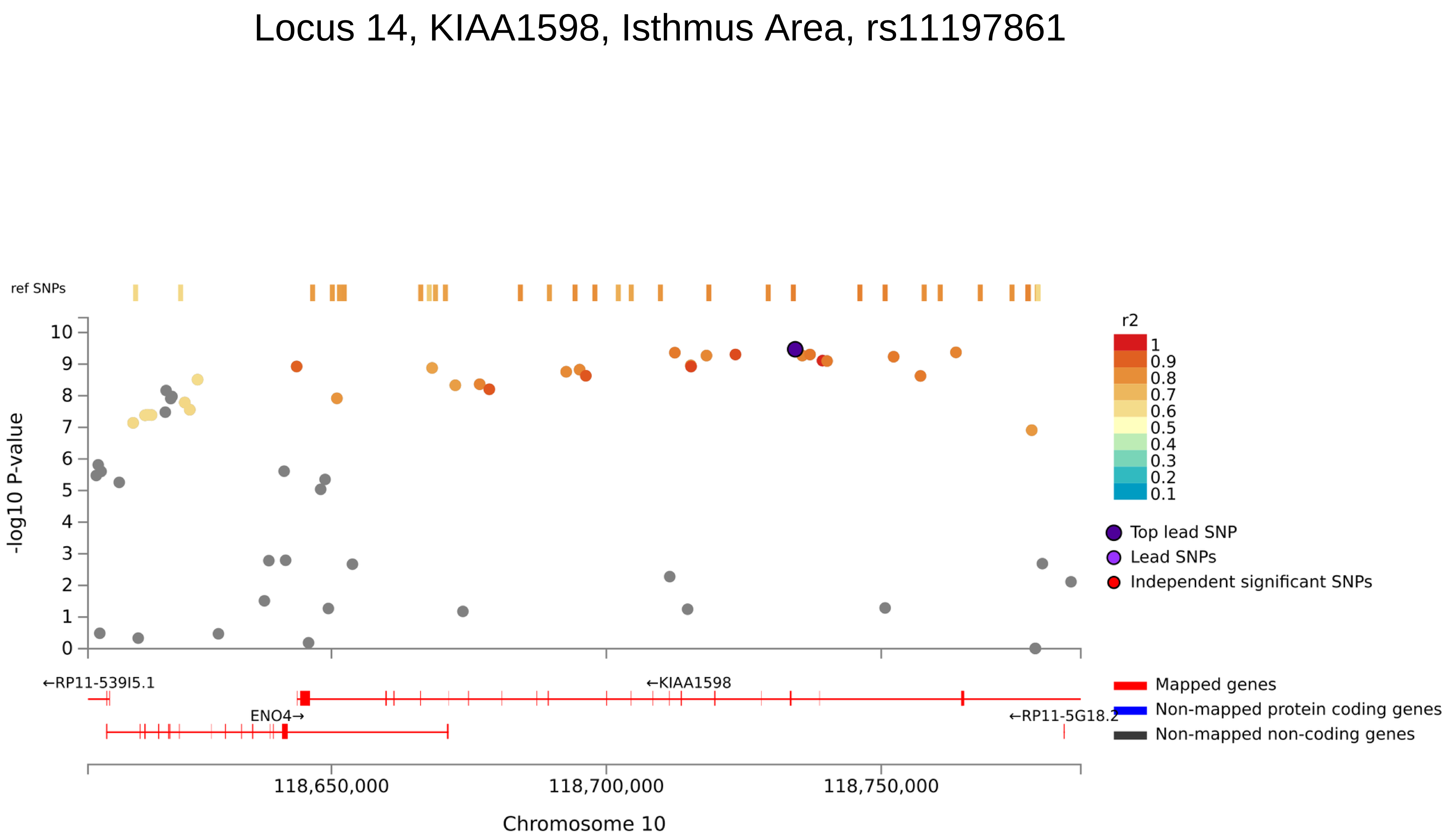
Locus 14, KIAA1598, Isthmus Area, rs11197861
ref SNPs
-log10 P-value
r2
Top lead SNP
Lead SNPs
Independent significant SNPs
←RP11-539I5.1
←KIAA1598
ENO4→
←RP11-5G18.2
Mapped genes
Non-mapped protein coding genes
Non-mapped non-coding genes
118,650,000
118,700,000
118,750,000
Chromosome 10

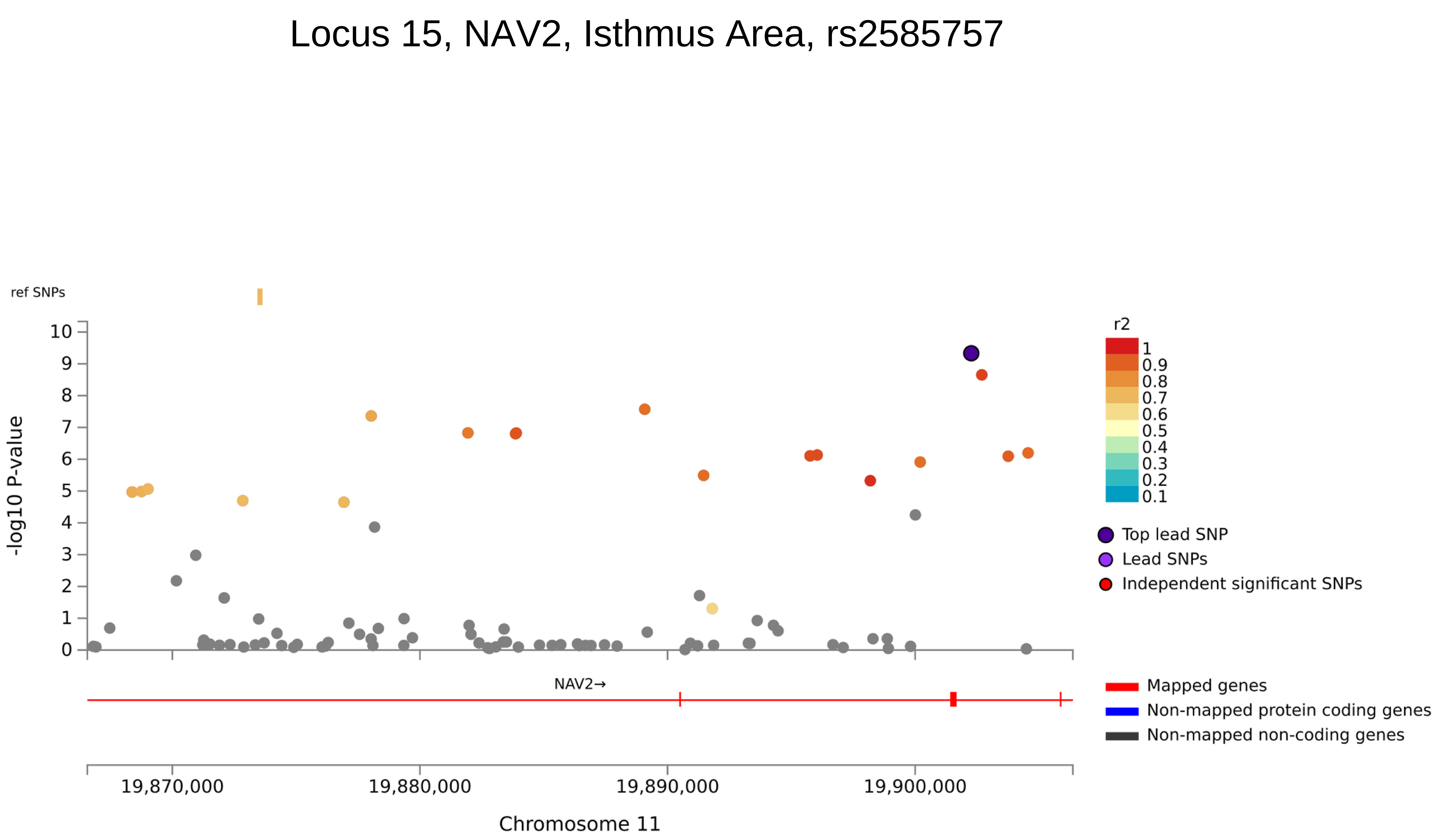

Locus 15, NAV2, Isthmus Area, rs2585757
ref SNPs
-log10 P-value
r2
Top lead SNP
Lead SNPs
Independent significant SNPs
NAV2→
Mapped genes
Non-mapped protein coding genes
Non-mapped non-coding genes
19,870,000
19,880,000
19,890,000
19,900,000
Chromosome 11

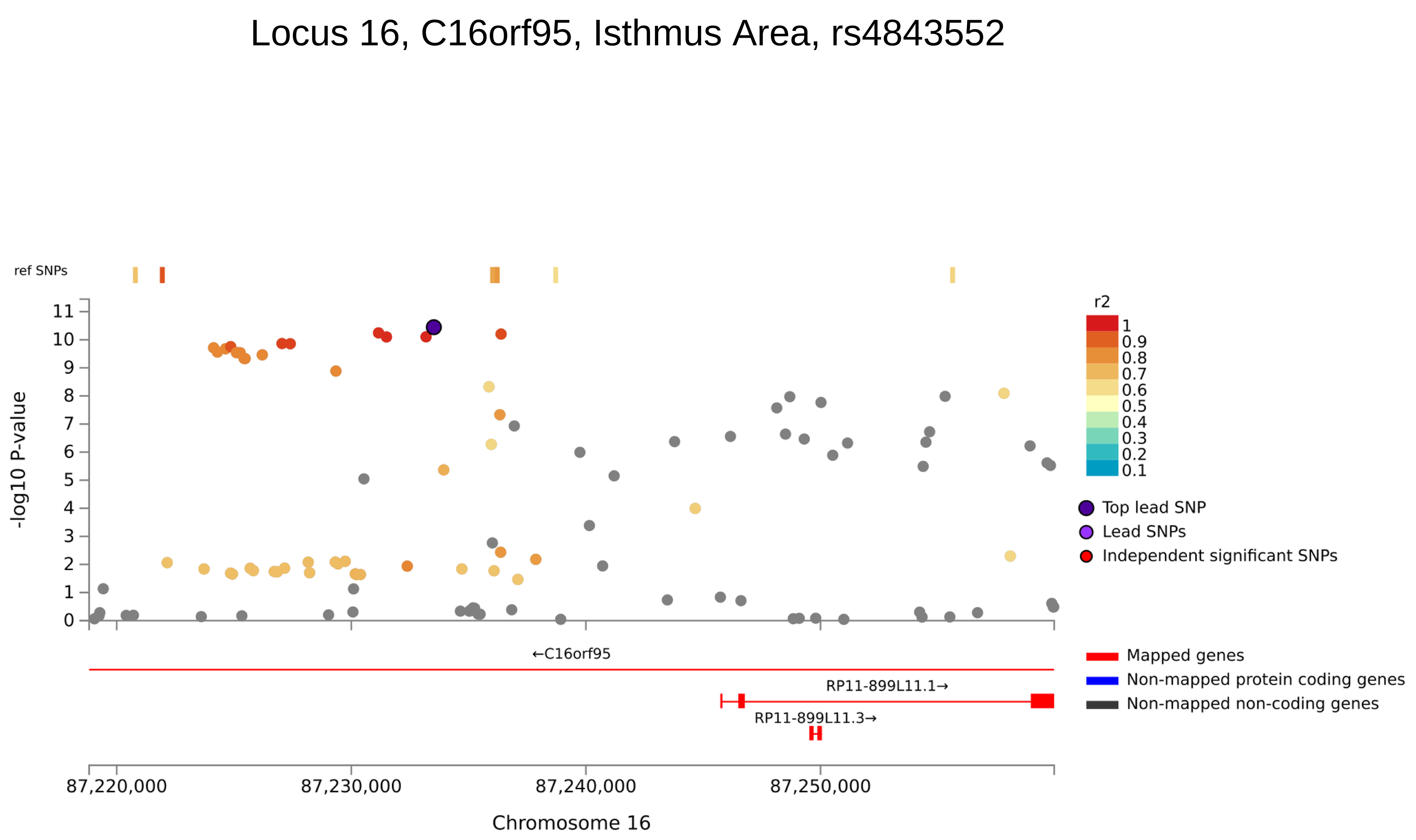

Locus 16, C16orf95, Isthmus Area, rs4843552
ref SNPs
-log10 P-value
r2
Top lead SNP
Lead SNPs
Independent significant SNPs
←C16orf95
RP11-899L11.1→
RP11-899L11.3→
Mapped genes
Non-mapped protein coding genes
Non-mapped non-coding genes
87,220,000
87,230,000
87,240,000
87,250,000
Chromosome 16

Locus 17, CABYR, Isthmus Area, rs752797

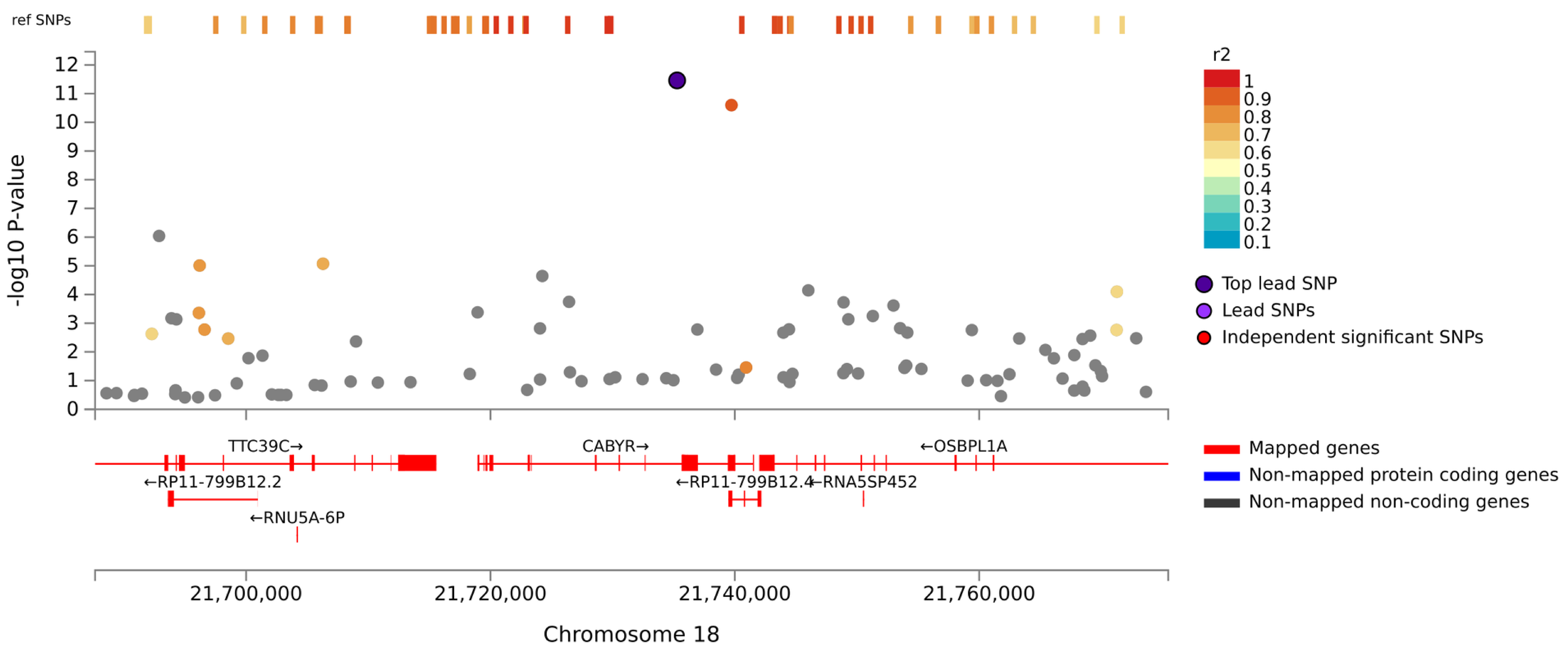

Locus 1, RP1-37C10.3:ATP13A2, Splenium Area, rs2076603

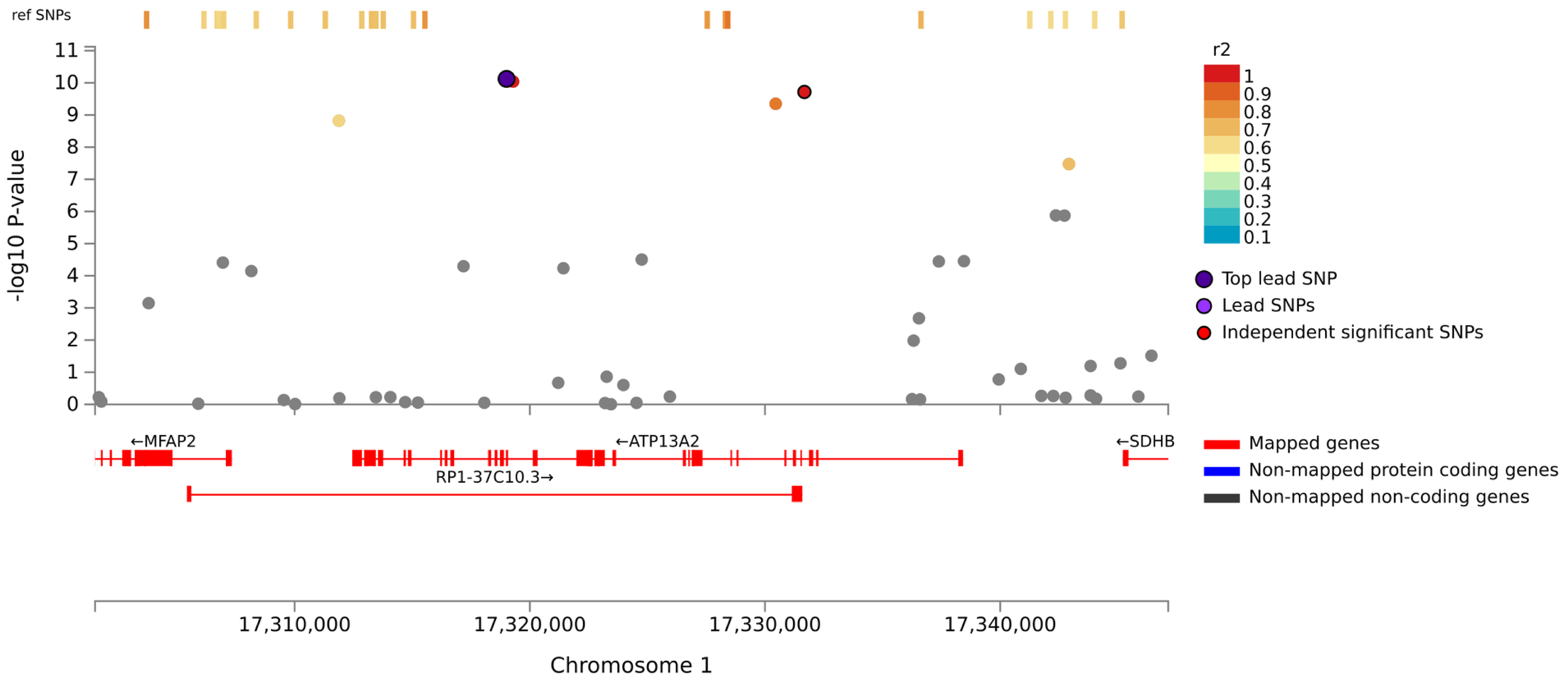

Locus 2, AKT3, Splenium Area, rs67027895

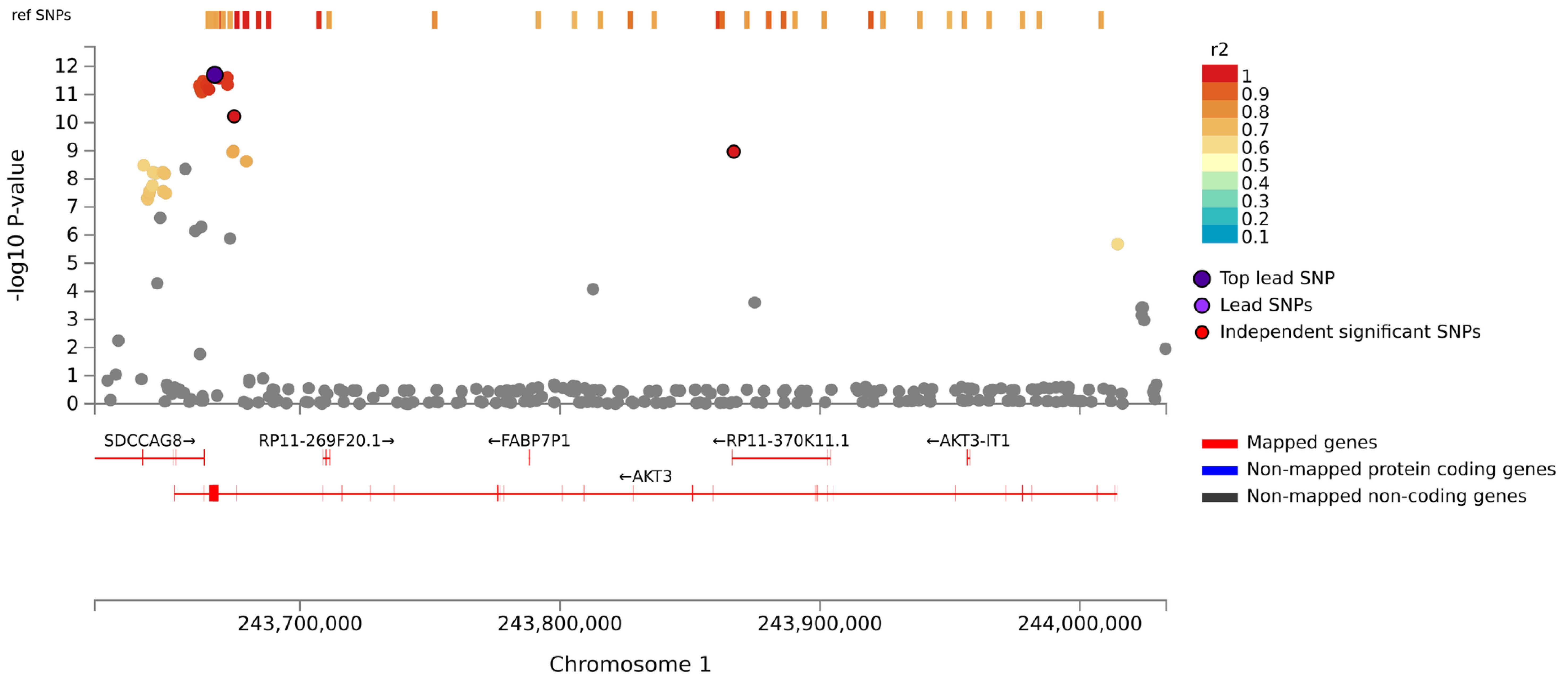

Locus 3, AC007382.1, Splenium Area, rs711244

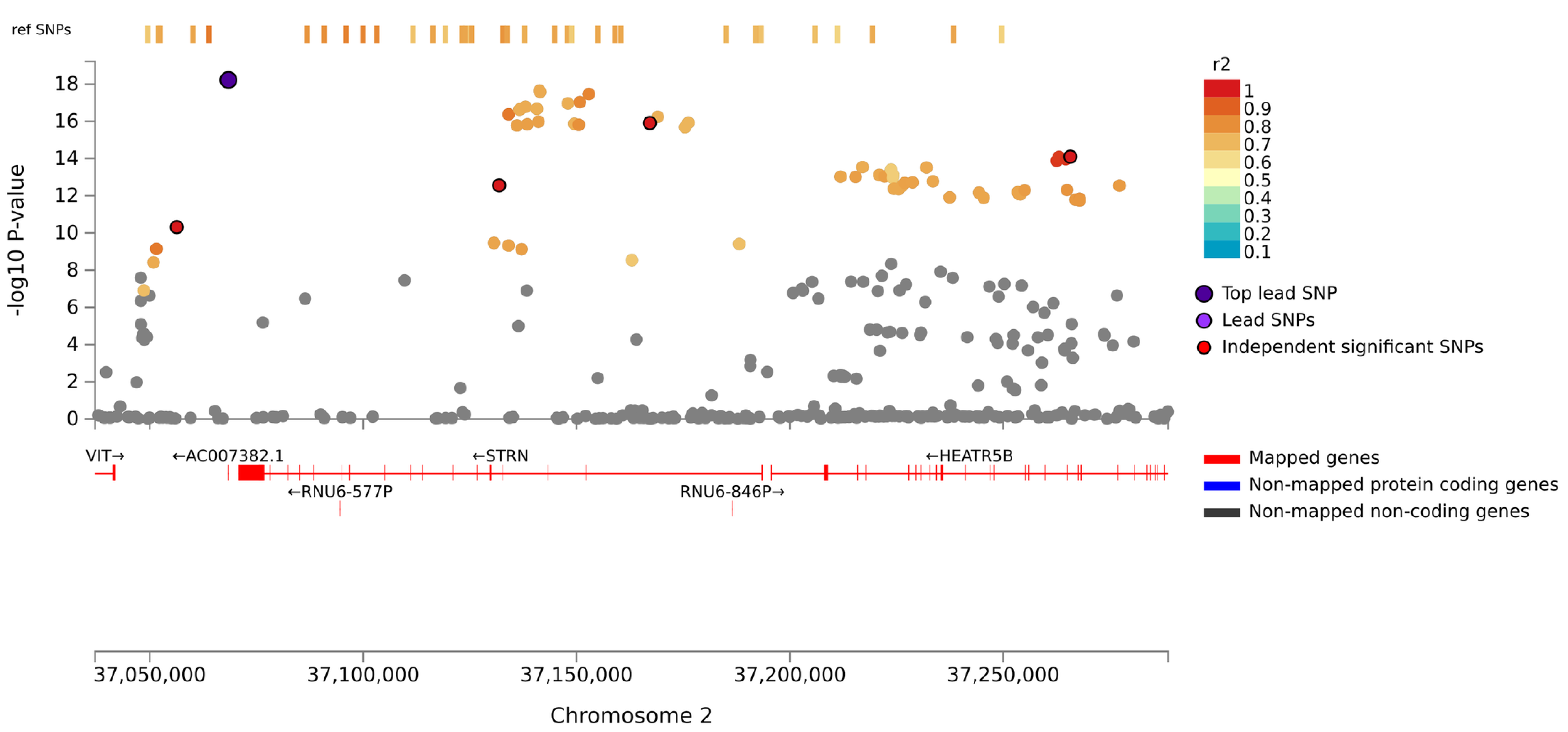

Locus 4, XPO1, Splenium Area, rs7570830

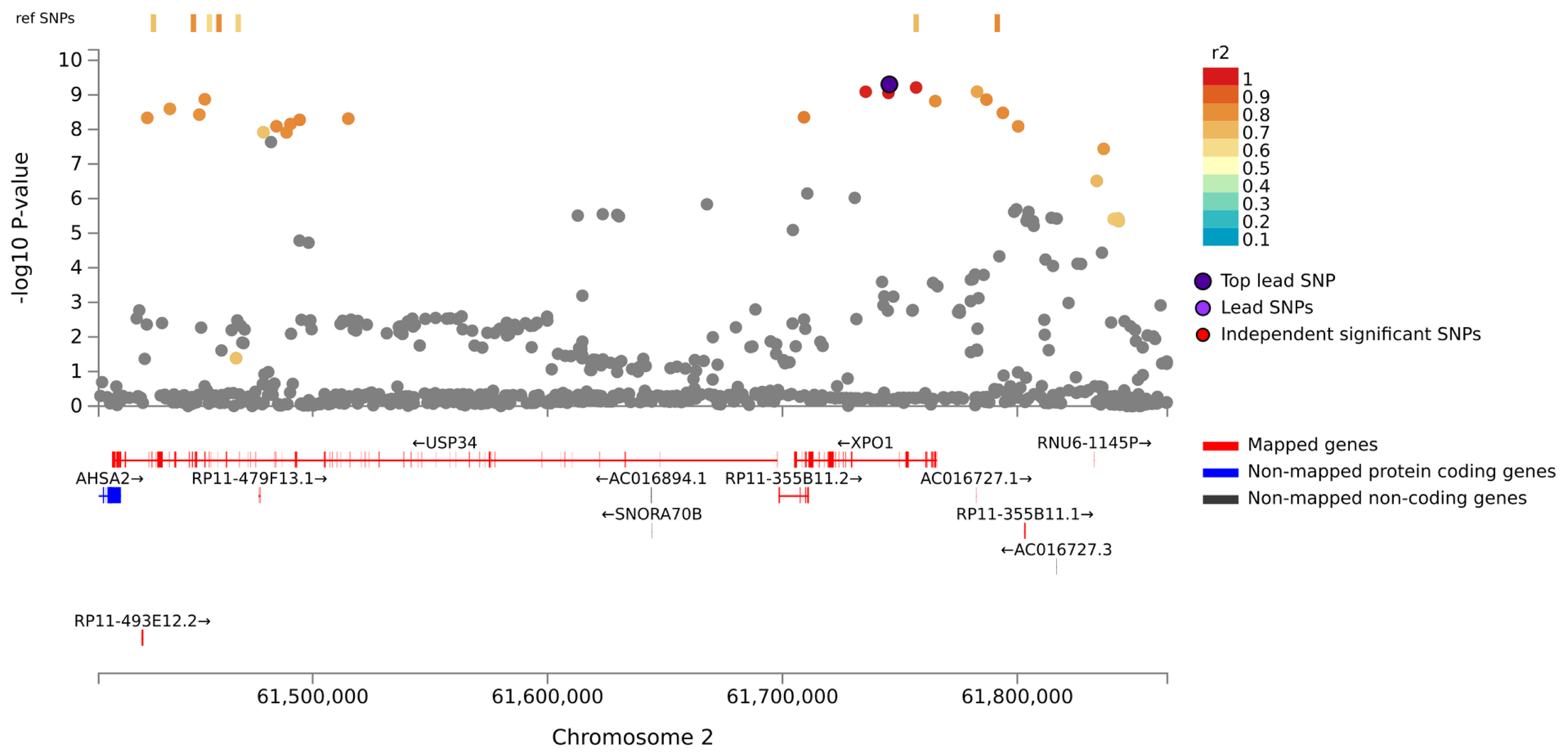

Locus 5, RP11-493K19.3, Splenium Area, rs2071206

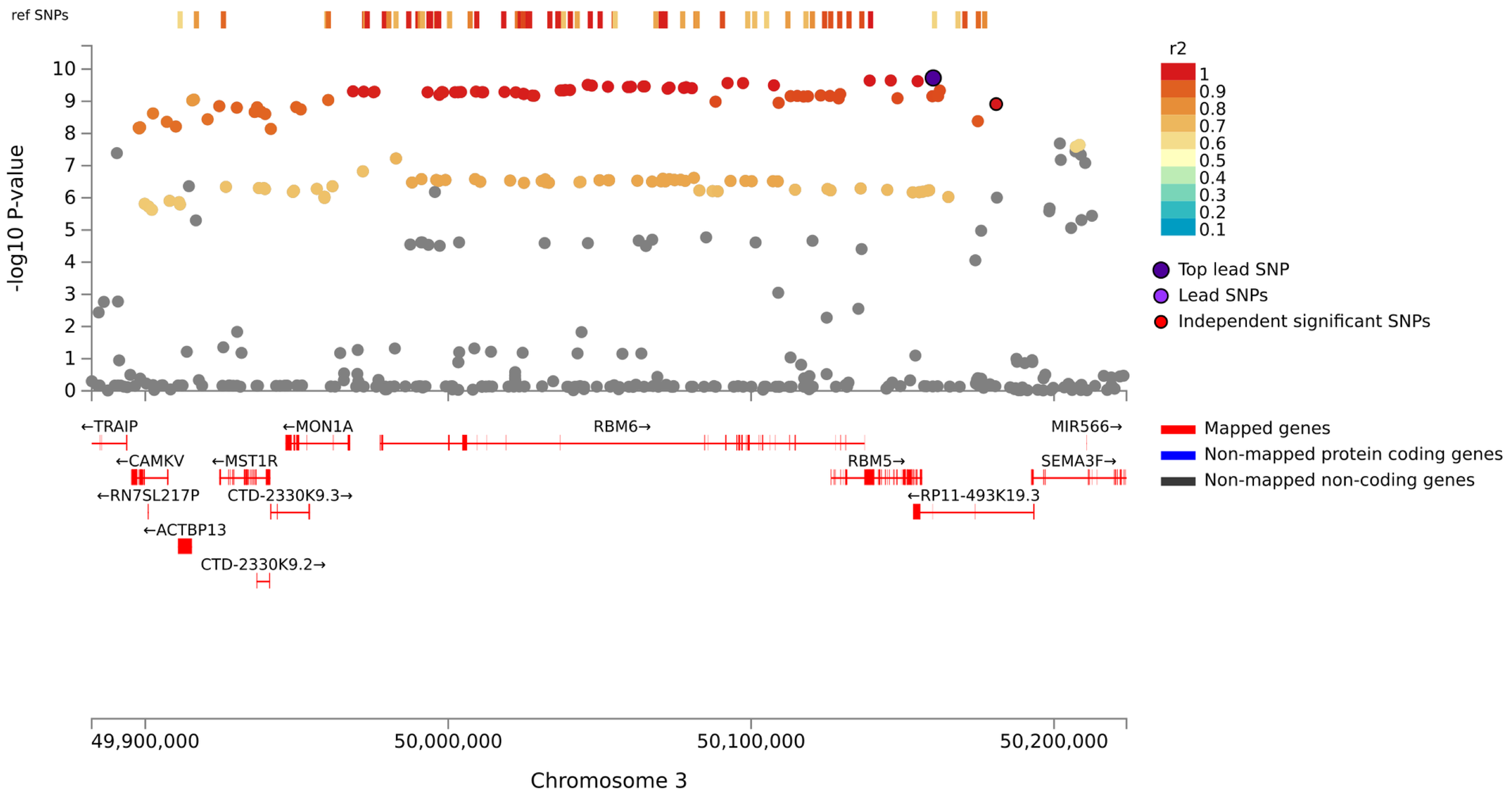

Locus 6, CCDC75P1, Splenium Area, rs56934393

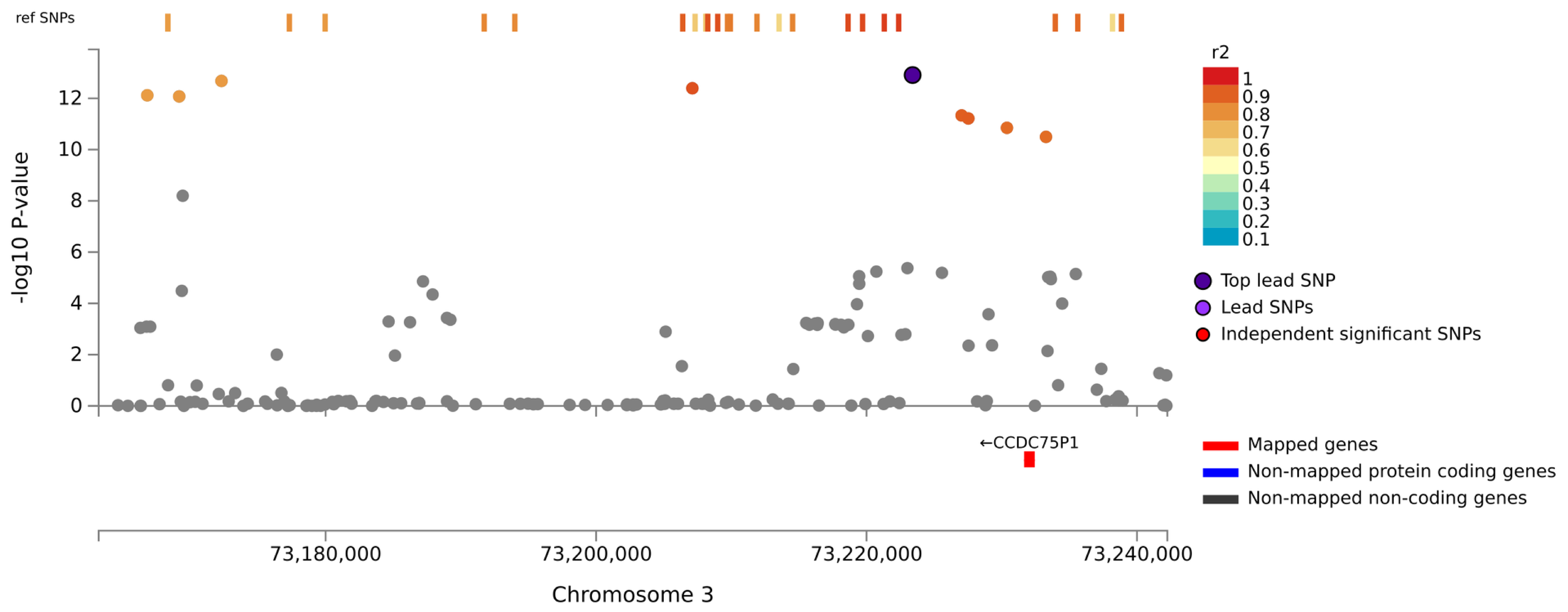

Locus 7, IQCJ-SCHIP1:IQCJ, Splenium Area, rs11717303

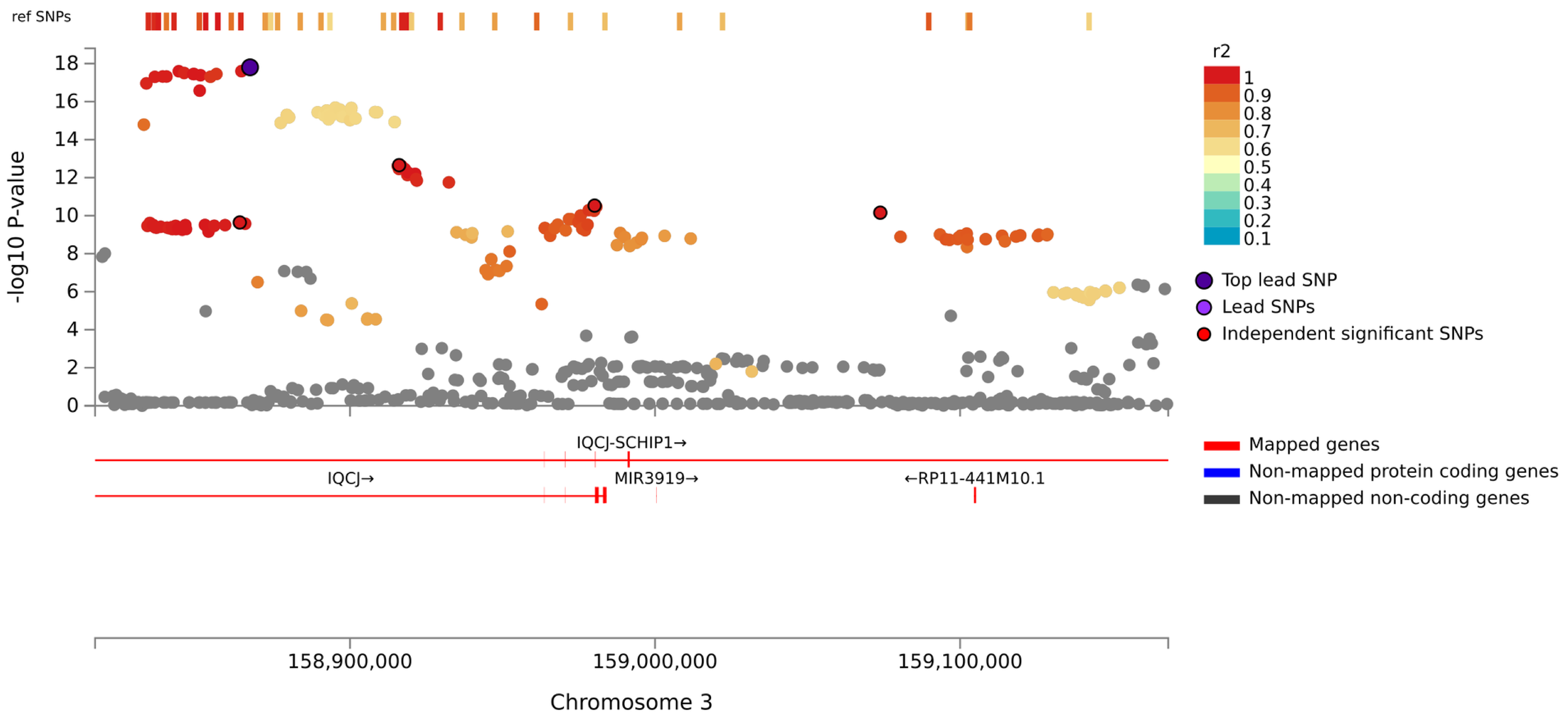

Locus 8, IQCJ-SCHIP1, Splenium Area, rs13064756

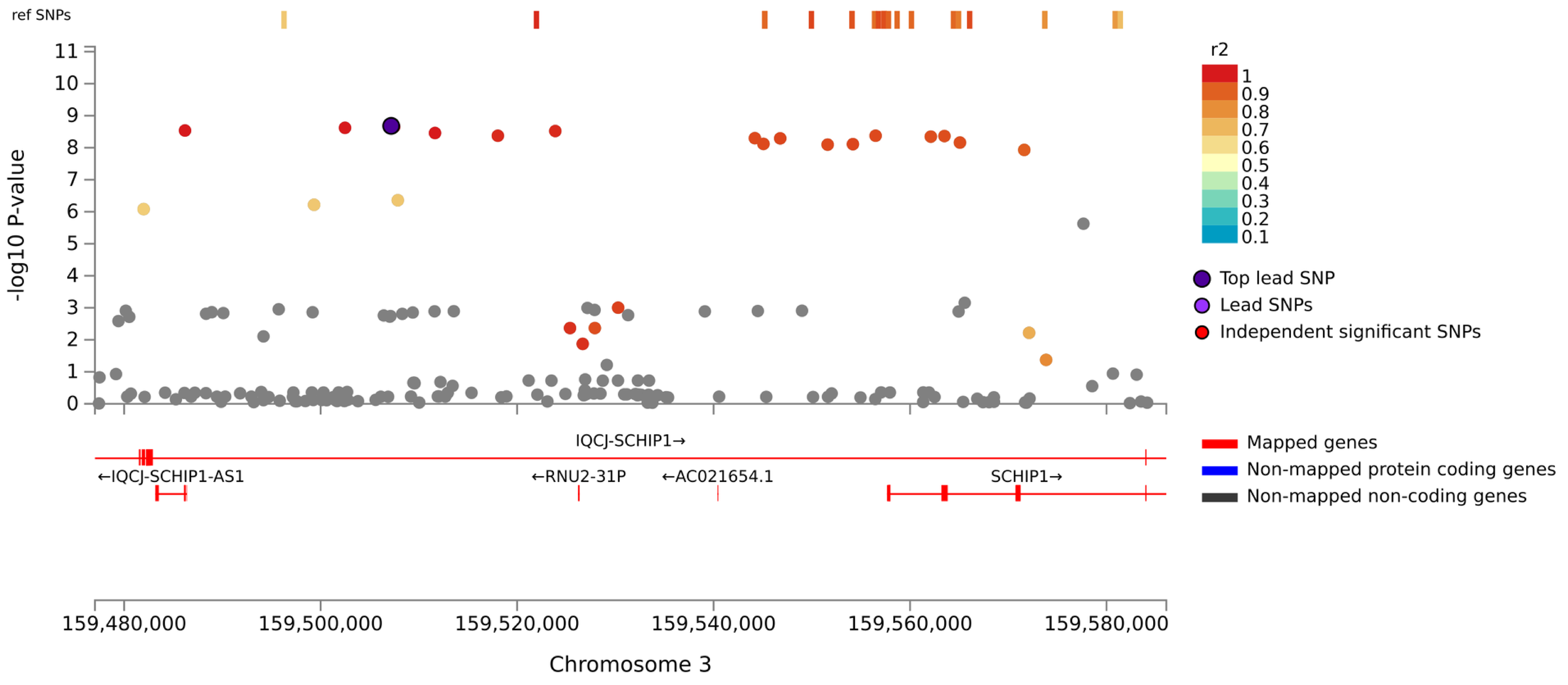

Locus 9, TNIK, Splenium Area, rs6444974

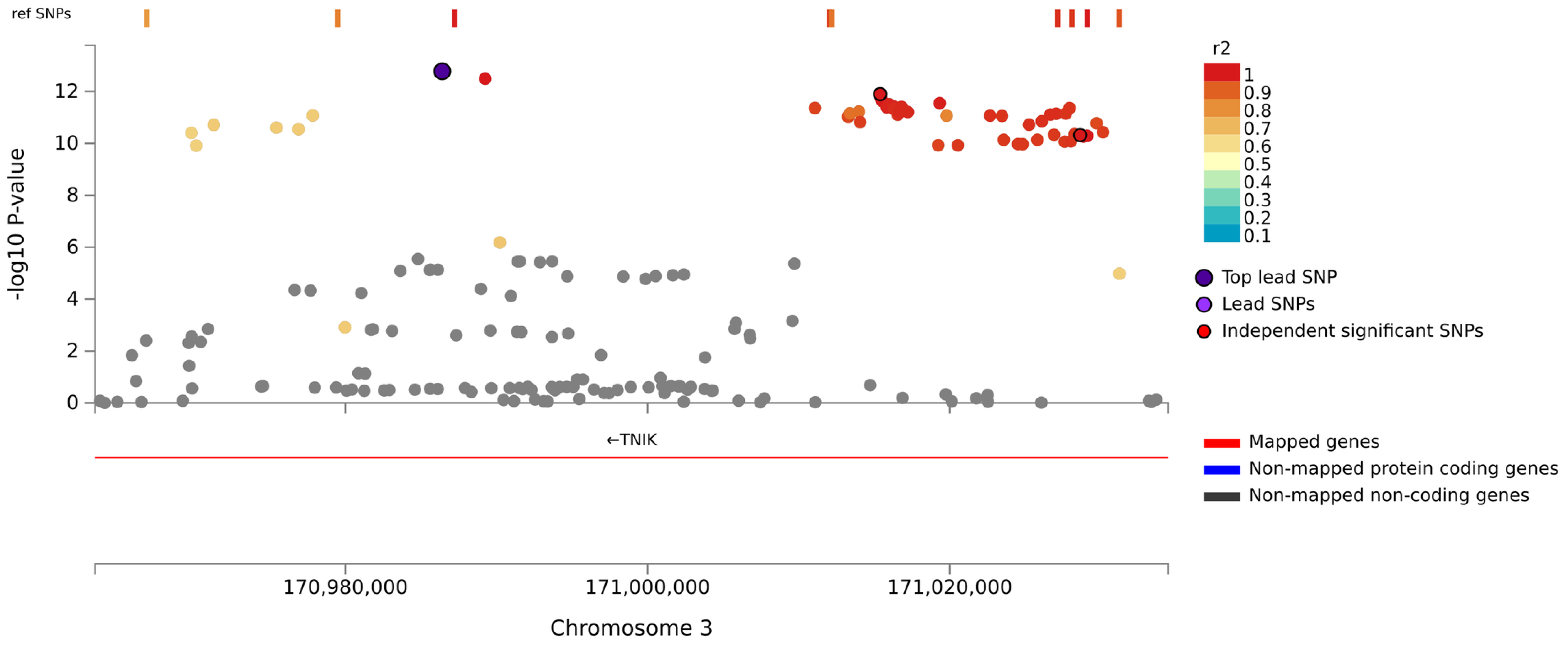

Locus 10, FIP1L1, Splenium Area, rs6849897

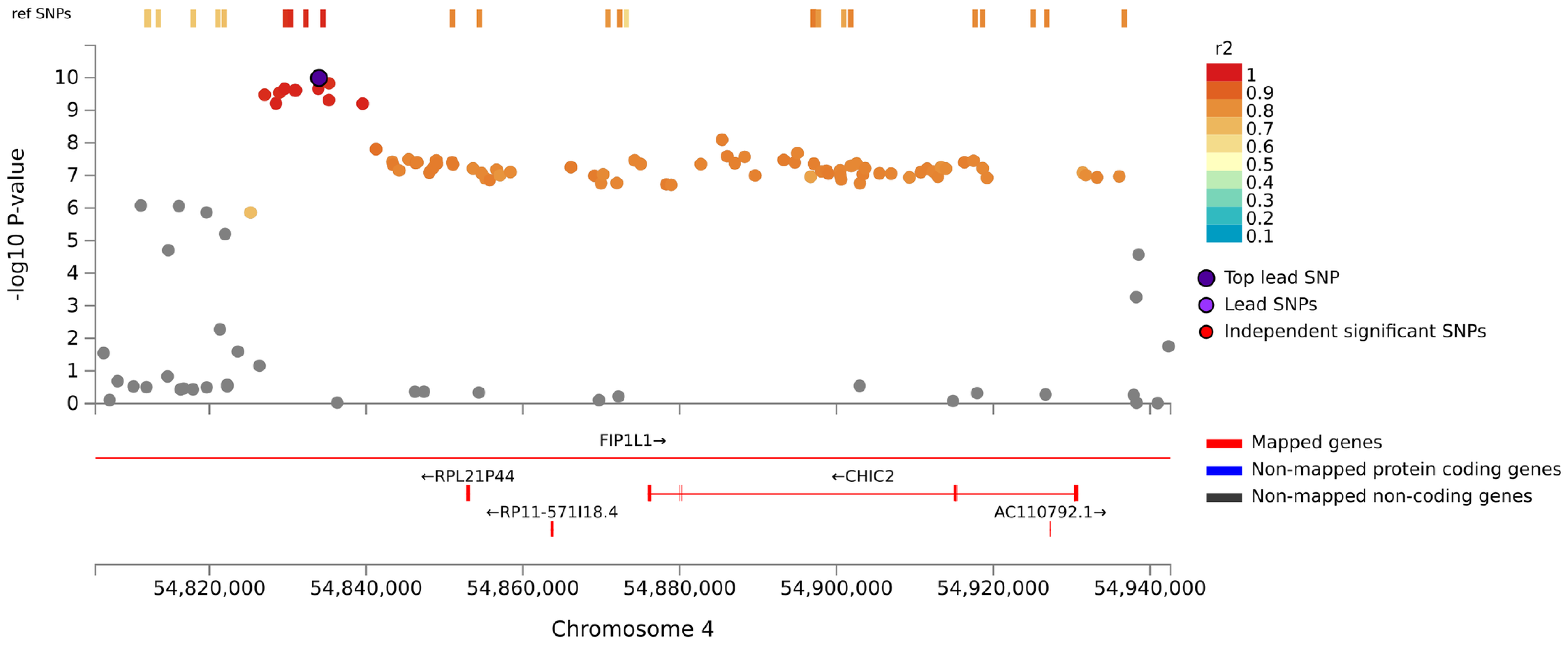

Locus 11, STPG2:RP11-681L8.1:STPG2-AS1, Splenium Area, rs62315838

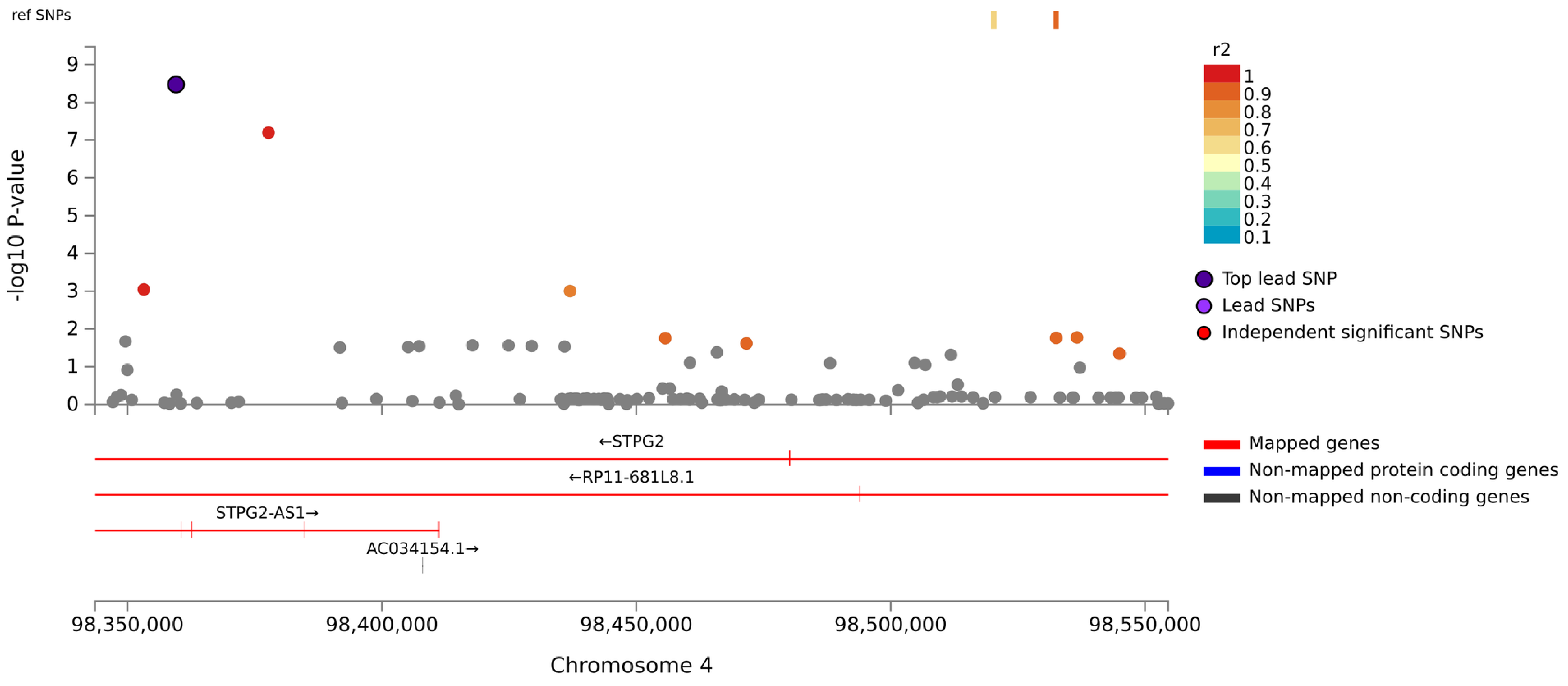

Locus 12, FBXL7, Splenium Area, rs4702099

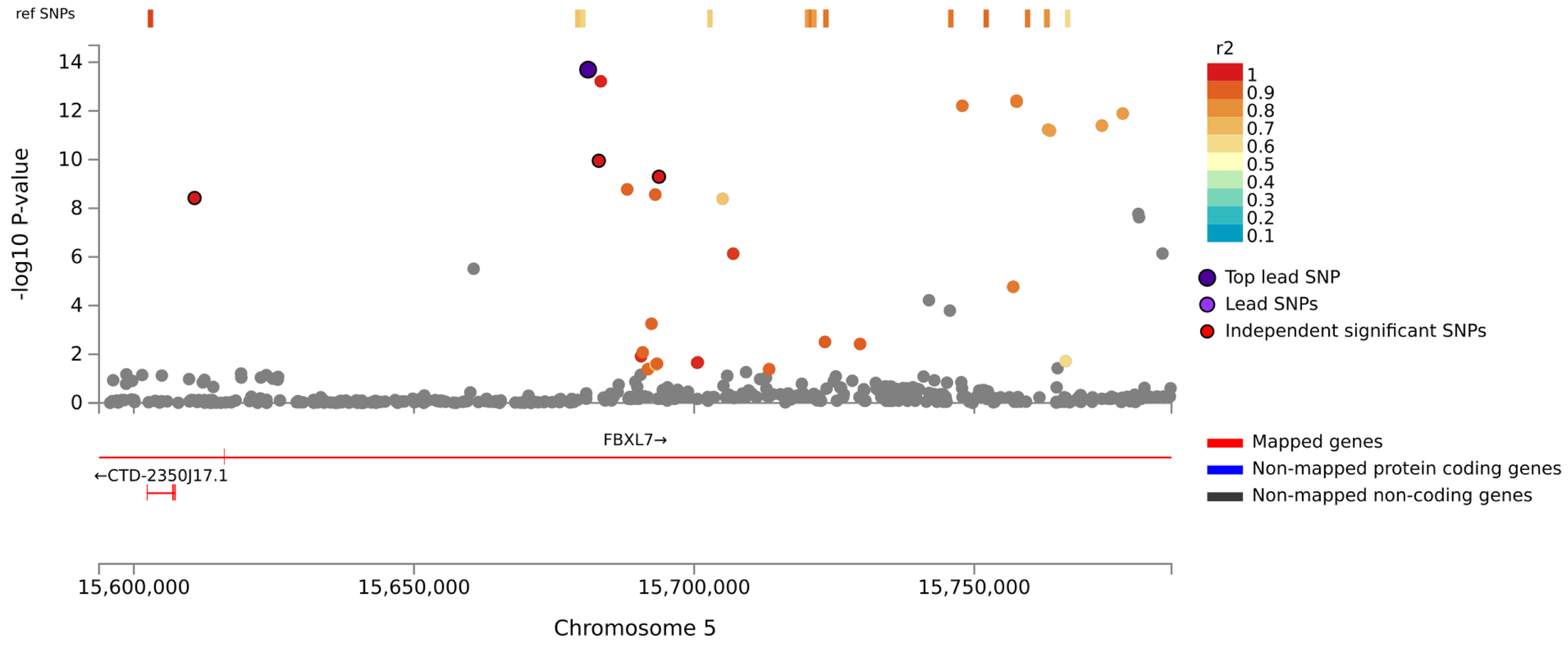

Locus 13, CTB-118N6.2, Splenium Area, rs686664

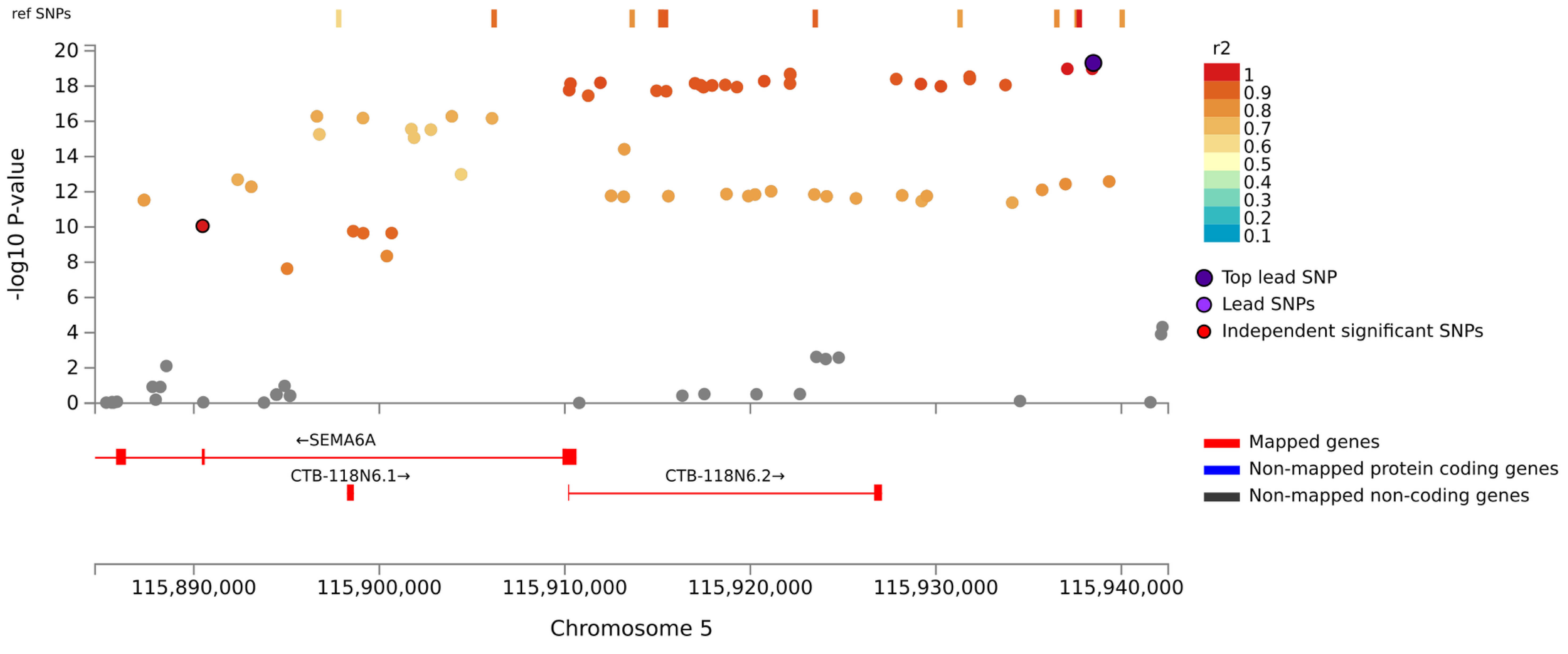

Locus 14, HBEGF, Splenium Area, rs58992612

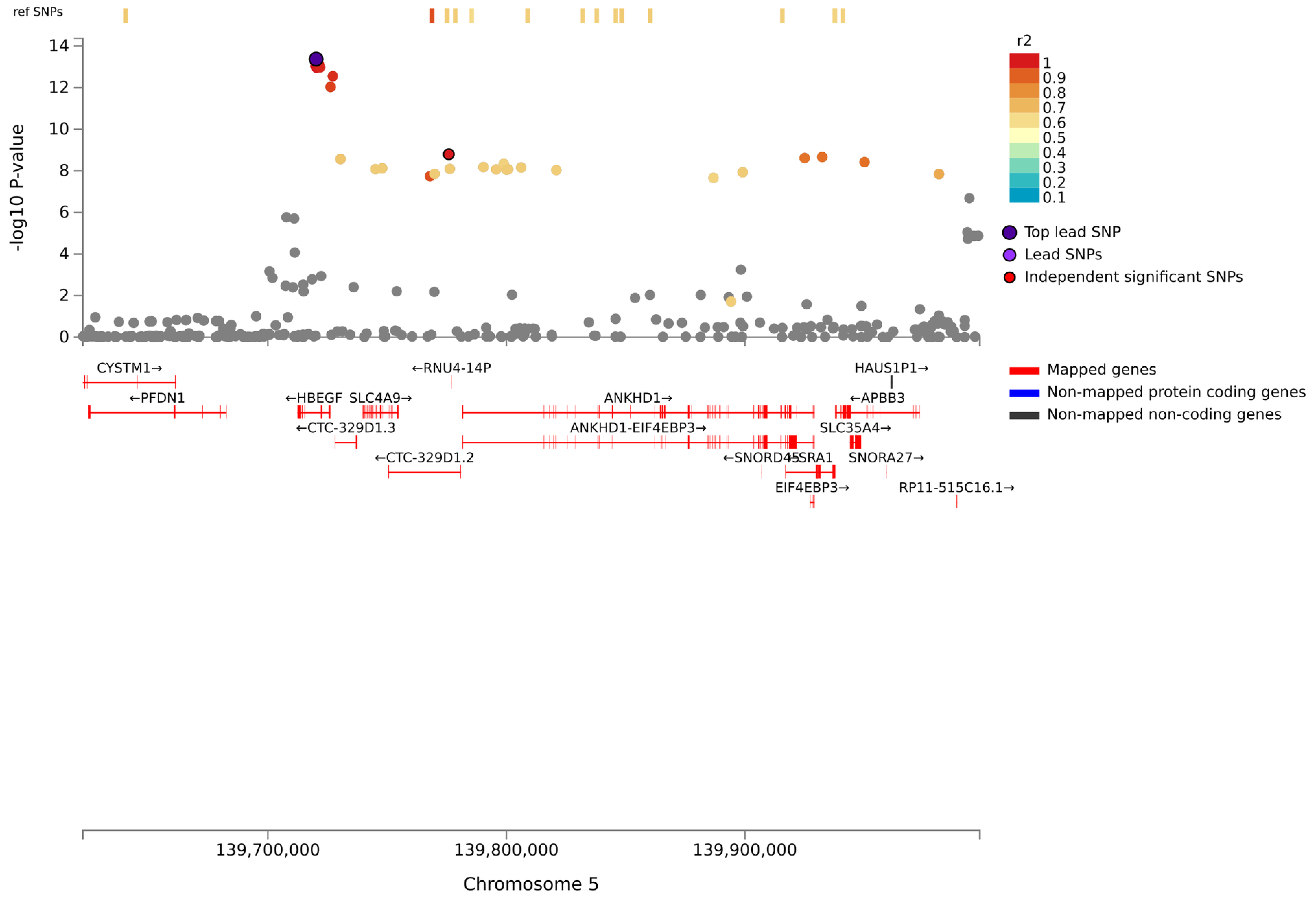

Locus 15, HGF, Splenium Area, rs60392694

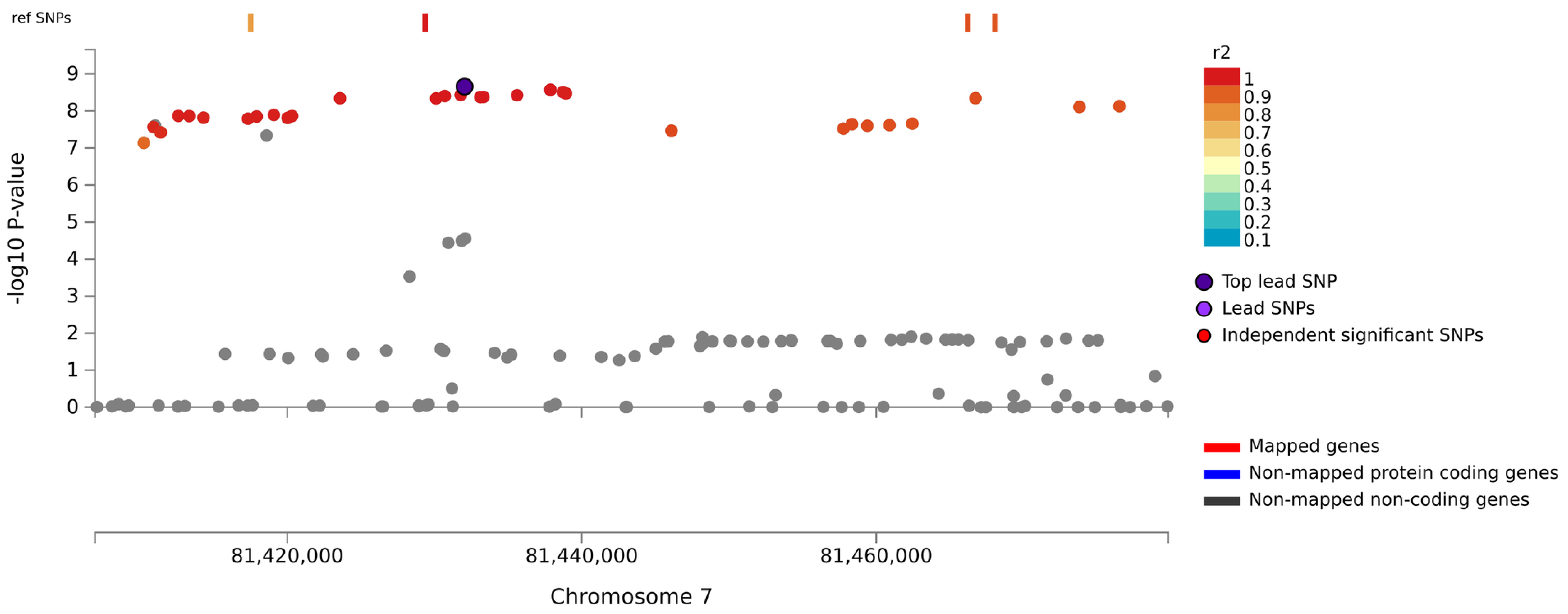

Locus 16, SEMA3A, Splenium Area, rs73712705

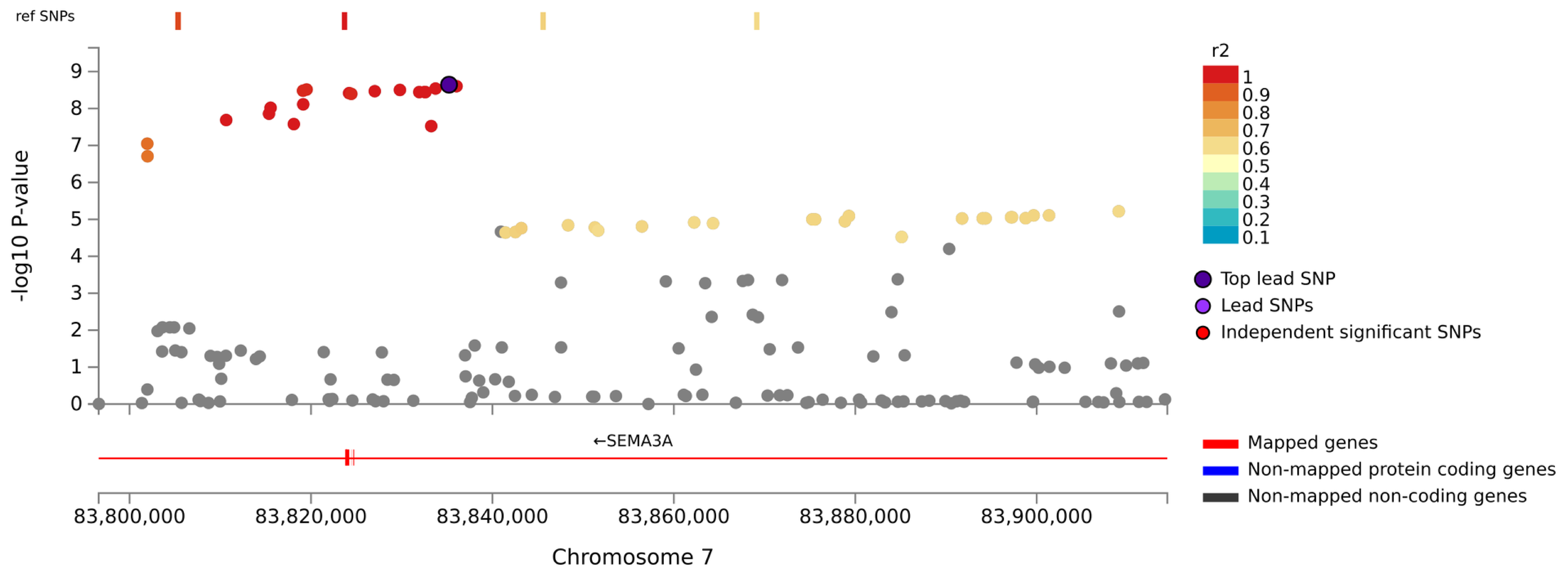

Locus 17, BICD2, Splenium Area, rs10992447

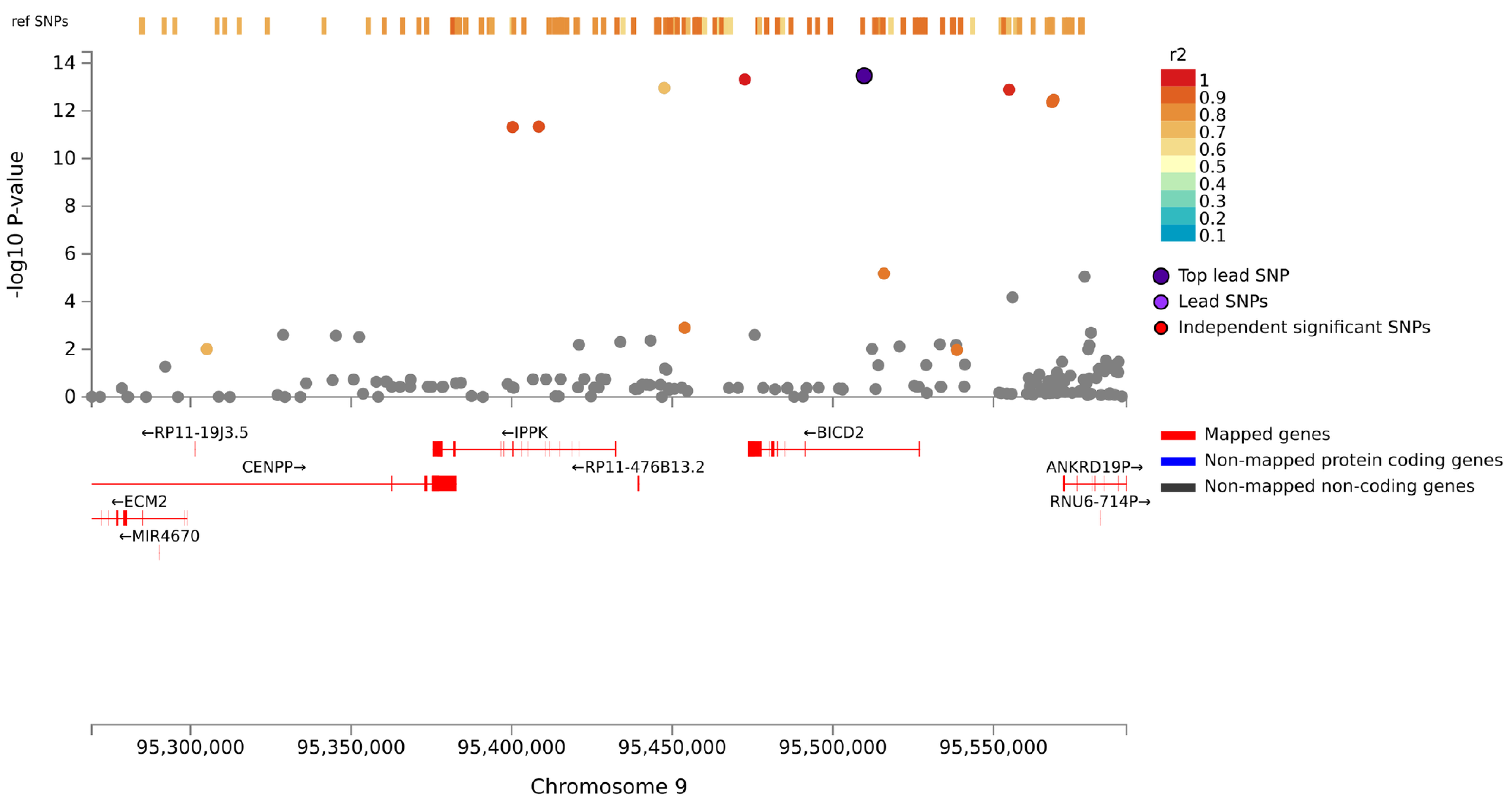

Locus 18, FAM107B, Splenium Area, rs10906729

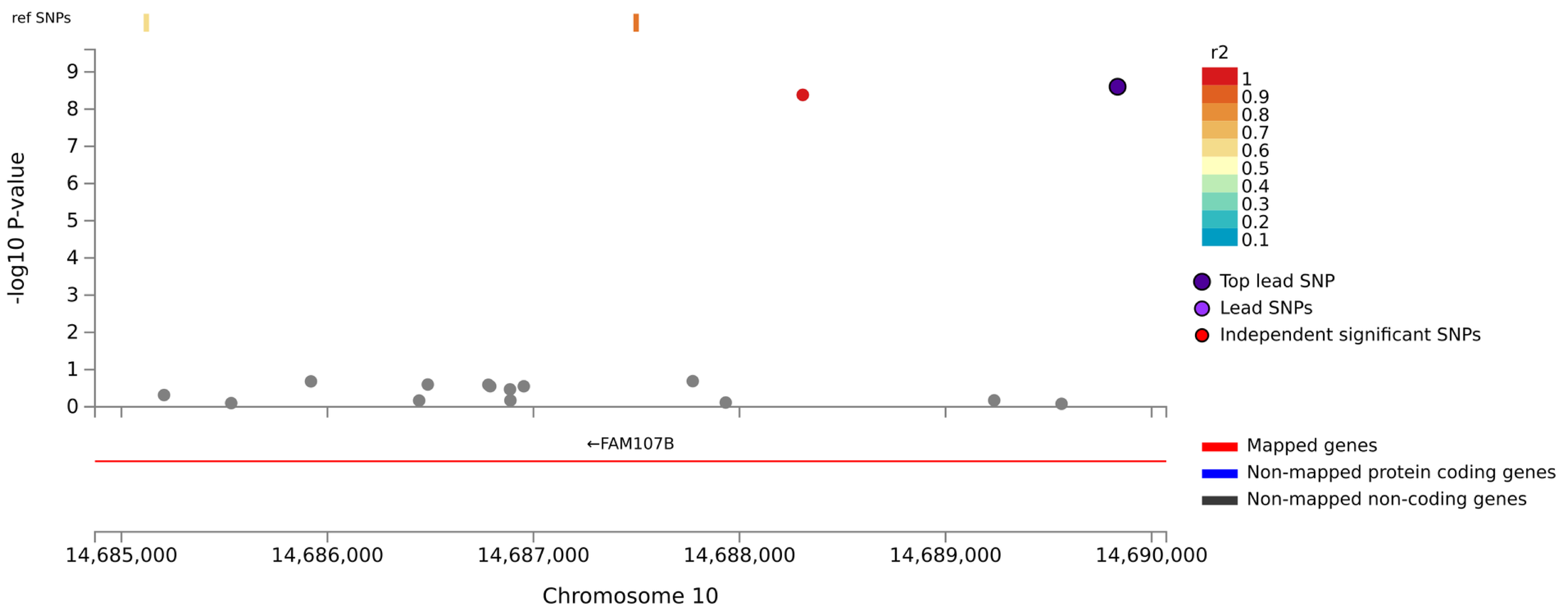

Locus 19, ENO4:KIAA1598, Splenium Area, rs10886016

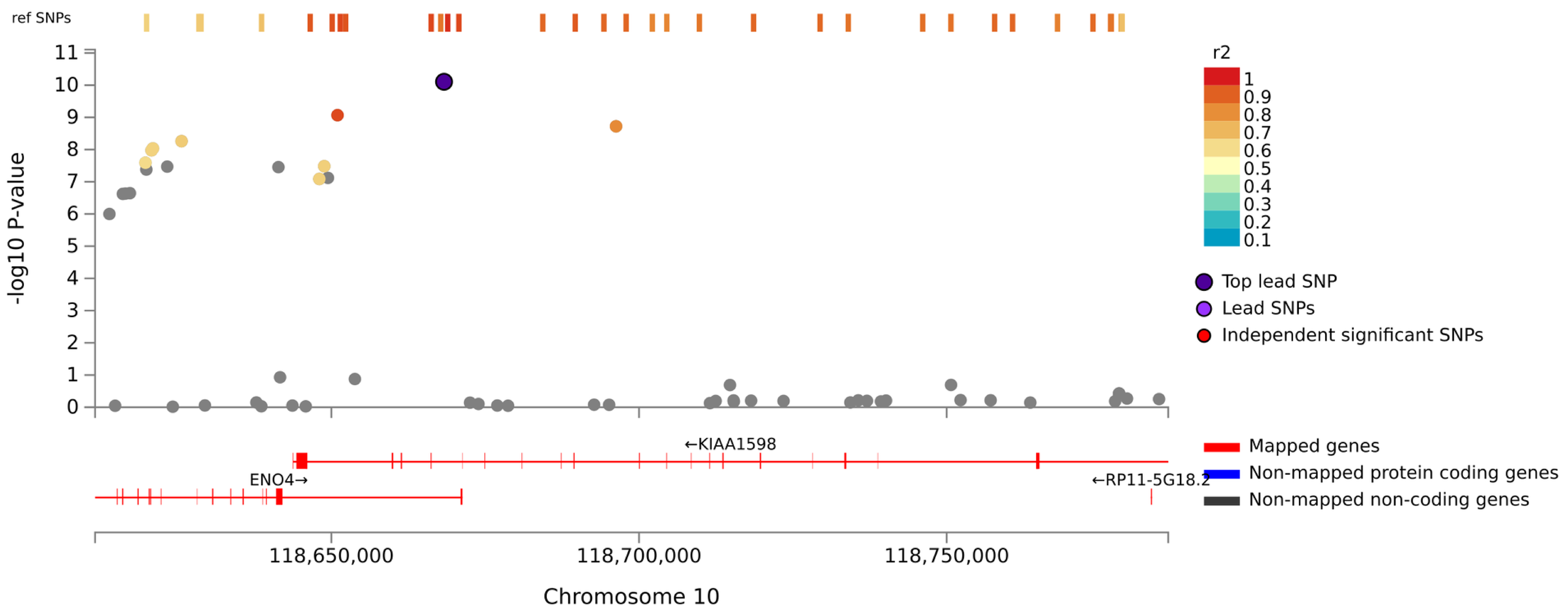

Locus 20, RP11-12J10.3:FAM53B, Splenium Area, rs10901814

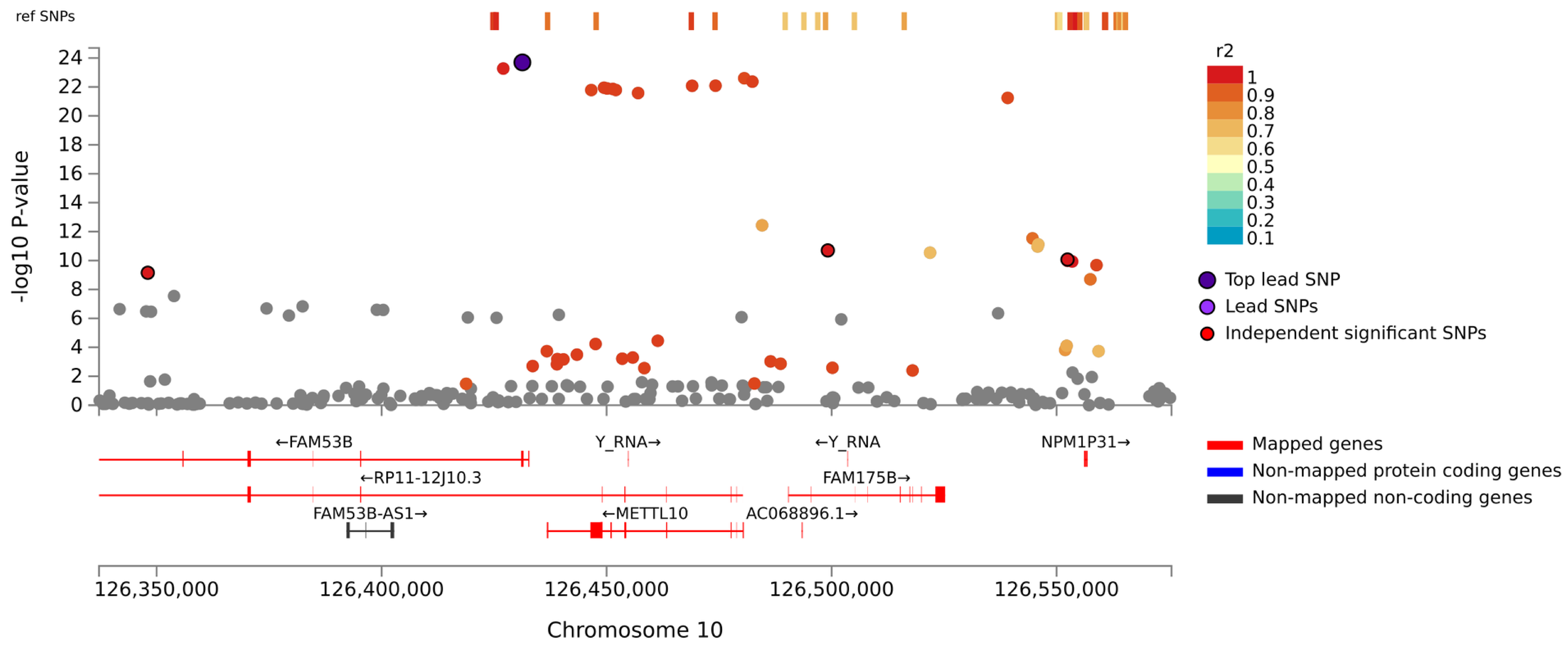

Locus 21, BRSK2, Splenium Area, rs7947308

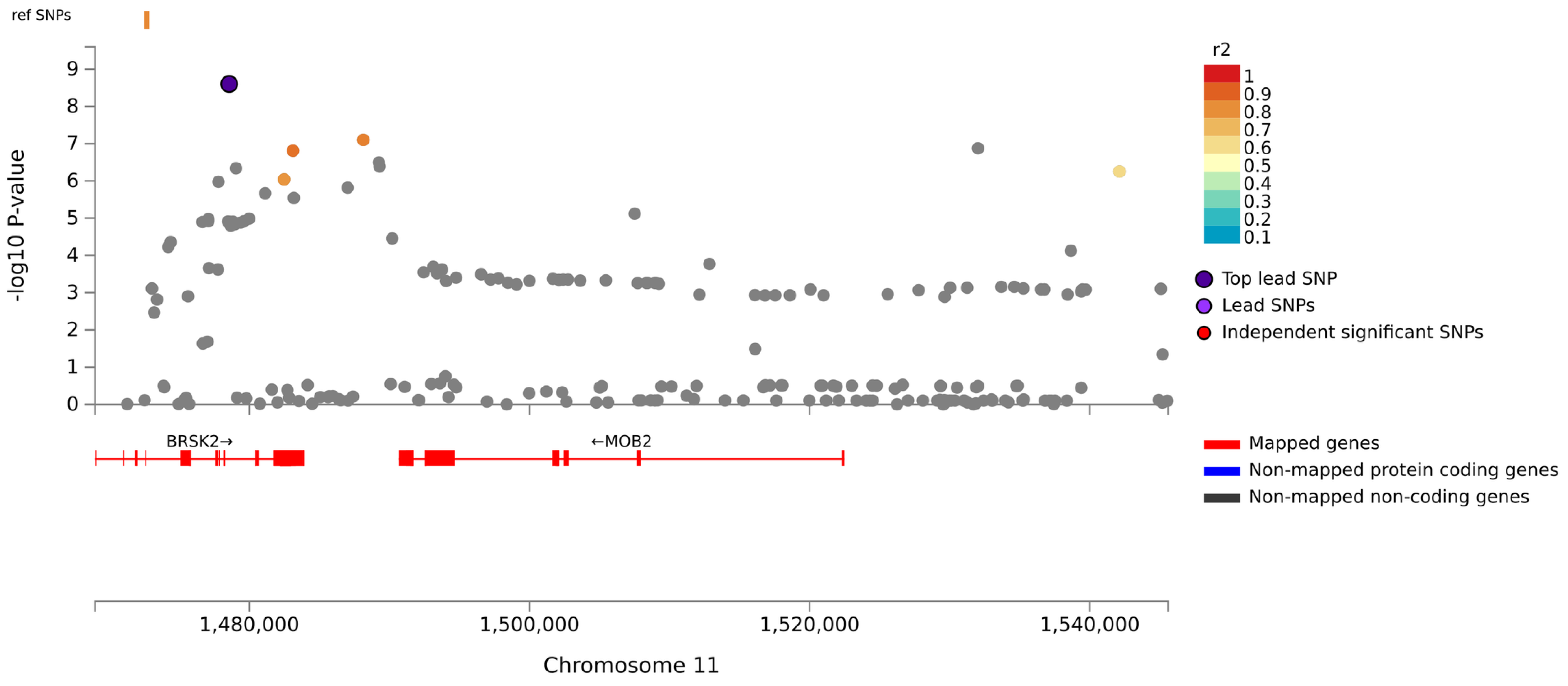

Locus 22, RRAS2, Splenium Area, rs35247669

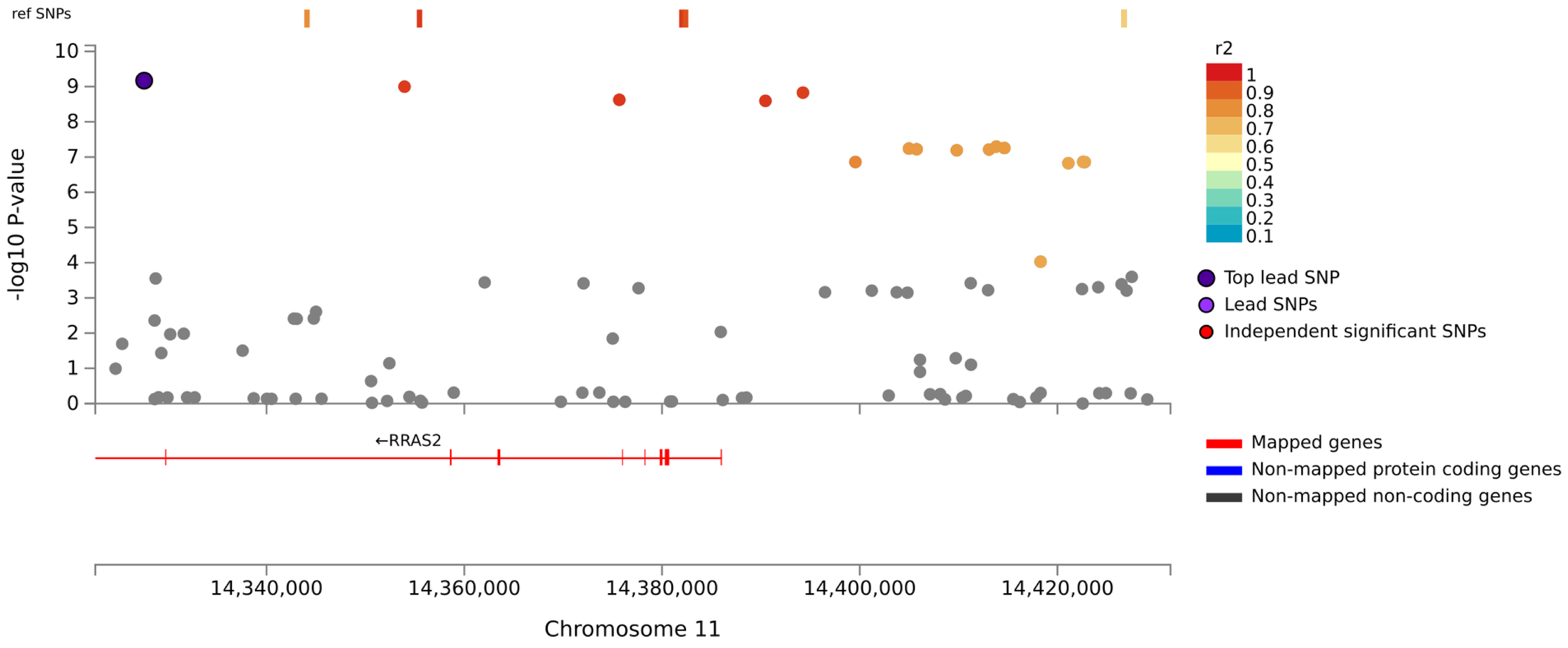

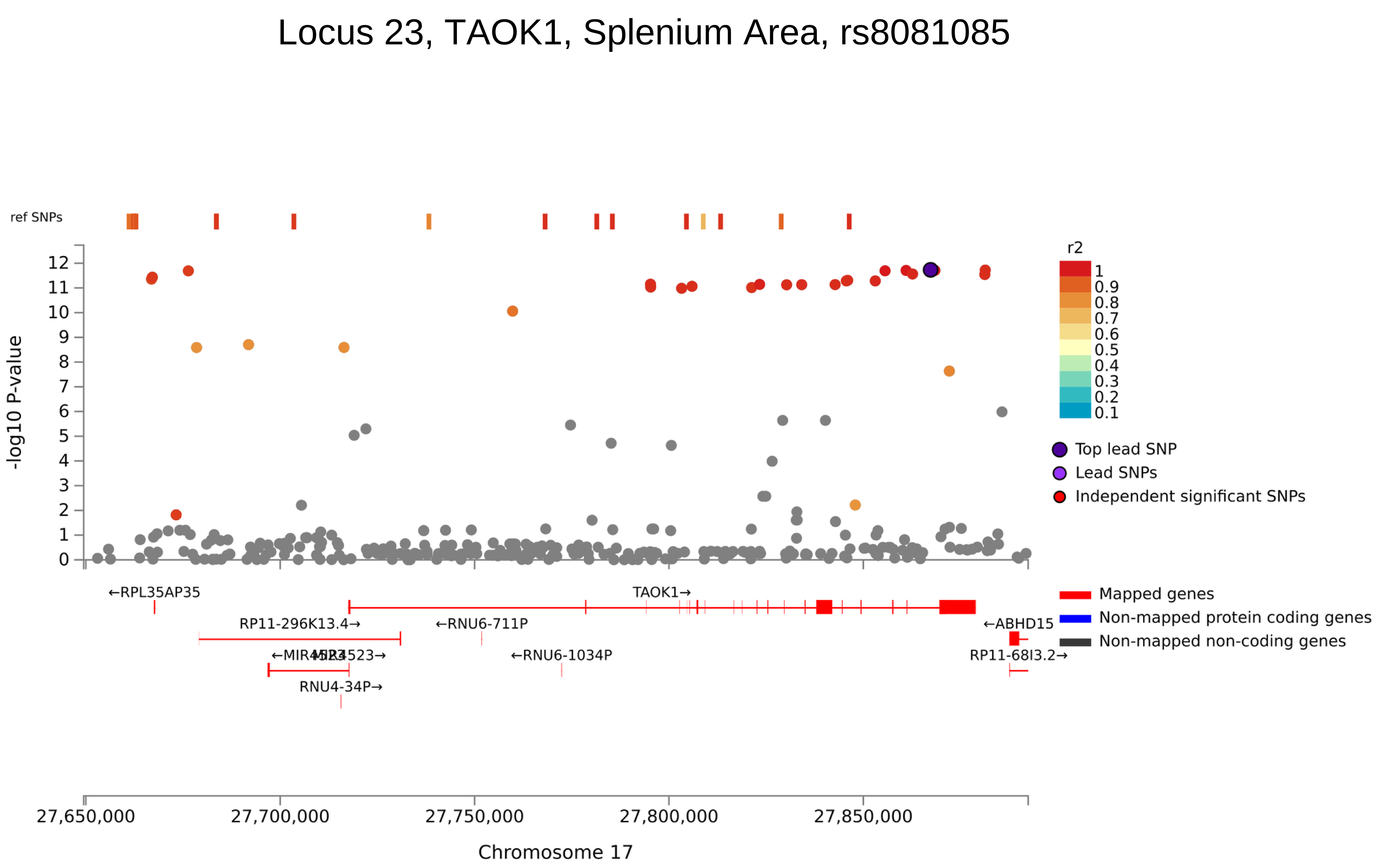
Locus 23, TAOK1, Splenium Area, rs8081085
ref SNPs
-log10 P-value
r2
Top lead SNP
Lead SNPs
Independent significant SNPs
←RPL35AP35
TAOK1→
RP11-296K13.4→
←RNU6-711P
←ABHD15
←RNU6-1034P
RP11-68I3.2→
RNU4-34P→
Mapped genes
Non-mapped protein coding genes
Non-mapped non-coding genes
27,650,000
27,700,000
27,750,000
27,800,000
27,850,000
Chromosome 17

Locus 1, MAST4, Genu Mean Thickness, rs147629510

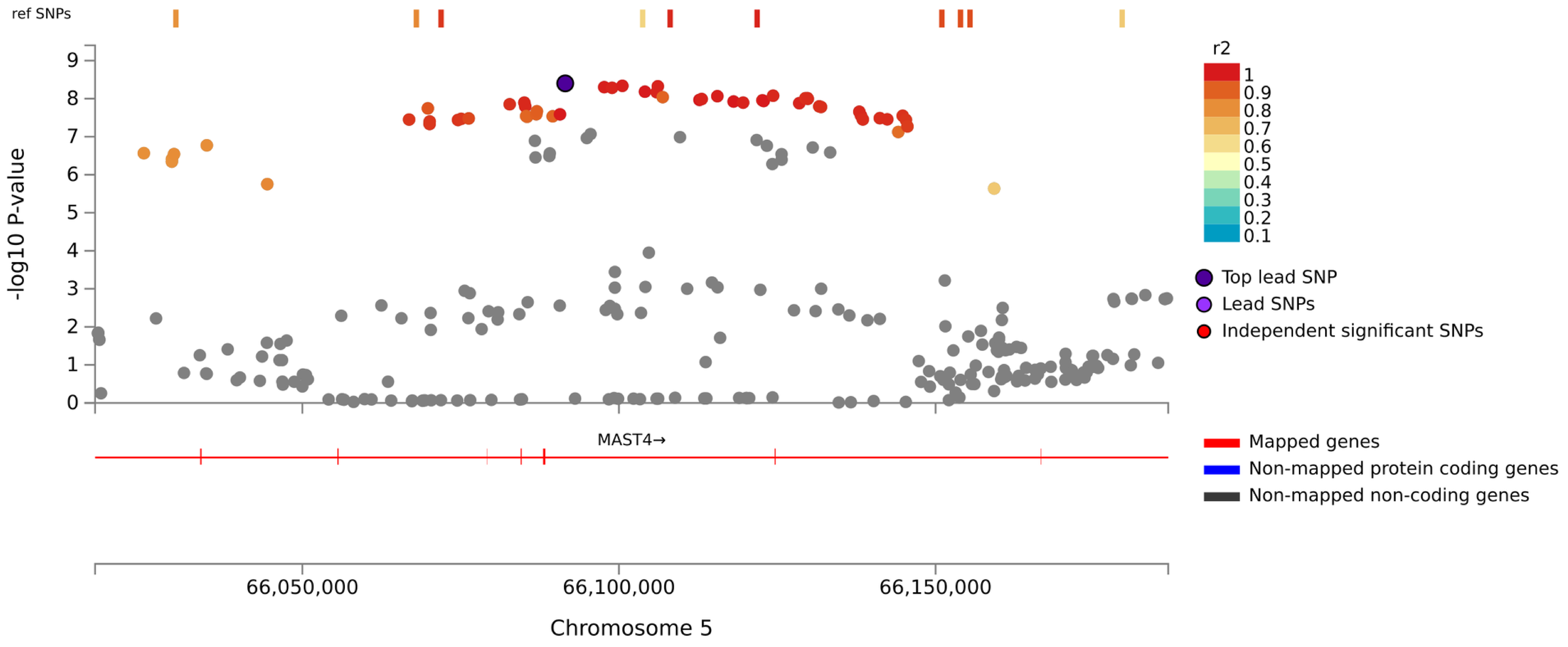

Locus 2, AC003084.2, Genu Mean Thickness, rs916784

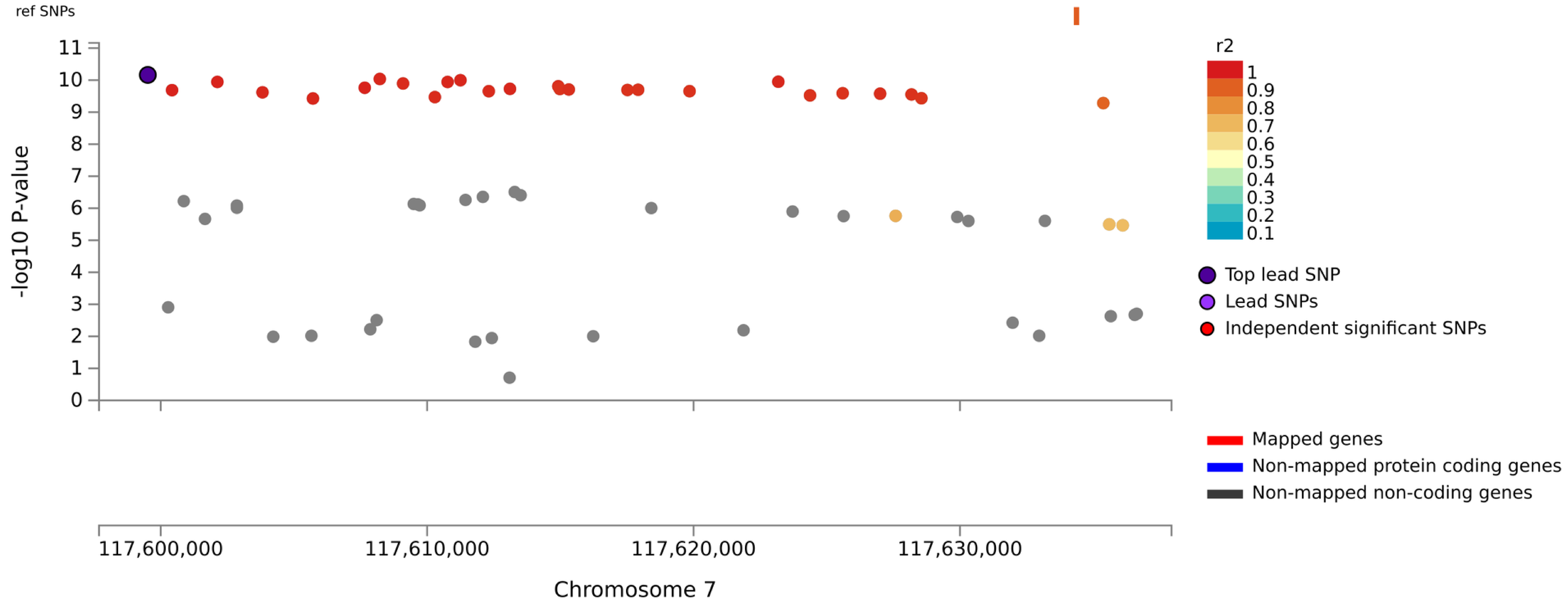

Locus 1, STRN, Anterior Body Mean Thickness, rs7561572

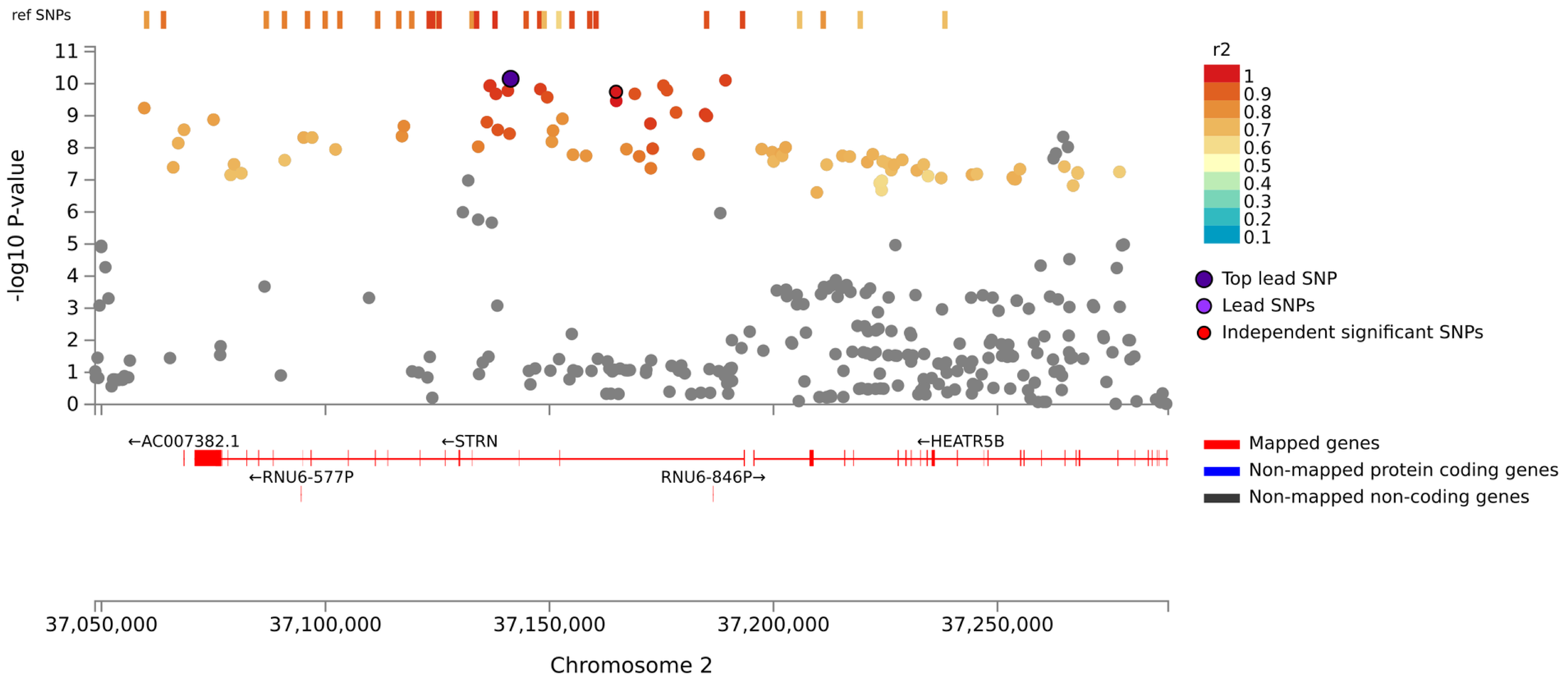

Locus 2, FIP1L1, Anterior Body Mean Thickness, rs1466831

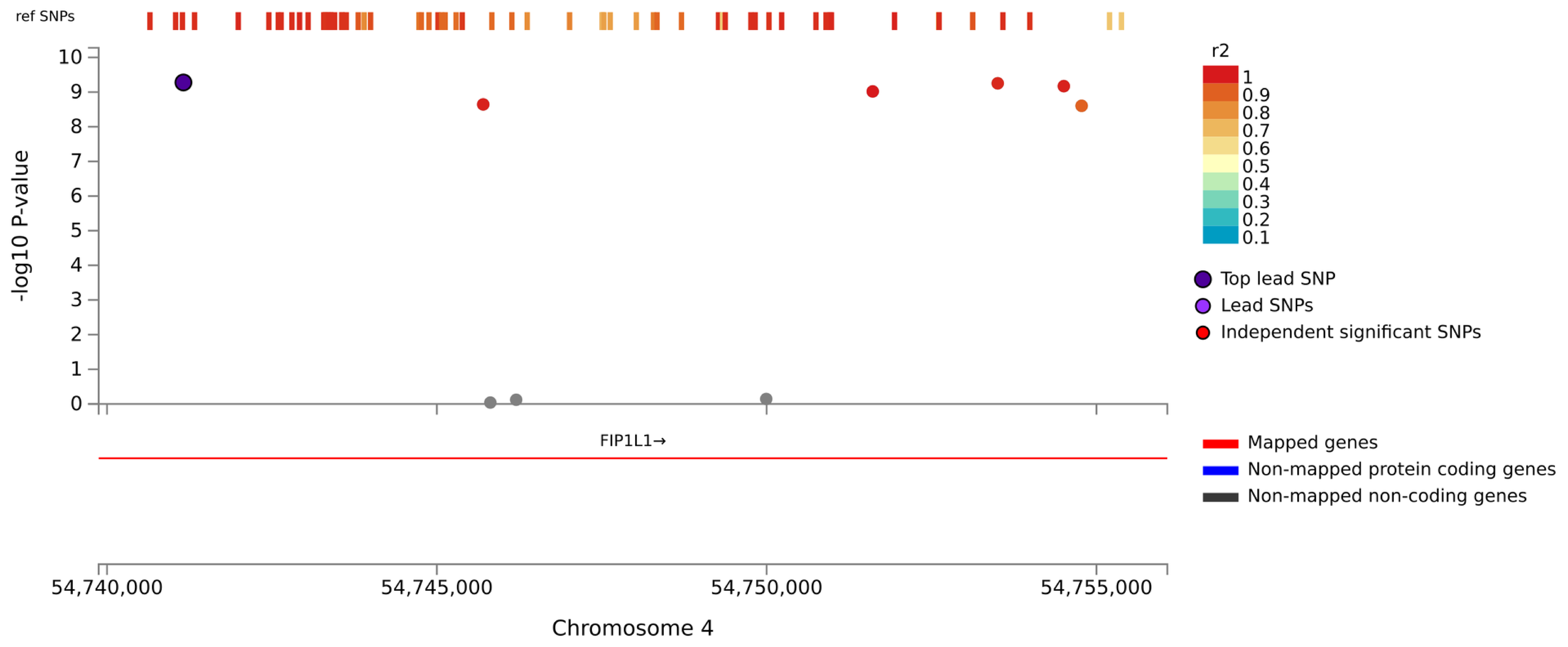

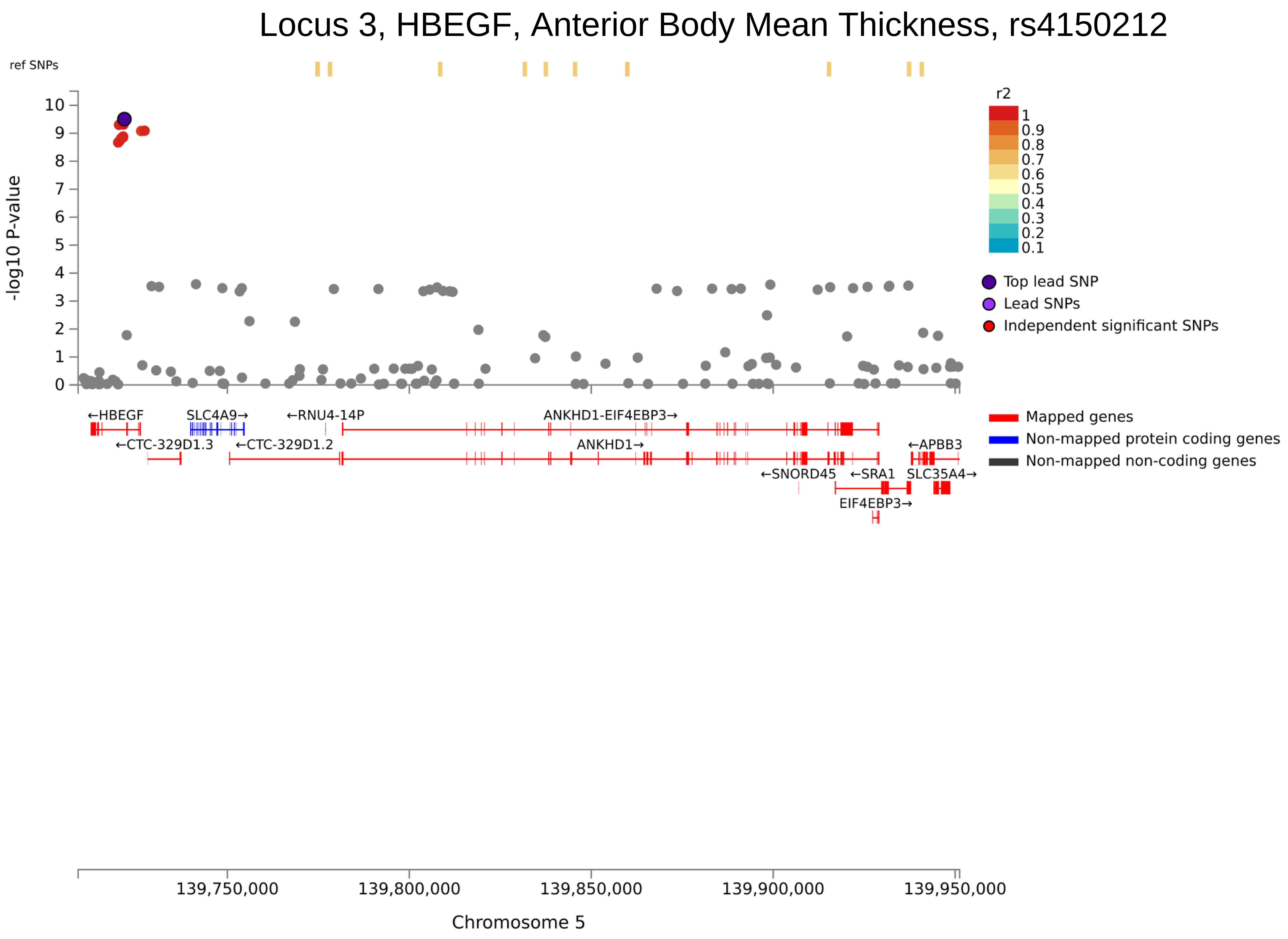

Locus 3, HBEGF, Anterior Body Mean Thickness, rs4150212
ref SNPs
-log10 P-value
r2
Top lead SNP
Lead SNPs
Independent significant SNPs
Mapped genes
Non-mapped protein coding genes
Non-mapped non-coding genes
←HBEGF
SLC4A9→
←RNU4-14P
ANKHD1-EIF4EBP3→
←CTC-329D1.3
←CTC-329D1.2
ANKHD1→
←APBB3
←SNORD45
←SRA1
SLC35A4→
EIF4EBP3→
139,750,000
139,800,000
139,850,000
139,900,000
139,950,000
Chromosome 5

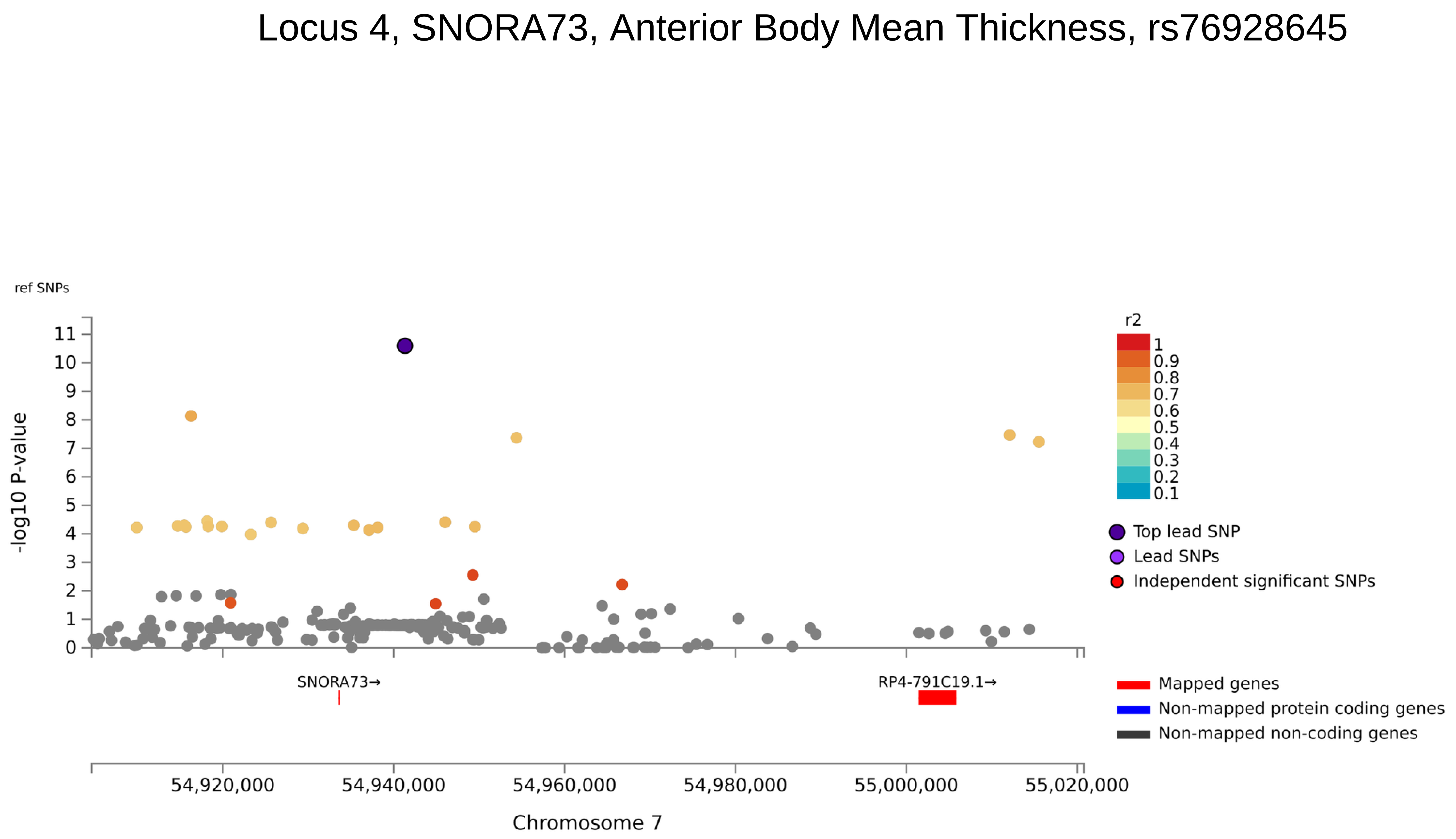

Locus 4, SNORA73, Anterior Body Mean Thickness, rs76928645
ref SNPs
-log10 P-value
r2
Top lead SNP
Lead SNPs
Independent significant SNPs
SNORA73→
RP4-791C19.1→
Mapped genes
Non-mapped protein coding genes
Non-mapped non-coding genes
54,920,000
54,940,000
54,960,000
54,980,000
55,000,000
55,020,000
Chromosome 7

Locus 5, PLEC, Anterior Body Mean Thickness, rs7464572

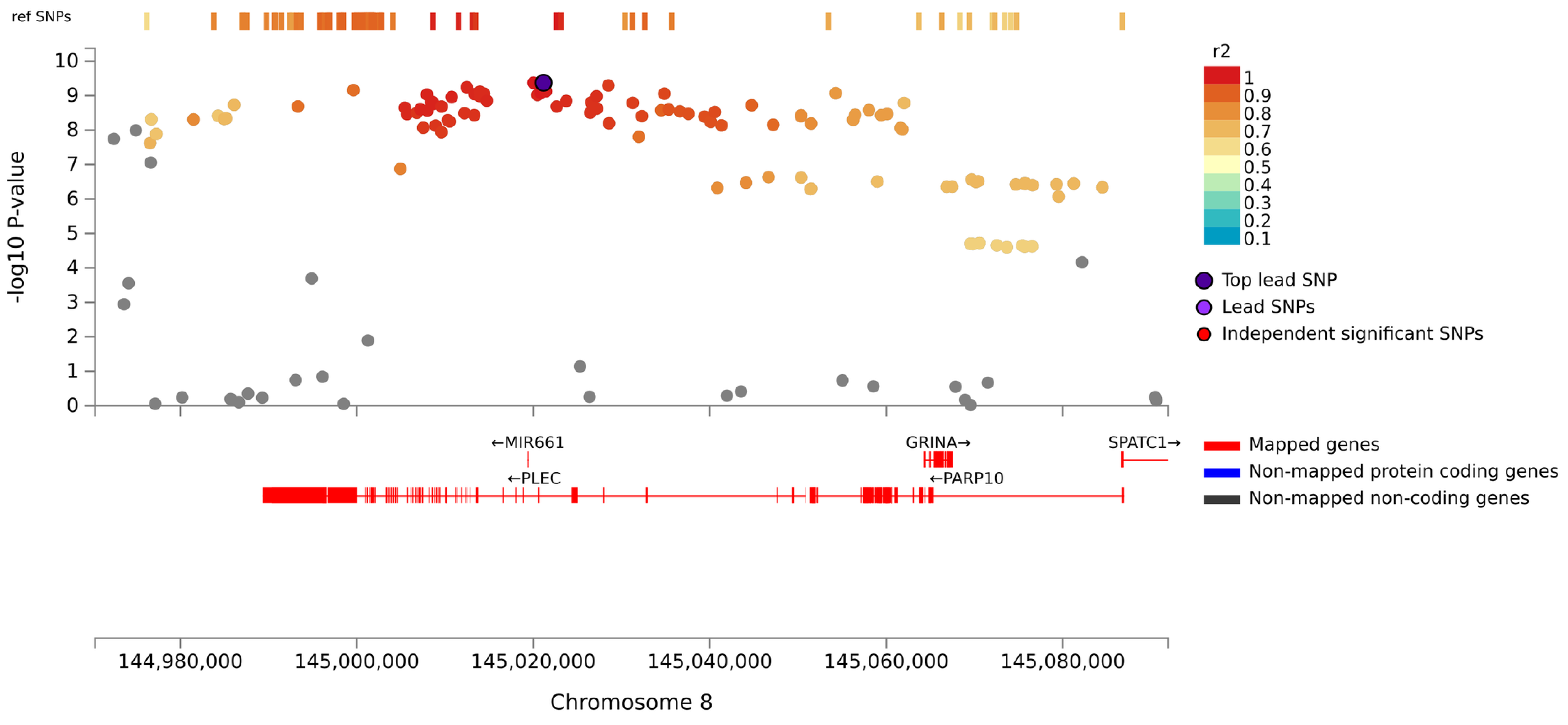

Locus 6, CABYR, Anterior Body Mean Thickness, rs752797

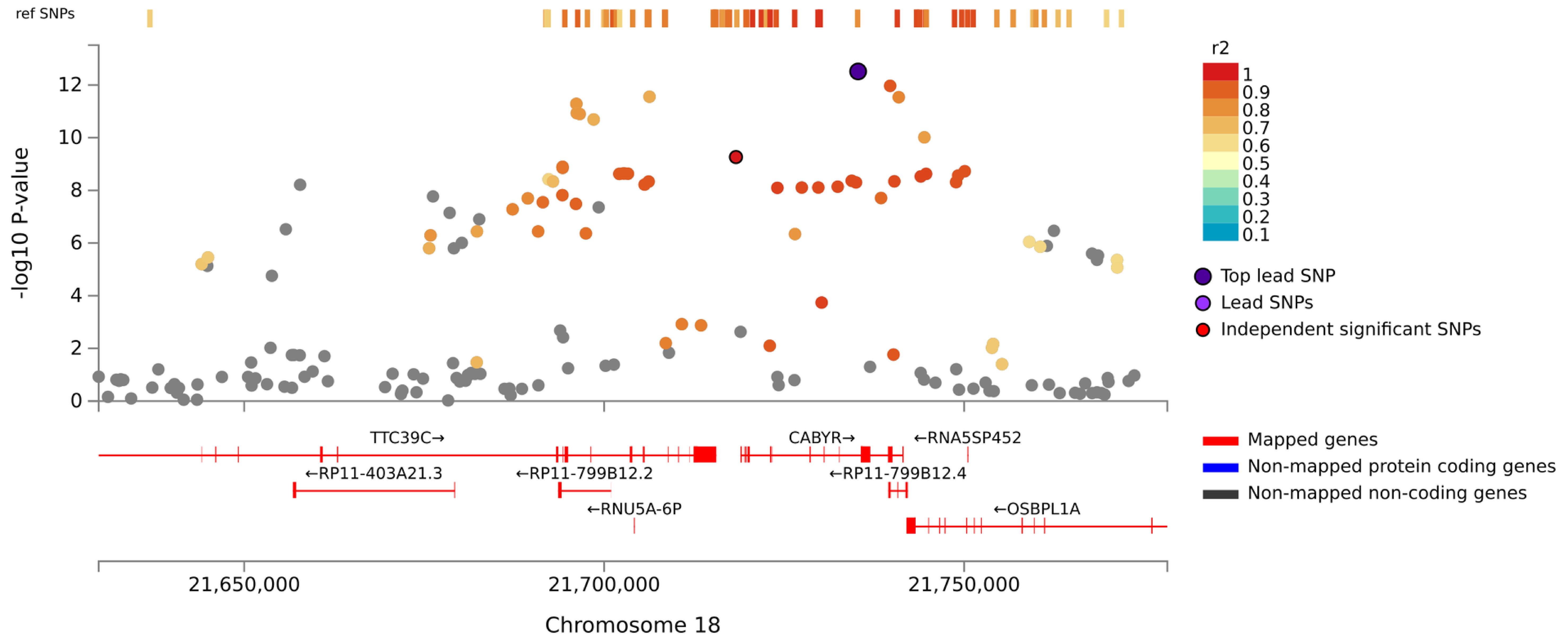

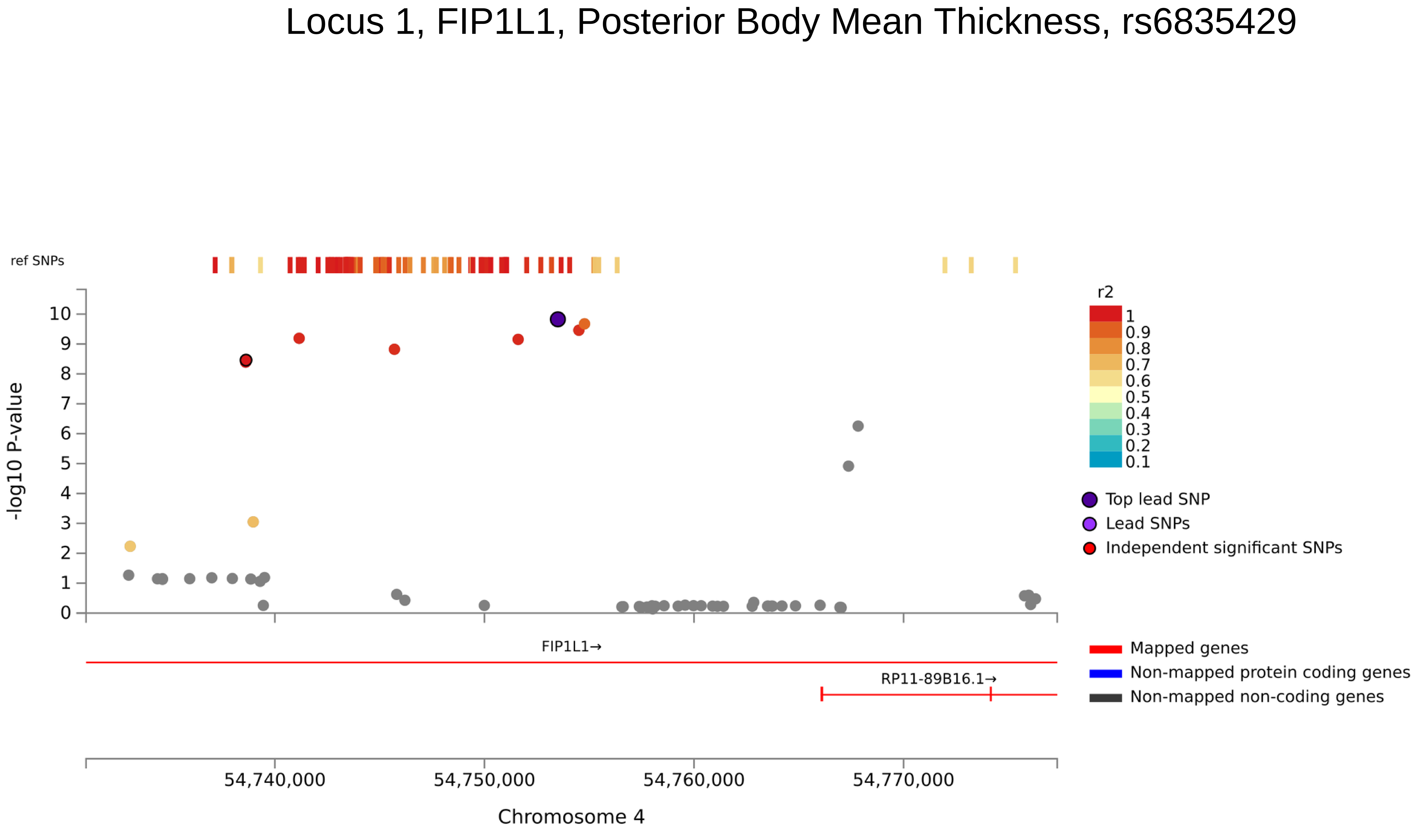
Locus 1, FIP1L1, Posterior Body Mean Thickness, rs6835429
ref SNPs
-log10 P-value
r2
1
0.9
0.8
0.7
0.6
0.5
0.4
0.3
0.2
0.1
Top lead SNP
Lead SNPs
Independent significant SNPs
FIP1L1→
RP11-89B16.1→
Mapped genes
Non-mapped protein coding genes
Non-mapped non-coding genes
54,740,000
54,750,000
54,760,000
54,770,000
Chromosome 4

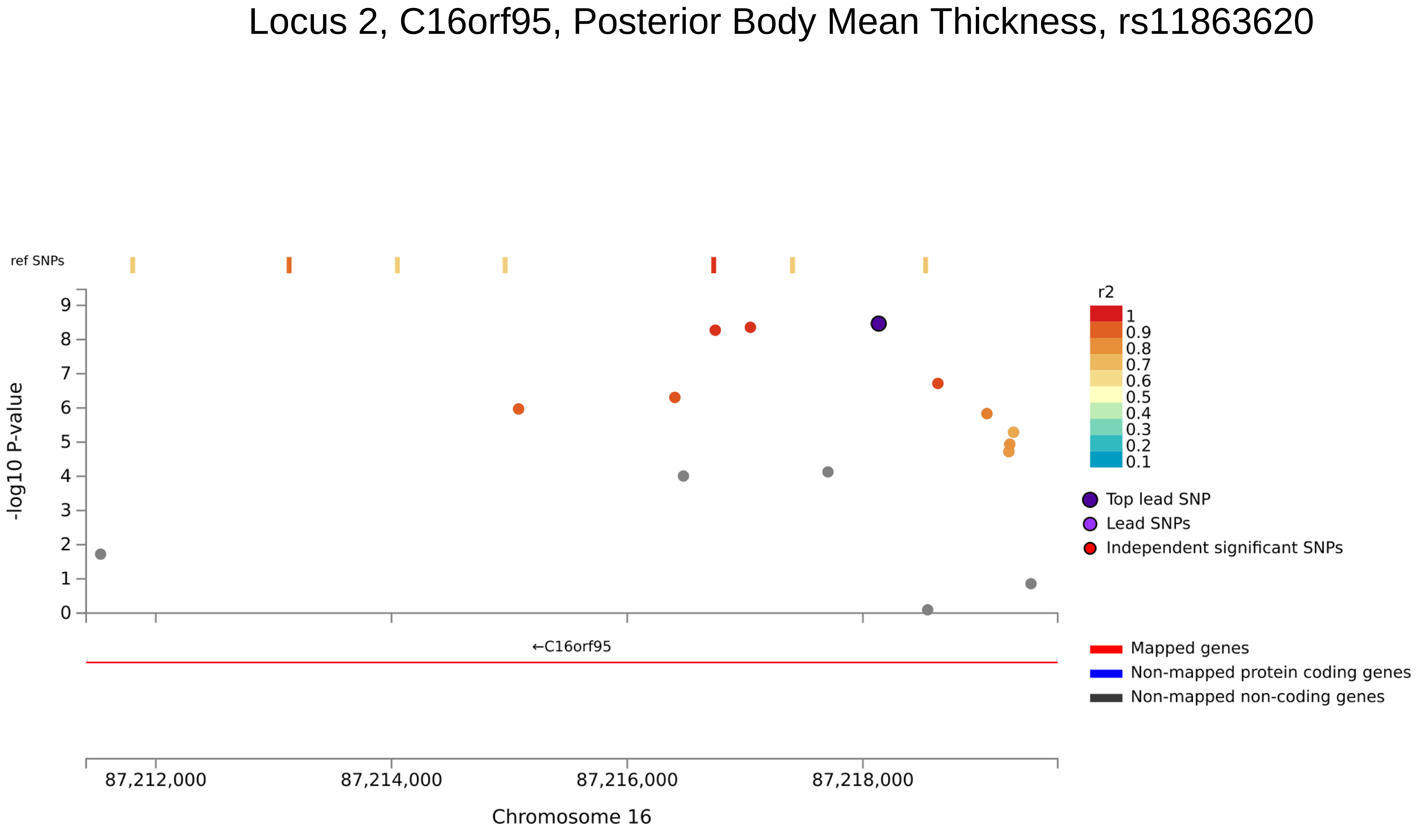
Locus 2, C16orf95, Posterior Body Mean Thickness, rs11863620
ref SNPs
-log10 P-value
r2
1
0.9
0.8
0.7
0.6
0.5
0.4
0.3
0.2
0.1
Top lead SNP
Lead SNPs
Independent significant SNPs
←C16orf95
Mapped genes
Non-mapped protein coding genes
Non-mapped non-coding genes
87,212,000
87,214,000
87,216,000
87,218,000
Chromosome 16

Locus 3, TTC39C, Posterior Body Mean Thickness, rs12967609

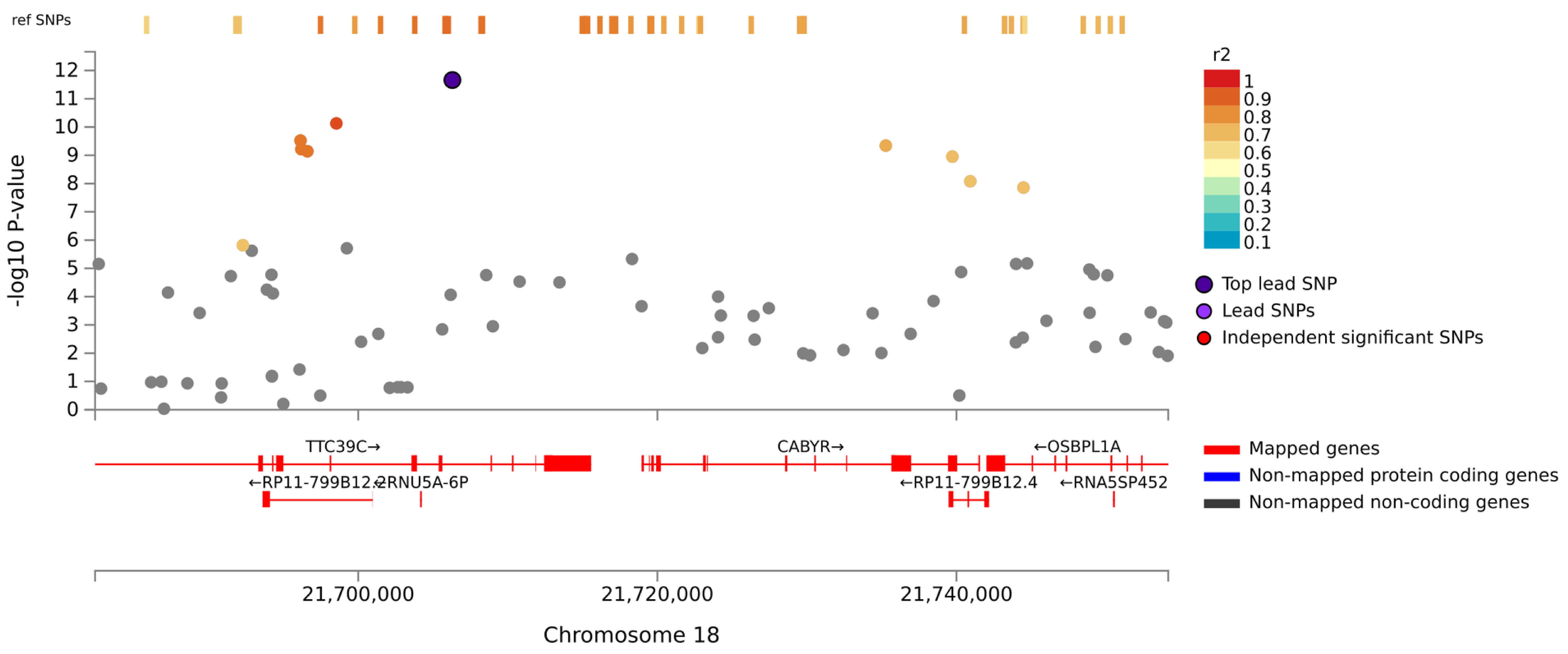

Locus 1, RP1-37C10.3, Isthmus Mean Thickness, rs6682671

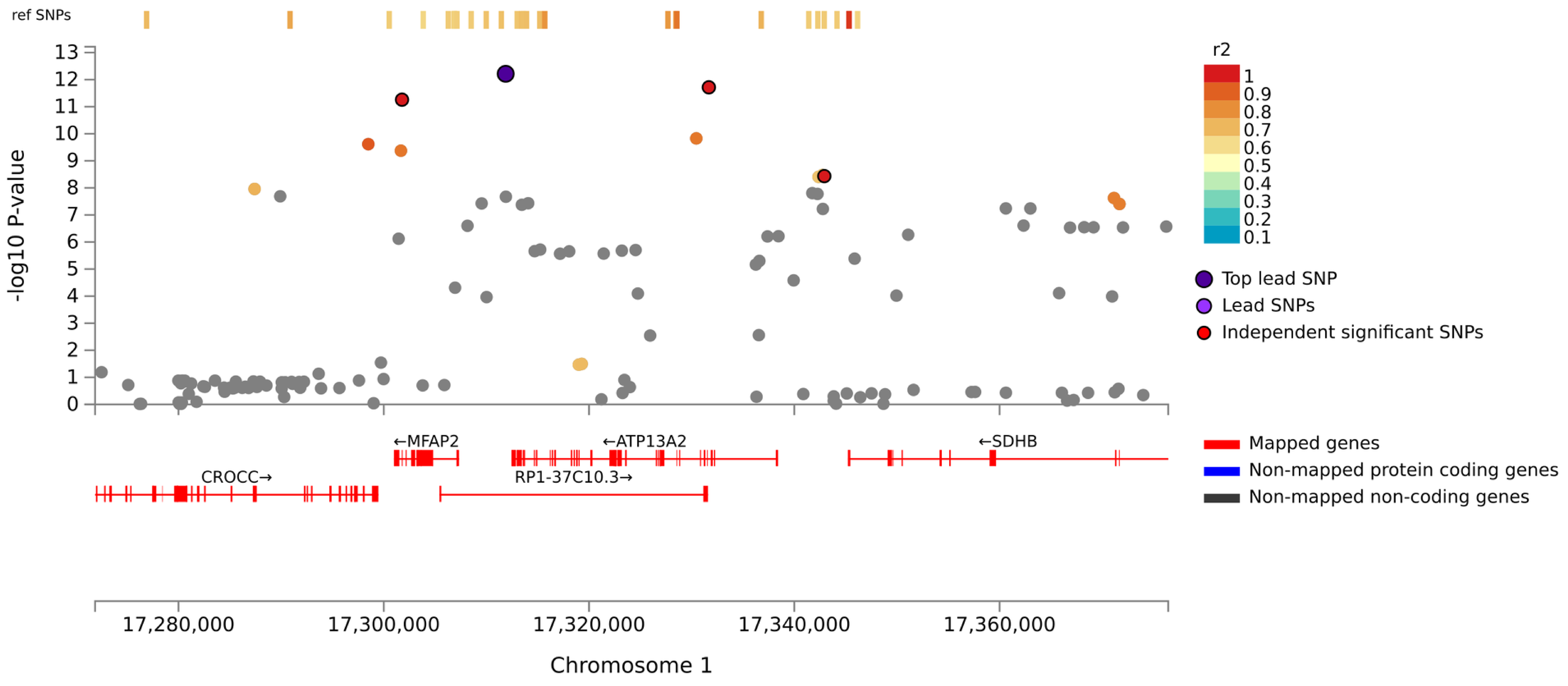

Locus 2, STRN, Isthmus Mean Thickness, rs7561572

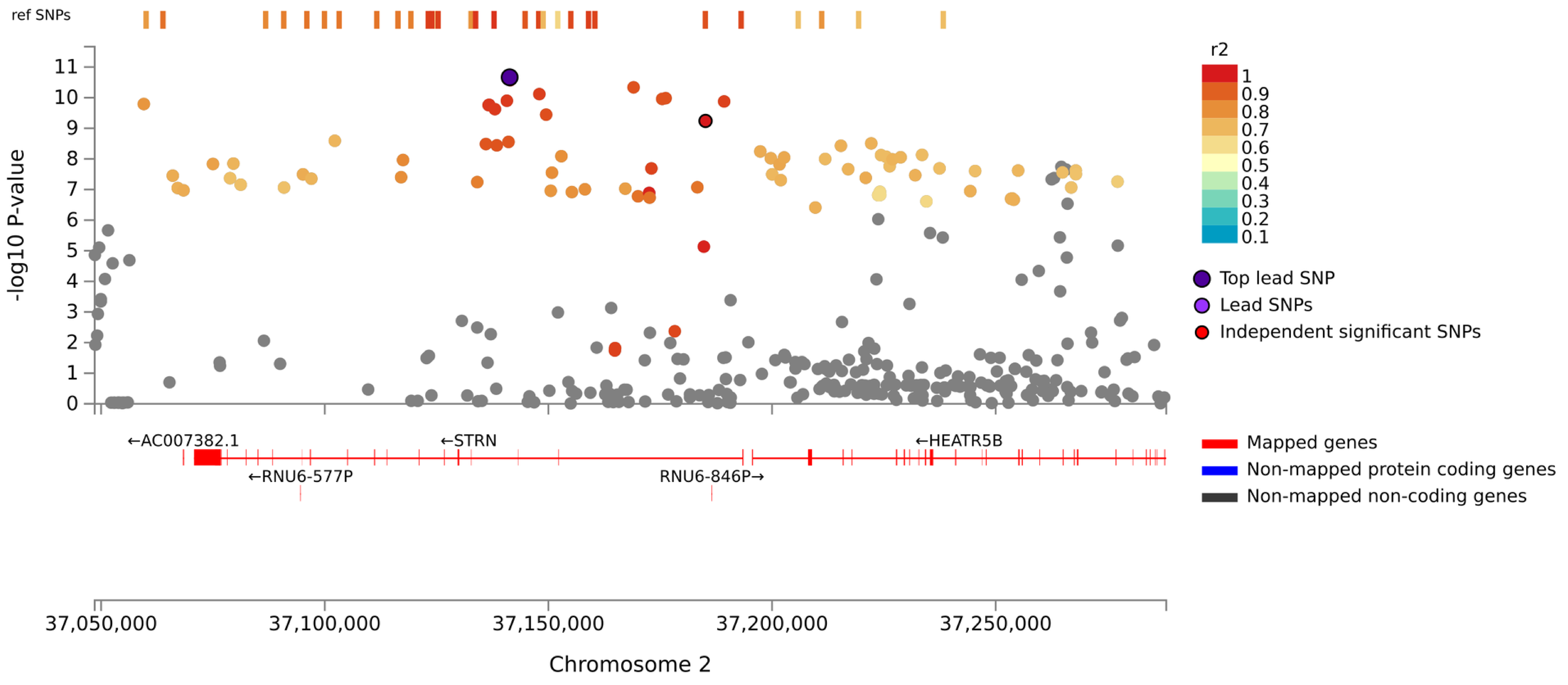

Locus 3, CCDC75P1, Isthmus Mean Thickness, rs9857083

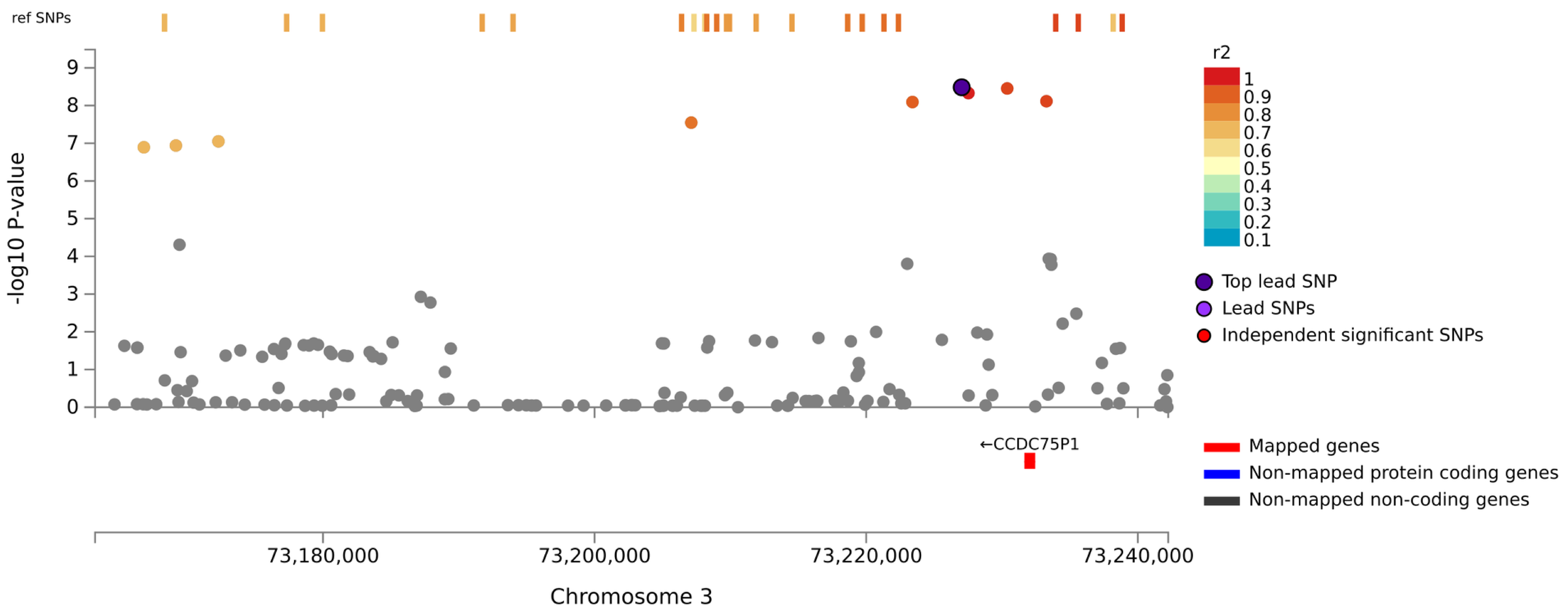

Locus 4, FIP1L1, Isthmus Mean Thickness, rs6554139

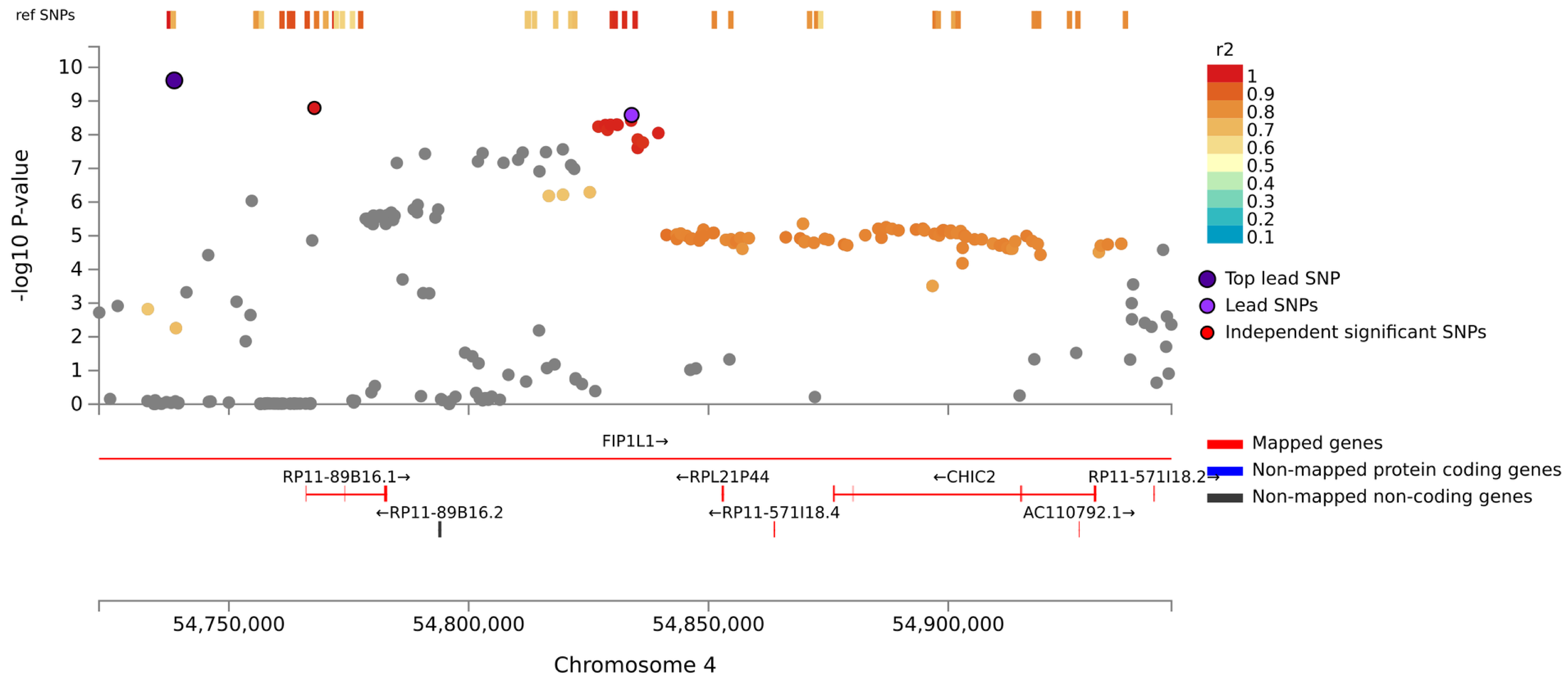

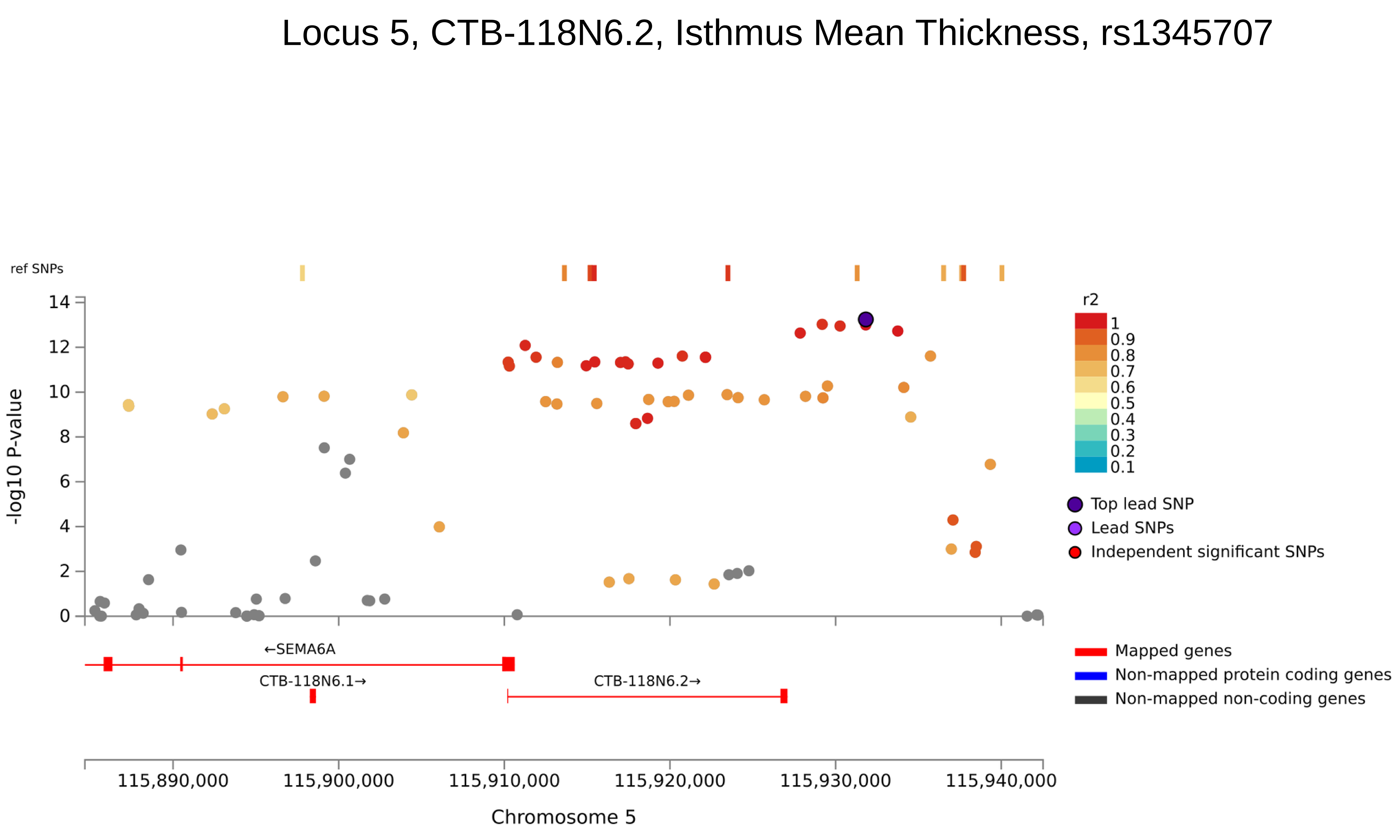

Locus 5, CTB-118N6.2, Isthmus Mean Thickness, rs1345707
ref SNPs
-log10 P-value
r2
Top lead SNP
Lead SNPs
Independent significant SNPs
←SEMA6A
CTB-118N6.1→
CTB-118N6.2→
Mapped genes
Non-mapped protein coding genes
Non-mapped non-coding genes
115,890,000
115,900,000
115,910,000
115,920,000
115,930,000
115,940,000
Chromosome 5

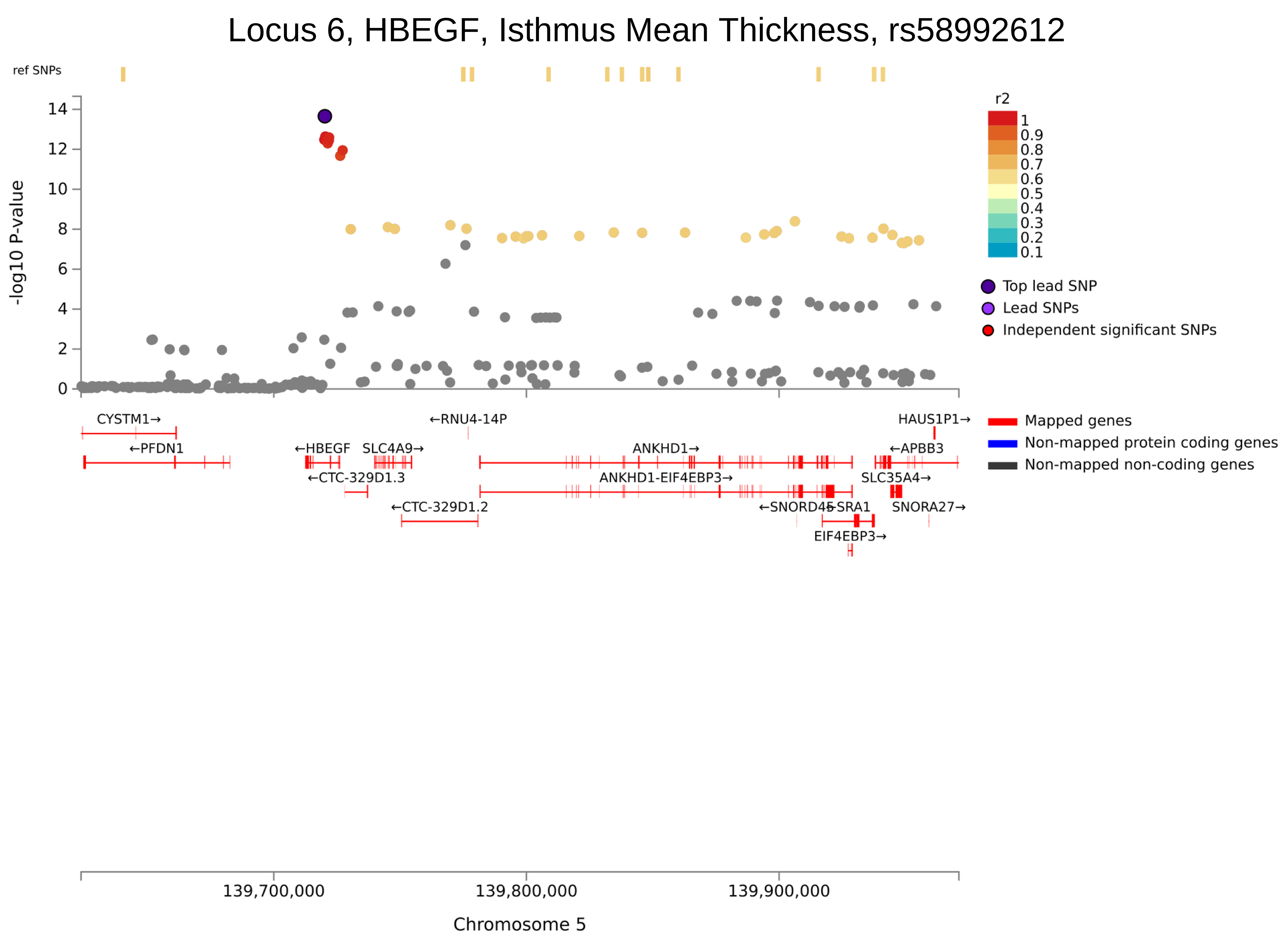

Locus 6, HBEGF, Isthmus Mean Thickness, rs58992612
ref SNPs
-log10 P-value
r2
Top lead SNP
Lead SNPs
Independent significant SNPs
CYSTM1→
←PFDN1
←HBEGF
SLC4A9→
←CTC-329D1.3
←RNU4-14P
←CTC-329D1.2
ANKHD1→
ANKHD1-EIF4EBP3→
←SNORD45
SRA1
EIF4EBP3→
HAUS1P1→
←APBB3
SLC35A4→
SNORA27→
Mapped genes
Non-mapped protein coding genes
Non-mapped non-coding genes
139,700,000
139,800,000
139,900,000
Chromosome 5

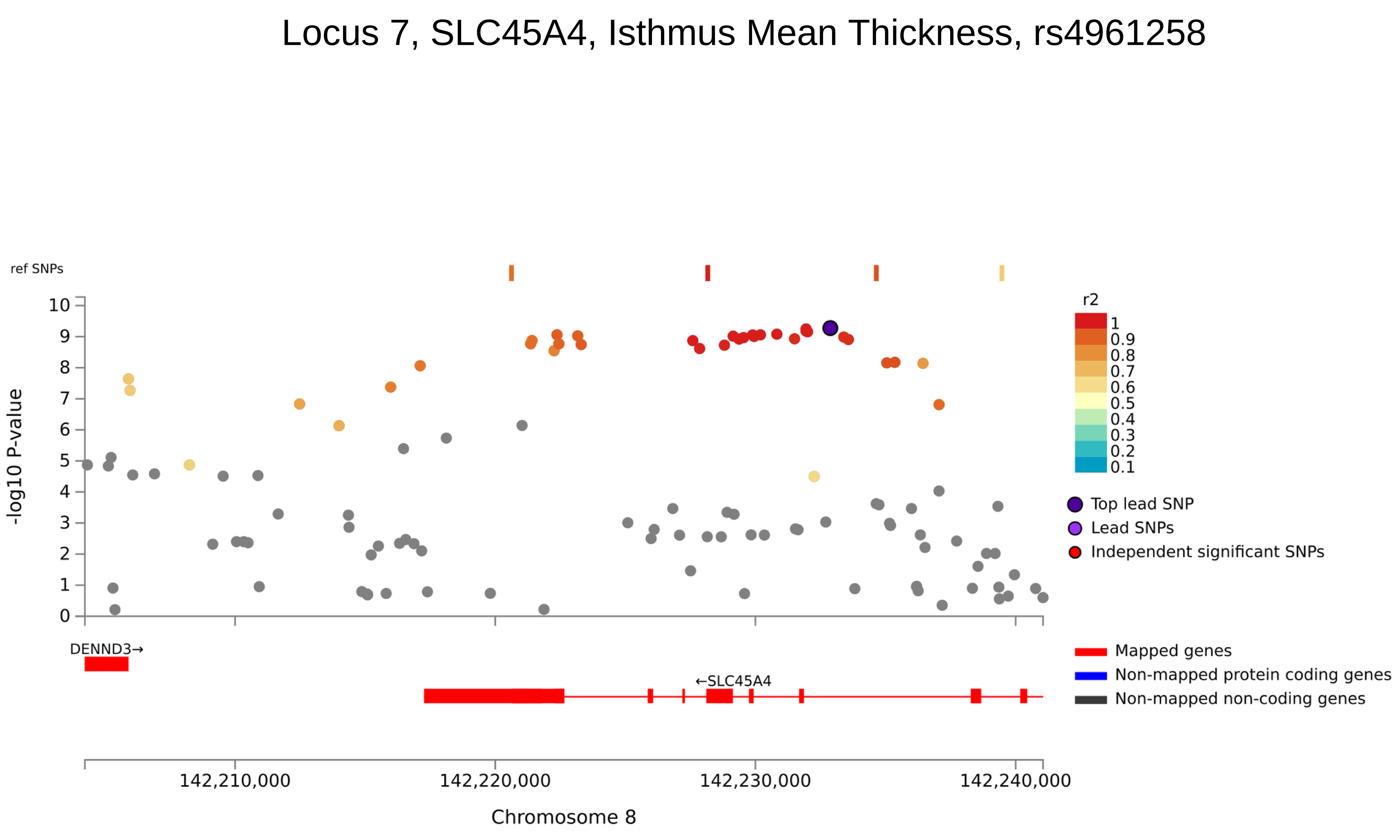
Locus 7, SLC45A4, Isthmus Mean Thickness, rs4961258
ref SNPs
-log10 P-value
r2
Top lead SNP
Lead SNPs
Independent significant SNPs
DENND3→
←SLC45A4
Mapped genes
Non-mapped protein coding genes
Non-mapped non-coding genes
142,210,000
142,220,000
142,230,000
142,240,000
Chromosome 8

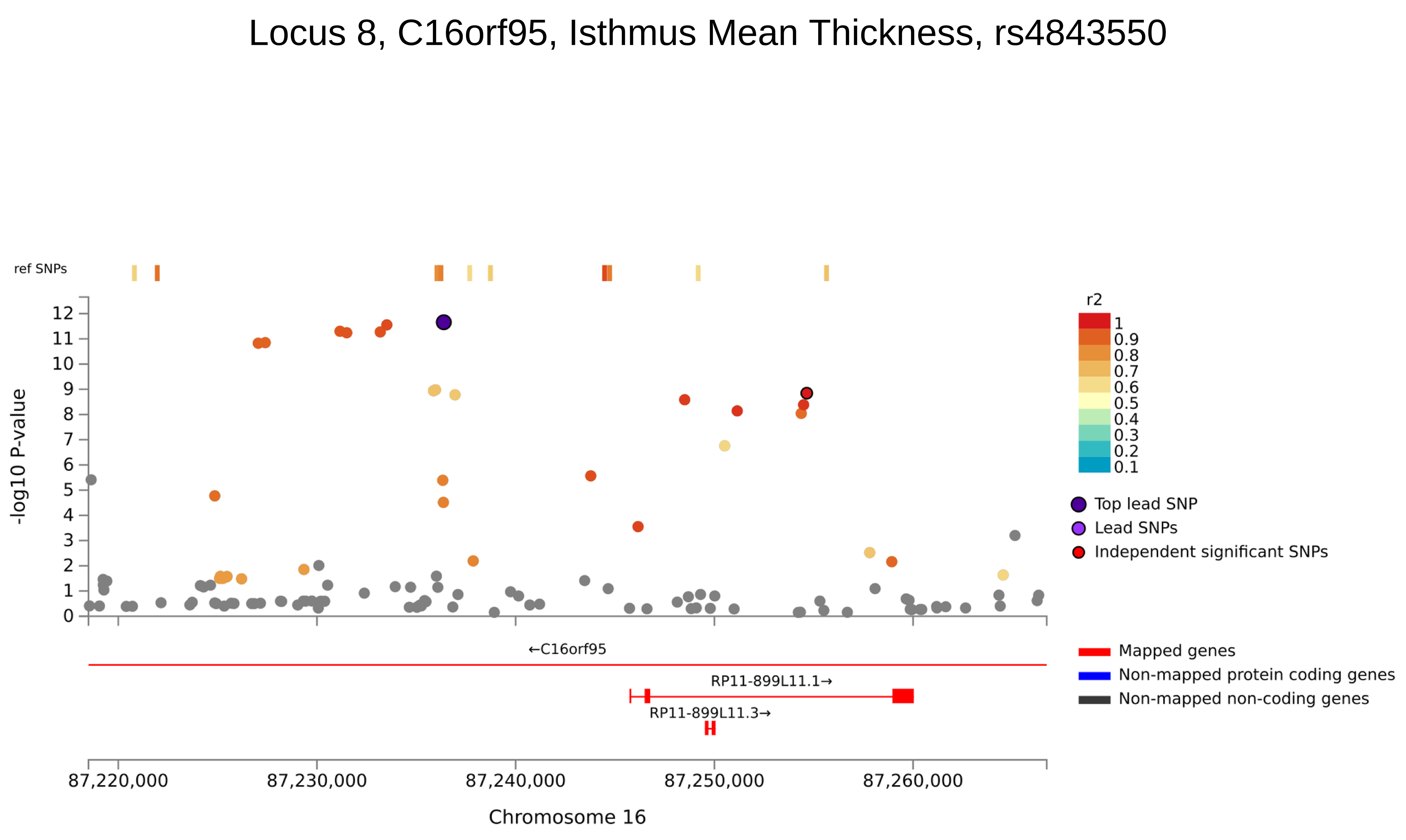
Locus 8, C16orf95, Isthmus Mean Thickness, rs4843550
ref SNPs
-log10 P-value
r2
Top lead SNP
Lead SNPs
Independent significant SNPs
←C16orf95
RP11-899L11.1→
RP11-899L11.3→
Mapped genes
Non-mapped protein coding genes
Non-mapped non-coding genes
87,220,000
87,230,000
87,240,000
87,250,000
87,260,000
Chromosome 16

Locus 1, STRN, Splenium Mean Thickness, rs62132522

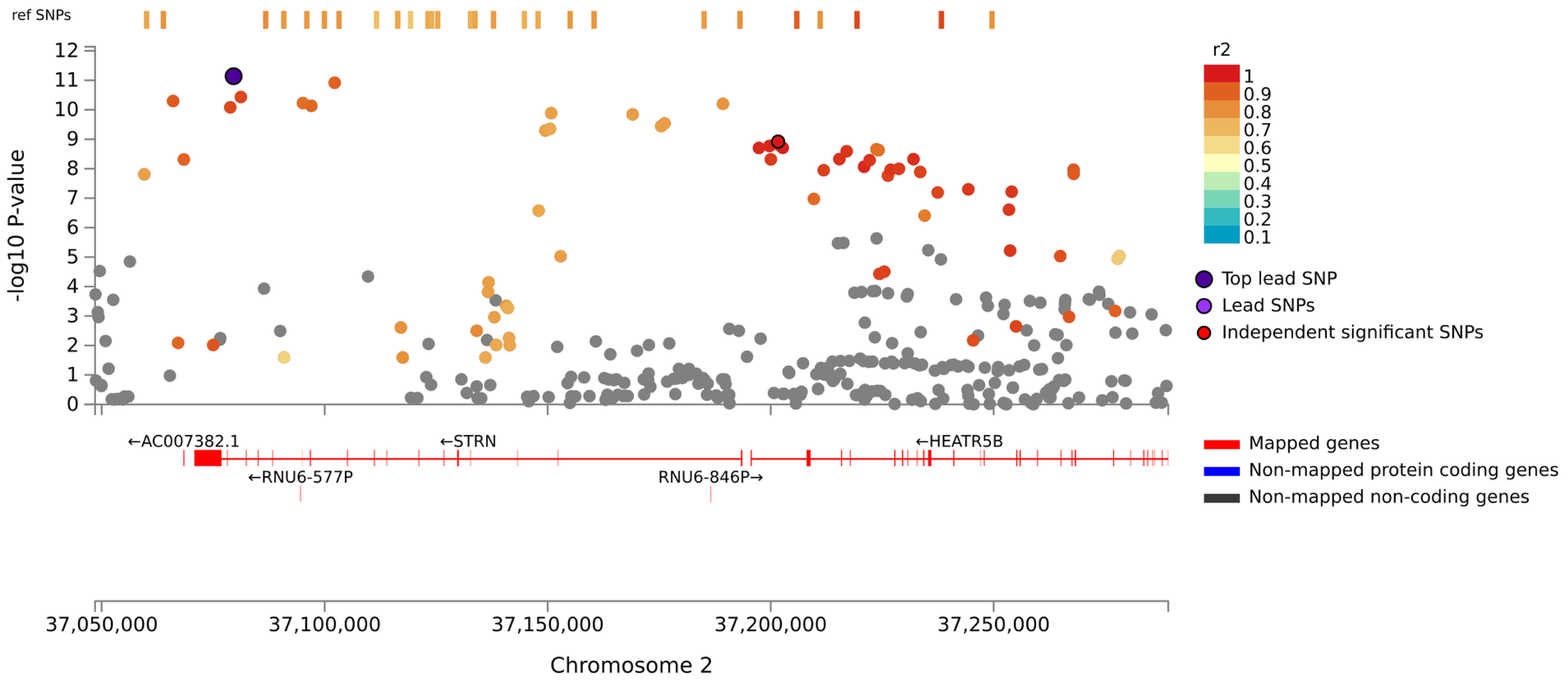

Locus 2, IQCJ-SCHIP1:IQCJ, Splenium Mean Thickness, rs55938743

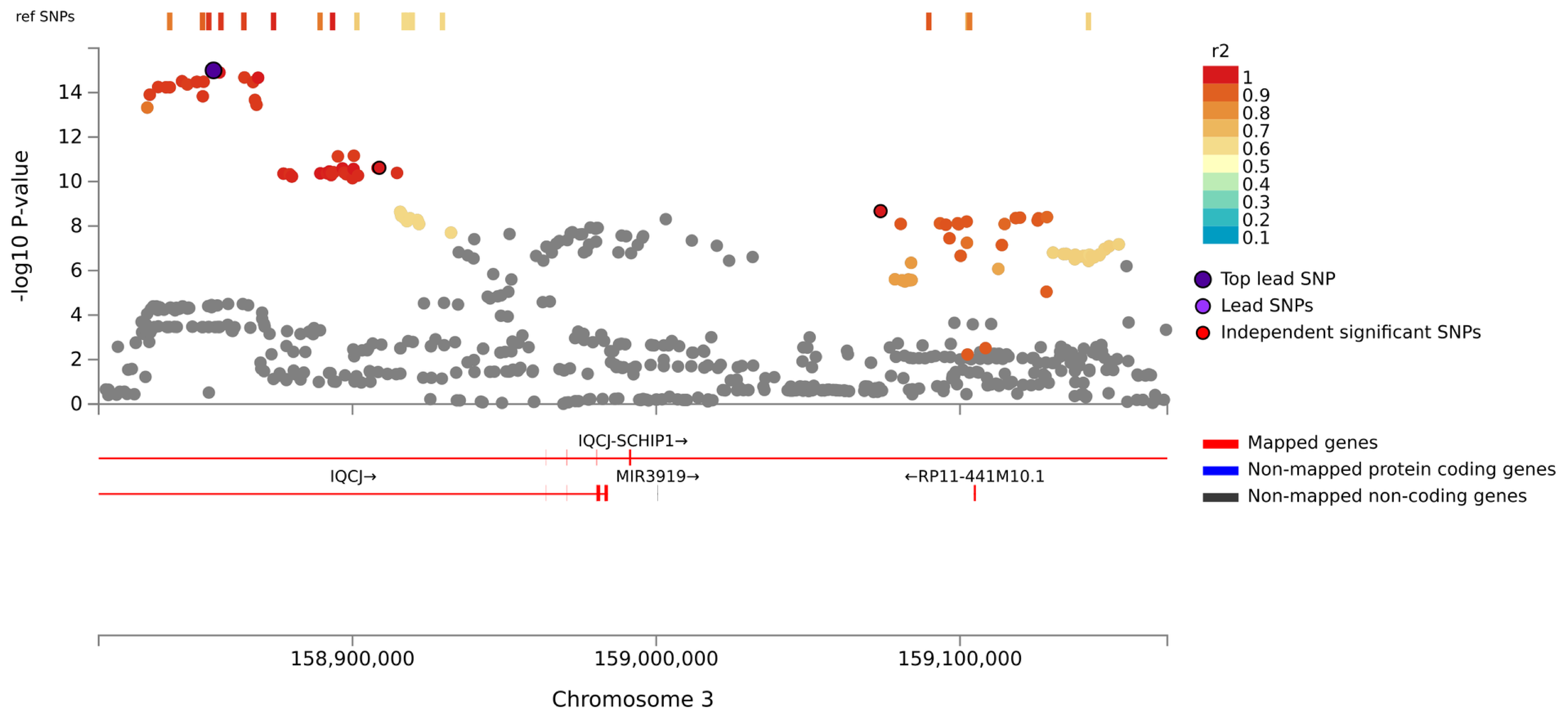

Locus 3, TBC1D14, Splenium Mean Thickness, rs11938701

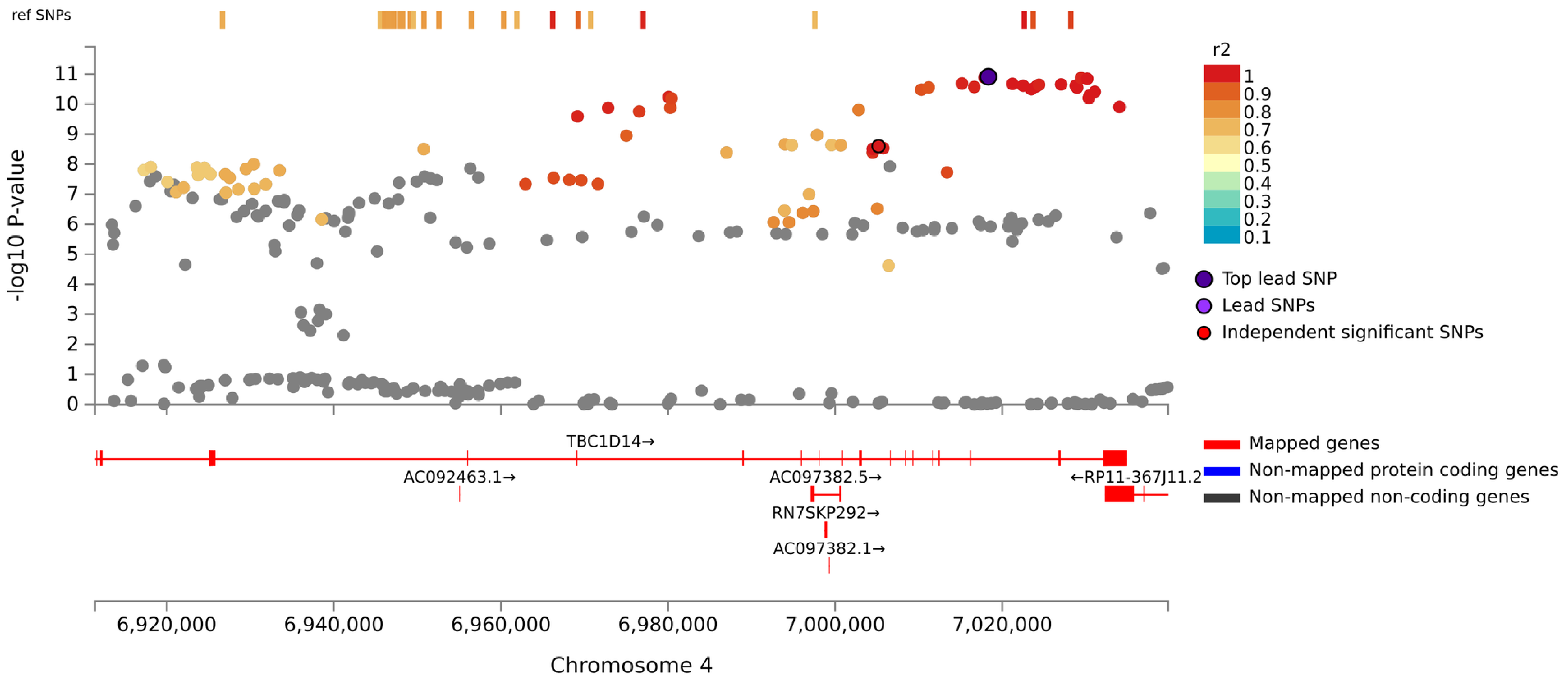

Locus 4, FIP1L1, Splenium Mean Thickness, rs6835429

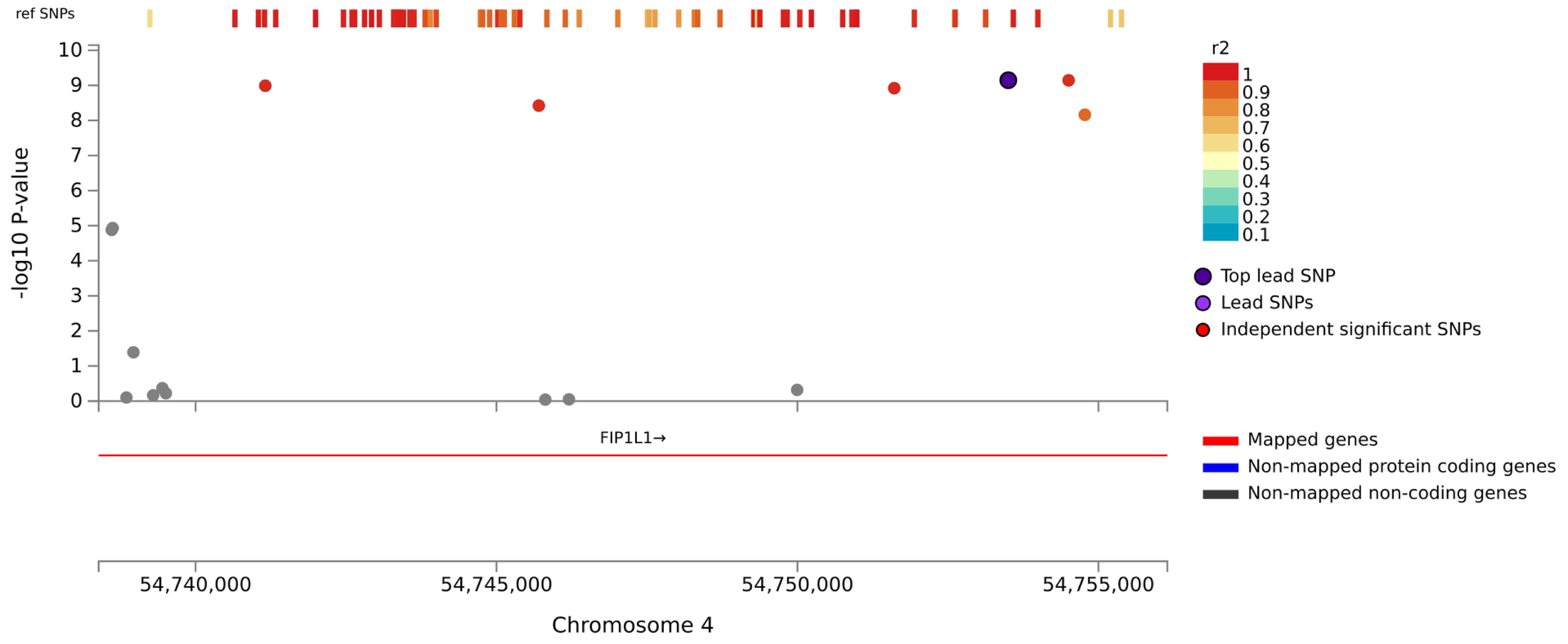

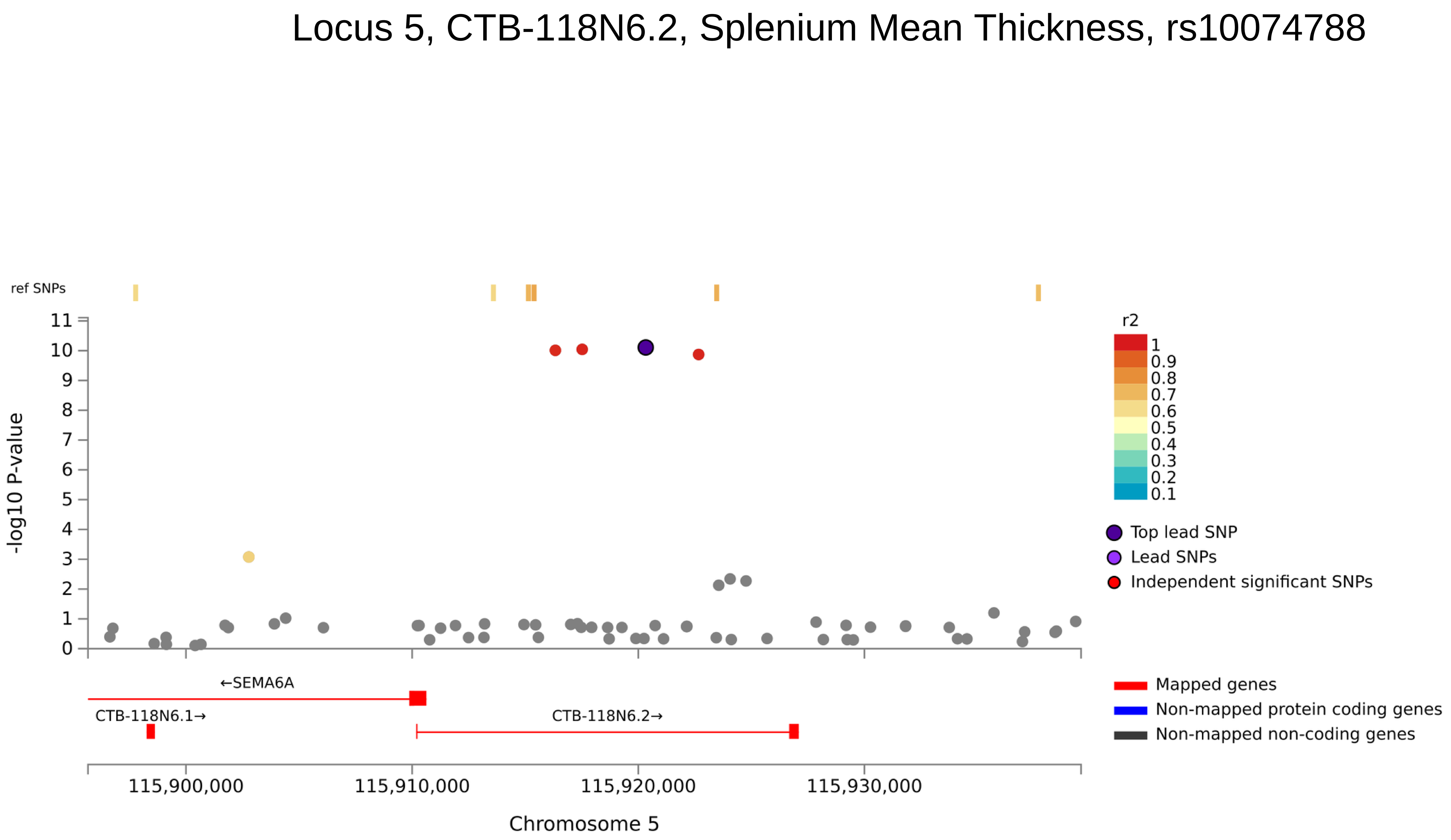
Locus 5, CTB-118N6.2, Splenium Mean Thickness, rs10074788
ref SNPs
-log10 P-value
r2
Top lead SNP
Lead SNPs
Independent significant SNPs
←SEMA6A
CTB-118N6.1→
CTB-118N6.2→
Mapped genes
Non-mapped protein coding genes
Non-mapped non-coding genes
115,900,000
115,910,000
115,920,000
115,930,000
Chromosome 5

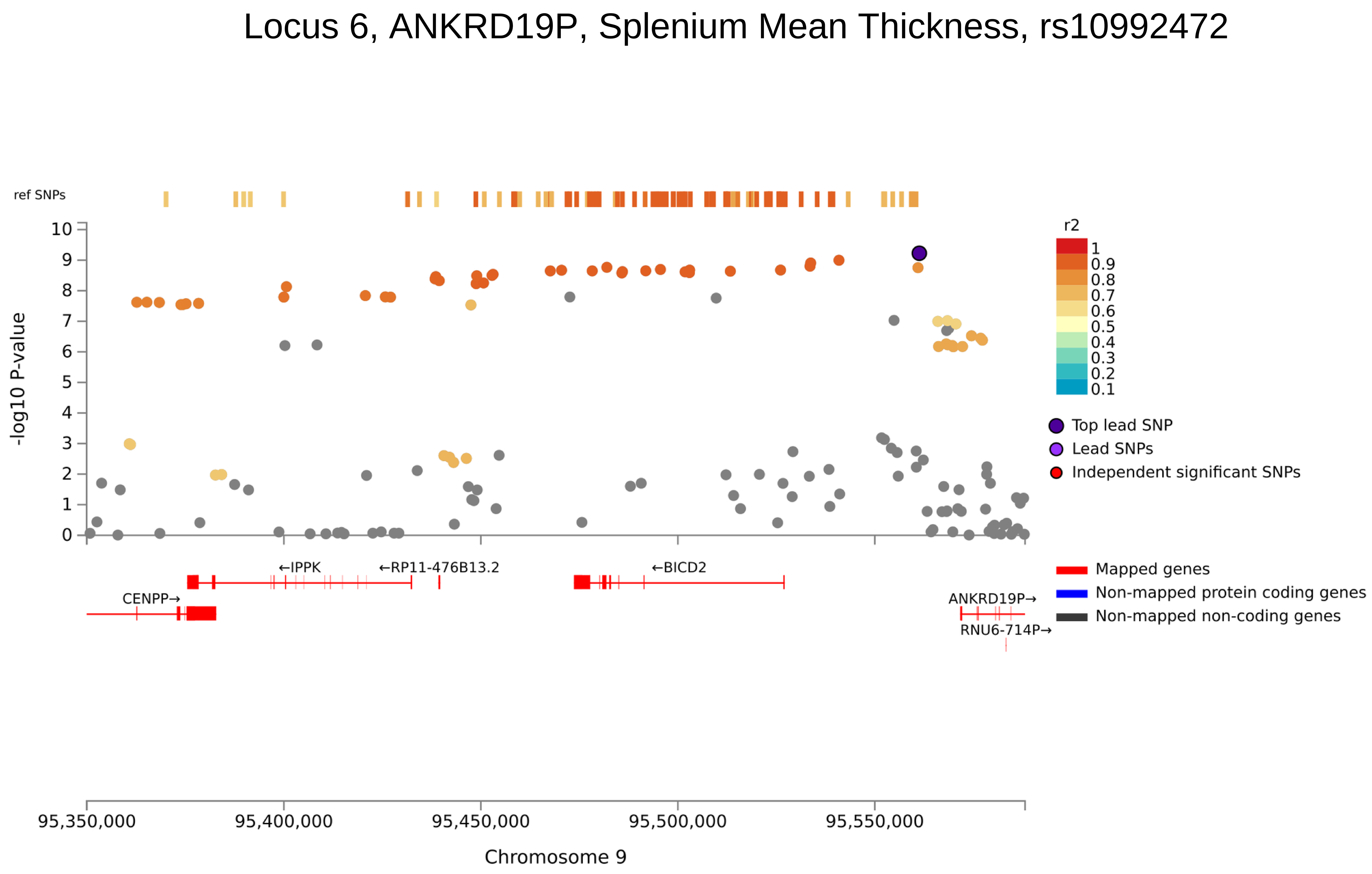
Locus 6, ANKRD19P, Splenium Mean Thickness, rs10992472
ref SNPs
-log10 P-value
r2
Top lead SNP
Lead SNPs
Independent significant SNPs
←IPPK
←RP11-476B13.2
←BICD2
CENPP→
ANKRD19P→
RNU6-714P→
Mapped genes
Non-mapped protein coding genes
Non-mapped non-coding genes
95,350,000
95,400,000
95,450,000
95,500,000
95,550,000
Chromosome 9

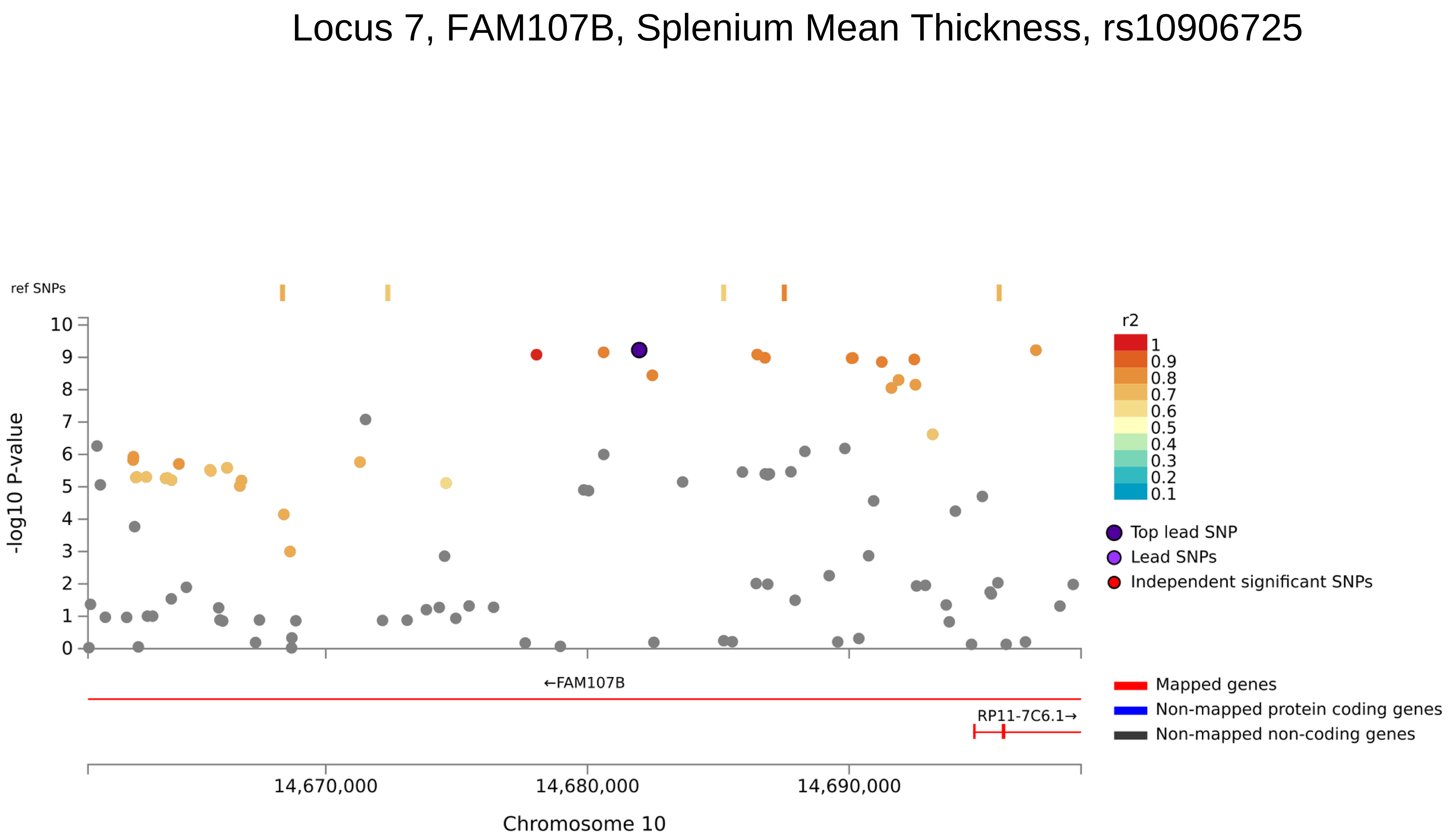
Locus 7, FAM107B, Splenium Mean Thickness, rs10906725
ref SNPs
-log10 P-value
r2
Top lead SNP
Lead SNPs
Independent significant SNPs
←FAM107B
RP11-7C6.1→
Mapped genes
Non-mapped protein coding genes
Non-mapped non-coding genes
14,670,000
14,680,000
14,690,000
Chromosome 10

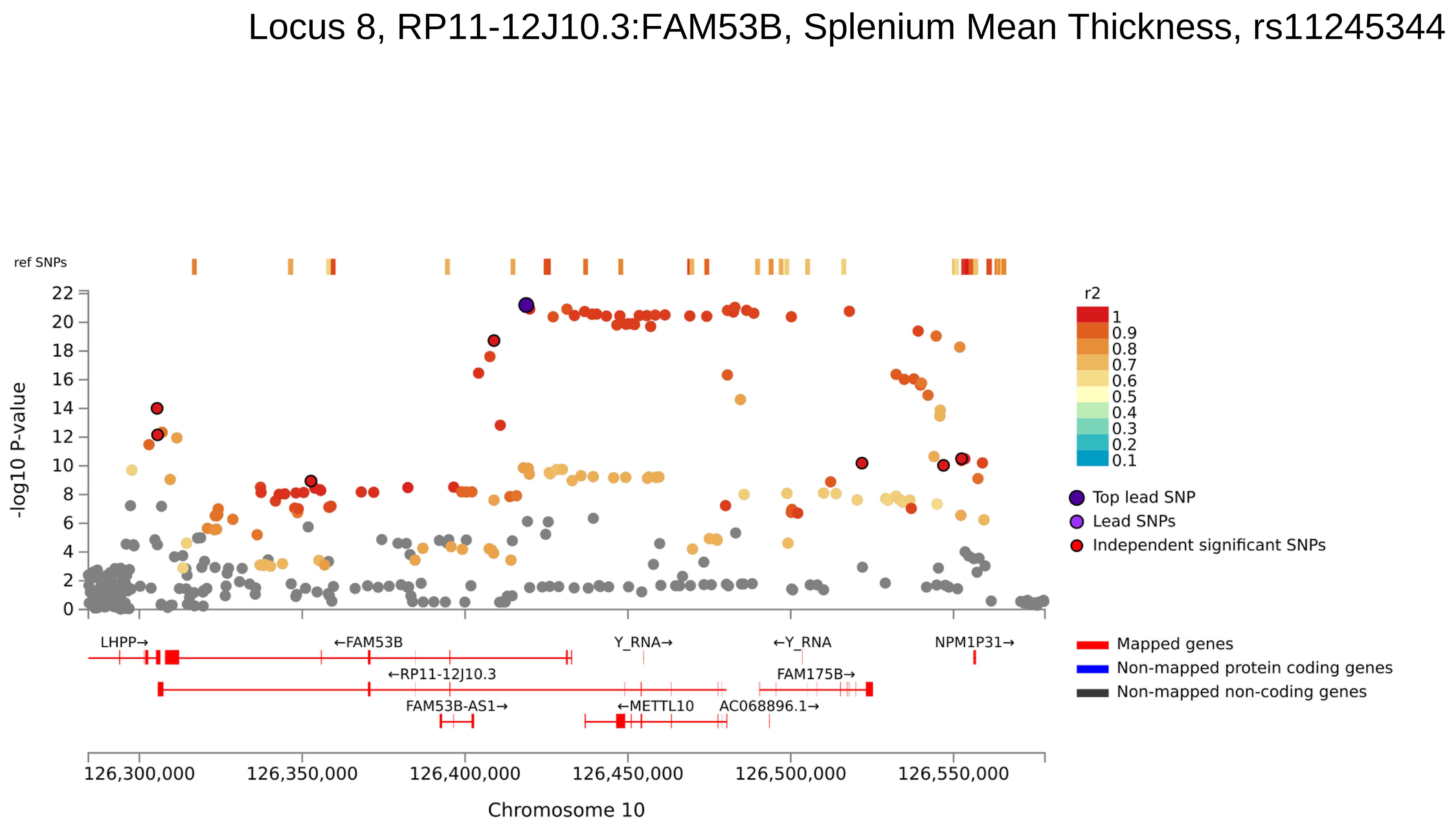
Locus 8, RP11-12J10.3:FAM53B, Splenium Mean Thickness, rs11245344
ref SNPs
-log10 P-value
r2
Top lead SNP
Lead SNPs
Independent significant SNPs
LHPP→
←FAM53B
Y_RNA→
←Y_RNA
NPM1P31→
←RP11-12J10.3
FAM175B→
FAM53B-AS1→
←METTL10
AC068896.1→
Mapped genes
Non-mapped protein coding genes
Non-mapped non-coding genes
126,300,000
126,350,000
126,400,000
126,450,000
126,500,000
126,550,000
Chromosome 10

Locus 9, RRAS2, Splenium Mean Thickness, rs35247669

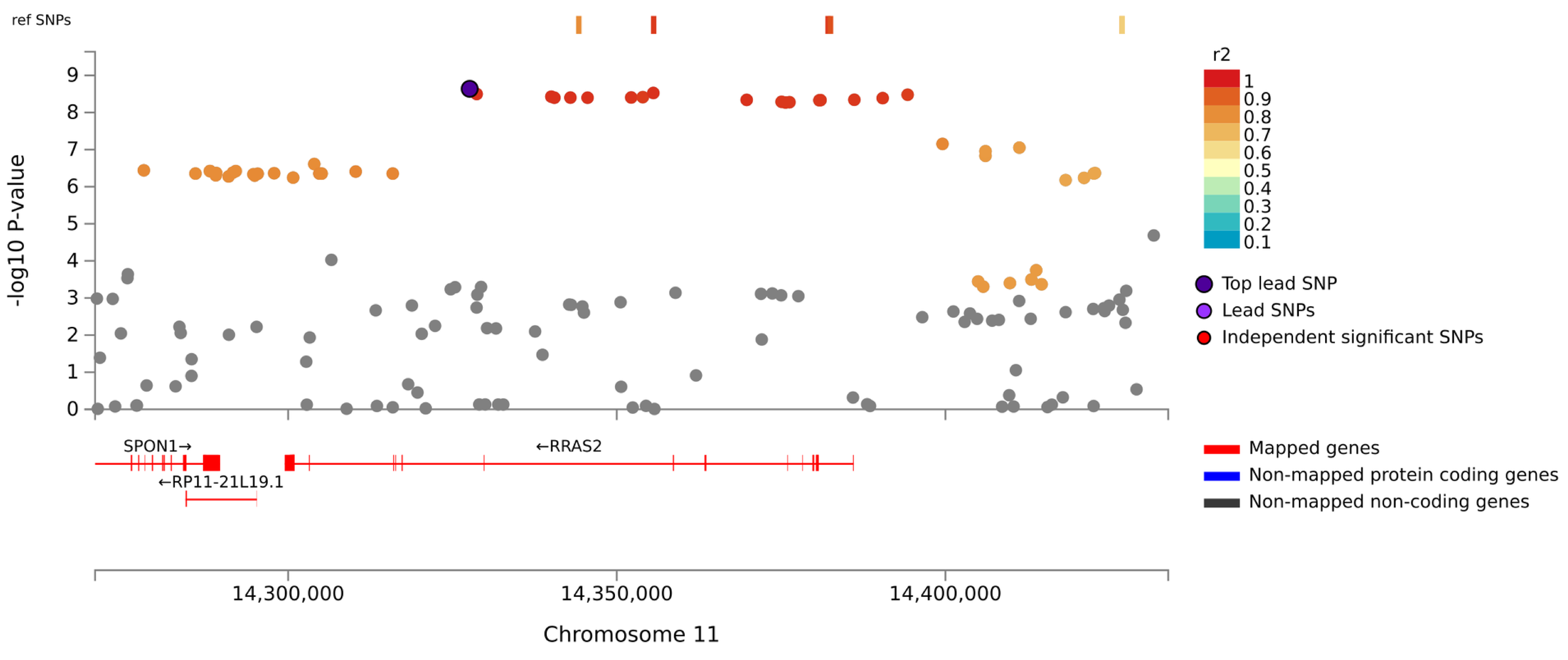

Supplement: Supplementary file 4 — Supplementary Data 42 [file 41467_2025_64791_MOESM4_ESM.pdf]
