# Supplementary material for: The Genetic Architecture of the Human Corpus Callosum and its Subregions
Source: Nat Commun. 2025 Nov 4;16:9708. doi: 10.1038/s41467-025-64791-3 (PMC12586663; doi:10.1038/s41467-025-64791-3)
Supplement: Supplementary file 5 — Reporting Summary [file 41467_2025_64791_MOESM5_ESM.pdf]

nature portfolio

Corresponding author(s): Ravi R. Bhatt
Neda Jahanshad

Last updated by author(s): Aug 3, 2025

Reporting Summary

Nature Portfolio wishes to improve the reproducibility of the work that we publish. This form provides structure for consistency and transparency in reporting. For further information on Nature Portfolio policies, see our Editorial Policies and the Editorial Policy Checklist.

Statistics

For all statistical analyses, confirm that the following items are present in the figure legend, table legend, main text, or Methods section.

n/a	Confirmed	
☐	☒	The exact sample size (*n*) for each experimental group/condition, given as a discrete number and unit of measurement
☐	☒	A statement on whether measurements were taken from distinct samples or whether the same sample was measured repeatedly
☐	☒	The statistical test(s) used AND whether they are one- or two-sided<br>*Only common tests should be described solely by name; describe more complex techniques in the Methods section.*
☐	☒	A description of all covariates tested
☐	☒	A description of any assumptions or corrections, such as tests of normality and adjustment for multiple comparisons
☐	☒	A full description of the statistical parameters including central tendency (e.g. means) or other basic estimates (e.g. regression coefficient) AND variation (e.g. standard deviation) or associated estimates of uncertainty (e.g. confidence intervals)
☐	☒	For null hypothesis testing, the test statistic (e.g. *F*, *t*, *r*) with confidence intervals, effect sizes, degrees of freedom and *P* value noted<br>*Give P values as exact values whenever suitable.*
☒	☐	For Bayesian analysis, information on the choice of priors and Markov chain Monte Carlo settings
☒	☐	For hierarchical and complex designs, identification of the appropriate level for tests and full reporting of outcomes
☐	☒	Estimates of effect sizes (e.g. Cohen's *d*, Pearson's *r*), indicating how they were calculated

Our web collection on statistics for biologists contains articles on many of the points above.

Software and code

Policy information about availability of computer code

Data collection	No special software was used for data collection
Data analysis	The code and model used to extract the corpus callosum and its metrics is available at https://github.com/USC-LoBeS/smacc/.

For manuscripts utilizing custom algorithms or software that are central to the research but not yet described in published literature, software must be made available to editors and reviewers. We strongly encourage code deposition in a community repository (e.g. GitHub). See the Nature Portfolio guidelines for submitting code & software for further information.

Data

Policy information about availability of data

All manuscripts must include a data availability statement. This statement should provide the following information, where applicable:

- Accession codes, unique identifiers, or web links for publicly available datasets
- A description of any restrictions on data availability
- For clinical datasets or third party data, please ensure that the statement adheres to our policy

This work is a meta-analysis. Upon publication, the full meta-analytic summary statistics will be made available in ENIGMA-Vis.

Research involving human participants, their data, or biological material

Policy information about studies with human participants or human data. See also policy information about sex, gender (identity/presentation), and sexual orientation and race, ethnicity and racism.

Reporting on sex and gender	Sex and Age*sex were used as a covariate in all analyses. From the UK Biobank, "sex was acquired from central registry at recruitment, but in some cases updated by the participant. Hence this field may contain a mixture of the sex the NHS had recorded for the participant and self-reported sex." From ABCD, sex was defined by the question "What sex was the child assigned at birth, on the original birth certificate?" In the UK Biobank, there were 20,262 males and 21,717 females. In ABCD, there were 2,678 males and 2,129 females. Analyses specific to sex was not performed not only to maximize power, but to make sure results were generalizable.
Reporting on race, ethnicity, or other socially relevant groupings	Ethnicity was determined using solely via genetics in the current study. We used multidimensional scaling of individual level genetic data to determine ethnicity. The mean and standard deviations of the first and second genetic components of individuals who were classified as Utah residents with Northern and Western European ancestry from the CEPH collection (CEU) from the HapMap 3 release were then calculated. Individuals in UKB and ABCD who were within a distance of 0.0101 on genetic principal components 1 and 2 were classified as of European white ancestry. All other individuals were classified as "non-European" in the current study. The first 10 genetic principal components were included as covariates in the GWAS analyses to control for any effect of ethnicity.
Population characteristics	Participants in the UK Biobank were aged between 40-69 years, with a mean age around 56 years. There is a roughly even split between males (46%) and females (54%). The vast majority of the participants are of white British ethnicity, but representation of minority groups is also included. Participants tend to be more highly affluent and more well-educated than the general UK population. Urban areas are more highly represented than rural ones. Participants in the ABCD study were between 9-10 years old at the time of recruitment. This cohort is roughly balanced between males (51%) and females (49%). The ABCD cohort is designed to reflect the racial and ethnic diversity of the U.S. population. Approximately 51% of participants are White, 20% Hispanic, 16% Black, and 8% Asian, with the remainder identifying as other or mixed races. The study aims to represent a broad range of socioeconomic classes, but there is a slight under-representation of the lowest income brackets compared to the US population. The study was designed to include individuals of a rich geographic diversity including urban, suburban and rural areas.
Recruitment	The UK Biobank recruited approximately 500,000 individuals aged between 40-69 years form across the United Kingdom. Invitations were sent out to individuals who were registered with the National Health Service (NHS) and lived within a certain distance of one of the 22 assessment centers. Participation was voluntary. There was no direct financial incentive. Potential self-selection biases include individuals of higher social economic status, and those who have the means and interest to participate in such a deep-phenotyping study. The ABCD study recruitment was carried out through school-based sampling, while trying to achieve a well-rounded representation of the US population in terms of sex, race/ethincity, and socioeconomic status with a sample of over 11,800. Potential sources self-selection biases include parental consent and engagement, where families more invested and concerned about their child's health may be overrepresented. School selection is another potential source of self-selection bias, as there could still be biases introduced by the types of schools willing to participate or the availability of children during recruitment.
Ethics oversight	From UK Biobank: "UK Biobank has approval from the North West Multi-centre Research Ethics Committee (MREC) as a Research Tissue Bank (RTB) approval. This approval means that researchers do not require separate ethical clearance and can operate under the RTB approval (there are certain exceptions to this which are set out in the Access Procedures, such as re-contact applications)." All researchers had approval to use the UK Biobank data via Resource Application No. 11559.<br><br>All authors have a data use agreement with the National Institute of Mental Health (NIMH) Data Archive (NDA) for the ABCD study. Institutional sponsorship from the University of Southern California was approved under application number 17367.

Note that full information on the approval of the study protocol must also be provided in the manuscript.

Field-specific reporting

Please select the one below that is the best fit for your research. If you are not sure, read the appropriate sections before making your selection.

☒ Life sciences ☐ Behavioural & social sciences ☐ Ecological, evolutionary & environmental sciences

For a reference copy of the document with all sections, see nature.com/documents/nr-reporting-summary-flat.pdf

Life sciences study design

All studies must disclose on these points even when the disclosure is negative.

Sample size	The GWAS meta-analysis of CC morphometry was conducted in 46,685 individuals of European ancestry, and replicated in 7,040 individuals of Non-European ancestry. No sample size calculation was made prior to this study.
Data exclusions	All individuals with a T1 weighted brain MRI in the UK Biobank and the Adolescent Brain Cognitive Development study were included.
Replication	We chose to include all individual samples in one large GWAS meta-analysis instead of opting for the discovery-replication approach. This apraoch has been shown to maximize power and is the more sensible approach for GWAS studies as explained in the methods. We

evaluated the consistency of the results across cohorts which are shown in the Supplementary Tables. We show consistency in results across cohorts on all genomic loci.

Randomization	Randomization is not applicable for this study as there were no case/control study design or no randomization needed for interventions.
Blinding	There was no group allocation so study blinding is not relevant in that manner. All MRI, genotype and clinical data from both population based studies were anonymized for HIPPA sensitive participant information which could obtain personally identifiable information.

Reporting for specific materials, systems and methods

We require information from authors about some types of materials, experimental systems and methods used in many studies. Here, indicate whether each material, system or method listed is relevant to your study. If you are not sure if a list item applies to your research, read the appropriate section before selecting a response.

Materials & experimental systems

n/a	Involved in the study	
☒	☐	Antibodies
☒	☐	Eukaryotic cell lines
☒	☐	Palaeontology and archaeology
☒	☐	Animals and other organisms
☒	☐	Clinical data
☒	☐	Dual use research of concern
☒	☐	Plants

Methods

n/a	Involved in the study	
☒	☐	ChIP-seq
☒	☐	Flow cytometry
☐	☒	MRI-based neuroimaging

Plants

Seed stocks	N/A
Novel plant genotypes	N/A
Authentication	N/A

Magnetic resonance imaging

Experimental design

Design type	No task was used in the MRI scanner for this study.
Design specifications	No task was used in the MRI scanner for this study.
Behavioral performance measures	No task was used in the MRI scanner for this study.

Acquisition

Imaging type(s)	Structural
Field strength	3 Tesla
Sequence & imaging parameters	All UKB participants completed a 31-minute neuroimaging protocol using a Siemens Skyra 3 Tesla scanner and a 32-channel head coil in one of three MRI scanning locations. All 3D structural T1-weighted brain scans were acquired using the following parameters: 3D MPRAGE, sagittal orientation, in-plane acceleration factor = 2, TI/TR = 880/2000 ms, voxel resolution = 1 x 1 x 1 mm, acquisition matrix = 208 x 256 x 256 mm. All scans were pre-scan normalized using an on-scanner bias correction filter. More details of the imaging protocols may be found in the following reference papers.<br><br>All ABCD participants completed a neuroimaging protocol in one of three scanner types at 21 different sites. The Siemens Prisma had the following parameters for the T1-weighted scans: TI/TR = 1060/2500 ms, TE = 2.88 ms, voxel resolution = 1 x 1 x 1 mm, acquisition matrix = 176 x 256 x 256, flip angle = 8 degrees. The Philips Achieva Ingenia had a TI/TR = 1060/6.31 ms, voxel resolution = 1 x 1 x 1 mm, acquisition matrix = 225 x 256 x 256 mm and a flip angle = 8 degrees. The GE MR750 had a TI/TR = 1060/2500 ms, TE = 2 ms, voxel resolution = 1 x 1 x 1 mm, acquisition matrix =

	208 x 256 x 256, and a flip angle = 8 degrees.
Area of acquisition	Whole-brain

Diffusion MRI ☐ Used ☒ Not used

Preprocessing

Preprocessing software	No preprocessing software was used other than FSL for normalization to the MNI152 Template.
Normalization	All T1w MRIs were registered to MNI152 1mm space with 6 degrees of freedom using FSL's flirt command.
Normalization template	All T1w MRIs were registered to MNI152 1mm space with 6 degrees of freedom using FSL's flirt command.
Noise and artifact removal	No noise and artifact removal was completed as raw T1 scan was used as input for SMACC.
Volume censoring	No volume censoring was conducted as entire T1 scan was used as input for SMACC.

Statistical modeling & inference

Model type and settings	N/A
Effect(s) tested	N/A

Specify type of analysis: ☐ Whole brain ☒ ROI-based ☐ Both

Anatomical location(s)	Extraction of the corpus callosum and it's segmentation was completed with our newly devleoped artificial intelligence based tool SMACC. Detailed explanation of it's development and use is in the methods of the manuscript. The code is also publicly available on GitHub at https://github.com/USC-LoBeS/smacc/
Statistic type for inference (See Eklund et al. 2016)	No cluster type statistical inferences were done with MRI data in this study.
Correction	No correction is applicable for the MRI data alone, but all data within the GWAS analysis was corrected using the Bonferroni approach.

Models & analysis

n/a	Involved in the study
☒	☐ Functional and/or effective connectivity
☒	☐ Graph analysis
☒	☐ Multivariate modeling or predictive analysis
